# Glucocorticoid resistance in T-lineage acute lymphoblastic leukaemia is associated with a proliferative metabolism

**DOI:** 10.1038/sj.bjc.6605072

**Published:** 2009-05-12

**Authors:** A H Beesley, M J Firth, J Ford, R E Weller, J R Freitas, K U Perera, U R Kees

**Affiliations:** 1 Division of Children's Leukaemia and Cancer Research, Telethon Institute for Child Health Research, University of Western Australia Centre for Child Health Research, West Perth, Western Australia, PO Box 855, Australia 6872, Australia; 2 Division of Biostatistics and Genetic Epidemiology, Telethon Institute for Child Health Research, University of Western Australia Centre for Child Health Research, West Perth, Western Australia, PO Box 855, Australia 6872, Australia

**Keywords:** drug resistance, lymphoid tumours, glucocorticoids, gene expression

## Abstract

Glucocorticoids (GCs) are among the most important drugs for acute lymphoblastic leukaemia (ALL), yet despite their clinical importance, the exact mechanisms involved in GC cytotoxicity and the development of resistance remain uncertain. We examined the baseline profile of a panel of T-ALL cell lines to determine factors that contribute to GC resistance without prior drug selection. Transcriptional profiling indicated GC resistance in T-ALL is associated with a proliferative phenotype involving upregulation of glycolysis, oxidative phosphorylation, cholesterol biosynthesis and glutamate metabolism, increased growth rates and activation of PI3K/AKT/mTOR and *MYC* signalling pathways. Importantly, the presence of these transcriptional signatures in primary ALL specimens significantly predicted patient outcome. We conclude that in lymphocytes the activation of bioenergetic pathways required for proliferation may suppress the apoptotic potential and offset the metabolic crisis initiated by GC signalling. It is likely that the link between GC resistance and proliferation in T-ALL has not been fully appreciated to date because such effects would be masked in the context of current multiagent therapies. The data also provide the first evidence that altered expression of wild-type *MLL* may contribute to GC-resistant phenotypes. Our findings warrant the continued development of selective metabolic inhibitors for the treatment of ALL.

In children with acute lymphoblastic leukaemia (ALL) cellular drug sensitivity is a major component of clinical outcome. Event-free survival for these patients is now greater than 75% but a significant number continue to relapse and the outlook for these is dismal (Gaynon, 2005). Around 10 different drugs are currently used in paediatric ALL-treatment protocols but among the most important are the glucocorticoids (GCs). Early response to prednisolone is one of the most informative prognostic factors for infant, childhood and adult ALL, with patients that respond well showing significantly better outcome (Dordelmann *et al*, 1999). Moreover, GC resistance is a well-documented feature of relapse (Klumper *et al*, 1995; Kaspers *et al*, 2005). Among the paediatric ALL subtypes, infants and those with T-lineage ALL are particularly resistant to GCs (Pieters *et al*, 1998).

Despite the clinical importance of this class of drug, the exact mechanisms involved in GC cytotoxicity and the development of resistance remain uncertain (Schmidt *et al*, 2006; Tissing *et al*, 2007). Mutations in the GC receptors (GRs) are commonly found in cell lines that have been selected for GC resistance by extended exposure to high drug concentrations, but are rarely found in primary ALL specimens and are not thought to contribute significantly to GC resistance in patients (Irving *et al*, 2005; Tissing *et al*, 2005). In lymphoid tumours, the ultimate effect of GC exposure is activation of apoptosis but it is important to remember that this differs from many other cell types where GCs in fact promote cell survival and proliferation (Zhang *et al*, 2007). It has been hypothesised that the phenomenon of GC cytotoxicity in lymphoid tissues is related to the repression of essential metabolic pathways in these cells (Tonko *et al*, 2001), indicating that there may be a distinctive metabolic phenotype associated with this tissue type. Examples of metabolic features that are specific to leukaemia cells include the unusual dependencies on endogenously synthesised cholesterol (Madden *et al*, 1986) and exogenously supplied asparagine (Richards and Kilberg, 2006), the latter forming the basis for the successful inclusion of L-asparaginase as a cornerstone of chemotherapy for ALL.

Over the past 20 years, our laboratory has developed an authenticated panel of paediatric ALL cell lines that have been grown in the absence of drug selection (Kees *et al*, 2003; Beesley *et al*, 2006). We have previously demonstrated that these cultures retain critical features of the primary disease and that their drug-resistance profile parallels the spectrum of resistance that has been observed in primary patient specimens, particularly in regards to dexamethasone (DEX; Kees *et al*, 2003; Beesley *et al*, 2006). Here we have performed gene-expression profiling of T-ALL cell lines within this panel to identify biological pathways involved in the development of GC resistance, and have examined their relevance for the prediction of clinical outcome using microarray data obtained from ALL patient specimens at the time of diagnosis.

## Materials and methods

### Cell lines

The cell line panel has been previously described (Kees *et al*, 2003; Beesley *et al*, 2006, 2007) and comprised nine T-ALL lines derived in our own laboratory (PER cell lines), plus six additional T-ALL cell lines obtained from external sources (HSB2, CEM, JURKAT, ALL-SIL, MOLT4, DU.528). PER cell lines were derived from paediatric ALL bone marrow specimens according to the method previously described (Kees *et al*, 1987). DNA fingerprinting confirmed the identity of each of the cell lines (Beesley *et al*, 2006).

### *In vitro* drug resistance

The sensitivity of the T-ALL cell lines to methylprednisolone (MPRED) and DEX has been previously published (Beesley *et al*, 2006) and was measured over 4 days. The IC50 (drug concentration that inhibits cell growth by 50%) was used as the measure of drug resistance. For testing of drugs identified by Connectivity Map (CMAP) analysis (quercetin, resveratrol, LY294002, rapamycin), the neutral red assay was used because antioxidants have been shown to interfere with the accuracy of the MTT assay (see Supplementary Methods). For serum starvation experiments, cells were incubated in normal tissue culture media containing either 10% or 1% fetal calf serum for 7 days before assessing drug sensitivity as described above.

### Gene-expression profiling

Briefly, RNA was extracted from cell lines in exponential growth phase and hybridised to Affymetrix HG-U133A microarrays (Affymetrix, Santa Clara, CA, USA) in accordance with our previously published protocols (Beesley *et al*, 2005; Hoffmann *et al*, 2005; Gottardo *et al*, 2007). Microarray data were normalised using robust multiarray analysis (Irizarry *et al*, 2003) and all passed quality control criteria for noise, background, absent/present calls and 3′/5′ signal ratios for *ACTB* and *GAPDH*.

### Analysis of drug–gene relationships

T-ALL cell line IC50 scores and gene-expression values were log_2_ transformed before performing Gene Set Enrichment Analysis (GSEA; http://www.broad.mit.edu/gsea) as described (Subramanian *et al*, 2005) for MPRED and DEX separately, using Pearson's correlation as metric (10 000 permutations). Because not all genes within a given biological pathway are regulated in the same direction, analyses were performed using absolute correlation values. Gene Set Enrichment Analysis gene set databases (C1, C2, C3, C4) were downloaded from the GSEA website (GSEA v2.0, May 2006), whereas the ICHR Curated database was compiled using published gene lists (see Results section and Supplementary Table S1).

### Connectivity Map analysis

The top 100 positive and top 100 negative probe sets correlating with MPRED or DEX IC50 values were analysed using the CMAP software (http://www.broad.mit.edu/cmap). This tool identifies compounds that elicit transcriptional responses with similarities to the gene signature under test, and thus with potential for synergistic activity. Compounds significantly associated with GC-resistance signatures were identified using the CMAP permuted output. Drugs that demonstrated significant enrichment scores against both DEX and MPRED were tested for cytotoxicity against ALL cell lines in the presence and absence of DEX (see above).

### Outcome prediction using *MPRED* gene sets

Gene Set Enrichment Analysis gene sets associated with MPRED resistance were modelled for their ability to predict clinical outcome using previously published microarray data from 59 B-lineage and 17 T-ALL primary paediatric ALL diagnostic bone marrow specimens (Beesley *et al*, 2005). For this we used the leading edge gene subsets from the top 20 gene sets in each database (C2, C3, C4 and ICHR Curated), that is a total of 80 gene sets, each of which represents a particular biological feature, process or pathway associated with *in vitro* MPRED resistance. For each of these gene sets, we first determined their equivalent levels of expression in the diagnostic patient specimens and then performed principal component analysis (PCA) to reduce the dimensionality of the data (B-lineage and T-ALL analysed separately). The first three principal components from each gene set were then used in logistic regression to generate a binary classification of predicted outcomes (i.e., relapse or nonrelapse) for the two patient cohorts. The log-rank statistics produced from these initial analyses were found to be highly (artificially) significant in almost all cases, an artefact of data overfitting by PCA and logistic regression. To control for this, the data were re-analysed 999 times using random permutations of the patient outcome labels to generate true (permuted) *P*-values for each gene set. Kaplan–Meier analyses to assess differences in relapse-free survival were generated using permutation testing to generate *P*-values for each gene set.

## Results

### Gene-expression signatures associated with GC resistance

The resistance profile of the 15 T-ALL cell lines to the GCs DEX and MPRED has been previously described (Beesley *et al*, 2006). IC50 values across the panel varied by 4–5 orders of magnitude, with lines demonstrating high (JURKAT, CEM, MOLT4, DU.528), intermediate (HSB2, ALL-SIL, PER-117, PER-487, PER-537) and low (PER-427, PER-604, PER-608, PER-255, PER-550, PER-606) GC resistances, based on IC50 ranges measured for MPRED of 0.019–500 *μ*g ml^−1^ and for DEX of 0.0014–500 *μ*g ml^−1^ (Beesley *et al*, 2006). We have documented that this naturally occurring spectrum of resistance cannot be explained by mutations in the GR or variations in GR*α* or GR*β* expression levels (Beesley *et al*, 2008), indicating that defects downstream of the GR are primarily responsible for GC-resistant phenotypes in these cell lines.

The resistance profiles of MPRED and DEX across the panel (IC50 scores) were correlated with gene-expression profiles as determined by HG-U133A microarray. The top 20 probe sets positively or negatively correlating with GC resistance are listed in Table 1 for both drugs, and extended list in Supplementary Tables S2 and S3. Among the top 20 probe sets there were four genes common to both drugs, namely *MLL, SOX14, NMT2* and *FLJ13769*; among the entire chip there were 550 probe sets that had a significant correlation (*P*<0.01) *vs* both drugs (combined *P*-value *P*<0.0001). Of the genes listed in Table 1, we have previously identified *OPHN1* and *NF1* as part of a gene signature that can distinguish diagnosis and relapse specimens from paediatric T-ALL patients (Beesley *et al*, 2005). Because increased GC resistance is one of the hallmarks of relapse in ALL, this provides encouraging evidence that the GC-resistance signature derived from this *in vitro* model is relevant to the *in vivo* situation.

### Biological pathways associated with GC-resistance signatures

**Figure 1:**
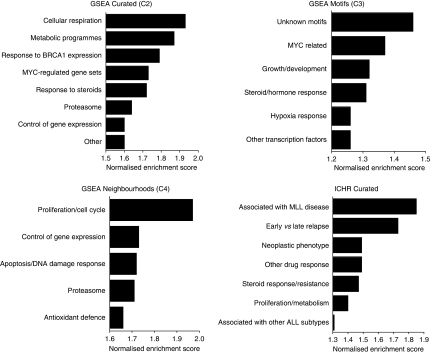
Functional categories represented by the top GSEA gene sets associated with MPRED resistance in T-ALL cell lines. The top 20 gene sets from (**A**) GSEA Curated (**B**) ICHR Curated (**C**) GSEA Gene Neighbourhood, and (**D**) GSEA Motif databases were grouped into biological categories. Bars indicate the highest normalised enrichment score (NES) achieved for gene sets within each category.

To interrogate the biological pathways represented by GC-resistance signatures, we used GSEA (Subramanian *et al*, 2005). Gene Set Enrichment Analysis examines ranked lists of genes for enrichment of biological pathways contained within four different databases: C1 (genomic loci), C2 (curated Biological pathways), C3 (genes containing *cis*-regulatory motifs) and C4 (computational gene networks). To incorporate pathway information specifically related to the biology of leukaemia, we manually curated an additional database (ICHR Curated database, Supplementary Table S1) using information from 40 ALL-related publications that were not captured by the GSEA databases, and included gene networks generated using the STRING algorithm (http://string.embl.de) for 63 genes known to be important in the biology of ALL resulting in 512 gene sets (Supplementary Data File 1). The output from this GSEA is a ranked list of biological gene sets for each database that are enriched in the MPRED- and DEX-resistance signatures. The biological pathways identified from this analysis were highly similar for both GCs, but were typically more robust, that is were associated with lower false discovery rates (FDRs) and *P*-values for MPRED than for DEX (full details in Supplementary Data Files 2 and 3). In view of this, further analysis was focused on the MPRED signature. A graphical summary of the biological categories most significantly enriched in the MPRED signature using the C2, C3, C4 and ICHR Curated databases is shown in Figure 1. The top-ranked gene sets associated with MPRED resistance from the C2 and ICHR Curated databases can be found in Table 2 (for full details see Supplementary Data File 2). The predominant pathways associated with MPRED resistance were those involving cellular respiration (oxidative phosphorylation, the electron transport chain and antioxidant defence), metabolic programs (starvation signalling, responses to rapamycin, glycolysis and gluconeogenesis, cholesterol biosynthesis, steroid biosynthesis), proliferation and regulation by the gene *MYC*. As one would expect, several gene sets were also identified that are involved in the cellular response to GCs or other drugs. Notably, this includes a previously described gene signature associated with GC resistance in primary ALL specimens (Wei *et al*, 2006) (Table 2, ICHR Curated gene set no. 7). Several gene sets were identified that are differentially expressed between early and late relapse in childhood ALL, an observation that reinforces the link between GC resistance and aggressive disease. In accordance with the observation that the *MLL* gene was highly correlated to both MPRED and DEX resistance (Table 1), several gene sets were derived from work studying gene-expression signatures associated with *MLL* translocations. No significantly enriched gene sets were identified using the C1 (Positional) database in this study for MPRED or DEX, indicating that genes associated with GC resistance were not confined to particular genomic loci.

In addition to identifying the biological pathways that are enriched within gene-expression data, the GSEA algorithm determines which genes from these pathways contribute most strongly to the observed phenotype. These are referred to as the ‘leading edge’ genes and those found to be associated with MPRED resistance are listed in Supplementary Data File 4 along with the correlation of each gene *vs* MPRED IC50 values in the T-ALL cell lines. It was noticeable that the pathways identified by GSEA were predominately upregulated, as evidenced by an overwhelming majority of genes in each set showing positive correlation of expression with MPRED IC50. This cannot be attributed to a bias towards detection of upregulated genes in the microarray data because upregulated and downregulated genes were equally associated with GC resistance when correlated individually (see Table 1 and Supplementary Tables S2 and S3). Thus, transcriptional activation appears to be a stronger phenotype of GC resistance in this model than transcriptional repression. Notable exceptions to this trend were genes that have previously been shown to be downregulated in GC-resistant ALL specimens (Wei *et al*, 2006); in the present study, downregulation of these genes was also associated with GC resistance (Supplementary Data File 4).

### GC resistance and cell metabolism

One of the most prominent features associated with GC resistance in T-ALL in the present study involved upregulation of metabolic pathways. Closer inspection of these data revealed a compelling pattern of gene regulation associated with these changes (Supplementary Data File 4). In the C2 data set ∼90% of the leading edge genes associated with mitochondrial processes were positively correlated with MPRED IC50, indicating a general upregulation of mitochondrial metabolism in resistant cells. This included a large number of genes involved in the electron transport and ATP synthetase complexes, as well as most of the major enzymes involved in the citric acid cycle. Likewise, leading edge gene sets contained almost complete representation of genes driving glycolysis and cholesterol biosynthesis. Of particular interest, expression levels of the GLUT1 glucose transporter, ATP citrate lyase, a critical enzyme in tumours that links glucose metabolism to lipid synthesis and hexokinase II, responsible for coordinating metabolic and apoptotic pathways at the mitochondrial membrane were significantly correlated with GC resistance (*P*<0.005 *vs* both DEX and MPRED for each gene).

Analysis of the small number of genes downregulated in GC-resistant cells revealed a complementary biological pattern. These included *CROT*, *CRAT* and *SLC25A20* (carnitine octanoyltransferase, carnitine acetyltransferase and carnitine-acylcarnitine transferase, which are important in the transport of fatty acids for mitochondrial *β*-oxidation); *PDK3* (pyruvate dehydrogenase kinase, responsible for inhibition of pyruvate dehydrogenase, the enzyme that provides the primary link between glycolysis and the tricarboxylic acid cycle by catalyzing the conversion of pyruvate into acetyl-CoA); *LCAT* (lecithin-cholesterol acyltransferase, which facilitates the export of cholesterol out of the cell); *DBT* (dihydrolipoamide branched-chain transacylase, involved in branched-chain amino-acid catabolism); and *GLUL* (glutamine synthetase). These observations are consistent with an alteration in amino-acid metabolism and the downregulation of fatty acid *β*-oxidation. Together with the enrichment of gene sets associated with cell cycle, transcription, translation, *MYC* regulation and proteasomal pathways (Figure 1, and Supplementary Data Files 2 and 3) the signatures are consistent with activation of biosynthetic and metabolic pathways to support a proliferative phenotype in resistant cells.

### GC resistance is associated with faster growth rate

**Figure 2:**
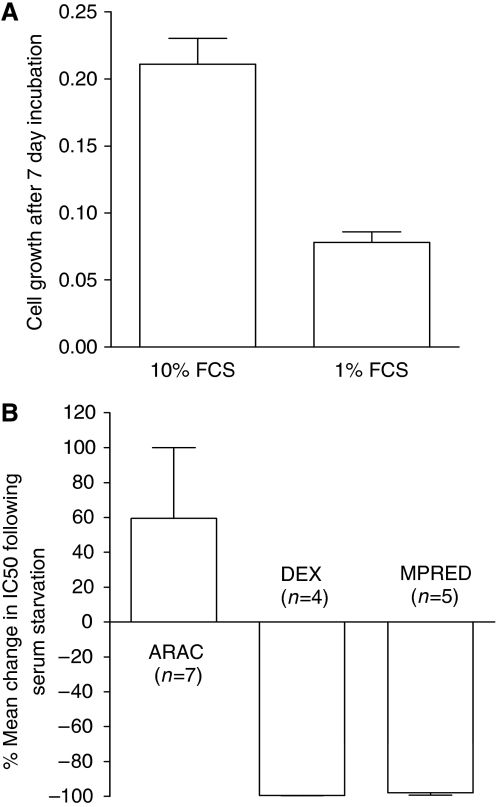
Effect of serum starvation on drug sensitivity in HSB2 cells. Cells were incubated in tissue culture medium containing either 10% or 1% fetal calf serum (FCS) for 7 days before measuring drug sensitivity. (**A**) Growth inhibitory effects of serum starvation after 7 days (arbitrary units, mean±s.e.m., *n*=9). (**B**) Cytotoxic effects of ARAC, DEX and MPRED (MTT assay, 4-day drug incubation). Data represent the mean percentage change in IC50 in serum-starved cells (1%) compared to control (10%) in 4–7 independent experiments.

To confirm the link between GC resistance and proliferation, we examined cell line doubling times (Beesley *et al*, 2006) and found a significant correlation with both MPRED (Spearman's *r* −0.86, *P*<0.001) and DEX IC50 values (Spearman's *r* −0.65, *P*<0.01), see Supplementary Figure S1, with the fastest-growing lines being the most resistant. To assess this relationship further, we used serum starvation to inhibit the growth of HSB2 cells and measured the effect on sensitivity to DEX, MPRED and the cell-cycle-dependent drug cytarabine (ARAC). Serum starvation in 1% *vs* 10% fetal calf serum for 7 days inhibited the growth rate of HSB2 cells by 63% (Figure 2A) and increased resistance to ARAC as expected (Figure 2B). In contrast, resistance to both DEX and MPRED significantly decreased following serum starvation (Figure 2B) consistent with the hypothesis that proliferation rate is a contributing factor in determining GC sensitivity.

### Prognostic relevance of pathways associated with GC resistance

**Figure 3:**
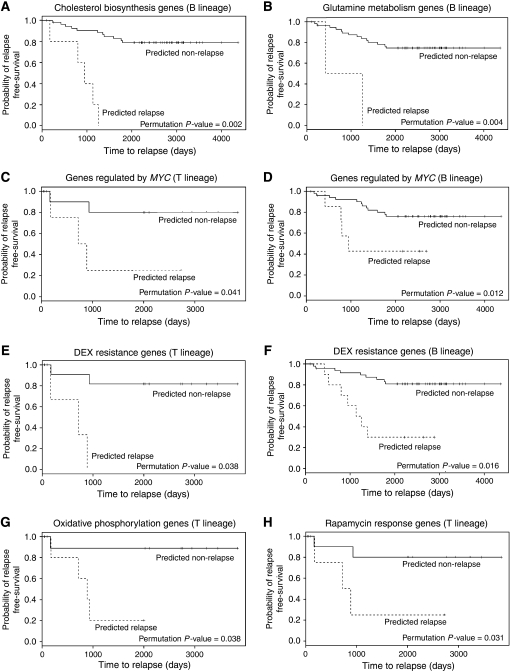
Prognostic significance of leading edge gene sets associated with MPRED resistance in T-ALL cell lines. Graphs show relapse-free survival in primary ALL patient cohorts (T lineage and B lineage as indicated) predicted based on expression of genes associated with MPRED resistance in T-ALL cell lines. The gene profiles represent the leading edges of the following GSEA gene sets: (**A**) CHOLESTEROL BIOSYNTHESIS; (**B**) STRING GLUL TOP100 ASSOCIATIONS (genes associated with glutamine synthetase); (**C** and **D**) V$MYC_Q2 (genes regulated by *MYC*); (**E** and **F**) WEI_DOWN_IN_DEX_RESISTANCE (genes associated with DEX resistance); (**G**) VOXPHOS (oxidative phosphorylation); (**H**) PENG_RAPAMYCIN_DOWN (genes downregulated in response to rapamycin). Further details of these gene sets can be found in Supplementary Table S4 and Supplementary Data File 4.

To investigate whether the gene sets associated with GC resistance in T-ALL cell lines *in vitro* might have prognostic relevance for patients at the time of diagnosis, we examined microarray data obtained during a previous study of diagnostic childhood ALL specimens (Beesley *et al*, 2005). We modelled the expression of the leading edge MPRED genes (Supplementary Data File 4) in this patient data set and assessed the ability of each gene set to predict relapse. Gene sets that significantly predicted relapse in T-ALL (*n*=17) or B-lineage (*n*=59) patient specimens are listed in Supplementary Table S4. Although the gene signatures used in this analysis were generated using T-ALL cell lines, several gene sets could predict patient relapse in both B-lineage and T-ALL patient cohorts, including those involved with cholesterol biosynthesis (*P*<0.005, Figure 3A, and *P*<0.05 Supplementary Table S4, respectively) those associated with *GLUL* (*P*<0.005, Figure 3B, and *P*<0.05. Supplementary Table S4, respectively), and those regulated by *MYC* (*P*<0.05 *vs* both lineages, Figures 3C and D). Gene signatures previously associated with DEX resistance in primary ALL specimens (Wei *et al*, 2006) also significantly predicted outcome in both cohorts (*P*<0.05, Figures 3E and F). This indicates that many of the biological processes contributing to GC resistance in ALL may be independent of lineage. In contrast, gene sets involved with oxidative phosphorylation (*P*<0.05, Figure 3G) and the cellular response to rapamycin (*P*<0.05, Figure 3H) only predicted relapse in the T-ALL cohort indicating that these pathways may have particular relevance for GC resistance in this lineage.

### Canonical GC-resistance mechanisms in T-ALL cell lines

Aside from abnormalities in the activation or functionality of the GR, known resistance mechanisms for GCs include defects in apoptosis (Erlacher *et al*, 2005) and altered expression of metabolising enzymes (e.g., 11*β*-hydroxyglucocorticoid dehydrogenase) or multidrug-resistance pumps. To directly assess the contribution of these mechanisms to GC resistance in our T-ALL cell lines, we correlated the expression of 142 genes curated from the literature (corresponding to 253 probe sets) involved in canonical mechanisms of multidrug-resistance, apoptosis and survival signalling with MPRED and DEX resistance (Supplementary Table S5). Genes significantly correlated with both MPRED and DEX resistance included *DCK* (deoxycytidine kinase; downregulated), *CYP3A4* (downregulated), *CCND1* (Cyclin D1; upregulated) and the multidrug transporter *ABCC9* (upregulated). In contrast to previous work reporting an association between prednisolone sensitivity and expression of *MCL1* or *DAPK1* in ALL patient specimens (Holleman *et al*, 2005; Wei *et al*, 2006), we found no correlation of these genes with GC resistance in T-ALL cell lines. Neither was there significant correlation with *BIM*, *PUMA* or *BCL-2* that have been shown to be key initiators of both GC and *γ*-irradiation-induced apoptosis (Erlacher *et al*, 2005). However, we did find a positive correlation between GC resistance and the expression of *AIF* (apoptosis-inducing factor 1), *PARP* (poly ADP-ribose polymerase), *SMAC* (homolog of *DIABLO*) and cytochrome *c*, all of which are key genes in the initiation of apoptotic signalling. Although initially counter-intuitive that expression of these critical pro-apoptotic factors might be associated with increased GC resistance in ALL cell lines, all of these genes have important functions in normal cellular function. *PARP* has multiple biological functions including regulation of gene expression, cell division, differentiation and DNA repair, whereas *AIF* and cytochrome *c* have important functions in mitochondrial metabolism and protection from oxidative stress. Thus, their positive correlation with GC resistance is likely to be indicative of biological processes other than initiation of apoptosis. The only anti-apoptotic factor significantly associated with resistance to both GCs was *TRAF2* (TNF receptor-associated factor 2). In summary, there was no compelling signature at the level of gene expression of a coordinated deregulation of apoptotic pathways underpinning *in vitro* GC resistance.

### Drug profiles related to GC resistance (the Connectivity Map)

Recently Lamb *et al* (2006) described a database of drug–gene response signatures known as the CMAP that can be interrogated to identify drugs that may have synergistic mechanisms of action. We used CMAP to analyse the gene signatures associated with MPRED and DEX resistance in the present study. Table 3 details the CMAP drugs that scored significantly in the T-ALL GC-resistance signatures. Rapamycin scored significantly against both GC signatures, but was particularly impressive *vs* DEX (*P*<0.0001), confirming previous findings (Wei *et al*, 2006). Of the other drugs identified by CMAP in Table 3, there were three that scored significantly against both DEX and MPRED. These were LY294002 (a PI3K inhibitor), quercetin and resveratrol (both polyphenol antioxidants).

**Figure 4:**
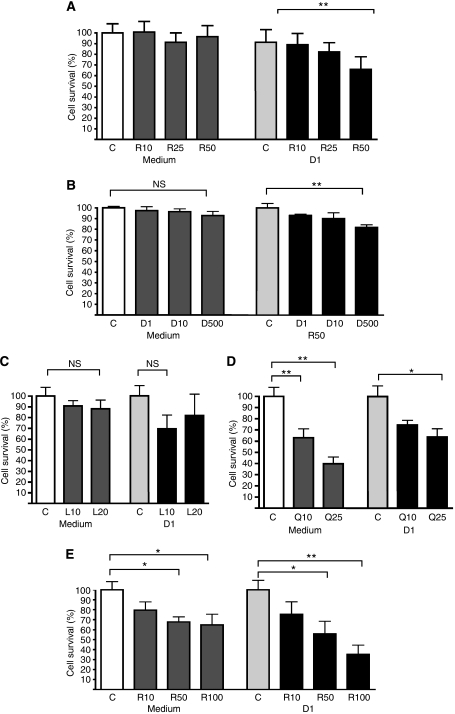
Synergy of CMAP-identified drugs with DEX in T-ALL cell lines. Graphs show cell survival following 48 h incubation. Comparisons were made in each data set to the respective control condition, which was set to 100%. Vehicle control (C) and the following drug treatments were analysed (**A**) 10 nM (R10), 25 nM (R25), 50 nM (R50) rapamycin and 1 *μ*M DEX (D1) in ALL-SIL; (**B**) 1 *μ*M (D1), 10 *μ*M (D10), 500 *μ*M (D500) DEX and 50 nM rapamycin (R50) in CEM; (**C**) 10 *μ*M (L10), 20 *μ*M (L20) LY294002 and 1 *μ*M DEX (D1) in ALL-SIL; (**D**) 10 *μ*M (Q10), 25 *μ*M (Q25) quercetin and 1 *μ*M DEX (D1) in ALL-SIL; (**E**) 10 *μ*M (R10), 50 *μ*M (R50), 100 *μ*M (R100) resveratrol and 1 *μ*M DEX (D1) in ALL-SIL; ^*^P<0.05, ^**^P<0.01 (repeated measures ANOVA). Data represent the mean of 4–5 experiments measured in triplicate (±s.e.m.).

To assess the potential synergy of these four agents with GC cytotoxicity, we tested their effect in ALL-SIL cells in the presence and absence of DEX for 48 h. Rapamycin at 50 nM had no cytotoxic effect on its own in ALL-SIL cells but was found to significantly enhance the cytotoxic effect of 1 *μ*M DEX in a dose-dependent manner (Figure 4A). Previous reports have demonstrated a synergistic effect of rapamycin on DEX cytotoxicity in T-ALL cell lines but in a cell-line-specific manner (Wei *et al*, 2006; Bornhauser *et al*, 2007). Sensitisation of the relatively DEX-resistant CEM-C1 cell line by rapamycin has been confirmed but the IC50 for these reported CEM variants is quoted as >10 *μ*M, substantially less than our own (spontaneously resistant) CEM line (>250 *μ*M) (Beesley *et al*, 2006). We therefore examined whether rapamycin could sensitise even this highly resistant CEM variant to the effects of DEX (Figure 4B). Although the effect of rapamycin was much less than with ALL-SIL, combination of 50 nM rapamycin with 500 *μ*M DEX did result in a statistically significant cytotoxic response, whereas DEX alone had no effect at this very high concentration. The findings confirm previous reports that rapamycin can sensitise T-ALL cells to the effect of DEX and demonstrate that this may be possible even in highly GC-resistant cell types. Rapamycin is an inhibitor of mTOR (the mammalian target of rapamycin), a key component of the PI3K/AKT/mTOR signalling pathway. The PI3K inhibitor LY294002 also targets this pathway and thus might be expected to have similar effects. Similar to rapamycin, LY294002 had no direct cytotoxic effects on ALL-SIL cells (Figure 4C) when added on its own but appeared to sensitise cells to the effects of DEX; however, the strength of this effect was more variable than for rapamycin and did not reach statistical significance.

Both quercetin and resveratrol demonstrated dose-dependent cytotoxicity in ALL-SIL cells in the absence of DEX (Figures 4D and E). Importantly, combination of either agent with 1 *μ*M DEX resulted in an additive cytotoxic effect. This is the first assessment of the cytotoxic effects of these compounds in combination with GCs in ALL and suggests that their inclusion in therapeutic regimens may have important clinical benefits.

## Discussion

The present study provides compelling evidence that cellular metabolism and proliferation are important aspects of GC resistance in ALL. It has long been recognised that an alteration in cellular metabolism is a fundamental phenotype of cancer (Warburg, 1956) including leukaemia (Boag *et al*, 2006), but debate continues as to the exact nature of the changes involved and their functional significance. The phenomenon was initially described by Otto Warburg as an enhanced rate of glycolysis to compensate for the energy deficiency caused by irreversible mitochondrial damage and loss of oxidative phosphorylation (Warburg, 1956). For many years this paradigm was indiscriminately applied to all types of tumours, but it is now known that mitochondrial function is often intact in cancer cells and that the metabolic phenotype is tumour specific, see Moreno-Sanchez *et al* (2007) for comprehensive review. Although the major form of energy production in many tumours is indeed glycolytic, others predominantly use oxidative phosphorylation or a combination of these pathways (Miccheli *et al*, 2006; Moreno-Sanchez *et al*, 2007). The compelling finding from the present study is that in GC-resistant T-ALL cells there is transcriptional upregulation of both glycolytic and oxidative phosphorylative pathways, and that these underpin macromolecule biosynthesis and a proliferative phenotype. It is well documented that activation of glycolysis has suppressive effects on apoptotic potential (Rathmell *et al*, 2003), whereas oxidative phosphorylation has been shown to exert both pro- (Tomiyama *et al*, 2006) and anti-apoptotic influences on the cell (Zong *et al*, 2004). Although the relative balance and thus final influence of these pathways on apoptotic potential cannot be directly determined from transcriptional data, it is possible that the proliferative phenotype described here confers resistance to GC cytotoxicity through the metabolic suppression of apoptotic potential. This would explain the resistance of T-ALL cell lines in the apparent absence of changes in the expression of key apoptotic regulators. Alternatively, a proliferative phenotype in ALL cells may indirectly confer resistance to GC cytotoxicity by offsetting the cytostatic pressures and metabolic suppression induced by GC signalling.

The conclusion that faster growing T-ALL cell lines are more GC resistant is supported by studies demonstrating that proliferating human peripheral T lymphocytes are more resistant to DEX than those that are growth arrested (Gilardini Montani *et al*, 1999; Lopes *et al*, 2007), and that the GC sensitivity of CCRF-CEM cells can be increased by overexpression of the cell-cycle inhibitor p16^INK4A^ (Ausserlechner *et al*, 2001). We also know that relapse specimens are both more proliferative and more GC resistant than diagnostic specimens (Klumper *et al*, 1995; Kaspers *et al*, 2005), and our own and other studies have reported increased expression of cell-cycle genes at relapse in ALL (Beesley *et al*, 2005; Bhojwani *et al*, 2006), particularly in patients that relapse early (Kirschner-Schwabe *et al*, 2006). Importantly, the observation contrasts with the cell-cycle dependency of most other ALL therapeutics (e.g., cytarabine, vincristine, thiopurines and the anthracyclines) where increased proliferation enhances cytotoxic effects. Overall the combined use of these drugs would be expected to exert the opposite effect to that of GCs, that is to provide a selective pressure for the emergence of leukemic clones that grow more slowly. Indeed a recent study concluded that detection of minimal residual disease after induction therapy in childhood ALL is associated with underexpression of genes promoting cell proliferation (Flotho *et al*, 2007). It is likely that the relationship between GC resistance and proliferation is not apparent from such studies because in the context of current multiagent therapy such effects would be masked. Only at relapse, when GC resistance emerges as a dominant feature, does a positive association with proliferation become more apparent.

It should be stressed that the data provided by this study represent correlations at the level of transcription and cannot account for post-transcriptional regulation and the kinetics of complex biochemical networks. In addition the experiments have been performed *in vitro* in conditions where availability of nutrients, particularly glucose and glutamine, are available in abundance. However, the data are compelling in their consistency and are complementary with the known metabolic actions of GCs (see Supplementary Figure S2). Previous reports have indicated an elevated glycolytic rate in prednisolone-resistant leukaemia cell lines (Tissing *et al*, 2007), and the involvement of carbohydrate metabolism genes in GC response (Tonko *et al*, 2001; Schmidt *et al*, 2006) and GC resistance in primary ALL specimens (Holleman *et al*, 2004). In the present study, GC resistance was significantly correlated with expression of hexokinase II, the rate-limiting glycolytic enzyme that has recently been associated both with induction failure in ALL (Winter *et al*, 2007) and discrimination between early and late ALL relapse (Bhojwani *et al*, 2006). Inhibition of cholesterol synthesis (required in highly proliferative cell types for the synthesis of cellular membranes) has been shown to be one of the earliest parameters affected by GCs in ALL cells (Cutts and Melnykovych, 1987), and is a critical metabolic event given that lymphocytes appear to be dependent on cholesterol synthesised endogenously (Madden *et al*, 1986). Finally, *GLUL* expression is induced by GCs (Harmon and Thompson, 1982) and we have recently reported that reduced expression of this gene is associated with adverse outcome in B-lineage ALL (Hoffmann *et al*, 2008). The importance of glutamine in ALL is highlighted by the fact that a significant proportion of the clinical efficacy of L-asparaginase, a cornerstone of therapy for this disease, is actually due to the glutaminase activity contained within such preparations (Reinert *et al*, 2006). The current data suggest that changes to amino-acid metabolism are not only fundamental to the leukemic phenotype but may also contribute to GC resistance.

The canonical pathway involved in nutrient sensing and metabolic control is one that involves PI3K (phosphatidylinositol 3-kinase), AKT and mTOR. Synergy between the AKT/mTOR pathway and GC sensitivity has previously been explored based on the finding that rapamycin responses show a significant relationship to DEX-resistance signatures in primary specimens from ALL patients (Wei *et al*, 2006; Bornhauser *et al*, 2007). In the present study, we have identified a similar association between rapamycin and GC resistance using *in vitro* data (CMAP analysis) and have confirmed the ability of this drug to sensitise even highly resistant ALL cell lines. Importantly, the expression of leading edge genes from the rapamycin-response signature in T-ALL diagnostic specimens could predict patient outcome; the same was true of the gene signatures associated with *ex vivo* DEX resistance, demonstrating the clinical relevance of this previously described signature (Wei *et al*, 2006). The Connectivity Map analysis also associated the polyphenol antioxidants resveratrol and quercetin with GC resistance, compounds that have been shown to inhibit proliferation and induce apoptosis in a variety of cancer cell types (Mertens-Talcott and Percival, 2005; Zunino and Storms, 2006; Roman-Gomez *et al*, 2007). The ability of these agents to enhance GC cytotoxicity, together with their favourable toxicity profile (they are abundant in everyday foods), reinforces the case for inclusion of such compounds in ALL therapeutic trials.

During the course of this investigation, we found an unexpected correlation between GC resistance and reduced expression of *MLL*, a master transcriptional regulator essential for normal mammalian development and haematopoiesis (Table 1, Figure 1). This correlation was confirmed to be highly significant *vs* both MPRED and DEX by quantitative RT-PCR (data not shown). On the basis of our data, we hypothesise that the observed correlation with GC sensitivity in T-ALL cell lines is related to expression levels of wild-type *MLL* rather than *MLL*-translocation products. The relationship between *MLL* expression, proliferation and GC resistance is currently being explored in our laboratory.

In summary, the present study provides compelling evidence that upregulation of cellular metabolism and proliferation is an important aspect of GC resistance in ALL and may contribute to patient outcome. We hypothesise that these changes simultaneously offset the adverse metabolic consequences of GC signalling whilst potentially suppressing apoptotic potential through the modulation of bioenergetic pathways. These observations support the continued development of selective metabolic inhibitors for the treatment of ALL, particularly for patients that are likely to be GC resistant, such as those with *MLL* rearrangements, induction failure or relapse. Since preparation of this paper, new observations to support our argument have been published by Hulleman *et al* (2009) who reported targeting the glycolytic pathway as a viable strategy for modulating steroid resistance in ALL.

## Figures and Tables

**Table 1:** Top 20 correlating probe sets associated with steroid resistance (IC50 scores) in T-ALL cell lines

**Methylprednisolone (MPRED)**				**Dexamethasone (DEX)**			
**Gene**	**Description**	**Probe**	***R* a**	**Gene**	**Description**	**Probe**	***R* a**
*GPHN*	Gephyrin	220773_s_at	0.915	*SOX14*	SRY-related HMG box 14	208574_at	0.884
*SOX14*	SRY-related HMG box 14	208574_at	0.91	*CLTC*	Clathrin, heavy polypeptide (Hc)	200614_at	0.881
*IGJ*	Immunoglobulin J polypeptide	212592_at	0.905	*NMT2*	*N*-myristoyltransferase 2	215743_at	−0.867
*MLL*	Mixed-lineage leukaemia	212079_s_at	−0.904	*DGKA*	Diacylglycerol kinase, alpha 80 kDa	203385_at	−0.863
*MLL*	Mixed-lineage leukaemia	212076_at	−0.895	*MLL*	Mixed-lineage leukaemia	212076_at	−0.859
*MLL*	Mixed-lineage leukaemia	216624_s_at	−0.893	*POLI*	Polymerase (DNA directed) I	219317_at	−0.858
*—*	Unknown	210491_at	−0.889	*SLC35B1*	Solute carrier family 35, member B1	202433_at	0.857
*MIF*	Macrophage migration inhibitory factor	217871_s_at	0.886	*PEX14*	Peroxisomal biogenesis factor 14	203503_s_at	0.856
*CLK1*	CDC-like kinase 1	214683_s_at	−0.882	*RPN2*	Ribophorin II	208689_s_at	0.852
*NCOA1*	Nuclear receptor coactivator 1	210249_s_at	−0.882	*MLL*	Mixed-lineage leukaemia	216624_s_at	−0.851
*—*	Unknown	216532_x_at	0.878	*—*	Hypothetical protein LOC283585	213929_at	−0.851
*NF1*	Neurofibromin 1	211914_x_at	−0.877	*MLL*	Mixed-lineage leukaemia	212080_at	−0.849
*EIF5*	Eukaryotic translation initiation factor 5	208708_x_at	0.876	*NDUFB1*	NADH dehydrogenase (ubiquinone) *β*1	206790_s_at	−0.849
*NMT2*	*N*-myristoyltransferase 2	215743_at	−0.873	*RPN2*	Ribophorin II	213399_x_at	0.843
*TREX2*	Three prime repair exonuclease 2	213334_x_at	0.872	*PSMC5*	Proteasome 26S subunit, ATPase, 5	209503_s_at	0.843
*TARBP2*	TAR (HIV) RNA-binding protein	203677_s_at	0.872	*—*	Unknown	221155_x_at	−0.841
*HSPC132*	Hypothetical protein HSPC132	218403_at	0.872	*FLJ13769*	Hypothetical protein FLJ13769	220719_at	−0.840
*OPHN1*	Oligophrenin 1	206323_x_at	−0.87	*ELK1*	Member of ETS oncogene family	217039_x_at	0.839
*FLJ13769*	Hypothetical protein FLJ13769	220719_at	−0.868	*MYCBP2*	MYC-binding protein 2	201959_s_at	−0.836
*ADSL*	Adenylosuccinate lyase	210250_x_at	0.866	*HIST1H4A*	Histone 1, H4a	208046_at	−0.835

a Pearson's correlation coefficient.

**Table 2:** Top 10 gene sets from GSEA database C2 (top) and ICHR Curated (bottom) datasets enriched in the MPRED-resistance signature

**GSEA rank**	**C2 Gene set name (C2 dataset)**	**Functional description**	***P*-value**	**FDR^a^**
1	VOXPHOS	Oxidative phosphorylation	0.002	0.115
2	BIOSYNTHESIS_OF_STEROIDS	Biosynthesis of steroids	0.013	0.108
3	PENG_RAPAMYCIN_DOWN	Downregulated in response to Rapamycin starvation in haematopoietic cells	0.003	0.093
4	PENG_GLUTAMINE_DOWN	Downregulated in response to glutamine starvation in haematopoietic cells	0.002	0.105
5	BRCA1_OVEREXP_DN	Downregulated in response to BRCA1 induction	0.001	0.098
6	CHOLESTEROL_BIOSYNTHESIS	Cholesterol biosynthesis pathway	0.038	0.090
7	ELECTRON_TRANSPORT_CHAIN	Electron transport chain	0.009	0.086
8	GLYCOLYSIS_AND_GLUCONEOGENESIS	Glycolysis and gluconeogenesis pathway	0.001	0.098
9	SCHUMACHER_MYC_P493_TET_UP	Upregulated by MYC expression in human B cells	0.015	0.099
10	IDX_TSA_UP_CLUSTER5	Upregulated during fibroblast differentiation induced by insulin, DEX, isobutylxanthine	0.002	0.100
				
**GSEA rank**	**Gene set name (ICHR- Curated dataset)**	**Functional description**	***P*-value**	**FDR^a^**
1	YOCUM_PROTEINS_IN_MLL_CELL_LINES_VS_CD34	Proteins expressed in MLL-translocated cell lines	0.001	0.030
2	BHOJWANI_EARLY_VS_LATE_PREB_LINREG	Genes differentially expressed between ALL early/late relapse	0.004	0.077
3	YEOH_DAV_MLL	Genes associated with MLL-patient subtypes	0.007	0.684
4	RHODES_NEOPLASTIC_META	Genes associated with the neoplastic phenotype	0.022	0.740
5	STAUNTON_696860	Response to compound NSC696860 in NCI cell lines	0.031	0.601
6	LAMB_DEX_UP	Upregulated in DEX-sensitive ALL cells	0.040	0.664
7	WEI_DOWN_IN_DEX_RESISTANCE	Downregulated in DEX-resistant ALL cells	0.040	0.569
8	LAMB_COMBINED_PHENOTHIAZINE	Upregulated in response to phenothiazine in cell lines	0.008	0.557
9	KIRSCHNER_SCWABE_ALL_EARLY_VS_LATE_RELAPSE	Genes differentially expressed between early/late ALL relapse	0.029	0.623
10	STAUNTON_668281	Response to compound NSC668281 in NCI cell lines	0.034	0.577

a FDR=false discovery rate calculated by GSEA.

**Table 3:** CMAP drugs significantly associated with MPRED- and DEX-resistance gene signatures in T-ALL cell lines

**MPRED**				**DEX**			
**Drug**	**Mechanism of action**	**Enrichment**	***P*-value**	**Drug**	**Mechanism of action**	**Enrichment**	***P*-value**
LY-294002	AKT/PI3K inhibitor	−0.507	0.0001	Rapamycin	mTOR inhibitor	−0.653	<0.0001
Trichostatin A	HDAC inhibitor	−0.423	0.018	Geldenamycin	HSP90 inhibitor	0.655	0.004
Carbamazepine	Antiepileptic	−0.792	0.020	LY-294002	AKT/PI3K inhibitor	−0.397	0.005
W-13	Calmodulin antagonist	0.903	0.021	Sodium phenylbutyrate	HDAC inhibitor	−0.564	0.014
Blebbistatin	Myosin II inhibitor	−0.896	0.023	Quercetin	ROS scavenger/antioxidant	0.903	0.019
Wortmannin	AKT/PI3K inhibitor	−0.497	0.024	Resveratrol	ROS scavenger/antioxidant	−0.630	0.020
Benserazide	Amino-acid decarboxylase inhibitor	0.885	0.028	Cobalt chloride	Hypoxia mimetic	−0.742	0.037
Rapamycin	mTOR inhibitor	−0.441	0.031	Fludrocortisone	Steroid	0.857	0.042
Indomethacin	Cyclo-oxygenase inhibitor	0.662	0.031	Deferoxamine	Hypoxia mimetic	−0.717	0.049
Quercetin	ROS scavenger/antioxidant	0.876	0.033				
Y-27632	Rho-kinase inhibitor	−0.872	0.036				
Tamoxifen	Oestrogen-receptor blocker	0.740	0.039				
Resveratrol	ROS scavenger/antioxidant	−0.583	0.040				

Data indicate the CMAP-calculated enrichment score and permuted *P*-value for association with GC expression profiles.
