# Glucocorticoid resistance in T-lineage acute lymphoblastic leukaemia is associated with a proliferative metabolism

**DOI:** 10.1038/sj.bjc.6605632

**Published:** 2010-03-30

**Authors:** A H Beesley, M J Firth, J Ford, R E Weller, J R Freitas, K U Perera, U R Kees

**Correction to**: *British Journal of Cancer* (2009) **100**, 1926–1936; doi:10.1038/sj.bjc.6605072

In reference to Figure 4D, the drug quercetin is clearly cytotoxic as a single agent in the ALL-SIL cell line, but its effect in combination with dexamethasone (DEX) is not synergistic as originally quoted. Rather, the combination of quercetin and DEX resulted in a small, though non-significant reduction in cytotoxicity. This is in contrast to resveratrol (Figure 4E), which demonstrated synergistic cytotoxicity in combination with DEX. This difference in the two agents is reflected in their connectivity map (CMAP) enrichment scores in Table 3, with quercetin showing a positive enrichment in contrast to the negative scores for resveratrol, LY-294002 and rapamycin. Both quercetin and resveratrol are polyphenols that activate the key metabolic regulator AMPK, but the results suggest that they are mechanistically different and are unlikely to be equally efficacious in combinatorial therapy with glucocorticoids in T-cell acute lymphoblastic leukaemia (T-ALL). Figure 4D and Table 3 from the original paper are reproduced for reference, below.

## Figures and Tables

**Figure 4:**
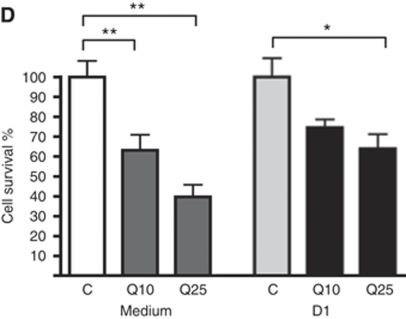
Synergy of CMAP-identified drugs with DEX in T-ALL cell lines. Graphs show cell survival following 48 h incubation. Comparisons were made in each data set to the respective control condition, which was set to 100%. Vehicle control (C) and the following drug treatments were analysed: (D) 10 mM (Q10), 25 mM (Q25) quercetin and 1 mM DEX (D1) in ALL-SIL.

**Table 3:** CMAP drugs significantly associated with MPRED- and DEX-resistance gene signatures in T-ALL cell lines

**MPRED**				**DEX**			
**Drug**	**Mechanism of action**	**Enrichment**	***P*-value**	**Drug**	**Mechanism of action**	**Enrichment**	***P*-value**
LY-294002	AKT/PI3K inhibitor	−0.507	0.0001	Rapamycin	mTOR inhibitor	−0.653	<0.0001
Trichostatin A	HDAC inhibitor	−0.423	0.018	Geldenamycin	HSP90 inhibitor	0.655	0.004
Carbamazepine	Antiepileptic	−0.792	0.020	LY-294002	AKT/PI3K inhibitor	−0.397	0.005
W-13	Calmodulin antagonist	0.903	0.021	Sodium phenylbutyrate	HDAC inhibitor	−0.564	0.014
Blebbistatin	Myosin II inhibitor	−0.896	0.023	Quercetin	ROS scavenger/antioxidant	0.903	0.019
Wortmannin	AKT/PI3K inhibitor	−0.497	0.024	Resveratrol	ROS scavenger/antioxidant	−0.630	0.020
Benserazide	Amino-acid decarboxylase inhibitor	0.885	0.028	Cobalt chloride	Hypoxia mimetic	−0.742	0.037
Rapamycin	mTOR inhibitor	−0.441	0.031	Fludrocortisone	Steroid	0.857	0.042
Indomethacin	Cyclo-oxygenase inhibitor	0.662	0.031	Deferoxamine	Hypoxia mimetic	−0.717	0.049
Quercetin	ROS scavenger/antioxidant	0.876	0.033				
Y-27632	Rho-kinase inhibitor	−0.872	0.036				
Tamoxifen	Oestrogen-receptor blocker	0.740	0.039				
Resveratrol	ROS scavenger/antioxidant	−0.583	0.040				

Data indicate the CMAP-calculated enrichment score and permuted *P*-value for association with GC expression profiles.

