# Supplementary material for: Glucocorticoid resistance in T-lineage acute lymphoblastic leukaemia is associated with a proliferative metabolism
Source: Br J Cancer. 2009 May 12;100(12):1926–36. doi: 10.1038/sj.bjc.6605072 (PMC2714233; doi:10.1038/sj.bjc.6605072)
Supplement: Supplementary Information [file 6605072x1.doc]

**SUPPLEMENTARY DOCUMENT**

*Glucocorticoid Resistance in T-Lineage Acute Lymphoblastic Leukemia is Associated with a Proliferative Metabolism (Beesley et al. 2008)*

Contents of Supplementary Document

- Supplementary Tables: Pages 2-22
- Supplementary Table S1: Publications and Databases Used to Compile the ICHR Curated Gene Set Dataset.
- Supplementary Table S2: Top 100 Probe Sets Correlating with MPRED IC50 in T-ALL Cells Lines.
- Supplementary Table S3: Top 100 Probe Sets Correlating with DEX IC50 in T-ALL Cells Lines.
- Supplementary Table S4: MPRED Leading Edge Genesets that Significantly Predict Relapse-free Survival in Primary Patient Specimens.
- Supplementary Table S5: Canonical Genes Involved in Multi-Drug Resistance / Apoptosis / Survival Pathways, and their Correlation with Steroid IC50 in T-ALL cell Lines.
  - Supplementary Figure: Pages 23-25
- Supplementary Figure S1: Comparison IC50 and doubling times in 15 T-ALL cell lines
- Supplementary Figure S2: Summary of the known cellular effects of glucocorticoids (GCs)
- Supplementary Methods: Page 26

Details of Separate Supplementary Data Files

- Supplementary Data File 1: Listing of Individual Gene Sets within the ICHR Curated Dataset.
- Supplementary Data File 2: Top Gene Sets Enriched in MPRED Signature.
- Supplementary Data File 3: Top Gene Sets Enriched in DEX signature.
- Supplementary Data File 4: Leading Edge Subsets for MPRED GeneSets showing individual genes and their correlations.

**SUPPLEMENTARY TABLES**

**Supplementary Table S1. Publications and Databases Used to Compile the ICHR Curated Gene Set Dataset.**

| **Source** | **Description** | **Gene Sets** |
| --- | --- | --- |
| Andersson et al. Leukemia 2005;19(6):1042-50. | Profiling of leukaemia cell lines with specific genetic aberrations | 10 |
| Andersson et al. PNAS 2005;102(52):19069-74. | Profiling of ALL versus normal haematopoietic sub-populations | 12 |
| Armstrong et al. Nat Genet 2002;30(1):41-7. | Signatures associated with MLL-translocations | 3 |
| Beesley et al. British J Haematology 2005;131(4):447-56. | Signatures associated with relapse in childhood ALL | 2 |
| Bhojwani et al. Blood 2006;108(2):711-7. | Biological pathways associated with relapse in childhood ALL | 5 |
| Boag et al. Leukemia 2006;20(10):1731-7. | Profile of non-leukaemic CD34+ cells versus ALL cells | 3 |
| Cario G et al. Blood 2005;105(2):821-6. | Profiles associated with molecular treatment response in childhood ALL | 4 |
| Chiaretti S et al. Clin Cancer Res 2005;11(20):7209-19. | Signatures of lineage derivation and transformation in adult B-lineage ALL | 4 |
| Dan et al. Cancer Res 2002;62(4):1139-47. | Chemosensitivity profiles for 55 anticancer drugs in human cell lines. | 2 |
| Dik WA et al. Leukemia 2005;19(11):1948-57. | CALM-AF10+ T-ALL expression profiles | 3 |
| Ferrando et al. Blood 2003;102(1):262-8. | Signatures in MLL-rearranged T-lineage and B-precursor acute leukaemias | 3 |
| Fine BM et al. Cancer Res 2005;65(1):291-9. | Profile of the *in vitro* response to l-asparaginase in ALL | 3 |
| Glinsky GV et al. J Clin Invest 2005;115(6):1503-21. | Signatures predicting therapy failure in patients with multiple types of cancer | 3 |
| Gyorffy B et al. Int J Cancer 2005. | Profiling of 30 cancer cell lines and resistance to 11 anticancer drugs | 11 |
| Hoffmann K et al. Genes Chromosomes Cancer 2004;41:309-20. | Profiling target genes of the proto-oncogene HOX11 | 1 |
| Holleman et al. N Engl J Med 2004;351(6):533-42. | Signatures of drug-resistant ALL and response to treatment | 8 |
| Kager et al. J Clin Invest 2005;115(1):110-7. | Folate pathway signatures in subtypes of ALL | 1 |
| Kirschner-Schwabe R et al. Clin Cancer Res 2006;12(15):4553-61. | Signatures of relapse in childhood ALL | 1 |
| Kohlmann et al. Leukemia 2004;18(1):63-71. | Signatures of paediatric and adult ALL subtypes | 11 |
| Lamb et al. Science 2006;313(5795):1929-35. | Small molecule signatures from the Connectivity Map | 13 |
| Lugthart S et al. Cancer Cell 2005;7(4):375-86. | Profiles of therapy resistance and treatment response in childhood ALL | 2 |
| Medh et al. Genomics 2003;81(6):543-55. | Profile of glucocorticoid-sensitive and resistant CEM cells | 3 |
| Muschen M et al. PNAS 2002;99(15):10014-9. | Molecular portraits of B cell lineage commitment | 1 |
| Obexer P et al. Oncogene 2001;20(32):4324-36. | Expression profiling of glucocorticoid-treated T-ALL cell lines | 3 |
| Ramaswamy S et al. Nat Genet 2003;33(1):49-54. | Molecular signature of metastasis in primary solid tumours | 2 |
| Ramaswamy S et al. PNAS 2001;98(26):15149-54. | Multiclass cancer diagnosis using tumour gene expression signatures | 16 |
| Rhodes DR et al. PNAS 2004;101(25):9309-14. | Transcriptional profiles of neoplastic transformation and progression | 2 |
| Rickardson L et al. Br J Cancer 2005;93(4):483-92. | Drug resistance gene expression profiles | 3 |
| Rozovskaia T et al. PNAS 2003;100(13):7853-8. | Profiles of acute leukaemias with ALL-1 rearrangements | 9 |
| Schmidt S et al. Blood 2006;107(5):2061-9. | Glucocorticoid-response genes in children with ALL | 10 |
| Schraets D et al. Oncogene 2003;22(23):3655-68. | Profile of MLL-mediated transcriptional gene regulation | 3 |
| Staib F et al. Cancer Res 2005;65(22):10255-64. | Profiles of the p53 tumor suppressor network | 5 |
| Staunton JE et al. PNAS 2001;98(19):10787-92. | Chemosensitivity prediction by transcriptional profiling | 232 |
| STRING database (http://string.embl.de) | Functional networks associated with individual genes | 63 |
| Toren A et al. Stem Cells 2005;23(8):1142-53. | CD133-positive haematopoietic stem cell profiles versus leukaemia | 3 |
| Tsutsumi S et al. Cancer Res 2003;63(16):4882-7. | Signatures in paediatric ALL patients with MLL rearrangements | 1 |
| Wei G et al. Cancer Cell 2006. | Transcriptional profile of glucocorticoid resistance | 3 |
| Willenbrock H et al. Leukemia 2004;18(7):1270-7. | Immunophenotype, treatment response and relapse in childhood ALL | 2 |
| Yeoh EJ et al. Cancer Cell 2002;1(2):133-43. | Subtype discovery and prediction of outcome in pediatric ALL | 35 |
| Yocum AK et al. J Proteome Res 2006;5(10):2743-2753. | Biomarkers and pharmacological targets in t(4;11) leukaemia | 1 |
| Zembutsu H et al. Cancer Res 2002;62(2):518-27. | Profiles of anticancer sensitivity in 85 human cancer xenografts | 10 |
|  | **Total** | 512 |

**Supplementary Table S2. Top 100 Probe Sets Correlating with MPRED IC50 in T-ALL Cells Lines.** Probe sets in bold represent those in common with the top 100 probe sets correlating with DEX resistance (Table S3).

| **Rank** | **Probe Set** | **Gene Symbol** | **Description** | **Correlation** |
| --- | --- | --- | --- | --- |
| 1 | 220773_s_at | *GPHN* | gephyrin | 0.915 |
| **2** | **208574_at** | ***SOX14*** | **SRY (sex determining region Y)-related HMG box 14** | **0.91** |
| **3** | **212592_at** | ***IGJ*** | **Immunoglobulin J polypeptide, linker protein for immunoglobulin alpha and mu polypeptides** | **0.905** |
| **4** | **212079_s_at** | ***MLL*** | **myeloid/lymphoid or mixed-lineage leukemia (trithorax homolog, Drosophila)** | **-0.904** |
| **5** | **212076_at** | ***MLL*** | **myeloid/lymphoid or mixed-lineage leukemia (trithorax homolog, Drosophila)** | **-0.895** |
| **6** | **216624_s_at** | ***MLL*** | **myeloid/lymphoid or mixed-lineage leukemia (trithorax homolog, Drosophila)** | **-0.893** |
| **7** | **210491_at** | ***-*** | **NA** | **-0.889** |
| 8 | 217871_s_at | *MIF* | macrophage migration inhibitory factor (glycosylation-inhibiting factor) | 0.886 |
| 9 | 214683_s_at | *CLK1* | CDC-like kinase 1 | -0.882 |
| 10 | 210249_s_at | *NCOA1* | nuclear receptor coactivator 1 | -0.882 |
| 11 | 216532_x_at | *-* | NA | 0.878 |
| **12** | **211914_x_at** | ***NF1*** | **neurofibromin 1 (neurofibromatosis, von Recklinghausen disease, Watson disease)** | **-0.877** |
| 13 | 208708_x_at | *EIF5* | eukaryotic translation initiation factor 5 | 0.876 |
| **14** | **215743_at** | ***NMT2*** | **N-myristoyltransferase 2** | **-0.873** |
| 15 | 213334_x_at | *TREX2* | three prime repair exonuclease 2 | 0.872 |
| 16 | 203677_s_at | *TARBP2* | TAR (HIV) RNA binding protein 2 | 0.872 |
| **17** | **218403_at** | ***HSPC132*** | **hypothetical protein HSPC132** | **0.872** |
| 18 | 206323_x_at | *OPHN1* | oligophrenin 1 | -0.87 |
| **19** | **220719_at** | ***FLJ13769*** | **hypothetical protein FLJ13769** | **-0.868** |
| 20 | 210250_x_at | *ADSL* | adenylosuccinate lyase | 0.866 |
| **21** | **220046_s_at** | ***CCNL1*** | **cyclin L1** | **-0.866** |
| **22** | **212080_at** | ***MLL*** | **Myeloid/lymphoid or mixed-lineage leukemia (trithorax homolog, Drosophila)** | **-0.865** |
| 23 | 217949_s_at | *VKORC1* | vitamin K epoxide reductase complex, subunit 1 | 0.865 |
| 24 | AFFX-CreX-3_at | *-* | NA | -0.863 |
| 25 | 215063_x_at | *FLJ20331* | Hypothetical protein FLJ20331 | -0.863 |
| 26 | 204516_at | *ATXN7* | ataxin 7 | -0.862 |
| 27 | AFFX-M27830_5_at | *SOX18* | SRY (sex determining region Y)-box 18 | -0.861 |
| **28** | **202433_at** | ***SLC35B1*** | **solute carrier family 35, member B1** | **0.86** |
| **29** | **209503_s_at** | ***PSMC5*** | **proteasome (prosome, macropain) 26S subunit, ATPase, 5** | **0.86** |
| 30 | 221932_s_at | *C14orf87* | chromosome 14 open reading frame 87 | 0.859 |
| 31 | 203791_at | *DMXL1* | Dmx-like 1 | -0.859 |
| **32** | **206936_x_at** | ***-*** | **NA** | **-0.859** |
| 33 | 209080_x_at | *TXNL2* | thioredoxin-like 2 | 0.859 |
| 34 | 201175_at | *TMX2* | thioredoxin-related transmembrane protein 2 | 0.857 |
| 35 | 207707_s_at | *SEC13L1* | SEC13-like 1 (S. cerevisiae) | 0.857 |
| 36 | 211882_x_at | *FUT6* | fucosyltransferase 6 (alpha (1,3) fucosyltransferase) | 0.856 |
| 37 | 211662_s_at | *VDAC2* | voltage-dependent anion channel 2 /// voltage-dependent anion channel 2 | 0.855 |
| 38 | 210573_s_at | *POLR3C* | polymerase (RNA) III (DNA directed) polypeptide C (62kD) | 0.855 |
| **39** | **221923_s_at** | ***NPM1*** | **nucleophosmin (nucleolar phosphoprotein B23, numatrin)** | **0.854** |
| 40 | 200818_at | *ATP5O* | ATP synthase, H+ transporting, mitochondrial F1 complex, O subunit (oligomycin sensitivity conferring protein) | 0.854 |
| 41 | 202151_s_at | *UBADC1* | ubiquitin associated domain containing 1 | 0.854 |
| 42 | 212980_at | *AHSA2* | AHA1, activator of heat shock 90kDa protein ATPase homolog 2 (yeast) | -0.853 |
| **43** | **212984_at** | ***-*** | **NA** | **-0.851** |
| 44 | 218836_at | *RPP21* | ribonuclease P 21kDa subunit | 0.849 |
| **45** | **213043_s_at** | ***THRAP4*** | **thyroid hormone receptor associated protein 4** | **0.849** |
| 46 | 209452_s_at | *VTI1B* | vesicle transport through interaction with t-SNAREs homolog 1B (yeast) | 0.848 |
| 47 | 208996_s_at | *POLR2C* | polymerase (RNA) II (DNA directed) polypeptide C, 33kDa | 0.847 |
| 48 | 216858_x_at | *-* | NA | -0.846 |
| 49 | 212087_s_at | *ERAL1* | Era G-protein-like 1 (E. coli) | 0.846 |
| 50 | 216769_x_at | *C9orf150* | Chromosome 9 open reading frame 150 | -0.845 |
| 51 | 215771_x_at | *RET* | ret proto-oncogene (multiple endocrine neoplasia and medullary thyroid carcinoma 1, Hirschsprung disease) | 0.845 |
| **52** | **200614_at** | ***CLTC*** | **clathrin, heavy polypeptide (Hc)** | **0.845** |
| 53 | 208290_s_at | *EIF5* | eukaryotic translation initiation factor 5 | 0.845 |
| 54 | 204323_x_at | *NF1* | neurofibromin 1 (neurofibromatosis, von Recklinghausen disease, Watson disease) | -0.844 |
| 55 | 221351_at | *HTR1A* | 5-hydroxytryptamine (serotonin) receptor 1A | 0.842 |
| 56 | 208787_at | *MRPL3* | mitochondrial ribosomal protein L3 | 0.842 |
| 57 | 215385_at | *FTO* | Fatso | -0.84 |
| 58 | 209107_x_at | *NCOA1* | nuclear receptor coactivator 1 | -0.839 |
| 59 | 215067_x_at | *PRDX2* | peroxiredoxin 2 | -0.839 |
| 60 | 202840_at | *TAF15* | TAF15 RNA polymerase II, TATA box binding protein (TBP)-associated factor, 68kDa | -0.838 |
| 61 | 202475_at | *NIFIE14* | seven transmembrane domain protein | 0.838 |
| 62 | 220232_at | *SCD4* | stearoyl-CoA desaturase 4 | -0.837 |
| **63** | **200643_at** | ***HDLBP*** | **high density lipoprotein binding protein (vigilin)** | **0.836** |
| **64** | **219317_at** | ***POLI*** | **polymerase (DNA directed) iota** | **-0.836** |
| 65 | AFFX-BioC-5_at | *-* | NA | -0.836 |
| 66 | AFFX-r2-P1-cre-3_at | *-* | NA | -0.836 |
| 67 | 212569_at | *KIAA0650* | KIAA0650 protein | -0.836 |
| 68 | 217884_at | *FLJ10774* | N-acetyltransferase-like protein | 0.835 |
| 69 | 204307_at | *KIAA0329* | KIAA0329 | -0.834 |
| 70 | 201180_s_at | *GNAI3* | guanine nucleotide binding protein (G protein), alpha inhibiting activity polypeptide 3 | 0.834 |
| 71 | 213989_x_at | *C21orf18* | chromosome 21 open reading frame 18 | 0.834 |
| 72 | 212577_at | *KIAA0650* | KIAA0650 protein | -0.834 |
| 73 | 218199_s_at | *NOL6* | nucleolar protein family 6 (RNA-associated) | 0.834 |
| 74 | 207365_x_at | *USP34* | ubiquitin specific protease 34 | -0.833 |
| 75 | 202400_s_at | *SRF* | serum response factor (c-fos serum response element-binding transcription factor) | 0.832 |
| 76 | 222315_at | *-* | NA | -0.83 |
| 77 | 213477_x_at | *EEF1A1* | eukaryotic translation elongation factor 1 alpha 1 | -0.828 |
| **78** | **203503_s_at** | ***PEX14*** | **peroxisomal biogenesis factor 14** | **0.828** |
| **79** | **201249_at** | ***SLC2A1*** | **solute carrier family 2 (facilitated glucose transporter), member 1** | **0.828** |
| 80 | AFFX-CreX-5_at | *-* | NA | -0.826 |
| 81 | 218305_at | *-* | NA | 0.826 |
| 82 | 204373_s_at | *CAP350* | centrosome-associated protein 350 | -0.826 |
| 83 | 200820_at | *PSMD8* | proteasome (prosome, macropain) 26S subunit, non-ATPase, 8 | 0.825 |
| 84 | 210386_s_at | *MTX1* | metaxin 1 | 0.825 |
| 85 | AFFX-r2-Ec-bioD-5_at | *-* | NA | -0.825 |
| 86 | 204233_s_at | *CHKA* | choline kinase alpha | 0.825 |
| 87 | 204774_at | *EVI2A* | ecotropic viral integration site 2A | -0.824 |
| 88 | 218898_at | *CT120* | membrane protein expressed in epithelial-like lung adenocarcinoma | 0.824 |
| 89 | AFFX-r2-P1-cre-5_at | *-* | NA | -0.823 |
| 90 | 212579_at | *KIAA0650* | KIAA0650 protein | -0.823 |
| 91 | 204366_s_at | *GTF3C2* | general transcription factor IIIC, polypeptide 2, beta 110kDa | 0.822 |
| 92 | 206136_at | *FZD5* | frizzled homolog 5 (Drosophila) | 0.822 |
| 93 | 202415_s_at | *HSPBP1* | hsp70-interacting protein | 0.822 |
| 94 | 208696_at | *CCT5* | chaperonin containing TCP1, subunit 5 (epsilon) | 0.821 |
| 95 | 219459_at | *POLR3B* | polymerase (RNA) III (DNA directed) polypeptide B | 0.821 |
| 96 | 209100_at | *IFRD2* | interferon-related developmental regulator 2 | 0.821 |
| 97 | 202937_x_at | *CGI-96* | CGI-96 protein | 0.82 |
| 98 | 219219_at | *FLJ20512* | hypothetical protein FLJ20512 | 0.819 |
| 99 | 207396_s_at | *ALG3* | asparagine-linked glycosylation 3 homolog (yeast, alpha-1,3-mannosyltransferase) | 0.819 |
| 100 | 212680_x_at | *PPP1R14B* | Protein phosphatase 1, regulatory (inhibitor) subunit 14B | 0.819 |

**Supplementary Table S3. Top 100 Probe Sets Correlating with DEX IC50 in T-ALL Cells Lines.** Probe sets in bold represent those in common with the top 100 probe sets correlating with MPRED resistance (Table S2).

| **Rank** | **Probe Set** | **Gene Symbol** | **Description** | **Correlation** |
| --- | --- | --- | --- | --- |
| **1** | **208574_at** | ***SOX14*** | **SRY (sex determining region Y)-related HMG box 14** | **0.884** |
| **2** | **200614_at** | ***CLTC*** | **clathrin, heavy polypeptide (Hc)** | **0.881** |
| **3** | **215743_at** | ***NMT2*** | **N-myristoyltransferase 2** | **-0.867** |
| 4 | 203385_at | *DGKA* | diacylglycerol kinase, alpha 80kDa | -0.863 |
| **5** | **212076_at** | ***MLL*** | **myeloid/lymphoid or mixed-lineage leukemia (trithorax homolog, Drosophila)** | **-0.859** |
| **6** | **219317_at** | ***POLI*** | **polymerase (DNA directed) iota** | **-0.858** |
| **7** | **202433_at** | ***SLC35B1*** | **solute carrier family 35, member B1** | **0.857** |
| **8** | **203503_s_at** | ***PEX14*** | **peroxisomal biogenesis factor 14** | **0.856** |
| 9 | 208689_s_at | *RPN2* | ribophorin II | 0.852 |
| **10** | **216624_s_at** | ***MLL*** | **myeloid/lymphoid or mixed-lineage leukemia (trithorax homolog, Drosophila)** | **-0.851** |
| 11 | 213929_at | *-* | Transcribed locus, weakly similar to XP_375099.1 hypothetical protein LOC283585 | -0.851 |
| **12** | **212080_at** | ***MLL*** | **Myeloid/lymphoid or mixed-lineage leukemia (trithorax homolog, Drosophila)** | **-0.849** |
| 13 | 206790_s_at | *NDUFB1* | NADH dehydrogenase (ubiquinone) 1 beta subcomplex, 1, 7kDa | -0.849 |
| 14 | 213399_x_at | *RPN2* | ribophorin II | 0.843 |
| **15** | **209503_s_at** | ***PSMC5*** | **proteasome (prosome, macropain) 26S subunit, ATPase, 5** | **0.843** |
| 16 | 221155_x_at | *-* | NA | -0.841 |
| **17** | **220719_at** | ***FLJ13769*** | **hypothetical protein FLJ13769** | **-0.840** |
| 18 | 217039_x_at | *ELK1* | ELK1, member of ETS oncogene family | 0.839 |
| 19 | 201959_s_at | *MYCBP2* | MYC binding protein 2 | -0.836 |
| 20 | 208046_at | *HIST1H4A* | histone 1, H4a | -0.835 |
| 21 | 205802_at | *TRPC1* | transient receptor potential cation channel, subfamily C, member 1 | -0.834 |
| 22 | 219700_at | *PLXDC1* | plexin domain containing 1 | -0.832 |
| 23 | 205934_at | *PLCL1* | phospholipase C-like 1 | -0.830 |
| 24 | 208819_at | *RAB8A* | RAB8A, member RAS oncogene family | 0.828 |
| 25 | 214852_x_at | *VPS13A* | vacuolar protein sorting 13A (yeast) | -0.826 |
| 26 | 54051_at | *PKNOX1* | PBX/knotted 1 homeobox 1 | -0.824 |
| 27 | 221800_s_at | *FLJ22175* | hypothetical protein FLJ22175 | 0.823 |
| 28 | 208014_x_at | *AD7C-NTP* | neuronal thread protein AD7c-NTP | -0.820 |
| 29 | 213491_x_at | *RPN2* | ribophorin II | 0.817 |
| 30 | 215221_at | *-* | NA | -0.816 |
| 31 | 211565_at | *SH3GL3* | SH3-domain GRB2-like 3 | -0.815 |
| **32** | **213043_s_at** | ***THRAP4*** | **thyroid hormone receptor associated protein 4** | **0.813** |
| **33** | **220046_s_at** | ***CCNL1*** | **cyclin L1** | **-0.812** |
| 34 | 212721_at | *SFRS12* | splicing factor, arginine/serine-rich 12 | -0.812 |
| 35 | 208803_s_at | *SRP72* | signal recognition particle 72kDa | 0.811 |
| 36 | 216557_x_at | *IGHG1* | immunoglobulin heavy constant gamma 1 (G1m marker) | -0.809 |
| 37 | 221103_s_at | *FLJ11142* | hypothetical protein FLJ11142 | -0.808 |
| 38 | 205803_s_at | *TRPC1* | transient receptor potential cation channel, subfamily C, member 1 | -0.807 |
| 39 | 205331_s_at | *C5orf19* | chromosome 5 open reading frame 19 | 0.806 |
| 40 | 202850_at | *ABCD3* | ATP-binding cassette, sub-family D (ALD), member 3 | -0.806 |
| 41 | 217906_at | *KLHDC2* | kelch domain containing 2 | -0.805 |
| 42 | 201695_s_at | *NP* | nucleoside phosphorylase | 0.805 |
| 43 | 205559_s_at | *PCSK5* | proprotein convertase subtilisin/kexin type 5 | -0.804 |
| 44 | 204258_at | *CHD1* | chromodomain helicase DNA binding protein 1 | -0.803 |
| **45** | **211914_x_at** | ***NF1*** | **neurofibromin 1 (neurofibromatosis, von Recklinghausen disease, Watson disease)** | **-0.802** |
| 46 | 209856_x_at | *-* | NA | -0.798 |
| 47 | 203533_s_at | *CUL5* | cullin 5 | -0.798 |
| 48 | 222167_at | *PVRL3* | Poliovirus receptor-related 3 | -0.797 |
| **49** | **206936_x_at** | ***-*** | **NA** | **-0.794** |
| **50** | **212984_at** | ***-*** | **NA** | **-0.794** |
| 51 | 205345_at | *BARD1* | BRCA1 associated RING domain 1 | -0.793 |
| 52 | 202355_s_at | *GTF2F1* | general transcription factor IIF, polypeptide 1, 74kDa | 0.793 |
| 53 | 215149_at | *-* | CDNA clone IMAGE:5260262, partial cds | -0.792 |
| 54 | 206460_at | *SHREW1* | transmembrane protein SHREW1 | -0.792 |
| 55 | 204387_x_at | *MRP63* | mitochondrial ribosomal protein 63 | -0.791 |
| 56 | 212723_at | *PTDSR* | phosphatidylserine receptor | 0.791 |
| **57** | **201249_at** | ***SLC2A1*** | **solute carrier family 2 (facilitated glucose transporter), member 1** | **0.788** |
| 58 | 218103_at | *FTSJ3* | FtsJ homolog 3 (E. coli) | 0.788 |
| 59 | 215656_at | *LMAN2* | Lectin, mannose-binding 2 | -0.786 |
| 60 | 209495_at | *CEP2* | centrosomal protein 2 | 0.786 |
| 61 | 217540_at | *-* | NA | -0.786 |
| **62** | **218403_at** | ***HSPC132*** | **hypothetical protein HSPC132** | **0.785** |
| **63** | **200643_at** | ***HDLBP*** | **high density lipoprotein binding protein (vigilin)** | **0.785** |
| 64 | 203201_at | *PMM2* | phosphomannomutase 2 | 0.784 |
| 65 | 205409_at | *FOSL2* | FOS-like antigen 2 | 0.783 |
| 66 | 213077_at | *YTHDC2* | YTH domain containing 2 | -0.781 |
| 67 | 215789_s_at | *SHREW1* | transmembrane protein SHREW1 | -0.781 |
| 68 | 212943_at | *KIAA0528* | KIAA0528 gene product | -0.781 |
| 69 | 221883_at | *PKNOX1* | PBX/knotted 1 homeobox 1 | -0.779 |
| 70 | 215566_x_at | *LYPLA2* | lysophospholipase II | 0.779 |
| 71 | 204096_s_at | *ELL* | elongation factor RNA polymerase II | 0.779 |
| 72 | 212921_at | *SMYD2* | SET and MYND domain containing 2 | -0.778 |
| 73 | 202622_s_at | *ATXN2* | ataxin 2 | -0.778 |
| 74 | 220041_at | *SMP3* | SMP3 mannosyltransferase | -0.778 |
| 75 | 215507_x_at | *RAB22A* | RAB22A, member RAS oncogene family | -0.778 |
| 76 | 215069_at | *NMT2* | N-myristoyltransferase 2 | -0.777 |
| **77** | **212079_s_at** | ***MLL*** | **myeloid/lymphoid or mixed-lineage leukemia (trithorax homolog, Drosophila)** | **-0.777** |
| 78 | 207663_x_at | *GAGE3* | G antigen 3 | 0.777 |
| 79 | 202121_s_at | *BC-2* | putative breast adenocarcinoma marker (32kD) | 0.776 |
| 80 | 204094_s_at | *KIAA0669* | KIAA0669 gene product | -0.776 |
| **81** | **212592_at** | ***IGJ*** | **Immunoglobulin J polypeptide, linker protein for immunoglobulin alpha and mu polypeptides** | **0.775** |
| 82 | 212513_s_at | *USP33* | ubiquitin specific protease 33 | -0.773 |
| 83 | 213649_at | *SFRS7* | splicing factor, arginine/serine-rich 7, 35kDa | -0.773 |
| **84** | **210491_at** | ***-*** | **NA** | **-0.772** |
| 85 | 219648_at | *FLJ10116* | whn-dependent transcript 2 | 0.772 |
| 86 | 201960_s_at | *MYCBP2* | MYC binding protein 2 | -0.772 |
| 87 | 213703_at | *LOC150759* | hypothetical protein LOC150759 | -0.772 |
| 88 | 204833_at | *APG12L* | APG12 autophagy 12-like (S. cerevisiae) | 0.770 |
| 89 | 204357_s_at | *LIMK1* | LIM domain kinase 1 | 0.770 |
| 90 | 211272_s_at | *DGKA* | diacylglycerol kinase, alpha 80kDa | -0.770 |
| 91 | 207218_at | *F9* | coagulation factor IX (plasma thromboplastic component, Christmas disease, hemophilia B) | -0.769 |
| 92 | 203262_s_at | *FAM50A* | family with sequence similarity 50, member A | 0.769 |
| 93 | 205407_at | *RECK* | reversion-inducing-cysteine-rich protein with kazal motifs | -0.768 |
| 94 | 211932_at | *HNRPA3* | heterogeneous nuclear ribonucleoprotein A3 | -0.768 |
| 95 | 212583_at | *-* | NA | -0.768 |
| **96** | **221923_s_at** | ***NPM1*** | **nucleophosmin (nucleolar phosphoprotein B23, numatrin)** | **0.766** |
| 97 | 216702_x_at | *-* | NA | -0.766 |
| 98 | 201805_at | *PRKAG1* | protein kinase, AMP-activated, gamma 1 non-catalytic subunit | 0.766 |
| 99 | 201790_s_at | *DHCR7* | 7-dehydrocholesterol reductase | 0.766 |
| 100 | 212402_at | *KIAA0853* | KIAA0853 | -0.766 |

**Supplementary Table S4. MPRED leading edge genesets that significantly predict relapse-free survival in primary patient specimens.** Leading edge subsets from the top 20 MPRED genesets from each database were used to predict outcome using microarray data from T-lineage and B-lineage primary patient specimens (Beesley et al BJH 2005). Significant leading edge genesets are bolded (p<0.05, by permutation modelling); those significant in both lineages are highlighted.

| **Geneset Name** | **Data Set** | **Geneset Description** | **GeneSet Size** | **Genes in Leading Edge** | **T-Lineage p-value** | **B-lineage p-value** |
| --- | --- | --- | --- | --- | --- | --- |
| AGUIRRE_PANCREAS_CHR17 | C2 | Chromosome 17 copy number driven expression in pancreatic cancer | 65 | 28 | 0.429 | **0.016** |
| BIOSYNTHESIS_OF_STEROIDS | C2 | Steroid biosynthesis pathway | 15 | 12 | 0.263 | **0.002** |
| CARBON_FIXATION | C2 | Carbon fixation pathway | 21 | 11 | **0.025** | 0.715 |
| CGGAARNGGCNG_UNKNOWN | C3 | Uncharacterised transcription factor motif | 39 | 22 | 0.053 | **0.014** |
| CHOLESTEROL_BIOSYNTHESIS | C2 | Cholesterol biosynthetic pathway | 16 | 13 | **0.033** | **0.002** |
| GGAANCGGAANY_UNKNOWN | C3 | Uncharacterised transcription factor motif | 77 | 23 | **0.023** | 0.674 |
| GKCGCNNNNNNNTGAYG_UNKNOWN | C3 | Uncharacterised transcription factor motif | 41 | 26 | 0.447 | **0.014** |
| GNF2_PA2G4 | C4 | Genes involved with PA2G4 proliferation-associated protein. | 73 | 43 | **0.020** | 0.217 |
| LAMB_COMBINED_PHENOTHIAZINE | ICHR | Genes up-regulated in response to phenothiazine in cell lines | 61 | 34 | 0.210 | **0.032** |
| LAMB_DEX_UP | ICHR | Genes up-regulated in DEX sensitive ALL cells | 47 | 15 | **0.042** | **0.024** |
| MORF_DEAF1 | C4 | Genes associated with DEAF1 (supressin - inhibitor of proliferation) | 55 | 35 | 0.442 | **0.027** |
| MORF_GMPS | C4 | Genes associated with guanine monphosphate synthetase (an MLL translocation partner). | 49 | 27 | 0.253 | **0.021** |
| MORF_MAP2K2 | C4 | Genes involved in MAP2K2 signalling | 125 | 67 | 0.670 | **0.004** |
| MORF_PRDX3 | C4 | Genes involved with Peroxiredoxin 3 - required for MYC-mediated proliferation and apoptosis after glucose starvation | 81 | 46 | 0.229 | **0.018** |
| MORF_SOD1 | C4 | Genes involved with Superoxide dismutase 1 | 258 | 141 | 0.235 | **0.009** |
| OXIDATIVE_PHOSPHORYLATION | C2 | Oxidative phosphorylation | 58 | 34 | **0.044** | 0.684 |
| PENG_RAPAMYCIN_DOWN | C2 | Down-regulated in response to Rapamycin starvation in haematopoietic cells | 200 | 120 | **0.031** | 0.681 |
| STAUNTON_668281 | ICHR | Response to compound NSC668281 in NCI cell lines | 28 | 13 | **0.039** | 0.682 |
| STRING_ELL_TOP100_ASSOCIATIONS | ICHR | Genes associated with Elongation Factor RNA Polymerase II (an MLL translocation partner) | 59 | 25 | 0.197 | **0.012** |
| STRING_GLUL_TOP100_ASSOCIATIONS | ICHR | Genes associated with GLUL (Glutamine synthetase) | 33 | 23 | **0.029** | **0.004** |
| V$ARNT_02 | C3 | Genes regulated by Aryl hydrocarbon receptor nuclear translocator (HIF1B). | 197 | 96 | **0.027** | 0.666 |
| V$ER_Q6 | C3 | Genes regulated by ESR1: estrogen receptor 1 | 190 | 101 | 0.119 | **0.021** |
| V$MAX_01 | C3 | Genes regulated by MAX (includes MLL) | 200 | 109 | 0.059 | **0.024** |
| V$MYC_Q2 | C3 | MYC-regulated genes (includes MLL) | 132 | 56 | **0.041** | **0.012** |
| V$MYCMAX_01 | C3 | Genes regulated by MYC/MAX (e.g. MLL) | 197 | 110 | **0.044** | **0.030** |
| V$MYCMAX_02 | C3 | Genes regulated by MYC/MAX (e.g. MLL) | 204 | 106 | 0.436 | **0.038** |
| V$NFMUE1_Q6 | C3 | Uncharacterised transcription factor motif | 183 | 82 | **0.028** | 0.670 |
| V$PAX3_01 | C3 | Genes regulated by PAX3: paired box gene 3 | 15 | 9 | **0.043** | **0.025** |
| V$STAT1_02 | C3 | Genes regulated by STAT1: Signal Transducer and Activator of Transcription | 181 | 83 | 0.109 | **0.004** |
| V$USF_C | C3 | Uncharacterised transcription factor motif | 208 | 102 | **0.025** | **0.020** |
| V$USF2_Q6 | C3 | Genes containing this uncharacterised transcription factor binding motif (e.g. MLL) | 183 | 76 | 0.174 | **0.014** |
| V$YY1_Q6 | C3 | Genes regulated by YY1 transcription factor – (involved in DNA repair/p53 pathways) | 176 | 89 | **0.047** | 0.128 |
| VOXPHOS | C2 | Oxidative phosphorylation | 81 | 50 | **0.038** | 0.692 |
| WCTCNATGGY_UNKNOWN | C3 | Uncharacterised transcription factor motif | 54 | 27 | 0.056 | **0.024** |
| WEI_DOWN_IN_DEX_RESISTANCE | ICHR | Genes down-regulated in DEX resistant ALL cells | 47 | 15 | **0.038** | **0.016** |

**Supplementary Table S5. Canonical Genes Involved in Multi-Drug Resistance / Apoptosis / Survival Pathways, and their Correlation with Steroid IC50 in T-ALL cell Lines.** Probe sets with correlations greater than expected by chance after adjustment for the number of genes tested are given in bold; those significant versus one drug only are highlighted yellow (significance cut-off p<0.004); those significant versus both drugs are highlighted red (significance cut-off p<0.05). The gene list was compiled from the literature.

|  | |  | **MPRED** | | **DEX** | |
| --- | --- | --- | --- | --- | --- | --- |
| **Gene Symbol** | **Gene** | **Probe Set ID** | **Correlation (r)** | ***P*-value** | **Correlation (r)** | ***P*-value** |
| *ABCB1* | ATP-binding cassette, sub-family B (MDR/TAP), member 1 | 209993_at | -0.400 | 0.140 | -0.326 | 0.236 |
| *ABCB1 /// ABCB4* | ATP-binding cassette, sub-family B (MDR/TAP) members 1 and 4 | 209994_s_at | 0.470 | 0.077 | 0.365 | 0.181 |
| *ABCB11* | ATP-binding cassette, sub-family B (MDR/TAP), member 11 | 208288_at | 0.518 | 0.048 | 0.395 | 0.145 |
| *ABCB11* | ATP-binding cassette, sub-family B (MDR/TAP), member 11 | 211224_s_at | -0.419 | 0.120 | -0.347 | 0.205 |
| *ABCB4* | ATP-binding cassette, sub-family B (MDR/TAP), member 4 | 207819_s_at | 0.515 | 0.050 | 0.267 | 0.335 |
| *ABCB6* | ATP-binding cassette, sub-family B (MDR/TAP), member 6 | 203192_at | 0.302 | 0.275 | 0.366 | 0.180 |
| *ABCB8* | ATP-binding cassette, sub-family B (MDR/TAP), member 8 | 206317_s_at | 0.519 | 0.047 | 0.237 | 0.394 |
| *ABCB9* | ATP-binding cassette, sub-family B (MDR/TAP), member 9 | 214209_s_at | 0.488 | 0.065 | 0.440 | 0.101 |
| *ABCB9* | ATP-binding cassette, sub-family B (MDR/TAP), member 9 | 207321_s_at | 0.300 | 0.278 | 0.178 | 0.526 |
| *ABCC1* | ATP-binding cassette, sub-family C (CFTR/MRP), member 1 | 202804_at | -0.080 | 0.776 | 0.233 | 0.403 |
| *ABCC1* | ATP-binding cassette, sub-family C (CFTR/MRP), member 1 | 202805_s_at | -0.349 | 0.203 | -0.037 | 0.896 |
| *ABCC10* | ATP-binding cassette, sub-family C (CFTR/MRP), member 10 | 215873_x_at | -0.011 | 0.969 | 0.276 | 0.319 |
| *ABCC10* | ATP-binding cassette, sub-family C (CFTR/MRP), member 10 | 213485_s_at | -0.153 | 0.586 | 0.197 | 0.483 |
| *ABCC2* | ATP-binding cassette, sub-family C (CFTR/MRP), member 2 | 206155_at | 0.228 | 0.414 | 0.221 | 0.429 |
| *ABCC3* | ATP-binding cassette, sub-family C (CFTR/MRP), member 3 | 208161_s_at | **0.751** | **0.001** | 0.464 | 0.082 |
| *ABCC3* | ATP-binding cassette, sub-family C (CFTR/MRP), member 3 | 214979_at | -0.228 | 0.414 | -0.396 | 0.144 |
| *ABCC3* | ATP-binding cassette, sub-family C (CFTR/MRP), member 3 | 209641_s_at | 0.009 | 0.975 | -0.132 | 0.640 |
| *ABCC4* | ATP-binding cassette, sub-family C (CFTR/MRP), member 4 | 203196_at | 0.093 | 0.743 | 0.277 | 0.317 |
| *ABCC5* | ATP-binding cassette, sub-family C (CFTR/MRP), member 5 | 209380_s_at | -0.362 | 0.185 | -0.411 | 0.128 |
| *ABCC6* | ATP-binding cassette, sub-family C (CFTR/MRP), member 6 | 208480_s_at | 0.410 | 0.129 | 0.382 | 0.160 |
| *ABCC6* | ATP-binding cassette, sub-family C (CFTR/MRP), member 6 | 215559_at | 0.089 | 0.753 | -0.040 | 0.888 |
| *ABCC8* | ATP-binding cassette, sub-family C (CFTR/MRP), member 8 | 210246_s_at | 0.402 | 0.137 | 0.167 | 0.553 |
| *ABCC9* | ATP-binding cassette, sub-family C (CFTR/MRP), member 9 | 208561_at | **0.711** | **0.003** | **0.640** | **0.010** |
| *ABCC9* | ATP-binding cassette, sub-family C (CFTR/MRP), member 9 | 208562_s_at | 0.137 | 0.626 | -0.132 | 0.640 |
| *ABCC9* | ATP-binding cassette, sub-family C (CFTR/MRP), member 9 | 208462_s_at | 0.028 | 0.921 | -0.034 | 0.905 |
| *AIF* | apoptosis-inducing factor, mitochondrion-associated, 1 | 205512_s_at | **0.695** | **0.004** | **0.571** | **0.026** |
| *AOX1* | aldehyde oxidase 1 | 205082_s_at | 0.132 | 0.638 | -0.148 | 0.599 |
| *AOX1* | aldehyde oxidase 1 | 205083_at | 0.255 | 0.359 | 0.081 | 0.773 |
| *APAF1* | apoptotic peptidase activating factor 1 | 204859_s_at | -0.500 | 0.058 | -0.267 | 0.336 |
| *APAF1* | apoptotic peptidase activating factor 1 | 211553_x_at | -0.452 | 0.091 | -0.238 | 0.392 |
| *ARF* | ARF | 208750_s_at | 0.344 | 0.209 | 0.543 | 0.036 |
| *ARF* | ARF | 200065_s_at | 0.337 | 0.220 | 0.497 | 0.059 |
| *ASNS* | asparagine synthetase | 205047_s_at | 0.066 | 0.815 | 0.171 | 0.543 |
| *ATM* | Ataxia telangiectasia mutated | 212672_at | -0.460 | 0.085 | -0.487 | 0.065 |
| *ATM* | ataxia telangiectasia mutated | 210858_x_at | -0.223 | 0.423 | -0.369 | 0.176 |
| *ATM* | ataxia telangiectasia mutated | 208442_s_at | -0.304 | 0.271 | -0.361 | 0.186 |
| *ATR* | ataxia telangiectasia and Rad3 related | 209903_s_at | -0.454 | 0.089 | -0.589 | 0.021 |
| *ATR* | ataxia telangiectasia and Rad3 related | 209902_at | 0.027 | 0.924 | -0.310 | 0.260 |
| *BAD* | BCL2-antagonist of cell death | 209364_at | 0.258 | 0.354 | 0.080 | 0.778 |
| *BAD* | BCL2-antagonist of cell death | 1861_at | -0.023 | 0.935 | -0.068 | 0.809 |
| *BAG1* | BCL2-associated athanogene | 211475_s_at | 0.587 | 0.022 | 0.275 | 0.321 |
| *BAG1* | BCL2-associated athanogene | 202387_at | 0.561 | 0.030 | 0.222 | 0.427 |
| *BAG3* | BCL2-associated athanogene 3 | 217911_s_at | 0.284 | 0.304 | 0.148 | 0.598 |
| *BAG4* | BCL2-associated athanogene 4 | 219624_at | 0.475 | 0.073 | 0.470 | 0.077 |
| *BAG5* | BCL2-associated athanogene 5 | 202985_s_at | 0.601 | 0.018 | 0.350 | 0.201 |
| *BAG5* | BCL2-associated athanogene 5 | 202984_s_at | -0.102 | 0.716 | -0.348 | 0.203 |
| *BAK1* | BCL2-antagonist/killer 1 | 203728_at | 0.353 | 0.197 | 0.385 | 0.156 |
| *BAX* | BCL2-associated X protein | 217029_at | -0.298 | 0.280 | -0.304 | 0.271 |
| *BAX* | BCL2-associated X protein | 208478_s_at | -0.347 | 0.205 | -0.056 | 0.842 |
| *BAX* | BCL2-associated X protein | 211833_s_at | -0.272 | 0.327 | -0.007 | 0.981 |
| *BBC3 // PUMA* | BCL2 binding component 3 | 211692_s_at | -0.220 | 0.432 | -0.231 | 0.407 |
| *BCL2* | B-cell CLL/lymphoma 2 | 207005_s_at | 0.396 | 0.144 | 0.559 | 0.030 |
| *BCL2* | B-cell CLL/lymphoma 2 | 203685_at | 0.104 | 0.711 | 0.373 | 0.171 |
| *BCL2* | B-cell CLL/lymphoma 2 | 207004_at | -0.071 | 0.802 | -0.016 | 0.956 |
| *BCL2* | B-cell CLL/lymphoma 2 | 203684_s_at | 0.300 | 0.279 | 0.465 | 0.081 |
| *BCL2A1* | BCL2-related protein A1 | 205681_at | -0.033 | 0.915 | 0.301 | 0.277 |
| *BCL2L10* | BCL2-like 10 (apoptosis facilitator) | 221320_at | -0.040 | 0.887 | -0.087 | 0.761 |
| *BCL2L11* | BCL2-like 11 (apoptosis facilitator) / Bim | 208536_s_at | 0.687 | 0.005 | 0.411 | 0.128 |
| *BCL2L11* | BCL2-like 11 (apoptosis facilitator) / Bim | 222343_at | 0.655 | 0.008 | 0.355 | 0.195 |
| *BCL2L14* | BCL2-like 14 (apoptosis facilitator) | 221241_s_at | -0.034 | 0.907 | -0.308 | 0.264 |
| *BCL2L2* | BCL2-like 2 | 209311_at | -0.107 | 0.706 | 0.200 | 0.475 |
| *BCL2L1* | BCL2-like 1 | 212312_at | 0.134 | 0.635 | 0.144 | 0.607 |
| *BCL2L1* | BCL2-like 1 | 215037_s_at | 0.100 | 0.722 | 0.079 | 0.779 |
| *BCL2L1* | BCL2-like 1 | 206665_s_at | -0.157 | 0.578 | -0.063 | 0.823 |
| *BCL2L13* | BCL2-like 13 (apoptosis facilitator) | 217955_at | 0.108 | 0.700 | 0.206 | 0.462 |
| *BFAR* | bifunctional apoptosis regulator | 218056_at | 0.440 | 0.101 | 0.419 | 0.120 |
| *BID* | BH3 interacting domain death agonist | 211725_s_at | 0.595 | 0.019 | 0.256 | 0.357 |
| *BID* | BH3 interacting domain death agonist | 204493_at | 0.404 | 0.135 | 0.055 | 0.845 |
| *BIK* | BCL2-interacting killer (apoptosis-inducing) | 205780_at | 0.690 | 0.004 | 0.456 | 0.087 |
| *BIRC5* | Survivin | 202094_at | 0.402 | 0.137 | 0.196 | 0.483 |
| *BIRC5* | Survivin | 210334_x_at | 0.367 | 0.178 | 0.192 | 0.494 |
| *BIRC5* | Survivin | 202095_s_at | 0.292 | 0.290 | 0.150 | 0.593 |
| *CASP1* | caspase 1, apoptosis-related cysteine protease | 209970_x_at | -0.021 | 0.941 | 0.183 | 0.513 |
| *CASP1* | caspase 1, apoptosis-related cysteine protease | 211367_s_at | -0.110 | 0.695 | 0.163 | 0.563 |
| *CASP1* | caspase 1, apoptosis-related cysteine protease | 211368_s_at | -0.110 | 0.695 | 0.150 | 0.593 |
| *CASP1* | caspase 1, apoptosis-related cysteine protease | 206011_at | -0.215 | 0.441 | 0.030 | 0.916 |
| *CASP1* | caspase 1, apoptosis-related cysteine protease | 211366_x_at | -0.269 | 0.333 | 0.003 | 0.990 |
| *CASP10* | caspase 10, apoptosis-related cysteine protease | 210955_at | 0.366 | 0.179 | 0.114 | 0.686 |
| *CASP10* | caspase 10, apoptosis-related cysteine protease | 211888_x_at | 0.080 | 0.777 | 0.074 | 0.794 |
| *CASP10* | caspase 10, apoptosis-related cysteine protease | 205467_at | -0.223 | 0.424 | -0.068 | 0.809 |
| *CASP10* | caspase 10, apoptosis-related cysteine protease | 210708_x_at | -0.051 | 0.856 | 0.028 | 0.920 |
| *CASP2* | caspase 2, apoptosis-related cysteine protease | 209811_at | 0.606 | 0.017 | 0.431 | 0.108 |
| *CASP2* | caspase 2, apoptosis-related cysteine protease | 211140_s_at | 0.515 | 0.049 | 0.330 | 0.230 |
| *CASP2* | caspase 2, apoptosis-related cysteine protease | 208050_s_at | 0.484 | 0.068 | 0.322 | 0.242 |
| *CASP2* | caspase 2, apoptosis-related cysteine protease | 34449_at | 0.494 | 0.061 | 0.311 | 0.259 |
| *CASP2* | caspase 2, apoptosis-related cysteine protease | 209812_x_at | 0.434 | 0.106 | 0.193 | 0.491 |
| *CASP3* | caspase 3, apoptosis-related cysteine protease | 202763_at | 0.344 | 0.209 | 0.427 | 0.112 |
| *CASP4* | caspase 4, apoptosis-related cysteine protease | 213596_at | -0.494 | 0.061 | -0.229 | 0.411 |
| *CASP4* | caspase 4, apoptosis-related cysteine protease | 209310_s_at | -0.426 | 0.113 | -0.172 | 0.540 |
| *CASP5* | caspase 5, apoptosis-related cysteine protease | 207500_at | 0.319 | 0.247 | 0.223 | 0.425 |
| *CASP6* | caspase 6, apoptosis-related cysteine protease | 211464_x_at | -0.571 | 0.026 | -0.453 | 0.090 |
| *CASP6* | caspase 6, apoptosis-related cysteine protease | 209790_s_at | -0.265 | 0.340 | -0.325 | 0.237 |
| *CASP7* | caspase 7, apoptosis-related cysteine protease | 207181_s_at | -0.501 | 0.057 | -0.603 | 0.017 |
| *CASP8* | caspase 8, apoptosis-related cysteine protease | 213373_s_at | -0.448 | 0.094 | -0.140 | 0.620 |
| *CASP8* | caspase 8, apoptosis-related cysteine protease | 207686_s_at | -0.412 | 0.127 | 0.025 | 0.930 |
| *CASP8AP2* | CASP8 associated protein 2 | 222201_s_at | 0.128 | 0.649 | -0.149 | 0.595 |
| *CASP9* | caspase 9, apoptosis-related cysteine protease | 203984_s_at | 0.130 | 0.643 | 0.408 | 0.131 |
| *CASP9* | caspase 9, apoptosis-related cysteine protease | 210775_x_at | -0.431 | 0.109 | -0.071 | 0.802 |
| *CCND1* | cyclin D1 (PRAD1: parathyroid adenomatosis 1) | 208711_s_at | **0.578** | **0.024** | **0.686** | **0.005** |
| *CCND1* | cyclin D1 (PRAD1: parathyroid adenomatosis 1) | 208712_at | -0.153 | 0.587 | -0.182 | 0.516 |
| *CDKN2A* | CDKN2A | 209644_x_at | -0.120 | 0.671 | 0.124 | 0.660 |
| *CDKN2A* | CDKN2A | 211156_at | 0.063 | 0.823 | -0.089 | 0.752 |
| *CDKN2A* | CDKN2A | 207039_at | -0.061 | 0.830 | -0.011 | 0.970 |
| *CDKN2B* | CDKN2B | 207530_s_at | 0.561 | 0.029 | 0.461 | 0.083 |
| *cIAP1* | baculoviral IAP repeat-containing 2 | 202076_at | -0.405 | 0.135 | -0.301 | 0.276 |
| *cIAP2* | baculoviral IAP repeat-containing 3 | 210538_s_at | -0.188 | 0.503 | 0.182 | 0.516 |
| *CRADD* | CASP2 and RIPK1 domain containing adaptor with death domain | 209833_at | **0.752** | **0.001** | 0.417 | 0.122 |
| *CYCS* | cytochrome c, somatic | 208905_at | **0.752** | **0.001** | **0.544** | **0.036** |
| *CYP1A1* | cytochrome P450, family 1, subfamily A, polypeptide 1 | 205749_at | 0.375 | 0.168 | 0.354 | 0.195 |
| *CYP2D6* | cytochrome P450, family 2, subfamily D, polypeptide 6 | 215809_at | -0.297 | 0.283 | -0.358 | 0.190 |
| *CYP2D6* | cytochrome P450, family 2, subfamily D, polypeptide 6 | 207498_s_at | -0.304 | 0.271 | -0.192 | 0.494 |
| *CYP3A4* | cytochrome P450, family 3, subfamily A, polypeptide 4 | 205998_x_at | **-0.704** | **0.003** | **-0.545** | **0.036** |
| *CYP3A4* | cytochrome P450, family 3, subfamily A, polypeptide 4 | 205999_x_at | 0.673 | 0.006 | 0.497 | 0.060 |
| *CYP3A4* | cytochrome P450, family 3, subfamily A, polypeptide 4 | 208367_x_at | 0.209 | 0.455 | 0.127 | 0.653 |
| *CYP3A4* | cytochrome P450, family 3, subfamily A, polypeptide 4 | 210726_at | 0.195 | 0.486 | 0.011 | 0.969 |
| *CYP3A43* | cytochrome P450, family 3, subfamily A, polypeptide 43 | 211442_x_at | -0.308 | 0.265 | -0.408 | 0.131 |
| *CYP3A43* | cytochrome P450, family 3, subfamily A, polypeptide 43 | 211441_x_at | 0.602 | 0.018 | 0.185 | 0.509 |
| *CYP3A43* | cytochrome P450, family 3, subfamily A, polypeptide 43 | 211440_x_at | 0.396 | 0.144 | 0.065 | 0.818 |
| *CYP3A43* | cytochrome P450, family 3, subfamily A, polypeptide 43 | 207773_x_at | 0.265 | 0.340 | 0.020 | 0.943 |
| *CYP3A5* | cytochrome P450, family 3, subfamily A, polypeptide 5 | 205765_at | -0.011 | 0.968 | -0.447 | 0.095 |
| *CYP3A5* | cytochrome P450, family 3, subfamily A, polypeptide 5 | 214234_s_at | -0.074 | 0.792 | -0.300 | 0.277 |
| *CYP3A5* | cytochrome P450, family 3, subfamily A, polypeptide 5 | 214235_at | -0.128 | 0.650 | -0.227 | 0.416 |
| *CYP3A7* | cytochrome P450, family 3, subfamily A, polypeptide 7 | 211843_x_at | **0.713** | **0.003** | 0.488 | 0.065 |
| *CYP3A7* | cytochrome P450, family 3, subfamily A, polypeptide 7 | 205939_at | -0.236 | 0.397 | -0.177 | 0.528 |
| *DAPK1* | death-associated protein kinase 1 | 203139_at | -0.194 | 0.488 | -0.317 | 0.249 |
| *DAXX* | death-associated protein 6 | 201763_s_at | 0.464 | 0.081 | 0.365 | 0.182 |
| *DCK* | deoxycytidine kinase | 203302_at | **-0.666** | **0.007** | **-0.686** | **0.005** |
| *DcR3* | regulator of telomere elongation helicase 1 | 206092_x_at | 0.093 | 0.743 | -0.219 | 0.433 |
| *DcR3* | regulator of telomere elongation helicase 1 | 213829_x_at | 0.290 | 0.295 | 0.135 | 0.631 |
| *DcR3* | regulator of telomere elongation helicase 1 | 206467_x_at | 0.184 | 0.512 | 0.108 | 0.702 |
| *DFFA* | DNA fragmentation factor, 45kDa, alpha polypeptide | 203277_at | 0.679 | 0.005 | 0.393 | 0.147 |
| *DFFB* | DNA fragmentation factor, beta (caspase-activated DNase) | 206752_s_at | 0.409 | 0.130 | 0.569 | 0.027 |
| *DHFR* | Dihydrofolate Reductase | 202532_s_at | -0.432 | 0.108 | -0.548 | 0.034 |
| *DHFR* | Dihydrofolate Reductase | 202534_x_at | -0.302 | 0.274 | -0.500 | 0.058 |
| *DHFR* | Dihydrofolate Reductase | 48808_at | -0.109 | 0.700 | -0.343 | 0.211 |
| *DHFR* | Dihydrofolate Reductase | 202533_s_at | -0.088 | 0.754 | -0.193 | 0.491 |
| *ETV6* | ETV6 | 205585_at | -0.284 | 0.304 | -0.235 | 0.399 |
| *FADD* | Fas (TNFRSF6)-associated via death domain | 202535_at | 0.316 | 0.251 | 0.256 | 0.357 |
| *FAS* | Fas (TNF receptor superfamily, member 6) | 204780_s_at | 0.371 | 0.173 | 0.601 | 0.018 |
| *FAS* | Fas (TNF receptor superfamily, member 6) | 216252_x_at | 0.270 | 0.330 | 0.565 | 0.028 |
| *FAS* | Fas (TNF receptor superfamily, member 6) | 204781_s_at | 0.241 | 0.388 | 0.556 | 0.032 |
| *FAS* | Fas (TNF receptor superfamily, member 6) | 215719_x_at | 0.292 | 0.291 | **0.546** | **0.035** |
| *FAS-L* | Fas ligand (TNF superfamily, member 6) | 210865_at | 0.200 | 0.474 | 0.263 | 0.344 |
| *FAS-L* | Fas ligand (TNF superfamily, member 6) | 211333_s_at | -0.106 | 0.707 | -0.051 | 0.856 |
| *FLASH* | CASP8 associated protein 2 | 222201_s_at | 0.128 | 0.649 | -0.149 | 0.595 |
| *FLIP* | CASP8 and FADD-like apoptosis regulator | 208485_x_at | -0.137 | 0.627 | 0.193 | 0.490 |
| *FLIP* | CASP8 and FADD-like apoptosis regulator | 211862_x_at | -0.189 | 0.500 | 0.139 | 0.621 |
| *FLIP* | CASP8 and FADD-like apoptosis regulator | 209939_x_at | -0.104 | 0.711 | 0.131 | 0.641 |
| *FLIP* | CASP8 and FADD-like apoptosis regulator | 214618_at | 0.078 | 0.783 | 0.115 | 0.684 |
| *FLIP* | CASP8 and FADD-like apoptosis regulator | 210563_x_at | -0.169 | 0.547 | 0.082 | 0.772 |
| *FLIP* | CASP8 and FADD-like apoptosis regulator | 211317_s_at | -0.289 | 0.297 | 0.063 | 0.824 |
| *FLIP* | CASP8 and FADD-like apoptosis regulator | 209508_x_at | -0.265 | 0.341 | 0.052 | 0.854 |
| *FLIP* | CASP8 and FADD-like apoptosis regulator | 211316_x_at | -0.393 | 0.147 | -0.048 | 0.865 |
| *FOLH1* | folate hydrolase (prostate-specific membrane antigen) 1 | 217487_x_at | -0.393 | 0.148 | -0.446 | 0.096 |
| *FOLH1* | folate hydrolase (prostate-specific membrane antigen) 1 | 217483_at | -0.132 | 0.639 | -0.280 | 0.311 |
| *FOLH1* | folate hydrolase (prostate-specific membrane antigen) 1 | 205860_x_at | -0.254 | 0.362 | -0.238 | 0.393 |
| *FOLH1* | folate hydrolase (prostate-specific membrane antigen) 1 | 215363_x_at | -0.116 | 0.681 | 0.031 | 0.914 |
| *FPGS* | folylpolyglutamate synthase | 202945_at | 0.647 | 0.009 | 0.490 | 0.064 |
| *GLUL* | glutamate-ammonia ligase (glutamine synthase) | 200648_s_at | -0.351 | 0.200 | -0.504 | 0.056 |
| *GLUL* | glutamate-ammonia ligase (glutamine synthase) | 217202_s_at | -0.330 | 0.229 | -0.476 | 0.073 |
| *GLUL* | glutamate-ammonia ligase (glutamine synthase) | 215001_s_at | -0.378 | 0.165 | -0.458 | 0.086 |
| *GSTM1* | glutathione S-transferase M1 | 204550_x_at | 0.192 | 0.494 | 0.355 | 0.195 |
| *GSTM1* | glutathione S-transferase M1 | 215333_x_at | 0.141 | 0.617 | 0.312 | 0.258 |
| *GSTP1* | glutathione S-transferase pi | 200824_at | 0.109 | 0.699 | 0.028 | 0.920 |
| *GSTT1* | glutathione S-transferase theta 1 | 203815_at | 0.498 | 0.059 | 0.562 | 0.029 |
| *HOXA9* | HOXA9 | 209905_at | 0.437 | 0.103 | 0.043 | 0.880 |
| *HRK* | harakiri, BCL2 interacting protein (contains only BH3 domain) | 206864_s_at | -0.243 | 0.382 | -0.458 | 0.086 |
| *HRK* | Harakiri, BCL2 interacting protein (contains only BH3 domain) | 206865_at | -0.162 | 0.564 | -0.258 | 0.354 |
| *HSP27* | heat shock 27kDa protein 1 /// Meis1, homolog 3 (mouse) | 201841_s_at | -0.303 | 0.273 | -0.451 | 0.091 |
| *IL10* | interleukin 10 | 207433_at | -0.266 | 0.339 | 0.142 | 0.614 |
| *LIVIN* | baculoviral IAP repeat-containing 7 (livin) | 220451_s_at | -0.451 | 0.091 | -0.379 | 0.163 |
| *MCL1* | myeloid cell leukemia sequence 1 (BCL2-related) | 200796_s_at | -0.079 | 0.779 | 0.278 | 0.317 |
| *MCL1* | myeloid cell leukemia sequence 1 (BCL2-related) | 200798_x_at | -0.063 | 0.823 | 0.260 | 0.350 |
| *MCL1* | myeloid cell leukemia sequence 1 (BCL2-related) | 200797_s_at | -0.125 | 0.658 | 0.226 | 0.418 |
| *MCL1* | Myeloid cell leukemia sequence 1 (BCL2-related) | 214057_at | -0.037 | 0.895 | 0.190 | 0.499 |
| *MCL1* | Myeloid cell leukemia sequence 1 (BCL2-related) | 214056_at | -0.260 | 0.349 | 0.045 | 0.874 |
| *MGMT* | O-6-methylguanine-DNA methyltransferase | 204880_at | 0.088 | 0.756 | -0.041 | 0.883 |
| *MTHFD1* | methylenetetrahydrofolate dehydrogenase (NADP+ dependent) 1 | 202309_at | 0.556 | 0.031 | 0.333 | 0.225 |
| *MTHFD2* | methylenetetrahydrofolate dehydrogenase (NADP+ dependent) 2 | 201761_at | 0.469 | 0.078 | 0.575 | 0.025 |
| *MVP* | Major vault protein | 202180_s_at | -0.150 | 0.593 | 0.191 | 0.494 |
| *MYC* | v-myc myelocytomatosis viral oncogene homolog (avian) | 202431_s_at | 0.484 | 0.068 | 0.233 | 0.404 |
| *NAIP* | NLR family, apoptosis inhibitory protein | 204861_s_at | -0.533 | 0.041 | -0.504 | 0.056 |
| *NAIP* | NLR family, apoptosis inhibitory protein | 204860_s_at | -0.330 | 0.230 | -0.404 | 0.135 |
| *NOD1* | nucleotide-binding oligomerization domain containing 1 | 221073_s_at | 0.374 | 0.170 | 0.364 | 0.182 |
| *NQO1* | NAD(P)H dehydrogenase, quinone 1 | 201467_s_at | 0.372 | 0.172 | 0.319 | 0.246 |
| *NQO1* | NAD(P)H dehydrogenase, quinone 1 | 210519_s_at | 0.319 | 0.246 | 0.295 | 0.285 |
| *NQO1* | NAD(P)H dehydrogenase, quinone 1 | 201468_s_at | 0.279 | 0.314 | 0.284 | 0.305 |
| *NR3C1* | nuclear receptor subfamily 3, group C, member 1 | 216321_s_at | -0.557 | 0.031 | -0.449 | 0.093 |
| *NR3C1* | nuclear receptor subfamily 3, group C, member 1 | 201865_x_at | -0.563 | 0.029 | -0.420 | 0.119 |
| *NR3C1* | nuclear receptor subfamily 3, group C, member 1 | 201866_s_at | -0.557 | 0.031 | -0.393 | 0.147 |
| *NR3C1* | nuclear receptor subfamily 3, group C, member 1 | 211671_s_at | -0.501 | 0.057 | -0.368 | 0.178 |
| *Omi-HtrA2* | HtrA serine peptidase 2 | 203089_s_at | 0.402 | 0.138 | 0.636 | 0.011 |
| *Omi-HtrA2* | HtrA serine peptidase 2 | 211152_s_at | 0.379 | 0.164 | 0.495 | 0.061 |
| *P53* | P53 | 211300_s_at | -0.377 | 0.166 | -0.415 | 0.124 |
| *P53* | P53 | 201746_at | -0.415 | 0.124 | -0.386 | 0.156 |
| *PARP* | poly (ADP-ribose) polymerase family, member 1 | 208644_at | **0.721** | **0.002** | **0.677** | **0.006** |
| *PARPL1* | poly (ADP-ribose) polymerase family, member 4 | 202239_at | 0.324 | 0.239 | 0.407 | 0.132 |
| *PARPL2* | poly (ADP-ribose) polymerase family, member 2 | 215773_x_at | 0.467 | 0.079 | 0.201 | 0.472 |
| *PARPL2* | poly (ADP-ribose) polymerase family, member 2 | 214086_s_at | 0.429 | 0.110 | 0.153 | 0.585 |
| *PARPL2* | poly (ADP-ribose) polymerase family, member 2 | 204752_x_at | 0.459 | 0.085 | 0.147 | 0.600 |
| *PECAM-1* | platelet/endothelial cell adhesion molecule (CD31 antigen) | 208981_at | -0.091 | 0.748 | -0.127 | 0.651 |
| *PECAM-1* | platelet/endothelial cell adhesion molecule (CD31 antigen) | 208983_s_at | -0.005 | 0.985 | -0.066 | 0.814 |
| *PECAM-1* | platelet/endothelial cell adhesion molecule (CD31 antigen) | 208982_at | -0.049 | 0.861 | -0.046 | 0.869 |
| *PMAIP1 // NOXA* | phorbol-12-myristate-13-acetate-induced protein 1 | 204286_s_at | -0.442 | 0.099 | -0.451 | 0.091 |
| *PMAIP1 // NOXA* | phorbol-12-myristate-13-acetate-induced protein 1 | 204285_s_at | -0.234 | 0.402 | -0.364 | 0.182 |
| *RB1* | RB1 | 203132_at | -0.516 | 0.049 | -0.281 | 0.310 |
| *RB2* | RB2 | 211540_s_at | 0.316 | 0.252 | -0.070 | 0.804 |
| *RIPK1* | receptor (TNFRSF)-interacting serine-threonine kinase 1 | 209941_at | 0.220 | 0.431 | 0.423 | 0.116 |
| *SMAC* | diablo homolog (Drosophila) | 219350_s_at | **0.753** | **0.001** | **0.572** | **0.026** |
| *TANK* | TRAF family member-associated NFKB activator | 209451_at | -0.426 | 0.114 | -0.141 | 0.615 |
| *TANK* | TRAF family member-associated NFKB activator | 210458_s_at | -0.167 | 0.553 | 0.112 | 0.690 |
| *TANK* | TRAF family member-associated NFKB activator | 207616_s_at | -0.311 | 0.259 | -0.015 | 0.958 |
| *TAP1* | transporter 1, ATP-binding cassette, sub-family B (MDR/TAP) | 202307_s_at | -0.194 | 0.489 | 0.146 | 0.605 |
| *TAP2* | transporter 2, ATP-binding cassette, sub-family B (MDR/TAP) | 204770_at | -0.038 | 0.894 | 0.189 | 0.500 |
| *TAP2* | transporter 2, ATP-binding cassette, sub-family B (MDR/TAP) | 208428_at | 0.155 | 0.582 | 0.129 | 0.647 |
| *TNF* | tumor necrosis factor (TNF superfamily, member 2) | 207113_s_at | 0.251 | 0.367 | 0.233 | 0.403 |
| *TNF* | tumor necrosis factor (TNF superfamily, member 2) | 207113_s_at | 0.251 | 0.367 | 0.233 | 0.403 |
| *TNFR1* | tumor necrosis factor receptor superfamily, member 1A | 207643_s_at | 0.438 | 0.102 | 0.258 | 0.353 |
| *TNFR2* | tumor necrosis factor receptor superfamily, member 1B | 203508_at | 0.170 | 0.544 | 0.352 | 0.198 |
| *TOP2A* | topoisomerase (DNA) II alpha | 201291_s_at | -0.470 | 0.077 | -0.534 | 0.040 |
| *TOP2A* | topoisomerase (DNA) II alpha | 201292_at | -0.236 | 0.398 | -0.399 | 0.141 |
| *TOP2B* | topoisomerase (DNA) II beta 180kDa | 211987_at | -0.387 | 0.154 | -0.442 | 0.099 |
| *TPMT* | thiopurine S-methyltransferase | 203672_x_at | 0.378 | 0.164 | 0.474 | 0.074 |
| *TPMT* | thiopurine S-methyltransferase | 203671_at | -0.315 | 0.252 | -0.161 | 0.566 |
| *TRADD* | TNFRSF1A-associated via death domain | 1729_at | -0.205 | 0.464 | 0.218 | 0.435 |
| *TRAF1* | TNF receptor-associated factor 1 | 205599_at | -0.211 | 0.450 | 0.109 | 0.700 |
| *TRAF2* | TNF receptor-associated factor 2 | 204413_at | **0.539** | **0.038** | **0.571** | **0.026** |
| *TRAF3* | TNF receptor-associated factor 3 | 208315_x_at | **0.535** | **0.040** | **0.711** | **0.003** |
| *TRAF4* | TNF receptor-associated factor 4 | 211899_s_at | 0.548 | 0.034 | 0.475 | 0.074 |
| *TRAF6* | TNF receptor-associated factor 6 | 205558_at | 0.332 | 0.227 | 0.047 | 0.868 |
| *TRAIL* | tumor necrosis factor (ligand) superfamily, member 10 | 202687_s_at | 0.119 | 0.672 | 0.353 | 0.197 |
| *TRAIL* | tumor necrosis factor (ligand) superfamily, member 10 | 202688_at | 0.140 | 0.618 | 0.338 | 0.218 |
| *TRAIL* | tumor necrosis factor (ligand) superfamily, member 10 | 214329_x_at | 0.043 | 0.879 | 0.328 | 0.233 |
| *TRAIL-R2* | tumor necrosis factor receptor superfamily, member 10b | 209295_at | -0.015 | 0.957 | 0.089 | 0.751 |
| *TRAIL-R2* | tumor necrosis factor receptor superfamily, member 10b | 210405_x_at | -0.003 | 0.991 | 0.076 | 0.789 |
| *TRAIL-R3* | tumor necrosis factor receptor superfamily, member 10c | 206222_at | -0.370 | 0.175 | -0.369 | 0.175 |
| *TRAIL-R4* | tumor necrosis factor receptor superfamily, member 10d | 210654_at | 0.225 | 0.420 | 0.244 | 0.380 |
| *TRAMP* | tumor necrosis factor receptor superfamily, member 25 | 219423_x_at | 0.251 | 0.366 | 0.160 | 0.568 |
| *TUCAN* | caspase recruitment domain family, member 8 | 204950_at | -0.281 | 0.311 | 0.013 | 0.965 |
| *TWEAK-R* | tumor necrosis factor receptor superfamily, member 12A | 218368_s_at | 0.233 | 0.402 | 0.154 | 0.583 |
| *TYMS* | thymidylate synthetase | 217684_at | -0.370 | 0.174 | -0.368 | 0.177 |
| *TYMS* | thymidylate synthetase | 202589_at | -0.111 | 0.694 | -0.248 | 0.372 |
| *UGT1A10* | UDP glycosyltransferase 1 family, polypeptide A10 | 204532_x_at | -0.425 | 0.115 | -0.484 | 0.068 |
| *UGT1A10* | UDP glycosyltransferase 1 family, polypeptide A10 | 215125_s_at | -0.152 | 0.590 | -0.355 | 0.194 |
| *UGT1A10* | UDP glycosyltransferase 1 family, polypeptide A10 | 208596_s_at | 0.517 | 0.048 | 0.346 | 0.207 |
| *UGT1A10* | UDP glycosyltransferase 1 family, polypeptide A10 | 207126_x_at | -0.291 | 0.293 | -0.320 | 0.245 |
| *XIAP* | baculoviral IAP repeat-containing 4 | 206537_at | -0.276 | 0.320 | -0.334 | 0.224 |
| *XIAP* | baculoviral IAP repeat-containing 4 | 206536_s_at | -0.429 | 0.111 | -0.098 | 0.727 |
| *XRCC1* | X-ray repair complementing defective repair in Chinese hamster cells | 203655_at | -0.010 | 0.973 | -0.034 | 0.903 |
| *ZBTB7* | Pokemon | 219186_at | -0.256 | 0.357 | -0.252 | 0.365 |
| *ZBTB7* | Pokemon | 222082_at | 0.077 | 0.785 | -0.234 | 0.401 |
| *ZBTB7* | Pokemon | 213303_x_at | -0.080 | 0.777 | -0.092 | 0.745 |
| *ZBTB7* | Pokemon | 213299_at | -0.088 | 0.756 | -0.018 | 0.949 |

**SUPPLEMENTARY FIGURE LEGENDS**

**Figure S1.** **Comparison IC50 and doubling times in 15 T-ALL cell lines**

Comparison in 15 T-ALL cell lines of IC50 (log2) with doubling times, measured in hours (log2). A: for MPRED, giving a correlation of –0.86 and B: for DEX giving a correlation of –0.65.

**Figure S2. Summary of the known cellular effects of glucocorticoids (GCs).**

A large number of studies have examined the cellular effects of GC exposure in lymphoid cells. These include: [1] inhibition of glucose uptake^1^; [2] down-regulation of glycolytic enzymes including hexokinase II ^1-5^; [3] down-regulation of *MYC* ^6-8^; [4] inhibition of cholesterol synthesis^9^; [5] inhibition of cell cycle and proliferation^10-11^; [6] up-regulation of glutamine synthetase (*GLUL*)^12-13^ and glutaminase^14^; [7] transactivation and transrepression of multiple pathways, inhibition of protein synthesis, inhibition of RNA polymerase II ^7,8,10,15^; and [8] ultimate apoptotic cell death^2,8,15^. In the present study GC-resistance was associated with (a) up-regulation of glycolysis, OXPHOS, cholesterol biosynthesis, RNA polymerases, transcription factors, proliferation, and MYC-regulated pathways, (b) down-regulation of MLL expression and beta-oxidation pathways, (c) selective changes to amino acid and folate metabolism, and (d) sensitivity to mTOR pathway and ROS inhibitors. These changes indicate a proliferative metabolism that may serve to offset the adverse metabolic consequences of GC signaling.

*References: (1) Tome ME et al. Biochim Biophys Acta 2004;169:57-72; (2) Schmidt et al. Blood 2006;107:2061-9; (3) Tonko M et al. FASEB J 2001;15:693-9; (4) Tissing et al. Blood 2007;109:3929-35; (5) Holleman et al. N Engl J Med 2004;351:533-42; (6) Thompson et al. J Steroid Biochem Mol Biol 1999;69:453-61; (7) Obexer et al. Oncogene 2001;20:4324-36; (8) Ploner et al. J Steroid Biochem Mol Biol 2005;93:153-60; (9) Cutts & Melnykovych. Exp Cell Res 1987;168:95-104; (10) Renner et al. Curr Mol Med 2003;3:707-17; (11) Ausserlechner et al. Cell Death Differen 2004;11:165-74; (12) Harmon & Thompson. J Cell Physiol 1982;110,155-60; (13) Olkku et al. Bone 2004;34:320-9; (14) Dudrick et al. AmJ Surg 1993;165:34-9; (15) Schmidt et al. Cell Death Differen 2004;11:S45-55.*

*Abbreviations: VDAC, voltage dependent anion channel; G6P, glucose-6-phosphate; OXPHOS, oxidative phosphorylation; LDH, lactate dehydrogenase; ROS, reactive oxygen species; TCA, tricarboxylic acid (Krebs) cycle.*

**Supplementary Figure S1**


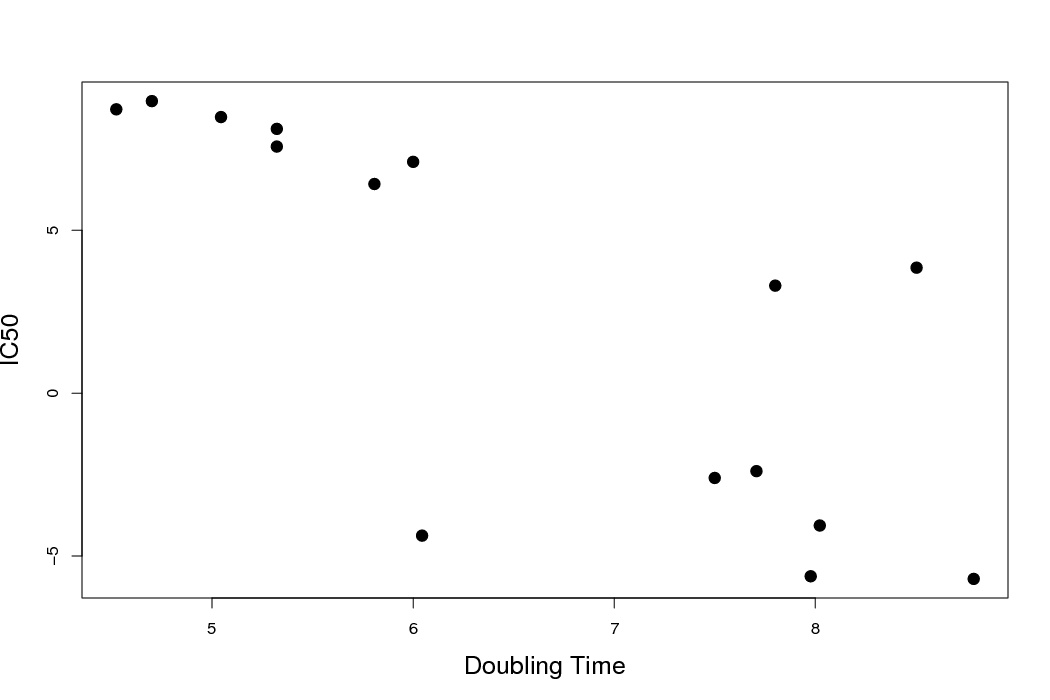


MPRED IC50 (log2)

Figure 1A


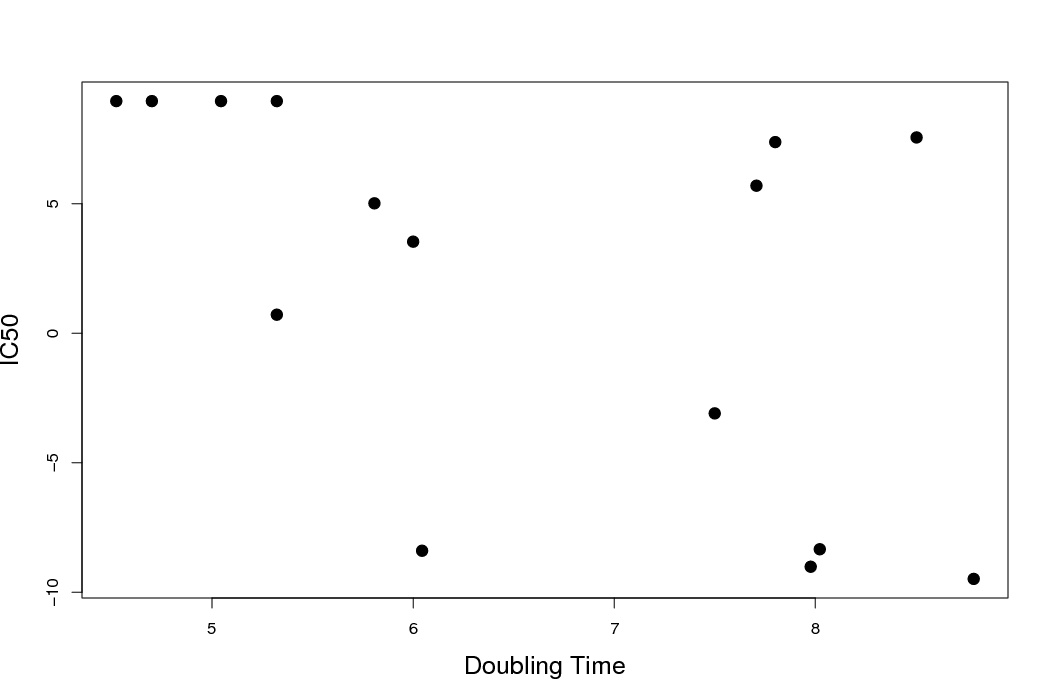


DEX IC50 (log2)

Doubling Time (Hrs, log2)

Figure 1B


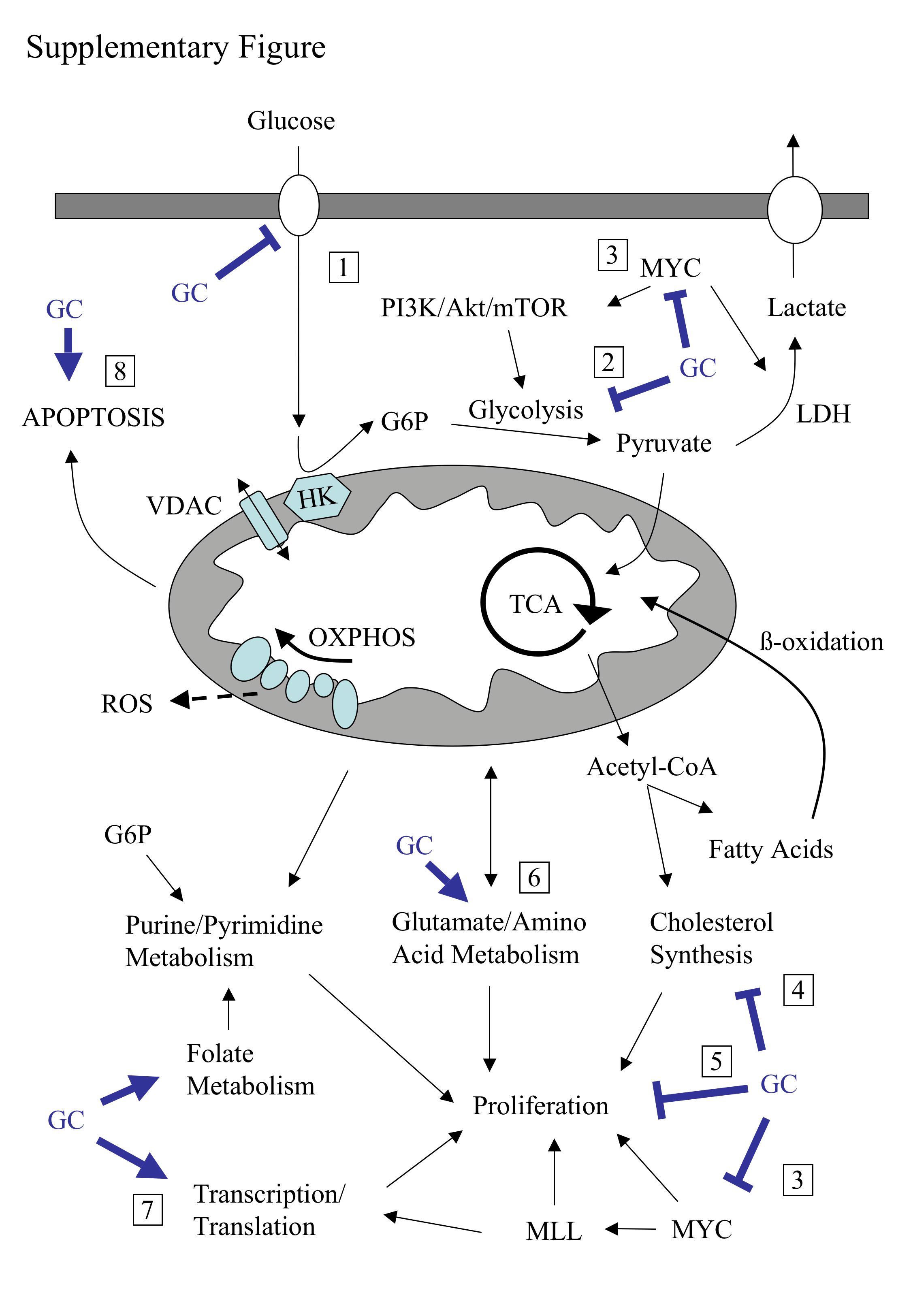


**Figure S2**

**SUPPLEMENTARY METHODS**

**Cell Lines**

The cell line panel has been previously described (Beesley et al. Br J Cancer 2006;95:1537-44; Beesley et al. Br J Haematol 2007;137:109-16; Kees et al. Mol Cancer Ther 2003;2:671-7) and comprised nine T-ALL lines derived in our own laboratory (PER cell lines), plus six additional T-ALL cell lines obtained from other sources. PER cell lines were derived from pediatric ALL bone marrow specimens according to the method previously described (Kees et al. Leuk Res 1987;11:489-98). DNA fingerprinting confirmed the identity of each of the cell lines (Beesley et al. Br J Cancer 2006;95:1537-44). CCRF-HSB2 (HSB2) cells were obtained from the American Type Culture Collection (ATCC); CCRF-CEM (CEM) from the Children’s Cancer Institute Australia for Medical Research, Sydney; JURKAT from the Basel Institute for Immunology, Switzerland; ALL-SIL from MRC Laboratory of Molecular Biology, Cambridge, UK; MOLT4 from the German Cancer Research Center, Heidelberg, Germany; DU.528 from the Division of Cancer Biology, Telethon Institute for Child Health Research, Perth. Cell lines were grown in RPMI-1640 supplemented with 2mM L-glutamine, 10nM 2-mercaptoethanol and 10-20% heat-inactivated fetal calf serum. The media for PER-cell lines contained additional non-essential amino acids and pyruvate, whilst 300 units/ml interleukin-2 is required for growth of PER-427 and PER-487 (Kees et al. Mol Cancer Ther 2003;2:671-7). Cell lines were cultured in the absence of antibiotics; testing for mycoplasma was routinely performed by PCR.

**Neutral Red Assay**

For testing of drugs identified by CMAP analysis (quercetin, resveratrol, LY294002, rapamycin) the neutral red assay (Borenfreund et al. Toxicol Lett 1985;24:119-24) was used since the anti-oxidant properties of resveratrol and quercetin have been shown to interfere with the accuracy of the MTT assay (Bernhard et al. Cancer Lett 2003;195:193-9). Briefly, cells were incubated for 48 hours with the indicated combinations of drug or DMSO (vehicle control), before transferral to a V-bottom plate and replacement of media with 100µl PBS and 10µl neutral red (Sigma, Castle Hill, NSW, Australia). Cells were incubated for 2 hours at 37°C, washed 3 times in PBS and resuspended in 100µl of a buffer consisting of 1% acetic acid 50% ethanol. These were then incubated for 10 minutes at room temperature before reading at 540nm and 630nm. Data points were measured in triplicates, and results averaged from 4-5 experiments (statistical analysis by repeated measures ANOVA).
