# Supplementary material for: Glucocorticoid resistance in T-lineage acute lymphoblastic leukaemia is associated with a proliferative metabolism
Source: Br J Cancer. 2009 May 12;100(12):1926–36. doi: 10.1038/sj.bjc.6605072 (PMC2714233; doi:10.1038/sj.bjc.6605072)
Supplement: Supplementary Data File 1 [file 6605072x2.doc]

ANDERSSON_CELL_LINES_AML_VS_ALL na SPFH1 FAH RGS19IP1 NCOA4 QSCN6 IGHG1 CITED2 GLRX MYO1F GLO1 MTF1 DUSP3 CPD PYGL ATP6AP2 IL7R CD9 AMH LGALS3BP ANXA1 STAR IGLC2

ANDERSSON_CELL_LINES_AML_VS_CML_BC na MR1 HIST1H2AL NUCB2 CTSG PSMB9 GSTO1 PRKCBP1 PLOD2 IL17R APOC1 MGAT2 PSMA6 MYL4 CFHL1

ANDERSSON_CELL_LINES_BALL_VSTALL na IGHG1 RPS28 FXYD2 GRAP2 RERE RAD21 MPHOSPH1 IRF4 TSC22D3 RNASET2 MAPRE1 PRDX1 SCARB1 IL7R ACTA2 HLA-DRB1 PRKRIR LOC90925 FOXO1A TYRP1 H2AFZ RPL12 RPL35A VCL PRKCBP1 TLE4 PLCG2 KPNA2 AMH CDKN1A IGLC2 PLK1 CREBBP STK6

ANDERSSON_AML_VS_RUNX1_CBFA2T1 na RBBP6 PSMB5 HLA-C UQCRC2 PSMB9 PLEC1 XRCC5 SEPT2 PAIP1 PTK7 DDAH1

ANDERSSON_CELL_LINES_AML_WITH_VS_WITHOUT_CBF_REARRANGEMENT na ARPC2 LGALS1 IDH1 ITGA5 XRCC5 CCNA2 ENG C1D RPS4X SERPINB1

ANDERSSON_CELL_LINES_PREB_WITH_VS_WITHOUT_TCF3_PBX1 na SNX3 UBE2E3

ANDERSSON_CELL_LINES_PREB_WITH_VS_WITHOUT_IGH@_MYC na MACF1 MEF2C AHCY EIF2S3 HSPD1 DUSP3 UPF3A IGLC2

ANDERSSON_CELL_LINES_PREB_WITH_VS_WITHOUT_MLL_REARRANGEMENT na PBX3 VAMP1 PCBD1

ANDERSSON_CELL_LINES_AML_WITH_VS_WITHOUT_MLL_REARRANGEMENT na PBX3 CST3 PLOD2

ANDERSSON_CELL_LINES_COMBINEDLINEAGES_MLL_VS_NOTMLL na NUCB2 PBX3 VAMP1 GCAT IL17R PME-1

CARIO_UP_IN_MRD_HR na RPS6KL1 SH2D1A SAMSN1 NUDT4 KLRK1 AMY2A FLJ14054 ZNF248 WDR35 GBP2 CYP4B1 STX12

CARIO_UP_DOWN_MRD_HR na GSTP1 FLJ20211 CDK6 SIVA SOX4 ATP5J ATF5 COL27A1 SOD1 KIAA0391 MCM5 OPN3 LGALS3BP PSMA6 QKI GCHFR CHEK1 CD81 BZRAP1 TNF PLXNB1 LOC116236 PBP SNRPC SUV39H2 CKLFSF7 SAG TIMP2 PEX26 SHBG HMGB3 FOXP4 CCDC28B WWP2 PHLDB2 ECHDC1 TNRC5 VAV1 ITIH3 PXMP2 HKE2 RHOJ MT LOC203069 MGC17943 AUTS2 C6ORF149 MYLK CHAF1B KLHL8 SERPING1 ELL3 PXDN NIN SNTA1 CNOT7 ZNF511 SPCS1 NDUFA13 SUZ12 C6ORF68 COL5A1 NARF SLC25A29 VRK1 AXL BVES M-RIP C10ORF56 CDCA1 CARHSP1 CREBL1 PPP2CA STK17A MLC1SA UGT3A1 CBR3 ICOSLG RNF40 MGC33948 MAD2L1 PFKL LOC150763 FRMD4B NUCKS1 MYBL2 BYSL MYADM TTC3 CYP19A1 CBX3 CDCA5 ASXL2 MPEG1 YIPF2 C6orf149 KIAA0179 KTN1 BATF TCEAL2 PRDX4 C6orf68 LZTFL1 JAG1 C9ORF77 CRNKL1 MRPL14 E2F6 SYNCRIP FLJ23514 MBD6 CDCA4 PLEKHE1 KCND1 LOC374946 CASC3 SFRS15 MGC5528 ACOT2 KIAA0339 FUT8 IMMT CETN2 ATXN2L PTK7 BCAR3 C1ORF117 TRIP3 CPXM HSPB2 C9orf77 TIE1 SLC35E3 C21orf18 CECR6 GPS1 MAGOH CIT MTVR1 SEMA6C KCTD15 BMP2 PARD3 RABAC1 KIAA1949 ELK3 DYRK3 RP9 BAT2 MGC39900 FLJ31952 BAX HSD17B8 ZNF292 ZCWCC2 PANX1 LOC199800 SLIT2 NS5ATP13TP2 TTK RING1 ZNF545 EBP E2F1 C21ORF18 FCGRT GAS2L1 LEF1 ADRBK1 POP7 ZNF198 SDC2 SAC3D1 MX1 STMN1 LOC338692 KIAA0683 LRCH4 ORC3L CCND3 RBM25 ELK1 SUMO3 NLGN2 FGFR1

CARIO_PAM_RESISTANCE_PREDICTOR na ICOSLG CSDA RABL4 ITIH3 HERC1 TIE1 LTA4H PABPC4 SEMA6C IRX6 LOC150763 PTPRC ELK3 COL27A1 DYRK3 YPEL2 PCDHGC3 KLRK1 EEF1G SERPING1 CD99 BATF TCEAL2 ITGA6 CSDE1 PLXNB1 TNF JAG1 NUDT4 E2F1 CREG1 GAS2L1 GBP2 LOC51326 MOBKL1A MAP4K3 SNCA MBD6 COBL GYG XRN2 WWP2 NLGN2 BNIP3L AKAP13

CARIO_COMBINED_MRD_HR na GSTP1 CDK6 SIVA SOX4 SAMSN1 SOD1 LGALS3BP CHEK1 BZRAP1 PBP LOC116236 SNRPC SUV39H2 CKLFSF7 HMGB3 WWP2 TNRC5 PXMP2 HKE2 LOC203069 MT CHAF1B C6ORF149 KLRK1 KLHL8 NIN SNTA1 ZNF511 SPCS1 NDUFA13 SH2D1A C6ORF68 COL5A1 SLC25A29 AXL BVES CDCA1 C10ORF56 CARHSP1 STK17A PPP2CA MLC1SA CBR3 UGT3A1 RNF40 MGC33948 MAD2L1 WDR35 PFKL LOC150763 NUCKS1 BYSL TTC3 CYP19A1 CDCA5 KIAA0179 KTN1 BATF C9ORF77 CRNKL1 MRPL14 MBD6 CDCA4 CASC3 AMY2A MGC5528 SFRS15 FUT8 CYP4B1 ATXN2L C1ORF117 C9orf77 C21orf18 SLC35E3 GPS1 CECR6 MAGOH KCTD15 BMP2 PARD3 RABAC1 ELK3 MGC39900 FLJ31952 BAX TTK NS5ATP13TP2 RING1 C21ORF18 POP7 SDC2 ZNF198 SAC3D1 LOC338692 LRCH4 ELK1 RBM25 FGFR1 SUMO3 FLJ20211 ATP5J ATF5 COL27A1 KIAA0391 MCM5 OPN3 PSMA6 QKI GCHFR CD81 PLXNB1 TNF SAG TIMP2 PEX26 SHBG FOXP4 CCDC28B PHLDB2 ECHDC1 STX12 ITIH3 VAV1 RHOJ MGC17943 AUTS2 MYLK SERPING1 FLJ14054 ELL3 PXDN CNOT7 SUZ12 NARF VRK1 M-RIP GBP2 CREBL1 ICOSLG FRMD4B MYBL2 MYADM CBX3 ASXL2 MPEG1 YIPF2 C6orf149 TCEAL2 PRDX4 LZTFL1 C6orf68 JAG1 ZNF248 E2F6 SYNCRIP FLJ23514 KCND1 LOC374946 PLEKHE1 ACOT2 KIAA0339 IMMT CETN2 PTK7 BCAR3 TRIP3 CPXM HSPB2 TIE1 CIT SEMA6C MTVR1 KIAA1949 DYRK3 RP9 BAT2 HSD17B8 ZNF292 ZCWCC2 LOC199800 PANX1 SLIT2 ZNF545 NUDT4 EBP E2F1 FCGRT GAS2L1 LEF1 ADRBK1 RPS6KL1 MX1 STMN1 KIAA0683 ORC3L CCND3 NLGN2

OBEXER_INDUCED_BY_DEX na FKBP5 BIRC3 PCYT2 NFKBIA PRKCB1 MTMR11 ACO1 AIM1 CCNF TYK2 TGFBR2 MGST2 SLA EPHA4 IL7R ABLIM1

OBEXER_REPRESSED_BY DEX na HRB2 GNA15 MOBK1B SLC9A1 UBE2G1 SKP1A PCBD FLOT2 SFRS11 CHD4 SLC16A1 PRR3 LOC157567 SUPT6H C21ORF106 GCLM CYB561 ZNF22 PRCC ZNF261 SNAPC1 RPGR SLC39A14 CCR7 CARS MCL1 DTYMK KDELR2 RCOR1 IRF5 TAF6 ITGA8 HSPA1B EZH2 FRAP1 PPP2R5A MTHFS MAP4 HNRPH1 STX1A CDKN2D IER2 NFE2L1 TUBB2 PPP5C SMARCD2 ZNF8 MMD CBX5 FASN ARMET SRPR IK DHFR /// DHFRP1 DGKD HA-1 RB1 ORC3L XBP1 RBM25 BAG1 ST13 HARS

OBEXER_COMBINED_DEX_UP_DOWN na GNA15 HRB2 MOBK1B SLC9A1 SKP1A UBE2G1 PCBD FLOT2 SFRS11 ACO1 CHD4 SLC16A1 PRR3 SLA LOC157567 SUPT6H EPHA4 C21ORF106 GCLM CYB561 ZNF22 BIRC3 PRCC SNAPC1 ZNF261 RPGR AIM1 TYK2 SLC39A14 TGFBR2 CCR7 CARS MCL1 DTYMK KDELR2 RCOR1 IRF5 NFKBIA ITGA8 TAF6 PRKCB1 HSPA1B EZH2 FRAP1 MGST2 PPP2R5A MTHFS MAP4 HNRPH1 IL7R STX1A CDKN2D IER2 NFE2L1 TUBB2 PPP5C SMARCD2 FKBP5 ARTS-1 PCYT2 MMD ZNF8 MTMR11 CBX5 FASN CCNF ARMET SRPR IK DHFR /// DHFRP1 DGKD HA-1 RB1 ORC3L XBP1 RBM25 BAG1 ABLIM1 HARS ST13

MUSCHEN_CD34POS_HSC_VS_PBCS na MSH5 RUNX2 IL10RA HGF ARHGAP26 TCL1A EREG HDAC2 DNTT EBF PBX1 ING4 TGFBR3 S100A8 HSPD1 PIM2 BLNK RAG2 ZNFN1A1 DLEU1 CD84 ZNF22 EP300 KYNU SMARCAL1 ABL1 SPTBN2 SHOX2 TCF3 MYB NFIC ARHI BCAP29 HSPA4 KLK3 MAEA BLK HK3 ARHGAP4 MPO TMSB10 GNRHR ELAVL1 SYNJ1 ATM CD19 RAG1AP1 CST8 SOS1 MAGOH HSPCA MCP CD83 SIAH1 NUP62 IL7R DRG1 LASS1 CD164 CASP8 NAP1L4 NFKB2 IL4R S100A9 NUDT4 RASA1 PBX3 FADD CD72 DNM1L POU2AF1 CDC2L1 STMN1 KIF5C RAB5A LIFR TRAF1 PAX5 FANCF TNFRSF25 KLK8 WNT16 ROR2

RHODES_NEOPLASTIC_META na TSTA3 FAP CKS2 MTHFD2 NME1 SNRPF SOX4 TRAF4 HNRPA2B1 SSR1 MMP9 ILF2 CBX3 IFNGR2 CCT5 NCBP2 HSPD1 TOP2A SSBP1 TARS CDKN3 PRDX4 LDHA PPP2R5C CANX PTMA RBM4 MRPS12 PSMC4 SMARCA4 KPNA2 KIAA0101 UBE2S KDELR2 TRA1 PLK1 CRIP2 MCM3 G3BP NONO E2F5 TPX2 TGIF CCT4 PAFAH1B3 HDAC1 AHCY IARS PSME2 HSPE1 OGT DVL3 COPB2 MRPL3 TUBB COL1A2 ARMET PAICS ACLY CDC2 DDX48 SNRPE NUP205 SDHC RFC4

RHODES_UNDIFFERENTIATED_META na PSMD14 CKS1B CKS2 FOXM1 MAD2L1 MTHFD2 IFI30 NME1 SLC16A1 RAD21 MYBL2 ILF2 CCT6A DLG7 TOP2A ADRM1 MCM6 H2AFX SSBP1 GCLM CDKN3 TRIP13 PRDX4 CDC6 EIF2S2 GGH KIF23 KPNA2 DPM1 KIAA0101 CNAP1 TMSB10 POLR2K UBE2S KIF14 MCM2 NSEP1 MCM3 CENPA GARS CEBPG EZH2 PSMD2 CTSL BIRC5 SLC7A5 KIF2C GPSM2 CDC20 HMGB2 H2AFZ CCNA2 TUBB4 TUBB PSMB7 COL1A2 PCNA BRRN1 RPA3 GAS6 UBE2C CDC2 MELK SEC61B TAP1 CCNB1 CXCL9 RFC4 NUDT1

FERRANDO_POS_CORR_WITH_MLL_TALL na CD3E CSDA FLNA H3F3A /// LOC440926 TUBA3 GDI1 RELA CBX3 PRDX1 FGR HYOU1 CLCNKA FKBP2 SPTBN1 KIAA0125 ZNFN1A1 BIN1 SREBF2 CBLB M11S1 ID3 FUT7 MRPS12 LENG4 C3F TDE1 ADCYAP1 TLE4 DDX5 PRKAR1B PTPN6 CLECSF2 AKAP1 IL32 TPM2 NFIL3 TCF7 VDAC1 GATA3 HLA-E DSTN CHI3L2 AEBP1 RALGDS LCP2 IL2RG VIM MYOM2 ELAVL3 SLC35B1 SLC7A5 STAT3 TSC22D1 MAD1L1 BTG1 CD47 SELL NUCB2 SSR4 FCGRT USF2 PRDX2 CDH4 ALPP WAS TRGC2 CD34 SPOCK2 HOXA9 TIAM1 TNFAIP3 PLEC1 PCGF4 LAPTM5 CTSH NRIP1

FERRANDO_NEG_CORR_WITH_MLL_TALL na RW1 AGTRL1 HMGA1 SLC9A1 LBR SLC29A1 DDX39 IGKC /// IGKV1-5 DNTT MCM5 PLP2 APLP2 POLD2 SORD PSMA6 TOP2A TRIP12 TYMS EIF3S9 HADHSC DOCK2 FYB VBP1 KHSRP HPRT1 CD2 CDK2 RANBP1 PSMB2 RRM1 LMNB1 KPNB1 BCLAF1 TFDP2 EPS15 ATP5G3 AP3S1 HIST1H4C PKMYT1 KIAA0101 POLA2 UBE2S IGFBP2 S100A4 PLCB2 TXN RPS4Y1 DUT CHAF1A PGAM1 NUP93 HMGN2 GOT2 SLC1A5 PRRX2 GLUL CSE1L MX2 EEF1D FKBP5 HMGB2 ACAA2 COX8A STRA13 GCGR PPP2R2A SDHA PCNA CD96 MAPKAPK3 GAPD UBE2C BARD1 PSMA2 RAFTLIN TCEB1 HINT1 SFRS3

FERRANDO_COMBINED_VS_MLL_TALL na PRSS3 CSDA FLNA LBR TUBA3 RELA DDX39 DNTT MCM5 PRDX1 APLP2 PSMA6 HYOU1 FKBP2 CLCNKA TOP2A TYMS KIAA0125 EIF3S9 DOCK2 FYB VBP1 M11S1 KHSRP ID3 CDK2 RANBP1 PSMB2 RRM1 LMNB1 ADCYAP1 TDE1 TFDP2 TLE4 ATP5G3 PTPN6 CLECSF2 AKAP1 PKMYT1 AP3S1 POLA2 UBE2S NFIL3 IGFBP2 S100A4 VDAC1 GATA3 CHI3L2 PLCB2 TXN DUT AEBP1 CHAF1A RALGDS NUP93 LCP2 IL2RG GOT2 MYOM2 ELAVL3 PRRX2 SLC7A5 EEF1D CD47 BTG1 MAD1L1 HMGB2 FKBP5 ACAA2 CD96 ALPP WAS CD34 TIAM1 SPOCK2 PCGF4 HINT1 SFRS3 CD3E RW1 AGTRL1 HMGA1 SLC9A1 H3F3A /// LOC440926 SLC29A1 GDI1 IGKC /// IGKV1-5 PLP2 CBX3 POLD2 SORD FGR SPTBN1 TRIP12 ZNFN1A1 BIN1 SREBF2 HADHSC CBLB FUT7 HPRT1 CD2 MRPS12 LENG4 C3F KPNB1 BCLAF1 EPS15 DDX5 PRKAR1B HIST1H4C IL32 KIAA0101 TPM2 TCF7 DSTN HLA-E RPS4Y1 PGAM1 HMGN2 VIM SLC1A5 SLC35B1 CSE1L GLUL MX2 STAT3 TSC22D1 SELL NUCB2 COX8A SSR4 FCGRT STRA13 GCGR PPP2R2A USF2 PCNA SDHA PRDX2 CDH4 MAPKAPK3 UBE2C GAPD TRGC2 PSMA2 BARD1 RAFTLIN TCEB1 HOXA9 TNFAIP3 PLEC1 LAPTM5 NRIP1 CTSH

STRING_MLL_TOP100_ASSOCIATIONS na C19ORF18 GMPS FUT4 FLI1 CD33 MEIS1 TAF4 MLL BRDT NCAM1 MLLT3 ARHGEF12 ASH2L MME MLLT10 MLLT6 SEPT7 MYC TLX3 FASLG ABL1 TCF3 MYO1F HRAS CBFB LPP E2F6 C19orf18 RUNX1T1 MLLT7 HCFC1 TP53 CDKN2A PRPF31 FNBP1 NUP98 PPIE CREBBP SH3GL1 CD19 MAX BCL6 MCRS1 EVER2 MLLT1 ELL RNF2 RUNX1 ETV6 LMO2 TEX10 FOXD4L3 ELL3 RBBP5 CD3D MYST1 FOXO3A BCR FOXO1A ZNF578 MLLT11 TYRO3 KIT CD7 SEPT6 MLLT4 MYH11 HOXA7 LAF4 LASP1 ASXL1 SENP3 CD34 TLX1 ANPEP HOXA9 CHD8 PCGF4 PRKAB2 SMARCB1

STRING_HOXA9_TOP100_ASSOCIATIONS na GALE GMPS CDK6 PROM1 GPHN MLLT1 ESR2 FGF17 MEIS1 EZH2 THRA WNT7A PBX1 MLL SEPT5 MLLT3 ARHGEF12 CD3G SMAD2 TOP2A PBX2 MLLT10 CD3D MLLT6 WHSC1L1 FZD6 DDX10 BCR PBX3 EED CBL ABI1 PLSCR1 LASP1 LAF4 NSD1 CD34 CCNG2 HOXA9 FNBP1 NUP98 RAP1GDS1 IFI16

STRING_MYC_TOP100_ASSOCIATIONS na PFKM EGR3 WRN MAPK14 RASD1 POLR3D MAZ CTNNB1 RAG2 BRCA1 RPS6KB1 BIN1 MYC MYOD1 FASLG NFATC1 ABL1 PIM1 SMAD1 TCF3 PTMA BCL2 MYB CDC25A TCF1 HRAS DDX18 YY1 PAM VEGF TLE1 MDM2 PIK3CA TP53 CDKN2A SRC T ID2 BCL2L1 MAPK8 CDKN2B CREBBP EGFR BCL6 MAX CCNT1 EVER2 SP1 CTBP1 CRK SMAD4 TRRAP JUN CCND2 MAPK3 AKT1 ERBB2 CCND1 TFAP2A PDGFB SP3 BBC3 MAP2K1 RAF1 NFYC MAD1L1 FALZ CDKN1B TERT ZBTB17 E2F1 NCL CTCF GTF2I TGFA THBS1 PFDN5 RBL1 DNMT3A PTK2B NFKB1 MAP3K12 TCEAL1 RB1 PAX5 FOS NFYB GRIN1 CDKN1A EGR2

STRING_IL7R_TOP100_ASSOCIATIONS na IL13 CSF2RB IL9 GHR JAK2 TM9SF2 LCK IL4 RAG1 SOCS3 PTPRC EBF SOCS5 CCL17 IL13RA2 IL23A CXCL5 IL19 RAG2 IL5RA SOCS1 ZNFN1A1 IL2RA SOCS7 IL24 IL12B CBLB CSF3 KITLG DCLRE1C TCF3 BCL2 IFNG CCL22 STAT5B TYK2 IGKC PTCRA IL6R CCR7 CRLF2 JAK3 PIK3CA PTPN6 IL22 CD79A CD8A SOCS2 SPI1 CSRP2 IL10 FYN PPBP CCL19 CD19 IL2 STAT5A CXCL13 TNFRSF1A CXCL3 IL15 IL2RG IL9R TNFRSF1B IL7R CSF1 VPREB1 IL21 IL4R IL2RB KIT CBL IGLC3 IL5 CBLC LYN IL8 BCL11A IL12A IL3 JAK1 TNFRSF4 CD34 PI3 RAB20 PTK2B IL7 IL11 PAX5 IL1R1 IL6 CSF1R FLT3

STRING_IL7_TOP100_ASSOCIATIONS na IL13 IL9 IL10RA LCK IL4 RAG1 PTPRC FIGF IL6ST IL21R NCAM1 IL13RA2 CCL17 IL3RA IFNGR2 CXCL5 CD40 IL5RA RAG2 IL2RA ENPEP TNF CSF3 KITLG BCL2 IFNG CD2 CCL22 STAT5B SPIB IL13RA1 STAT6 MUC1 IL10RB IL6R JAK3 SPN PIK3CA CD79A CD8A CD80 SPI1 FYN IL10 LTA CCL19 PPBP BCL2L11 EPOR CD19 IL22RA2 IL2 STAT5A LIF IL20RA CD44 CXCL3 TNFRSF8 IL1A IL15 IL2RG CSF2 IL9R IL7R BAX CSF1 VPREB1 IL22RA1 IL21 IL4R IFNGR1 BCR IL2RB IL12RB1 IL5 IL8 IL12RB2 IL15RA IL3 CCL5 CSF3R JAK1 CD34 IL11RA PTK2B IL11 IL7 ICAM1 PAX5 IL6 CSF1R CD28 FLT3

STRING_GPHN_TOP100_ASSOCIATIONS na GMPS GLRA1 GPHN GABARAP SLC17A8 AMPH GAD1 UBQLN1 ULK1 MLL STARD13 TNFSF14 ARHGEF12 MOCS2 GRIA1 DMD SUOX AGRN SLC6A5 XDH GLRB GAD2 SYP LAF4 LASP1 MOCS1 DLGAP1 DLG4 HOXA9 SV2A ADARB2 ENAH PHLDA2

STRING_CTGF_TOP100_ASSOCIATIONS na IL13 CD36 ANG ATF3 TGFB2 FGF18 RTN3 CKS2 IGF2R TIMP3 WNT1 HGF IL4 MEIS1 MMP9 PPARG PBX1 EGR1 CCL2 MMP19 COL4A2 IGFBP7 TIMP1 MMP2 TGFBR1 SERPINE1 ERBB4 PTGDS CTGF FRAT2 MYB RGS5 TGFB3 TGFBR2 HSPA7 VEGF TLE4 IFI44 SERPINH1 BGN IGFBP5 FMOD RARRES1 C10ORF116 LRP1 BMP4 TPM1 HOXD1 GDF2 MAPK8 TGFB1 MMP28 NOV JUNB SP1 F2R CRK CRMP1 ANTXR2 INSL4 BMP2 UCN3 JUN MAPK3 SRD5A1 FGF2 IGFBP3 MMP1 INHBE AGTR1 VWF IER2 ADAMTS17 PDK3 OCLN GABRE BTG1 CCDC2 NDRG1 AGT MBTPS1 UBE2C CDK10 THBS1 FOXE3 EDA ACTG2 ITM2B F2RL1 ACTB

STRING_BSG_TOP100_ASSOCIATIONS na PELO ALCAM QSCN6 SLC16A1 PTPRC IL17R ITGB1 TIMP1 MMP2 CD81 SSPN ITGA6 CD84 DAF SDC1 KAI1 SELPLG TNFAIP2 CD48 TIMP2 SPN MMP15 MCAM ITGAV MMP14 ITGB3 LAMP3 PLAUR CD44 DHRS3 CD58 CD63 CD151 SGCB MMP1 ITGA7 CD164 PPIA SLC7A5 CD47 CD59 BST1 CD109 HN1 BSG TRIM5 TRHR SLC16A7 SFRP2 CD97 ICAM1 SDCBP2 TAP1

STRING_LAMP1_TOP100_ASSOCIATIONS na AP1M1 SELP RPL14 IGF2R NIPSNAP3B VPS28 AP2M1 CD63 CD151 JUN HPS3 RAB4A RAB31 LGALS3BP GGA3 LAMP1 TFAP2A CD164 HSPA5 CD81 DDEF1 CANX TYRP1 SCARB2 TGOLN2 SPIC PRSS1 AP2B1 EEA1 CENTG2 TFRC SLC36A1 RAB5A NPC1 HPS1 AP3B1 ENTPD4 TYR

STRING_GTF2F1_TOP100_ASSOCIATIONS na SF3B4 TCEB3 CPSF3 GTF2H1 SF3A1 CCNH TAF7 GTF2F2 SNRP70 CDK9 CPSF5 TAF1 CPSF1 FUS DHX38 SFRS6 TAF10 SNRPB HNRPU SUPT5H TAF12 GTF2H3 POLR2F ERCC3 CPSF2 POLR2G PCF11 ERCC2 SFRS4 U2AF1 TAF6 POLR2H SFRS1 ELL HNRPUL1 POLR2C GTF2H4 SF3B5 SNRPA MNAT1 NCBP1 GTF2B SRF SF3A2 POLR2A GTF2A1 HNRPL SFRS2 GTF2A2 SFRS3 POLR2E SNRPD1 METTL3 SNRPG SF3B14 SNRPF HNRPA2B1 SUPT4H1 CDK7 POLR2D C20ORF14 HNRPK POLR2I RBM8A SNRPD3 GTF2E1 SNRPA1 CTDP1 CCNT1 UPF3B TH1L MAGOH TAF4B HNRPH2 RNMT PCBP2 SFRS9 SSRP1 C20orf14 CCNT2 SNRPB2 GTF2E2 TAF9 PRPF4 RNPS1 NHP2L1 DNAJC8 RBMX GTF2F1 EFTUD2 TBP CSTF2 TCEB1 LSM2 SFRS5 TCEA1 TAF5 TCEB2 CSTF1

STRING_DCK_TOP100_ASSOCIATIONS na PDE10A ABCD4 PDE3A BRAP PDE1A PDE4A ZFP36 AK5 TYMS APRT AK3L1 AMPD2 HPRT1 PDE4B PRKCA SLC28A3 POLA ENTPD8 ADK SLC28A2 ABCC5 DCTD PDE9A PDE6A DCK CDA PDE3B DTYMK PDE6G AMPD1 POLG MLLT2 AADAC PDE6H DCD ANXA3 USF1 PDE6C SP1 AMPD3 PDE5A DDC PDE1B PDE7B CTPS CALU ABCC4 PDE2A PDE4C DDN ENTPD3 PDE6D ABCB8 PDE8A PDE4D ENTPD1 PDE6B PRDX2 NT5C2 AK1 CKLFSF3 TAP1 TK1 PDE1C AK2 PDE8B PDE7A

STRING_MEIS1_TOP100_ASSOCIATIONS na MYCN MAFG HOXB4 HOXA3 PAX6 PROM1 SFRP1 FLI1 MEIS1 MAF MLL PBX1 MAFB HOXA10 BAMBI PBX2 HOXB13 DDX6 HOXD4 EMX2 MYOD1 ETS1 CTGF FRAT2 TCF3 ITPA MAFK HOXA7 GBX2 HOXA4 MAFF IGFBP6 HOXA9 NUP98 HOXB6 HOXD1 HOXD12 HOXB3 FLT3

STRING_PHB_TOP100_ASSOCIATIONS na SHMT1 NCOR1 NDUFS2 FOXD4L1 SMARCA2 HADHB E2F1 SHMT2 BRMS1 RBL2 SNRPB NDUFS8 ANXA2 PHB SP7 NME2 IPO7 TP53 HDAC1 RB1 CDCA7 SIN3A PRDX4

STRING_ELL_TOP100_ASSOCIATIONS na TCEB3 SKP1A GTF2H1 KCNC3 CCNH MLL GTF2F2 EAF1 RTKN MLLT3 CDK9 SUPT4H1 NCBP2 MLLT10 MLLT6 MYO1F CDK7 POLR2D POLR2I HNRPU VHL SUPT5H TP53 GTF2H3 ERCC3 POLR2F FNBP1 WHSC2 POLR2K EAF2 POLR2G CTDP1 ERCC2 SH3GL1 POLR2L CCNT1 TH1L POLR2H ELL SNX1 GTF2H2 POLR2C GTF2H4 SSRP1 POLR2B CCNT2 POLR2J MNAT1 NCBP1 TJP3 GTF2B MLLT4 POLR2A GTF2F1 TACSTD1 TBP TLX1 PDCD6IP TCEB1 TCEA1 RDBP TCEB2

STRING_P53_TOP100_ASSOCIATIONS na BLM WRN NR1H2 NME1 WT1 RASD1 TOPORS MLL HSPA9B ING4 CHEK1 TP53BP2 BRCA1 MYC ETS1 EP300 TNF PRKDC ABL1 BCL2 CDK2 XPC YY1 TOP1 VEGF TP73 HSPA4 MDM2 HIF1A TP53 PIN1 HD NF1 CDKN2A KRT8 CHEK2 RRM2B RAD51 ABCB1 BCL2L1 EEF2 MAPK8 CREBBP ATM EGFR CCNG1 S100B NFKBIA SP1 CRK HSPCA CD58 JUN S100A2 IGFBP3 BRCA2 TP53I3 GADD45A TP53BP1 SIAH1 HSPA1A BAX POU4F1 NQO1 BBC3 TIPARP FOXO3A STAT3 RPA1 DNCH1 CD59 BTAF1 TERT PMAIP1 PLK3 E2F1 SERPINB5 PCNA USP7 CDC2 THBS1 TBP ING1 ICAM1 MDM4 UBE2I CCNB1 TCEAL1 MAPK1 CDKN1A PTTG1 PCAF STK6 NPM1 PTEN DDB2

STRING_BCL2_TOP100_ASSOCIATIONS na BIK PSEN1 IL4 CASP6 RELA WT1 RASD1 BNIPL HAX1 CASP3 CASP7 BAG4 CFLAR CD40 RAD9A TP53BP2 EP300 BCL2 PAX8 HRAS CDK2 VEGF HSPA4 TP53 TEGT MITF MCL1 PPP2R4 BCL2L1 IL10 BID MAPK8 BCL2L11 HRK RUNX3 SNURF AVEN CASP9 IL2 STAT5A BCL6 HSPA8 BNIP2 TNFRSF1A PPP3CC RUNX1 JUN IL15 BMF IL2RG AKT1 BNIP3 FGF2 CCND1 IL7R BAX POU4F1 BBC3 CASP8 NFKB2 BNIP1 NR4A1 BCR RAF1 PMAIP1 KRAS2 E2F1 BCAP31 FADD BCL2L14 GLI2 YWHAB BAG3 JAK1 CDC2 BAD IL7 BAG2 CASP2 BAG1 BNIP3L CD28 APAF1

STRING_MTOR_TOP100_ASSOCIATIONS na UBE2L3 PDCD1 RHEB CORO1A RASD1 ATF5 ILK PIK3R1 EEF1A1 RHEBL1 NEK6 EIF4EBP1 SPARCL1 CFLAR MAP3K11 IRS1 RPS6KB1 ABL1 MAP7 AKT2 BCL2 RPS6KB2 PTK2 GJA1 VEGF YWHAQ IGF1 HIF1A RPS6 TP53 EIF4G3 RPS6KA1 EEF2 MAPK8 GALNT2 PIK3R2 EGFR RAC2 SLC9A3R1 RRAD GPHN CRK RUNX1 MAPK3 AKT1 RORC PRKAA2 EIF4E UBQLN1 FRAP1 INS AKT3 CCND1 SRRM2 INSR MAP2K1 STAT3 RAF1 CDC20 TSC1 INPPL1 SORCS3 EIF4G1 ARNT ROCK2 CDC2 STK11 PRKCD IRS2 PI3 CDK4 DOCK7 MAPK1 PPP2CA PTEN

STRING_AKT1_TOP100_ASSOCIATIONS na IGF1R CDC42EP1 RASD1 PIK3C2B ILK PIK3R1 TCL1B METTL1 RHOQ HSPCB IRS1 MYC RPS6KB1 PLXNB1 GNAI1 OCRL GSK3B PDPK1 CDC42 BCL2 CHUK GRB2 WASF1 ABI1 VEGF IGF1 TNFSF11 MDM2 RPS6 PIK3CA EPS8 IFIT1 ARHGEF2 GNB1 TNFRSF11A PIK3R2 CASP9 WASL RAC2 CRK HSPCA RAC1 PXN GSN IL1A JUN AKT1 FRAP1 INS CCND1 IKBKB PIK3CD CDC42EP4 PAK1 SHC1 FOXO3A RSN EDG5 RAF1 SLC2A4RG FOXO1A GFER MAP2K4 TSC1 CDKN1B TERT CBL INPPL1 MBTPS1 YWHAB WAS DOCK1 CYFIP2 EDG1 BAD RAB5A NFKB1 PPP2CA PLEK FGD1 NCF2 PIK3CG PTEN

STRING_MCL1_TOP100_ASSOCIATIONS na BIK ALK TP53I11 ABCD4 CASP1 CDK6 EVI2B GPR17 TRAF3 CASP6 POLR3D PIK3R1 CASP3 CASP7 BIRC2 CFLAR HDAC9 CD40 CHEK1 DUSP14 HTLF TNFRSF10B FASLG ABL1 BIRC3 BOK MNDA BCL2 CDK2 TNFSF10 CTSK IL6R VEGF IFI44 TP53 MCL1 BCL2L1 SFRP5 DIABLO MAPK8 TGFB1 BCL2L11 CASP9 STAT5A DEK DUT IER3 CRK JUN MAPK3 CCND2 CSF2 GADD45A TIAL1 CCND1 BIRC4 IER2 BAX CASP8 MAP2K1 STAT3 BCR RAF1 CASP4 PMAIP1 ABCB8 CTSS PCNA STAT1 STK17B PI3 BAD TNKS PDCD8 DDIT3 CDK4 TCEAL1 MAPK1 CCND3 CASP2 DAD1 IL6 CDKN1A DFFA BAG1 NPM1 APAF1

STRING_VCP_TOP100_ASSOCIATIONS na PTPRT AKAP4 FAF1 NFKBIA STX5A C4B VCP GNE PSMD4 PSMD2 PTPN13 NAP1L4 PTPRG UBB BRCA1 PTPN3

STRING_BIM_TOP100_ASSOCIATIONS na BIK BZRP DLC1 CBFA2T2 SPTLC1 CRADD IL21R SSTR5 STARD13 PIK3R1 CASP3 NMBR RAG2 MYC SSTR2 BOK BCL2 CORT BCL2A1 CDC25A PRKCA DDX18 TNFSF10 SNRPB PKDREJ TP53 SSTR1 SMS HSPG2 METAP2 MCL1 BCL2L1 DIABLO MAPK8 GAL3ST1 SSTR3 HRK BCL2L11 VDAC1 CASP9 SST DDB1 JUNB CCND2 IL15 JUN MAPK3 AKT1 BMF ZFPM1 CCND1 BIRC4 IL7R BAX PAK1 BBC3 CASP8 FOXO3A GAS1 PRKRIR NR4A1 GFER FOXO1A NGFB PMAIP1 ZBTB17 CASP5 IL3 DLGAP1 BANF1 BAD BCL2L2 NBL1 PPAN APAF1

STRING_GAS7_TOP100_ASSOCIATIONS na GAS8 GMPS CCL4 GAS7 GAS2 APRIN IL15 GAS6 CAPZA2 DDIT3 TLR6 MARCO GAS1 FGFR1 FGF8

STRING_MAX_TOP100_ASSOCIATIONS na MXD4 MYCN MAX MCRS1 TFDP1 SP1 RNF2 SMAD4 TRRAP TEX10 TAF4 MLL CBX3 ASH2L SP3 RBBP5 MYST1 RING1 MYC MXI1 MAD1L1 SPAG9 MYCL1 E2F6 TEAD1 SENP3 HCFC1 SMAD3 PRPF31 CHD8 AMPD1

STRING_MAD_TOP100_ASSOCIATIONS na HDAC2 SAP30 MYCN MAX CSPG6 MYCL1 HDAC1 PML AMPD1 SIN3A MYC

STRING_PUMA_TOP100_ASSOCIATIONS na BIK CDK6 CARM1 CBFA2T2 RBPSUH MAP3K8 PRDX1 ABI2 E2F3 IREB2 MYC EP300 BCL2 BCL2A1 DDX18 TNFSF10 TP73 BNC1 MDM2 TP53 PIN1 SET7 CDKN2A METAP2 TADA3L MCL1 BCL2L1 DIABLO MTA2 HRK BCL2L11 ATM CASP9 TADA2L TFDP1 TBL1X SP1 E2F5 CCND2 BMF BNIP3 TP53I3 GADD45A ENDOG CCND1 BAX BBC3 RNU2 GAS1 TP53INP1 CSPG2 CDKN1B TAF9 TERT XRCC4 PMAIP1 ZBTB17 E2F1 SAH CDC2L2 SAV1 USP7 MAPK9 BAD PDCD8 BCL2L2 CDK4 RB1 TCEAL1 CCND3 PLAGL1 CDKN1A PTTG1 BNIP3L PPM1D APAF1

STRING_OPHN1_TOP100_ASSOCIATIONS na ARHGEF7 PHF6 SLC6A8 TMEM1 TUBE1 OPHN1 RPS6KA3 RAC1 VCX GDI1 PAK3 RHOA ILK RIPK2 APLP2 MAPK8IP1 RPL23 ACSL4 ARHGEF6 STATIP1 EIF5B SLC2A4RG IL1RAPL1 AKT2 NLGN4X EIF3S10 PPP2R2A PARVA CDC27 SERPINH1 MSN SLC2A2 ATRX SPP1 FMR2 MECP2 ALAS2 TM4SF2 GULP1

STRING_NONO_TOP100_ASSOCIATIONS na SF3B1 THOC4 LMNB2 NCOR1 BCL6 SFPQ MYH10 CRY2 CLTC CST8 NONO C14ORF166 NR2E3 U2AF2 TIMELESS KRT6A PURA HSF1 PLEKHF2 MLLT3 TOP2B SIM1 TFE3 MATR3 HTATIP2 FOXO3A SIN3A PRCC ZNF261 NPAS1 WDR5 NCL POLR2A PARK7 SNRPB ARNT TLE1 C14orf166 MLLT7 DDX3X PARP1 PER1 PHKA1 ASCC3L1 PER2 ASPSCR1 POU2F1 ADORA2B

STRING_MLLT1_ENL_TOP100_ASSOCIATIONS na SH3GL1 NRGN GMPS CDK6 MLLT1 ELL SNX1 ABR LMO1 MLL RTKN ARHGEF12 MLLT10 ENG MLLT6 LYL1 BCR NR4A1 TLX3 TNF MLLT11 DCXR PBX3 TCF3 MYO1F SEPT6 MLLT4 MYH11 ABI1 LASP1 EPS15 MLLT7 TLX1 HOXA9 FNBP1 RAP1GDS1

STRING_MLLT2_AF4_TOP100_ASSOCIATIONS na BMP3 ALK ZNF578 ANXA3 ADH4 DIAPH2 KIT HRAS FMR1 RUNX1 CXCL10 MLLT3 DCK AREG MLLT2 CXCL1 BCR DNCH1

STRING_MLLT3_AF9_TOP100_ASSOCIATIONS na SH3GL1 GMPS BCOR LMO4 CDK6 SARA1 PROM1 RUNX1 FHL1 CD14 MLL HSF1 MLLT3 ARHGEF12 CRHBP MLLT10 MLLT6 RING1 BCR ZNF578 HDAC3 MLLT11 ABL1 DAB2IP CBFB MLLT4 MYH11 ALDH1A1 LASP1 RUNX1T1 PDAP1 TCF7L2 EPS15 MLLT7 TLX1 HOXA9 FNBP1 SMARCB1 MLLT2

STRING_MLLT4_AF6_TOP100_ASSOCIATIONS na COPS5 FLOT2 RASD1 MLL MRAS RYK GRB14 MLLT3 RIN1 MLLT10 MLLT6 EPHB2 RASGRP3 TCF3 HRAS CLDN5 CBFB RAPGEF1 RUNX1T1 BRD8 SRPK2 MLLT7 CTNNBL1 ARHGDIA CTNNA1 ENC1 RAP1GA1 EFNA5 TJP2 RRAS2 EPHB3 RAPGEF5 RALGDS ELL RUNX1 AKT1 EFNB1 LMO2 CDH2 OCLN BCR RAF1 SLC2A4RG MLLT11 TJP3 RIT1 F11R F2RL2 MLLT4 PVRL3 EPHA1 RAP2B SIPA1 TLX1 GRB7 PVRL1 TJP1 EPHB6 GRB10

STRING_MLLT6_AF17_TOP100_ASSOCIATIONS na CYR61 BRPF1 C19ORF18 EFNA5 NEDD5 ATF4 GMPS CDK6 MLLT1 ELL SRGAP2 MLL BRDT SEPT5 ARHGEF12 MLLT3 CACNB2 MLLT6 SEPT7 SIPA1L1 PIAS1 PCGF2 FOXD4L1 MLLT4 C19orf18 ABI1 LAF4 LASP1 LDOC1 HOXA9 ARHGAP1 UBE2I FNBP1

STRING_MLLT7_AFX1_TOP100_ASSOCIATIONS na BCL6 ZNF261 WDR5 MLLT4 SMAD4 PTER PAX3 MLL HSF1 MLLT7 MLLT3 SMAD3 PHKA1 FOXG1A CTNNB1

STRING_MLLT10_AF10_TOP100_ASSOCIATIONS na ARIH2 GMPS EVER2 CDK6 MLLT1 SSX1 ELL RUNX1 SRGAP2 CPSF6 LMO1 LMO2 MLL BRDT SEPT5 ARHGEF12 MLLT3 CACNB2 MLLT10 LYL1 ETV4 PICALM BCR TLX3 MLLT11 ABL1 PCGF2 TCF3 CBFB MLLT4 MYH11 ABI1 LASP1 LAF4 RUNX1T1 IL6R EPS8 TLX1 HOXA9 PDC SENP2 FNBP1

STRING_CDKN2A_TOP100_ASSOCIATIONS na MTAP ARFGEF2 WRN CDK6 GIT2 RELA GGA2 ARFGEF1 ARF1 GGA1 DNMT1 COPB IFI27 BRCA1 MYC PPP1R9B TWIST2 ABL1 CDK2 ARL4 TOP1 CENTG2 TP73 ARL2 ID1 MDM2 VHL HIF1A KIF23 TP53 PIK3CA ARHGAP4 NF1 ANKRA2 SMAD3 CDKN2A ARL3 CDKN2B RABEP1 GBF1 EGFR CHC1 CCNG1 CITED2 JUNB TFDP1 GLYAT PXN SMAD4 JUN CCND2 AKT1 ARFRP1 BRCA2 EPHB4 CCND1 INSR CDKN2D BAX ARF6 CENTD1 RING1 SLC2A4RG DDEF1 PLD1 ZNF578 CDKN1B CDKN2C TERT E2F1 ARL1 CTCF PCNA PSCD2 DNMT3B THBS1 MGMT RBL1 ARF4 DNMT3A CDK4 MDM4 RB1 TCEAL1 CCNB1 DCC CCND3 CDKN1A PLEK FHIT PTEN NPM1

STRING_IGJ_TOP100_ASSOCIATIONS na ITPR1 BMP3 ANXA3 ADH4 MTP TXNDC4 HRAS CTSS QDPR DCK AREG IGJ MLLT2 CXCL1

STRING_NR3C1_TOP100_ASSOCIATIONS na ONECUT1 VPS13B SMARCA2 IL9 SUMO1 STIP1 RELA JARID1A FGF1 PBX1 ADRB3 SMARCC2 TGFB1I1 ETS1 UBE2B STAT5B C20ORF14 HMGB1 SELENBP1 RNF14 MDM2 SMARCC1 HNRPU TP53 SMAD3 DNCI2 NCOR2 NR2F6 CTNNA1 CREBBP NR3C2 POU1F1 NCOR1 POU2F2 CALR STAT5A TXN CRSP2 DRD1 NR3C1 HSPCA JUN TIF1 NCOA2 DAP3 DDX54 HSPA1A GSTT2 NCOA1 ETS2 HSD17B3 NFKB2 C20orf14 STAT3 DUSP1 RAF1 ADRB2 CEBPB NCL IL12A PTGES3 ZBTB16 PPARGC1A MLH3 SMARCE1 UBE2I FOS POU2F1 NRIP1 SLC25A4 CSF1R SMARCB1

STRING_GLUL_TOP100_ASSOCIATIONS na ALDH5A1 AKAP9 GMPS C1ORF16 QARS RELA ALDH18A1 GOT2 GFPT1 DNCL1 PRDX1 EPRS FTCD GGTLA1 C3ORF15 GLUD1 C1orf16 CPSF4 SYT1 HSPD1 GLUL GCLM PRDX4 RAB7L1 ALDH4A1 GAD2 GOT1 PRDX3 STEAP GLUD2 ABAT GGT1 GLS GUSB MAPKAPK2 SGCE

STRING_PEX14_TOP100_ASSOCIATIONS na PEX10 PEX6 PEX13 NFE2 CTNNBIP1 PEX14 HDAC2 PHYH HDAC1 UBE4B PEX11B PGD KIF1B PEX1 PEX16 PHEX HDAC3 CORT ABCD1 PEX19 PTS ABCD3 PXMP3 POLL PEX26 PEX7 PEX5 PEX12 DFFA PEX11A

STRING_TYMS_TOP100_ASSOCIATIONS na SST NT5C P2RY4 DUT YES1 SHMT2 MTHFD2 ECGF1 TK2 RHO MTHFD1L GPR34 NT5C1B AMT GPR39 SSTR5 MTHFR P2RY1 SLC6A16 BIRC5 TYMS TRIM16 GPR44 ENTPD3 SHMT1 PTGDS UPF2 NT5C3 ITPA ENTPD1 NT5C2 POLA NYX ENTPD8 SLC28A2 MTX1 DCTD DCK NT5E DHFR EMP1 TK1 MS4A3 DTYMK SMOC1 C10ORF116 TPM1 S100A4

STRING_NF1_TOP100_ASSOCIATIONS na EVI2B FOXM1 FLOT2 GH1 NME1 RFC1 RASD1 CASK MRAS EIF4EBP1 AK3L1 BRCA1 NFIB REPIN1 GDNF NRAS NFIC TCF1 HRAS YY1 BRD8 TEAD1 VHL TP53 MSH2 NF1 SDHD CDKN2A CHEK2 EVI2A ADARB1 NF2 CYP17A1 C2orf3 GABRA5 MYCN USF1 PTPN11 RRAS2 SP1 SDC3 RUNX1 JUN THRA BRCA2 NME2 INS RNF135 CSF1 HDAC1 HNF4A TFAP2A SP3 CHRNB1 CENTA2 BLVRB RAF1 OPTC C2ORF3 TSC1 QRSL1 KRAS2 FOXD4L1 DNASE1 RGN OMG NFIA APOBEC3B GATA6 CRYBA1 MYEF2 CRLF3 SDHC TCEAL1 RB1 RET POU2F1 LPL

STRING_FUS_TOP100_ASSOCIATIONS na SF3B1 THOC4 SF3B4 PABPC1 SNRPD1 CPSF3 POLR2E SNRPG SNRPF SF3A1 HNRPA2B1 GTF2F2 SNRP70 CPSF5 DHX9 NCBP2 CPSF1 FUS SF3A3 DHX38 POLR2D SMC1L1 SF3B2 SFRS6 C20ORF14 SNRPB SNRPD2 HNRPK POLR2I CD2BP2 HNRPU RBM8A CREB3L2 SNRPD3 CDC40 POLR2F ASCC3L1 SNRPA1 POLR2K CPSF2 POLR2G RBM5 PCF11 SFRS4 SFRS7 POLR2L U2AF1 PRPF8 UPF3B POLR2H SFRS1 MAGOH PAPOLA U2AF2 HNRPH2 HNRPUL1 HNRPF PCBP2 POLR2C HNRPH1 SFRS9 SNRPA SF3B5 POLR2B C20orf14 SNRPB2 POLR2J HNRPD PRPF4 RNPS1 NCBP1 NHP2L1 HNRPM DNAJC8 SF3B3 SF3A2 RBMX GTF2F1 POLR2A HNRPR HNRPA0 PTBP1 EFTUD2 HNRPL LSM2 SFRS2 CSTF2 FUSIP1 SNRPE DDIT3 SFRS5 PCBP1 CSTF1 SFRS3

STRING_CCND1_TOP100_ASSOCIATIONS na ANG FOSL1 CARM1 CDK6 PSMC6 TK2 WNT1 NCOA3 ORC1L RELA RASD1 BTG2 CCNH ILK MYBL2 TAF1 E2F3 SYT1 HDAC9 CTNNB1 IFI27 BRCA1 MYC CDC6 EP300 ABL1 GSK3B BCL2 CDC42 CHUK PTK2 CDC25A TCF1 CDK2 JUND PRKCA CDK7 POLA TLE1 CCNE1 MDM2 CAV1 TP53 RRM2 PIN1 DHFR CDKN2A ID2 CTTN ORC4L CREBBP CDKN2B CDC45L STAT5A EVER2 TFDP1 JUNB CTBP1 SP1 CRK E2F5 SMAD4 CCND2 MAPK3 JUN AKT1 EIF4E FRAP1 ERBB2 CCNA1 GADD45A E2F4 CCND1 HDAC1 BBC3 STAT3 MNAT1 PMAIP1 CDKN1B E2F1 IL5 LEF1 PCNA TGFA CDC2 RBL1 CDK4 TCEAL1 RB1 FOS CCND3 CDKN1A FGFR1

STRING_RUNX1_TOP100_ASSOCIATIONS na MN1 C19ORF18 FRMD3 PDCD1 CBFA2T2 CD33 RARA C9orf64 RHD MLL MLLT3 C9ORF64 NTRK3 MLLT10 HDAC9 PADI4 MYC ETS1 EP300 ABL1 TCF3 BCL2 C9orf76 CBFB C19orf18 IL10RB RUNX1T1 ABL2 TP53 MYST4 NF1 CDKN2A GATA1 MPO KDR SPI1 NUP98 PML NCOR2 ABCB1 MLLT2 NCOR1 CD19 SLC9A3R1 MYST3 ETV7 CTBP1 GATA2 RUNX1 JUN ETV6 CSF2 PRAME HDAC1 DF C9ORF76 ZFPM2 BCR TLE2 SH3MD1 CEBPB SUV39H1 QRSL1 BPI TYRO3 MDS1 HDAC8 LEF1 RPL22 MLLT4 MYH11 IL3 CBFA2T3 CD34 TLX1 ANPEP PAX5 FOS CSF1R GART WBSCR5 FLT3 ELF4

STRING_BCL6_TOP100_ASSOCIATIONS na ALK BZRP MTA3 BCOR FUT4 HDAC4 SIN3B PTPRC CD38 MLL IL21R IRF4 CD5 CD24 MME MYBL1 HDAC9 CD40 IFI27 GRHPR FCGR2B MYC MXI1 HDAC7A EP300 PIM1 BIRC3 BCL2 HRAS JUND SYN1 S100A16 RUNX1T1 PPARD TNFRSF13B IGLL1 MLLT7 SPN TP53 RHOH CD8A CDKN2A SPI1 NCOR2 LCP1 BCL9 TNFRSF7 NCOR1 CD19 BCL6 EVER2 MS4A1 BCL7A JUNB NTRK1 TAF6 NONO SP1 CD44 ZNF482 TNFRSF8 JUN CCND2 RHOA CCND1 HDAC1 SIAT1 IL21 FOXO3A SIN3A LY6D BCR BCL10 NR1D1 ZBTB16 AICDA POU2AF1 KLHDC2 REL PAX5 EIF4A2 CCND3 LRMP XBP1 EBI2

STRING_CBL_TOP100_ASSOCIATIONS na ALDH7A1 FGFR2 JAK2 LCK SPRY2 RASD1 CSK MET SLA PDGFRB PIK3R1 GP6 UBE3A ITK HCK AOX1 ALDH1B1 IL2RA TNF ABL1 ALDH9A1 ALDH2 CD2 GRB2 SH3KBP1 RAPGEF1 SORBS1 TYK2 MUT EDD1 YWHAQ MDM2 JAK3 NFX1 BLK PTPN6 NEDD4 RNF25 MTR UBE2D2 SRC SYK FYN VAV2 PIK3R2 IL2 LAT EGFR EPOR CD19 PPP1R13B ZAP70 CUBN VAV1 PTPN11 PTPN22 CRK LTK MMAA AKT1 SPRY1 FCGR3A NDUFB3 INS TCN1 NCK1 INSR PLCG1 ALDH1A2 CSF1 SHC1 IL4R MMAB BCR RAF1 MMD CBL LYN CRKL BTK ALDH1A1 JAK1 SH3GL3 PTK2B SH3GL2 RET CSF1R CD28

STRING_PBX2_TOP100_ASSOCIATIONS na PPT2 MCRS1 ATP6V1G2 CSNK2B AGER TNXB MEIS1 NOTCH1 NR1I2 CLIC1 PBX1 BAT2 BAT5 HSPA1A HOXA10 BAIAP2 PBX2 BRD3 COL11A2 PSTPIP1 EMX2 IKBKAP COL5A1 AGPAT1 RPL4 SMYD3 TCF3 GPSM3 BTNL2 MEIS2 TCF1 TCF2 UGT2B15 AGPAT2 HOXB1 STK19 TLX1 HOXA9 DOM3Z RNF5 HSPA1L RDBP IL6 ENC1 TCF7

STRING_TAF15_TOP100_ASSOCIATIONS na C2orf3 C2ORF3 CSF2RB ZNF384 SLC4A3 NPAT SAFB FLI1 ELOVL2 H3F3A TCF12 NR4A3 TAF7 CREB3L2 FUSIP1 MMP7 NPEPPS TAF1 NR4A2 TAF15 ETV4 DUSP6

STRING_NUMA1_TOP100_ASSOCIATIONS na PIM1 PRKAR2A CSPG6 EVER2 CDK6 SMC1L1 NUMA1 CCNA2 RARA TIF1 EPB41L2 STAG1 RAD21 CCNH EPB41 TNKS NPAS2 MAK ARNTL PML CDKN1A NPM1

STRING_SSR3_TOP100_ASSOCIATIONS na SST SSR4 SSR3 EIF5A2 EIF4G1 PIK3CA TPH1 TP73L SMC4L1 SSR2 TIPARP TICAM2 SKIL

STRING_PIP5K1A_TOP100_ASSOCIATIONS na CDC2L5 ALS2CR7 RIPK4 IRAK2 CASK GRK4 IRAK1 PAK6 ARF1 EEF2K SYNJ2 CSNK1G3 PIK4CA OCRL TESK1 DCAMKL1 CSNK1G1 PCTK3 EIF2AK3 PIP5K1A EIF2AK2 PIK3CA PLCG2 PLD2 TLN1 PLCB4 SNF1LK DMPK PIK3R3 DYRK4 SYNJ1 PLCB3 GRK7 FN3K PLCB2 HIPK1 GSN PRKAA2 GRK5 HIPK2 RHOA DYRK3 PLCG1 GRK6 PIK3CD DYRK1B PIK3CB PRKAA1 ARF6 FASTK TTK BCR PLD1 PIK3C2G CDC2L2 PAK7 PLCD1 CSNK1G2 PLCB1 CDC7 DYRK2 GAK PIP5K2A DYRK1A PIP5KL1 LIMK2 PTEN

STRING_GAS1_TOP100_ASSOCIATIONS na ALCAM ATF4 EGR3 IL9 CBFA2T2 GAS7 H3F3A ANXA2 CCNB2 SLC26A4 PTCH CLDN7 IREB2 IFI27 RAG2 CLU MYC IKBKAP GAS8 MT1X CDC25A DDX18 APRIN RAB23 LIG4 ALG12 TFDP2 ANGPT2 TP53 METAP2 ATOH7 CIDEA BCL2L11 STAT5A CITED2 GAS2 GRK5 MOCOS GADD45A DHH CDKN2D BBC3 GAS1 COQ7 CCNC DLK1 NR4A1 TERT ZBTB17 XRCC5 CDH4 GAS6 GADD45G IL11 DDIT3 TRNT1 PAX5 HIP1 SLC25A18

STRING_BIRC7_TOP100_ASSOCIATIONS na PROK2 CASP9 KCNJ11 C21ORF33 DNM1 PMAIP1 DGCR14 ILVBL GBA CDC2L2 CSF2 CASP3 CASP7 DHFR CTAG2 BAX ACHE C21orf33 BBC3 DIABLO TNFRSF21 APAF1

STRING_LCK_TOP100_ASSOCIATIONS na CD3E SCAP1 OSIL CD3Z SLC39A2 LCK DOK2 RASD1 PTPRC CSK CD38 CD5 PIK3R1 IL2RA TUB FASLG DLG1 CDC42 CD2 MUC1 CD48 BRD8 ZNF271 JAK3 LNK PIK3CA FAS PTPN6 CD8A TEK HSPG2 PLD2 CCR5 SYK FYN NPY6R LAT IL2 ZAP70 VAV1 INPP5D PTPN11 UNC119 CTLA4 CD44 HSPCA CRK PXN LCP2 JUN IL2RG FCGR3A CD3G PLCG1 IL7R NEDD9 SHC1 STAT3 CXCL12 RAF1 IL2RB KIT PTPRH KHDRBS1 SH3BP2 CBL AXL LYN MAPRE2 RASGRF1 CXCR4 CD69 JAK1 CTNND2 PTK2B PTPRS IL7 FOS TNFAIP8 PLEK CD28

STRING_ACLY_TOP100_ASSOCIATIONS na STARD3 ACACB MDH1 PIP5K2B ACO1 ACACA BAAT RPL27 INS ARRB1 PGD SCD DUSP14 SREBF2 SREBF1 MDH2 JUP CS FASN AP2B1 ACLY G6PD CASC3 SHBG PC PCK1 G6PC LPL LHX1

STRING_CUL5_TOP100_ASSOCIATIONS na ATM TCEB3 WSB1 RBX1 SKP1A TFDP1 CUL5 ASB2 SATB1 HIP2 VCAM1 LRRC41 APOBEC3F CXCL11 SOCS1 CIAS1 ESAM SELE IQGAP1 RNF7 CDC20 PPIL5 KITLG UBE2M CCNF MRE11A APOBEC3B CYSLTR1 FEM1B CUL7 CDK10 MDM2 VHL NEDD8 TCEB1 ICAM1 TCEB2 PLAGL1 APOBEC3G

STRING_DHCR7_TOP100_ASSOCIATIONS na SHH MTSS1 EVER2 CYP11A1 CYP39A1 RPL41 KCNH6 CYP7A1 OTX2 SOAT2 CNR2 MAP2 SOAT1 APOC1 DHCR7 CEL COQ6 LIPA CYP7B1 HHIP CYP17A1 CLU

STRING_NOXA_TOP100_ASSOCIATIONS na BIK CDK6 MAP3K8 E2F3 HDAC9 HCCS BOK BCL2 BCL2A1 TNFSF10 MDM2 DDIT4 TP53 BAI1 MCL1 RRM2B BCL2L1 DIABLO HRK BCL2L11 ATM CASP9 DDB1 TFDP1 JUNB IRF1 E2F5 CCND2 CUL4A BMF HGS BNIP3 LRDD CCND1 ENDOG BAX BBC3 TP53INP1 PMAIP1 E2F1 PERP CDC2L2 CYFIP2 PDCD8 BCL2L2 CDK4 RB1 CCND3 PLAGL1 BNIP3L PPM1D APAF1 DDB2

STRING_NOTCH1_TOP100_ASSOCIATIONS na PSEN1 OLIG1 PSEN2 TREML1 LCK RBPSUH PCP4 RELA HDGF NOTCH1 EBF FABP7 TLE3 MAML2 SYT1 LCN2 ERBB4 MAML3 GSK3B JAG1 NUMB ELTD1 YY1 AGPAT2 VEGF SAP30 DTX1 NEUROG2 AMBN SMAD3 JAG2 ASCL1 RAB27A ATOH1 CDH5 LFNG SOX17 WNT7A ADAM17 PSARL HES7 SKP2 PBX2 FOLH1 COL5A1 TLE2 AGPAT1 PBX3 LEF1 STOM NES APP OLIG2 GJA4 GR6 FOXA2 CNTN1 MAML1 DLL1 DVL1 RRBP1 FBXW7 PCAF SEL1L

STRING_NOTCH2_TOP100_ASSOCIATIONS na WNT4 SFRP1 TREML1 WNT1 RBPSUH DDR2 IGSF2 SSR2 TLE3 GFI1 EMX2 GCLM JAG1 GSK3B NRAS COL6A2 DTX1 FOXC1 WNT2B GBP1 SYCP1 ERCC3 H2AFY2 JAG2 FCER1G ASCL1 FGF10 PCTK1 CCT3 ADORA3 SH2D1B CDC5L CSF2 EYA1 COL4A3 FZD5 ADAM10 HES7 RBP7 DF FZD6 PTGFR LMNA TLE2 PTGFRN KCND3 NOTCH2 COL6A3 GBP2 ABCD3 FZD4 LMX1B FCER2 CNTN1 SFRP2 MAP3K4 MAML1 DLL1 DVL1 S100A10 OR5AC2 COL6A1 MFNG EXTL2

STRING_CYP3A4_TOP100_ASSOCIATIONS na PPIF ALDH7A1 CYP4A11 SLCO1B1 PON2 CBX8 FOXM1 EPHX1 CYP2B6 ABCC2 ENTH CYP2E1 CYP2C18 CHST1 NAT2 CYP19A1 UGT1A@ KNTC2 ALDH1B1 NR1I3 ALDH9A1 ALDH2 CYP1B1 TCF1 CYB5 TST CYP4F11 CYP2J2 CYP3A5 EDD1 TOP1 ZNF161 CYP2C19 DDX3X HNF4G ATBF1 CYP2D6 UGT2B7 ABCB1 PGPEP1 CYP4B1 INDO NAP1L1 AZGP1 ELAC2 CHST3 CYP4F12 NR1I2 GSTM1 FMO5 ABCG2 CYP3A4 ALDH1A2 HNF4A CYP1A2 ABCC4 DEFA1 PON3 CYP1A1 CYP4F8 PGR POR ALDH1A1 PON1 CYP3A43 CYP2C8 FMO1 CYP2A6 SRD5A2 ZNF185 SLC38A6 CYP2C9 CYP3A7

STRING_ATM_TOP100_ASSOCIATIONS na BLM HUS1 WRN ROS1 TREX1 RFC1 WT1 PIK3R1 CASP3 EIF4EBP1 FANCC RAD9A H2AFX CHEK1 BRCA1 PMS2 PRKDC ABL1 RAD52 DCLRE1C CDC25A CDK2 HRAS SMC1L1 MLH1 G22P1 AP1B1 LIG4 TERF2 MDM2 TP53 MSH2 SERPINA1 CHEK2 PEX5 RAD51 IL10 PLK1 ATM RRAD NPAT SLC25A3 SP1 GLYAT SATB1 RBBP8 BRCA2 RAD17 INS FANCD2 GADD45A WEE1 TP53BP1 HDAC1 BAX BBC3 TIPARP RPA1 FANCG DDX41 PMAIP1 XRCC4 MSH6 PLK3 E2F1 RPA2 XRCC5 PCNA MRE11A RAD50 STK11 CDC2 BARD1 TERF1 PI3 NFKB1 RB1 TCEAL1 HINT1 FANCF CDKN1A RFC4 PPM1D

STRING_ATR_TOP100_ASSOCIATIONS na ORC2L POLE HUS1 MAT1A RRAD NR1H2 TREX1 ARIH1 EXO1 RFC1 RORC BRCA2 RAD17 FANCD2 EIF4EBP1 RAD18 UBE2V2 RAD9A CLSPN H2AFX CHEK1 CHTF18 UBR1 RPA1 CDC16 BRCA1 CDC20 MNAT1 FBXO18 RAD52 BUB3 REV3L PLK3 XRCC5 MLH1 MRE11A LIG4 RAD50 POLA TP53 MSH2 PI3 CHEK2 PVRL1 HINT1 RAD51 PTTG1

DAN_MULTIPLE_RES_10_DRUGS na RER1 CYR61 TSPAN3 FHL2 FIBP SQSTM1 CCT3 LAMB1 PFN2 EXT1 TOB2 PTTG1IP IGFBP3 SYBL1 NDUFB6 SERPING1 LDHB CAPN2 CCNC UCHL1 TSC22D1 PPP3CA UQCRB TUSC3 CD47 MLLT11 KRT7 CD59 NDRG1 ACTN4 MYL6 PPL OSTF1 AKR1C1 PGRMC1 AKR1B1 LGALS3 SFN ACOT2 CYFIP1 NOL3 MCL1 CTSH LIMK2 MIRAB13 CKB DDB2

DAN_MULTIPLE_RES_ VS_ANTHRACYCLINES na S100P KIAA0284 KRT19 MYBL2 ATP1A1 SHC1 PIM2 UCHL1 ELF3 TUSC3 KRT7 MLLT11 RGS19IP1 JUP OSTF1 AKR1C1 WDR68 G6PD PGRMC1 DSP AKR1B1 DDR1 SFN KRT8 CTSH LIMK2 DDB2

SCHRAETS_UP_IN_MLL_KNOCKOUT na MSX2 MMP3 HAS2 ALCAM WNT5A DCN TIMP3 GM2A FHL1 IL17R NCAM1 BTG3 TOB1 GALNT11 DMD ACTA2 CD24 MYBL1 HTATIP2 GPC4 CTGF PTGS2 TNC JUP RAMP3 DUSP9 KAI1 SPATA13 ITGA3 CYP51A1 MDM2 COL18A1 FAS SERPINE2 SH3BGRL2 DSC2 HOXD8 HOXA1 TSPAN7 CYR61 MMP10 EFNB2 NDN DNM1 CTNNAL1 CD44 MLLT1 TNNT2 PRSS23 CRMP1 PCDH7 PEG3 FKBP11 GNG11 PRKD3 ITGA7 FZD6 CBR1 TES NR4A1 CXCL12 CTSC D4S234E F11R MAPK12 SEMA3A PDE8A SLC8A1 PERP TMSB4X PAPSS2 TSPAN6 MPG NES SNAP91 DAB2 GAS6 MLF1 CTSZ RGS2 FOS AGTRAP IGSF4 CLCA1 TPD52L1 NID2

SCHRAETS_DOWN_IN_MLL_KNOCKOUT na HOXC8 WNT4 THBD SASH1 GREM1 PTPN22 CD53 CCL9 ARHGDIB GSTA4 C1QR1 NDRG4 FIGF LIMK1 PDGFRB RND2 MME APOB GAS1 THBS2 TWIST2 PDK1 ANGPT1 IL1RL1 AGT COL6A2 FOXC2 ACADM PITX2 COL6A3 CAP1 TGFBI SPRR1A TCF2 HFE GATA6 MGP IL1RN ALDH3A1 ITIH2 DFFB SRXN1 CTSH COL6A1

SCHRAETS_COMBINED_MLL_LIST na MMP3 HAS2 WNT5A GREM1 DCN TIMP3 C1QR1 FHL1 NDRG4 ACTA2 MME HTATIP2 GPC4 PDK1 ANGPT1 PTGS2 JUP PITX2 RAMP3 SPRR1A SPATA13 HFE COL18A1 FAS SH3BGRL2 SRXN1 DSC2 CYR61 MMP10 THBD DNM1 CTNNAL1 CD44 ARHGDIB MLLT1 TNNT2 PEG3 LIMK1 FKBP11 ITGA7 APOB CBR1 TES THBS2 NR4A1 CXCL12 CTSC CDKN1B AGT MAPK12 SEMA3A SLC8A1 TMSB4X TSPAN6 CAP1 TCF2 NES SNAP91 GATA6 MLF1 CTSZ MGP ITIH2 IGSF4 MSX2 HOXC8 WNT4 ALCAM SASH1 GSTA4 GM2A FIGF IL17R NCAM1 PDGFRB BTG3 TOB1 GALNT11 DMD CD24 RND2 MYBL1 TWIST2 IL1RL1 CTGF TNC COL6A2 FOXC2 ACADM DUSP9 KAI1 TGFBI ITGA3 CYP51A1 MDM2 IL1RN SERPINE2 HOXD8 HOXA1 TSPAN7 EFNB2 NDN PTPN22 CD53 CCL9 CRMP1 PRSS23 PCDH7 GNG11 PRKD3 FZD6 GAS1 D4S234E F11R PDE8A PERP COL6A3 PAPSS2 DAB2 MPG GAS6 ALDH3A1 DFFB FOS RGS2 CTSH COL6A1 AGTRAP CLCA1 TPD52L1 NID2

GLINSKY_BMI_1_PATHWAY na UBE2T FGFR2 MYH1 NUCB1 PRC1 LBR EPHX1 HNRPLL PRIM2A PCP4 CDCA8 CCNB2 CDH13 KLF3 CEBPD ITPR2 CES3 AURKA DSG2 TOP2A PLK4 ARHGAP5 TACC3 BRCA1 CDC6 CDCA3 BUB3 CKAP2 ALDH2 TRIM59 VAMP8 NUSAP1 CDO1 LAMB3 SYTL4 RRM1 BCAP29 DUSP16 BCAT2 DNA2L KIF23 HCFC1 PLA2G2A NEFH CST3 AGR2 DSC2 HMGB3 PCSK1N ECT2 CTTN PLK1 CYR61 TPM4 CD44 TNXB GCH1 BNIP3 PRIM1 GSTM1 CHAF1B RCN1 MYLK ANK3 CCND1 SMC4L1 NFE2L1 SUZ12 MGLL MCM7 HES6 BUB1 CCNA2 MKRN1 MYH11 STIM1 GTF2I LYPLA1 RBL1 RB1 PTBP2 TNFAIP8L1 GAA RFC4 BNIP3L USP22 SNCG RAB7 WSB1 ASPM CSPG6 CKS2 TMPO MAD2L1 USP1 MYBL2 CDCA5 DLG7 SQRDL SMAD2 NCDN SEPT4 KIF20A POLE2 LDHA CTGF HELLS GBX2 AOC3 CAND1 PDE9A SERPINE2 MKI67 ANLN ALDOC CRYL1 CSRP2 SMC2L1 MRVI1 DEK LIN9 CCT3 RNF2 SSR3 KCNK3 CDC25C LMO2 NPY CD83 BIRC5 HPGD MCM10 TTK MAPRE3 TOPBP1 CDC20 TAF9 KIF11 PAPSS2 RBMX RBP1 CDC7 DEFB1 STMN1 TEC FOS HAT1 WNT6 KIF4A

GLINSKY_20GENE_BMI_1_PATHWAY_LIST na USP22 FGFR2 ANK3 HCFC1 ASPM MKI67 CES3 BUB1 PAPSS2 RNF2 GBX2 MCM10

GLINSKY_COMBINED_TRAMP_PNS_CNS_CANDIDATES na USP22 SNCG FGFR2 ASPM EPHX1 RNF2 BNIP3 ANK3 CCND1 CES3 MCM10 POLE2 SUZ12 CTGF ALDH2 BUB1 PAPSS2 GBX2 RBP1 VEGF BGN DEFB1 HCFC1 MKI67 CD97 FOXA1 TEC WNT6

STAIB_KNOWN_P53_TARGETS_CURATED na BIK TP53I11 CASP1 SIVA CASP6 PLK2 BTG2 PRSS25 MMP2 CCNK TNFRSF10B SOD2 HRAS BAK1 TP73 GPX1 MDM2 EI24 DDIT4 BAI1 FAS DUSP5 DKK1 PML RRM2B C20orf10 ENC1 P53AIP1 C20ORF10 AMID IRF5 CCNG1 CX3CL1 CD82 TRIM22 IGFBP3 TP53I3 LRDD GADD45A BAX BBC3 CASP10 TP53INP1 PMAIP1 SERPINB5 FDXR SESN1 GDF15 DDR1 SFN CDKN1A PPM1D DDB2 APAF1 PTEN

STAIB_UP_DOWN_P53_GENES_SPER_NO_TREATMENT na TUBB4Q BCAR3 CYR61 ZNF250 FHL2 HSPA8 GABRG3 SLC7A7 HSPA1B PLK2 BTG2 NUAK2 GADD45A PHLDA3 HSPA1A SLC10A2 AMOTL2 SERPINE1 SRY BIRC3 CTGF PMAIP1 SLC37A4 EPHA2 PPIL5 C14ORF104 MYB HMGCL MAFF C14orf104 THBS1 FOXC1 GDF15 LOC115265 FBXO5 DUSP5 RGS2 MAT2A SAMHD1

STAIB_UP_DOWN_P53_GENES_H202_TREATMENT na EFNB2 FHL2 ANKRD10 JUNB KLF9 PEX3 JUN PLK2 BTG2 TRAF4 CNKSR1 TP53I3 POLH PHLDA3 NR2C2 ETS2 DBP CENPF FLT3LG KIAA0247 GPR87 ID2B S100G SLC12A6 MAN1C1 MDM2 FDXR ING1 NR1D2 GDF15 HOXA9 SESN1 PRKAB1 PER1 RGS2 PPM1D CHL1 DST ENC1

STAIB_UP_DOWN_P53_GENES_HU_TREATMENT na HMMR ASPM CENPA KIF18A DHRS3 H1FX PLK2 BTG2 CNKSR1 S100A2 TP53I3 HIST1H3J BITE POLH PIF1 PHLDA3 DLG7 ANAPC7 TP53INP1 DBP ASF1A TP53I5 SERPINB5 NEK2 NR1D1 ADAMTS7 ARHGAP18 PLXNB3 FDXR SESN1 CEL SULF2 SFN CCNB1 MRPL50 NRIP1 KIF14 PPM1D CRY1 DDB2

STAIB_UP_DOWN_P53_GENES_HYPOXIA_TREATMENT na CYR61 AVPI1 NDUFC2 PLAUR UQCRFS1 ARRDC3 KIAA0562 PRKAG2 SEPX1 HSMPP8 CLSPN MARS HERC3 IL1RAP ETS1 ELL2 BTG1 AASDHPPT GEM CNIH4 NUFIP1 RIOK3 TST PKD2 HMGCL KIAA1729 PURB HSPC157 GDF15 SLC36A1 GADD45B NR4A2 DUSP5 ZNF302 PRG1 LACTB2 CSTF2T PAIP1 PCAF

ARMSTRONG_2002_DOWN_IN_MLL_VS_ALL na EDEM1 MONDOA 3.8-1 MAN1A1 HLA-DQB1 ATP6V1G2 SCHIP1 PIK3C2B DNTT CD24 RNF167 SPTBN1 MYO1B LOC55565 TRIM38 CD84 ITGA6 HBS1L GNAI1 ZNF254 TCF3 SPTAN1 MGC12518 LOC54103 NPR1 LIG4 ZNF273 TCF7L2 TERF2 NEDD4 WWP1 NT5E AKAP12 SMARCA4 ALOX5 ERG TM4SF2 TNFRSF7 ZNF268 KIAA0082 DBN1 HNRPH3 C1ORF16 PRKCB1 GAB1 E2F5 CCND2 DYRK3 PRKCH MYLK ZNF263 WFS1 FOXO1A ITPR1 ZNF45 GNA11 HLA-DMA ECM1 APP HLA-DMB POU2AF1 LDOC1 DDR1 CD22 ZNF423 LARGE SPTA1 ITPR3 FGFR1 IFI16

ARMSTRONG_UP_IN_MLL_VS_ALL na FLNA BZRP THRAP2 PROM1 ATP2B3 MAP2K6 ABR ANXA2 PTPRC BASP1 SERPINB1 CCNH CCL2 EGR1 IGFBP7 AHR HOXA5 MT1B CIAS1 MAP7 WIT-1 MBNL1 GRB2 LILRA1 TBC1D8 ARL4 CYB561D2 MT1G CSPG4 ANXA1 MGC5395 QPRT ANXA2P1 CRABP2 EMP3 CD44 MLC1 LMO2 CCNA1 ADAM10 KLRK1 GLUD1 SCGF TAGLN2 BCAT1 TES CSPG2 TETRAN LMNA RNASE2 LGALS1 LILRB1 ITPA ABTB2 RNASE3 KCNAB2 BTK PTX3 KIAA0992 HOXA9 PLXNC1 PER1 DAD1 EBI2 FLT3

ARMSTRONG_COMBINED_MLL_VS_ALL na MONDOA FLNA MAN1A1 BZRP HLA-DQB1 THRAP2 ATP6V1G2 ATP2B3 ABR PTPRC SERPINB1 CCNH PIK3C2B DNTT CCL2 EGR1 IGFBP7 AHR RNF167 CIAS1 MT1B ITGA6 CD84 GNAI1 GRB2 TBC1D8 LILRA1 MGC12518 ARL4 LIG4 ZNF273 TERF2 NEDD4 ANXA1 ERG QPRT TM4SF2 ANXA2P1 ZNF268 KIAA0082 CRABP2 EMP3 CD44 PRKCB1 GAB1 CCND2 CCNA1 PRKCH MYLK KLRK1 TAGLN2 BCAT1 TES TETRAN CSPG2 LILRB1 LGALS1 ITPA ABTB2 KCNAB2 ECM1 APP PTX3 KIAA0992 HLA-DMB LDOC1 DDR1 DAD1 IFI16 EBI2 EDEM1 3.8-1 PROM1 SCHIP1 MAP2K6 ANXA2 BASP1 CD24 HOXA5 SPTBN1 MYO1B LOC55565 TRIM38 HBS1L MAP7 ZNF254 TCF3 WIT-1 SPTAN1 MBNL1 LOC54103 NPR1 TCF7L2 CYB561D2 MT1G WWP1 NT5E AKAP12 SMARCA4 ALOX5 CSPG4 MGC5395 TNFRSF7 DBN1 HNRPH3 C1ORF16 E2F5 MLC1 LMO2 DYRK3 ADAM10 ZNF263 GLUD1 WFS1 SCGF LMNA FOXO1A RNASE2 ITPR1 GNA11 ZNF45 HLA-DMA RNASE3 BTK POU2AF1 HOXA9 PER1 PLXNC1 CD22 ZNF423 LARGE SPTA1 ITPR3 FGFR1 FLT3

ZEMBUTSU_5FU na FARP1 TTC7A CTDSP2 BZRP GCNT3 FAM38A PRPS1 ATF2 ATP11C MAGEA6 HGFAC TMEM9B PIK3CB RPS6KB1

ZEMBUTSU_CISPLATIN na PCDH1 LLGL2 HMGCL HLA-B FLJ22833 TRPC4 ATG4C HSD17B2 LOC90139 NPR3 PGLYRP1 ABP1 ANXA4 TNFRSF14 FBP1 DES

ZEMBUTSU_ACNU na PABPC1 CKS2 CRKL FLJ21865 PRSS8 LAF4 TOB2 GDA EXTL3 MMP7 SIM1 PGLYRP1 ETV4 KIF4A

ZEMBUTSU_ADR na C1QTNF6 MSLN MORF4L2 CCNB1 PARP9 GPC3 EPS8L2 FSTL3 DES EVA1 DNMT3B

ZEMBUTSU_CPM na ELF3 LSM10 DIRAS1 NRP1 ATP1B1 CPX AGR2 GPX2 TDRD7 SEPT11 DLK1 KCNH2

ZEMBUTSU_MMC na MMP3 PCDH1 SHC2 TRAF2 C20orf174 OTC LOC51315 C6ORF75 C20ORF174 ARHE CPX IL32 PGLYRP1 TNFRSF14 S100A4 COX6A2

ZEMBUTSU_MTX na COL3A1 MYBL2 KIAA0971 KRTHB6 WDTC1 U1SNRNPBP ST7 POLA2 PDK3 GABRA6 TRH

ZEMBUTSU_VCR na OKL38 STC1 GCNT3 VGLL1 NALP2 BARD1 ATP1B1 CPT1A MMP7 NNAT ANXA4 ABLIM1 ETS1

ZEMBUTSU_VLB na UCP3 HSD17B2 PABPC1 RNASET2 PDIA4 CUTL1 CKS2 MYH14 INADL SPTBN1 SLC12A2 GJB1

ZEMBUTSU_MULTIPLE_RESISTANCE na UGT1A1 PDIA4 ANKRD12 GJB1 SYNGR2 MAP3K8 RNASET2 LOC90139 MVP MYH14 USH1C CD74 ACSL5 PCDH1 FCGRT ARSE LAMB3 LLGL2 CYP3A5 BF C11ORF9 TMEPAI BUB1B ATP1B1 SERPINA1 IL32 CCNB1 ABP1 TNFRSF14 LGALS4 ABLIM1

RICKARDSON_GENERAL_RESISTANCE na CLTC THBS3 ROBO1 MAPK11 NEFL ABCB6 CASP3 SLC39A1 AGRN PLXNB1 NAB2 MAGEF1 BCKDK MNDA MYO10 PTK2 PLXNA3 FAM3C CKAP4 SERPINH1 ADAM9 ABCC5 PLXNB2 CORO1C BMPR1A PKN3 ECT2 TRA1 NR2F6 CTTN ITGAV CREBBP CRIP2 MAFG BCAR1 PTK9 DDAH1 PME-1 RIPK2 PRAME CBR1 CSPG2 RRAGA FALZ DVL3 MYL6 LRP4 APP STAT1 LHFP CXX1 RAPGEF2 RB1 S100A10 NBL1 PEA15 IGSF4 MDK MLC1SA RDX SCHIP1 CASP6 BASP1 GPR161 TTF2 CAST TULP3 CCL8 LTBP1 MYO1B ZA20D2 FAT PDLIM7 GLRB EPHA2 SNAPC1 SPAG9 DEGS1 CFL2 MAP4K3 HIF1A AKAP12 DAG1 FARP1 DRPLA EPHB3 JUN PAWR SDCBP PTTG1IP FAIM2 ITSN1 PTPN13 SLC11A2 PPAP2C BACE2 PLS3 TEAD4 FXYD1 NFKBIE TPI1 BAG3 CD9 MAPK1 ITPR3 SGCE

RICKARDSON_GENERAL_SENSITIVITY na TEX264 HCLS1 GNA15 HPRP8BP ATPIF1 MTHFD2 GAS7 PSD4 TPBG NOL5A NCF4 RCD-8 MAP4K1 PPIH RAB18 MCM5 FBL CDX2 COX17 POLD1 DOCK2 HADHSC MYCBP2 MYB NUDC GNL2 EDG6 SUCLG1 COX5B OSTF1 CD37 LCP1 HNRPDL RPIA NIFIE14 TFR2 RAC2 STAT5A INPP5D PTPN7 BNIP2 ARHGDIB SLC25A5 LCP2 IL2RG SLC1A5 HDAC1 BCCIP IDH3A TMED3 SLC7A5 CCNC NFYC PHF2 PPP1R8 TIMM10 IFRD2 EBNA1BP2 CD4 WAS GMFG IDH3B RNUT1 POLQ DUSP7 GCAT CCND3 PENK KIAA1545 IMPDH2

RICKARDSON_DOX_RESISTANCE na HLA-DOA RAC2 DRPLA BNIP2 GAS7 BCAR1 ARHGDIB LCP2 TPBG PSD4 DDAH1 SURB7 MAPK11 PTTG1IP TTF2 ASCC3 CAST TULP3 ABCB6 IDH3A TMED3 AGRN SLC39A1 GNA11 PHF2 EBP SPAG9 EXTL1 FXYD1 TIMM10 TPI1 WAS RNUT1 STAT1 POLQ NIPBL GCAT CTTN

WILLENBROCK_IMMUNO_PREDICTION na CD19 CTBP2 IFNA2 PPP3CC NUBP1 LILRA2 SNX2 INSR RGS10 TOP2B PIK3CD CD24 STK32B CD3D CD6 HLA-DRB1 MSRA TSPAN13 NUCB2 MEF2C RAI14 JUP LILRB2 /// LILRB3 EGLN1 SCMH1 CXCR4 HLA-DMB TIAF1 /// MYO18A

WILLENBROCK_OUTCOME_PREDICTION na ZNF202 ZW10 MYB HSA277841 FADD LILRA6 GGPS1 CNR2 HMGCL APPBP2 MAPK4 GPR89 UBL4 PTPN9 MRPS35 ATF1 COG2 ZNF146

BEESLEY_BPAIR_RELAPSE200 na SDF4 SOX4 SPTLC2 TMOD1 APLP2 UGCGL1 GIPC1 CFLAR YWHAH RNF123 RNF167 MLLT10 C14ORF111 DDOST FUS GPR107 DDX17 ZNF330 SOD2 LPHN1 CDK2 NUMA1 FBXW4 KLHL7 TFDP2 HNRPU TP53 C14ORF139 PI4KII SMCX MPO ARMC9 CES4 P4HB ARS2 DLGAP4 COPA DC13 WDR1 GANAB U2AF2 HGS WHSC1 YKT6 HIPK2 ABHD5 NCOA2 TTLL5 POLR2C HAND2 NFE2L1 LASS4 PBX2 ZNF528 CREBL1 /// TNXB C1QTNF3 CD47 FALZ TSC1 SCARB2 GCLC ANK1 KIAA0792 BAZ2A CRKL PPIG HBLD2 POLR2A WAS EIF4G1 JAK1 RPN1 PTBP1 MED18 ARF4 DYRK1A LOC348162 ARG1 GALNT4 CREBL1 AKAP8L EDA FAM46C ATP13A1 ATP6V0A1 PPIF YEATS4 HIRA RAB7 SFRS2IP MACF1 ANKHD1 DALRD3 OPHN1 LRP8 PSMF1 TAF4 VPS37A LAMP1 PVR DHX9 TRAM2 SPTBN1 CGB /// CGB5 /// CGB TOLLIP ZNF444 PCDH1 SLC25A28 CBLB NAPA TMED2 ANAPC5 POLR2D HMGB1 PIP5K1A ADAM28 G22P1 ARPP-19 SERPINA10 C16ORF35 RBM8A MYST4 ANP32E PRKCSH STEAP3 DDX11 FECH PRO1843 C6ORF106 ALG3 JMJD2B CFDP1 PTPN11 CXORF34 C5 SAFB TRIM2 TRRAP FLJ11710 SSR3 ZC3H7B CGGBP1 FLJ10246 MFN2 DKFZP761I2123 CAPZA2 HNRPH1 LEPR LTF PDIA3 RAB5C CACNA1G LOC23117 /// DKFZP54 C5ORF13 SH3MD1 EWSR1 HBBP1 RIOK3 PES1 BSG GSR NUP50 HNRPUL2 GTF2F1 AP1S1 SYT12 GLUD2 PIP5K2A KIAA0265 RAB5A ZNF451 SFRS5 PAX5 MORF4L1 /// MORF4 TAF15 ACTB GTPBP1

BEESLEY_TPAIR_RELAPSE200 na GNL3L MOBK1B TACC2 MGC2889 KCNK4 GJB3 COL14A1 PTPRC BPY2 /// BPY2B /// B PRLR GRLF1 HTR3A UGCGL1 FAM12A PP2447 FAM61A RNF125 SLCO2A1 C14ORF111 SCAND2 FUS C14ORF92 /// LOC2854 PGS1 NUMA1 HSHUR7SEQ CYSLTR2 CD37 HNRPU SP110 RHOH SPN MAGEA12 EPB41 SMCX FMOD ZNF652 KCNMB1 GALGT CD160 AD7C-NTP COPA CDH18 NONO SLPI GAB1 SKIV2L C6ORF69 WDR1 PRKAR1A COL8A1 WHSC1 C14ORF92 HTR1D POLR2C EPRS STAT2 PBX2 CNTNAP1 FLJ11506 RABL2B /// RABL2A RARB COLEC11 KIF2 ARHGAP28 GK ZIC3 GDI2 GLE1L C2ORF18 GTF2I CNNM1 TIAM2 DYRK1A MOGAT2 TRPM3 CREBL1 AGMAT KRT5 SLC8A2 FLJ35348 NOX1 YES1 SET /// LOC389168 OPHN1 GGA2 LOC56902 OR5V1 /// OR12D3 MCM3AP MANSC1 THEA LRP2BP OBSCN BTBD9 LAMP1 ASH2L PRKCE DSCR3 STX16 IL2RA ETS1 GAD2 TCF3 COL6A2 PBXIP1 DNAH3 ANAPC5 PIP5K1A LMO7 IGF1 RBM8A NF1 TP73L CAPN6 MCL1 LACTB2 PML NOS1 IFNA10 TRIB2 C6 BC37295_3 TAT ROD1 SLC25A17 PTPN11 CXORF34 SAFB SKIV2L2 KIAA1579 ESR2 SSR3 PIGO ZC3H7B SLC25A6 PDPN TCN1 CSF1 PPFIBP1 BAX CLCA2 MFAP3L PDIA3 STAT3 MGC4308 RAB5C C12ORF8 OAZ2 KRT6A /// KRT6B /// TNCRNA ZFHX4 RBBP4 TMEM8 SLC17A4 APH1A C6ORF62 CAMK2A ARL6IP2 RASGRP2 GTF2F1 MEFV ACLY ZGPAT RGR ASMTL CLN8 PI3 CSNK1A1 SFRS5 SFN LARGE AP1M2 SMU1 RGNEF TAF15 RNF39 PCNXL2

CHIARETTI_ADULT_ALL1_AF4 na ITGAE RHOBTB3 FUT4 THRAP2 PROM1 WT1 MEIS1 VLDLR TRIM9 IMP-3 IGFBP7 ADCY9 HOXA5 TPD52L2 KIAA0125 GPM6B PPM1H PPP2R5C DIAPH2 TBC1D8 DPYSL3 MAP3K5 SPN CSRP2 QPRT BID DSTN PIK3C3 SPINK2 CD44 RUNX1 CCNA1 NPTX2 KLRK1 HOXA10 SLC1A4 LGALS1 RNASE3 CD72 KIAA1043 PRSS12 KIAA0992 DKFZP434H132 HOXA9 PKIG RALBP1 DAD1 FLT3 EBI2

CHIARETTI_ADULT_E2A_PBX na SLAMF1 CSF2RB DBN1 FGF9 E2F5 ARL7 PBX1 KIAA0802 KIAA0889 AOX1 NCBP2 GP5 PRKCZ TNK2 TMSL8 FAT LAMA5 PKM2 MERTK ALDH1A1 BLK ANKRD15 CRYM CYFIP1 SCCPDH TCF4 LRMP TRIB2 NID2

CHIARETTI_ADULT_BCR_ABL na HLA-DOA HLA-DQB1 YES1 SCHIP1 FHL1 HLA-A AHNAK ITGA5 CD24 LST1 MME WSB2 ENG HLA-B /// HLA-C CD99 MYO1B ITGA6 CD74 MTSS1 ABL1 HLA-DRA GNAI1 ID3 UGCG SMAD1 PDE4B IFITM1 TNFAIP2 TLE4 HIF1A PSAP CDC42EP3 NT5E SOCS2 ANXA1 IFITM1 /// IFITM3 // LSP1 HLA-DRB1 /// HLA-DRB TSPAN7 FYN DUSP6 HLA-DPB1 HLA-DPA1 KLF9 GAB1 LCP2 ANKRD17 CCND2 ACTN1 PRKCH CASP8 FZD6 UBE2E3 TUBA1 SLC2A5 IGJ ITPR1 OPTN CD52 ENPP2 TXNIP MARCKS XPNPEP1 DDR1 NINJ1 RGL1 ACVR2A NRIP1 CD2AP SEMA6A

CHIARETTI_ADULT_ALL_RELAPSE_PREDICTION_PARTIAL_LIST na IQGAP1 SDFR1 PIP5K2B PHKB PRKCB1 HIST1H2BD ATR HIST1H2AC PIK3C2B CAPZB ITGB1 LGALS8 ATRX CD79A MVP TLN1 DNCI2 CD24 DAAM1 LSP1 ADD3 ASNS ITPR3 CD79B

YEOH_CHI_BCR_ABL na ATM TNFRSF7 PON2 SPARC CCND2 BTG2 CRADD ACTN1 CAPZB CASP8 ENG TRAM2 SAS10 TUBA1 SLC2A5 IGJ LGMN CASP10 GPAA1 ABL1 CNN3 STARD7 BST2 ECM1 TSC22D4 IL10RB GYPC AKR1C3 DGKD CTDSPL NPC1 DPYD AGPS CLEC2B SEPT11

YEOH_CHI_E2A_PBX1 na SLAMF1 CSF2RB KIAA0922 FLI1 CD58 PBX1 HCAP-D3 IRF4 MYBPH NP SLC27A2 KIAA0802 MME SYNPO PRKCZ CIRBP RAG2 HIP1R FAT ELOVL5 BUB3 KIAA0247 MERTK GOLGA3 BLK C1ORF121 ANKRD15 SNRPE PARP1 RNPEP SOCS2 MAPKAPK2 LRMP ADARB1 TRIB2 NID2

YEOH_CHI_HYPERDIP50 na PGK1 MPP1 TCEAL4 TMED10 HNRPH2 UBE2A CALM1 NPY SH3BP5 SYBL1 PSMC1 POU4F1 NDUFA1 KIAA0179 PSMD10 CSTB SCML2 VBP1 DKC1 DXS9879E HPRT1 HMGN1 IL1B BCAP31 CYB5 PRPS1 SLC9A6 MX1 HUWE1 ATP6AP2 PRPS2 MED12 TCEAL1 MTCP1 EIF3S12 FYN SUMO3

YEOH_CHI_MLL na RHOBTB3 PCDHGC3 /// PCDHGB4 CD44 MPP1 MEIS1 IGKC /// IGKV1-5 PTPRC DNTT ADAM10 KLRK1 IGFBP7 LY75 MME GLUL TES LGALS1 GUCY1A3 FKBP5 RASA1 ANXA2 /// ANXA2P1 // DNAJB6 WDR18 BLK GOLGA3 ELF1 PTPN6 C1ORF121 HOXA9 SAP18 PLXNC1 TIAF1 /// MYO18A ACAA1 S100A10 XBP1 DAD1 CAPG

YEOH_CHI_NOVEL na RAC2 PCDH9 BCL7A PLTP CLTC LHFPL2 PDGFRA LIMK2 /// PPP1R14BP1 PTPRM EHBP1 AFF1 SMAD7 C11ORF8 LGR5 ITGA6 CHST7 IQGAP1 RXRA TRAF3IP2 LSS PTGDR SATB2 CENTG2 TLE4 GLDC CD34 CNR1 CTNS FCGR2A MEF2A APOBEC3G LCP1

YEOH_CHI_TALL na CD3E CD19 TRBV21-1 /// TRBV5-4 HLA-DPA1 HLA-DPB1 LCK NCF4 TCL1A IGHM TOP2B CD24 TRBV19 /// TRBC1 BLNK PRKCQ CD79B FOXO1A CD74 SH2D1A TRBV21-1 /// TRBV19 HLA-DRA NUCB2 HLA-DMA PTP4A2 HLA-F TERF2 USP20 MAL AKR1C3 PLCG2 CD79A ITM2A HLA-DRB1 /// HLA-DRB SEPW1 TCF7

YEOH_CHI_TEL_AML1 na TNFRSF7 TCFL5 VGLL4 PIK3C3 IDI1 CLIC5 PRKCB1 CRMP1 RAG1 PCLO FKBP1A NME2 GNG11 SCARB1 TSPYL5 TXNRD1 KCNN1 FCHSD2 FYB PTP4A3 ITPR1 FUCA1 PDLIM1 MYO10 TRAF5 SMAD1 ANXA2 /// ANXA2P1 // ARHGEF4 TERF2 CBFA2T3 POU2AF1 SMARCA4 KHDRBS3 PTPRK ITPR3

YEOH_CFS_BCR_ABL na ATM TNFRSF7 LAIR1 IDI1 SCHIP1 LCP2 CRADD ACTN1 TNFRSF1B ITGA4 LSM6 PAK1 FSCN1 CASP8 TPD52L2 ENG TUBA1 TRAM2 SAS10 CASP10 PICALM LGMN GPAA1 MAP1LC3B ABL1 TRAF5 BZW1 /// LOC151579 TCF3 SHOC2 STARD7 BST2 WASF1 RAB6IP1 TSC22D4 TNFSF4 PECAM1 C3F JAK1 ADCY3 AKR1C3 DGKD DR1 CTDSPL SOCS2 NPC1 AES CLEC2B SEPT11

YEOH_CFS_HYPERDIP50 na TUFM PROM1 EVI2B RAP1B MORC3 PRIM2A RPS6KA3 ATP5J RAG1 SF3A1 TMED10 CALM1 DKFZP586A0522 ACTL6A CEBPD SNRP70 NP NCBP2 PSMD10 ADRM1 CDC16 COX5A SCML2 STK25 UBE1L UGDH SREBF1 FNDC3A CYB5 PPM1A STAT6 PPM1G C18ORF10 AFG3L2 ZHX2 AKAP12 LGALS9 CEP4 C20ORF47 TIAF1 /// MYO18A MTCP1 EIF3S12 SYK CEP1 FYN RBM5 COMT PCDH9 MDM1 SATB1 RUNX1 GNG7 UCKL1 CAPZB UBR2 HSF1 HDAC1 POU4F1 PSMC1 ZNF91 SNRPA ETS2 RYBP UBE2G2 /// TAX1BP3 SELL TKT PMP2 CASP4 CAPN3 BTAF1 ASF1A CHD9 NOL1 MARCKS STAG2 PDE8A BCAP31 LPXN MGC10471 SMS /// LOC442230 MX1 MRCL3 PFDN5 HUWE1 RB1 EIF3S2 GBE1 FOS SON SUMO3

YEOH_CFS_MLL na HCLS1 RHOBTB3 CXORF9 CUGBP1 MEIS1 MGAT1 BTG2 BASP1 PTPRC SERPINB1 DNTT SLC39A8 LY75 IGFBP7 CCT5 SMAD2 MME BLNK MBD4 MYC IQGAP1 SYPL1 GPSM3 SMAD1 STAT6 LIG4 SPAG6 HIF1A BLK ELF1 PSD3 SMAD3 PPID MEF2A TLN1 UBE2D2 DAXX TNFRSF14 POP5 NAP1L1 UBE2J1 RAC2 ANP32B DDEF2 TLOC1 ATP2B4 TGIF FTH1 BTN3A2 SLC25A6 PHF15 GUK1 LRIG1 ADAM10 KLRK1 MTF2 POU4F1 ZNF91 CALU GLUL TES LGALS1 FKBP5 CD52 CDKN1B CHD9 PBX3 TXNL1 RAB9A ARPC2 PPP1CA IGHA1 /// IGHA2 /// C1ORF121 HOXA9 DDR1 CD9 PLXNC1 PCBP1 FLJ12443 DBI EIF3S2 UBE1C LIMK2 ACAA1 S100A10 XBP1 ODC1 CD2AP CAPG

YEOH_CFS_TELAML1 na NUP210 CASP1 HLA-DQB1 ATF2 AMD1 ISG20 IRF4 CDK9 NBEAL2 MYC CALM3 KARS GPR18 FADS3 PDLIM1 PIP5K3 AP1B1 TERF2 SPCS2 IRAK1BP1 LTB B4GALT1 IQSEC1 C7ORF28A /// C7ORF28 UBE2J1 C6ORF80 DARS VAV1 BLCAP CLSTN1 MYST3 PRKCB1 PCLO RBBP8 ACTN1 EPRS MGC40157 PSME2 FCHSD2 PTP4A3 STARD7 ECM1 CDK2AP2 JAK1 HLA-DMB SPOCK2 CLEC2B MDK EIF2S1 TCFL5 USP9X SMARCA2 IDI1 TOMM7 /// LOC201725 ADIPOR2 CHN2 RERE FRMD4B TMED10 ITGA4 CBX3 PLP2 SCARB1 SORD LAMP1 DKFZP434C171 PRKDC S100A13 SMAD1 WASF1 JARID2 DDIT4 PNRC1 SDHD RPS6KA1 UBL3 ATXN2L TNFRSF7 DBN1 ARHGAP25 HLA-DPB1 PTPN2 RUNX1 SH3BP5 PSCD1 TSPYL5 CD164 IQGAP2 LGR5 NFE2L2 DSIPI SERPINF1 ITPR1 CD52 HLA-DMA GLUD2 CBFA2T3 POU2AF1 PPP1CA SPTA1

YEOH_TSTAT_BCRABL na FOXJ2 RUTBC1 LRPAP1 ERCC5 TNFRSF1A NOL5A CRADD GPSN2 PHYH ADAM10 ZFPL1 RANBP2 LSM6 LY75 PAK1 PTPN9 FSCN1 CASP8 HCK WSB2 ISLR TUBA1 EP300 MXRA7 MPHOSPH9 PPP1R12B HSF2 CHERP RRM1 KIAA0692 PPP2R5D PECAM1 MGP SLC30A1 SH3GLB1 NPC1 CEP1 PTEN

YEOH_TSTAT_E2APBX1 na CSF2RB SCHIP1 DAPK1 AEBP1 TNFRSF1A PTPN2 PBX1 IRF4 NP CBX1 KIAA0802 IGFBP7 FSCN1 SYNPO HIP1R GNAQ FAT CYBB IL1B KIAA0247 PECAM1 CSF3R IFI44 BLK ALOX5AP C1ORF121 PARP1 ALOX5 SOCS2 AIF1 SCCPDH CDKN1A XBP1 ODC1 TRIB2

YEOH_TSTAT_HYPERDIP50 na CGI-96 PCDH9 TUFM PROM1 PGK1 AEBP1 TCEAL4 EFNB1 ZBED1 CALM1 NPY TSC22D3 SH3BP5 SNRP70 POU4F1 NDUFA1 MAZ RAG2 ARHGEF6 EIF3S7 BTG1 KARS PSMB1 HMGN1 BCAP31 IL1B FNDC3A MX1 PFDN5 PSAP ATRX MED12 FOS PTPRK EIF3S12 ODC1 RBM5

YEOH_TSTAT_MLL na C10ORF10 XRCC1 SCHIP1 IGFBP4 SIN3B ATP2B4 CCND2 PTPRC EFNB1 MRC1 /// MRC1L1 SLC25A6 TIAL1 CEACAM6 IGFBP7 POU4F1 MME DPEP1 HBS1L GNA11 LGALS1 SMAD1 IL1B PLCL3 PECAM1 ARPC2 LDOC1 C1ORF121 PTPN6 DDR1 SV2A PTPRK ITSN2 FHIT

YEOH_TSTAT_NOVEL na STXBP2 BCL6 SLC9A3R1 MS4A1 ADCY7 CLTC RGS9 ICAM3 IBRDC3 PTPRM GNG11 MKL1 CENPC1 SLC1A4 UBE2E3 TUBA1 DDX10 ITGA6 ETEA SMAD1 FUT7 MXRA7 MEF2C ECM1 HIST1H2BD TNFSF4 ZYX PECAM1 CSRP1 CENTG2 LOC92482 MPO EIF4G3 CHST2 TSPAN7

YEOH_TSTAT_TALL na PTPN18 CD3E CD19 SLC9A3R1 C10ORF10 HLA-DQB1 MAG SCHIP1 GAB1 HLX1 GNG7 TCL1A NPY LILRA2 GNG11 TOP2B CD24 CD3D ENG PFTK1 CD79B SH2D1A TRBV21-1 /// TRBV19 CD22 /// MAG CTGF MEF2C PDE4B DENND3 LOC54103 GALNAC4S-6ST MAL PSD3 CD79A ITM2A RGL1 CDKN1A CTNNA1

YEOH_TSTAT_TELAML1 na TCFL5 ARHGEF18 PIK3C3 CLIC5 PRKCB1 CRMP1 RAG1 PCLO ACTN1 NME2 PHYH GNG11 SORD TSPYL5 ZNF91 HLA-DOB KCNN1 FOXO1A PTP4A3 FYB MYO10 SMAD1 STARD7 ARHGEF4 TERF2 MRCL3 CBFA2T3 LTB PTPRK SPTA1 ITPR3 MDK SEMA6A

YEOH_WILKINS_BCRABL na ATM FOXJ2 OLFML2A PARG RUTBC1 LRPAP1 ERCC5 SFRS1 PTGER2 DHRS3 MANBA RUNX1 CCND2 CRADD GPSN2 ADAM10 ZFPL1 RANBP2 INSR LSM6 LY75 PAK1 ZFHX1B FSCN1 CASP8 WSB2 TUBA1 ISLR CASP10 BZRAP1 ITGA6 CNN3 MXRA7 MPHOSPH9 C6ORF62 CD72 WASF1 CHERP RRM1 PRDX2 PECAM1 SLC30A1 AKR1C3 ST3GAL5 SH3GLB1 SV2A DPYD PTEN

YEOH_WILKINS_E2APBX1 na SLAMF1 KIAA0882 SORBS3 SCHIP1 TNFRSF1A C1ORF38 GAB1 ZMYND11 PTPN2 CCND2 PBX1 IRF4 DKFZP586A0522 MYBPH KIAA0802 SLC27A2 FSCN1 LST1 PRKCZ TUBA1 ETS2 ITGA6 TNFRSF10B HIP1R ITPR1 FAT FAM38A MERTK STIM1 IFI44 CD34 BLK ALOX5AP ANKRD15 HA-1 ALOX5 LTB SOCS2 AIF1 FNBP1 ADARB1 TSPAN7 TGFB1 TRIB2 FLT3 NID2

YEOH_WILKINS_HYPERDIP50 na CGI-96 SCHIP1 PROM1 FLI1 AEBP1 TCEAL4 TMED10 HNRPH2 EFNB1 PHB UBE2A UCKL1 ANXA5 NPY HSF1 SH3BP5 POU4F1 RAG2 SCML2 ITPKB USP4 VBP1 CAPN3 DXS9879E SMAD1 IL1B CYB5 FNDC3A PRPS1 PLCL3 MX1 ATP6AP2 NEK9 PRPS2 HDHD1A TCEAL1 IFIT1 ILF3 MTCP1 FOS PTPRK TCF4 SYK TSPAN7 FYN SUMO3

YEOH_WILKINS_MLL na POLE C10ORF10 SCHIP1 MPP1 ATP2B4 CCND2 FRMD4B PTPRC BASP1 TNFRSF1B ACTN1 DNTT MRC1 /// MRC1L1 PRKCH SH3BP5 PHF15 POU4F1 IGFBP7 LST1 MME LGALS1 ID3 S100A13 SMAD1 PDE4B IL1B LIG4 PECAM1 PLXND1 VAMP5 TPST2 OAS2 BLK C1ORF121 ALOX5 PLXNC1 LTB LOC57228 LARGE TNFRSF14 ERG S100A10 TSPAN7 CD2AP MDK

YEOH_WILKINS_NOVEL na ADCY7 CLTC LHFPL2 IFI30 IGFBP4 FHL1 EHBP1 GNG7 PTPRM PHYH GNG11 PLP2 LY86 C1ORF2 LST1 C11ORF8 WSB2 TUBA1 ITGA6 UNC84A PPM1H MXRA7 SMAD1 MEF2C CRIP1 FAM38A DENND3 VCL RAB6IP1 HIST1H2BD CD48 GALNAC4S-6ST CENTG2 COX7B PGRMC1 ITM2A OSBPL8 MAP2K2 LTB MPO EIF4G3 PRG1 ARCN1 CHST2 TSPAN7

YEOH_WILKINS_TALL na CD3E LAT CD19 CHI3L2 CTBP2 MAG HLA-DQB1 HLA-DPA1 HLA-DRB4 NCF4 TCL1A IGHM NPY LILRA2 CDK9 INSR CD24 CD3D ENG PFTK1 CD79B FOXO1A SH2D1A FADS3 CD22 /// MAG PDLIM1 CTGF HLA-DMA MEF2C JUP IL1B PDE4B DENND3 BTK GALNAC4S-6ST MAL POU2AF1 PLCG2 ITM2A CD79A CD9 SOCS2 CTNNA1

YEOH_WILKINS_TELAML1 na TNFRSF7 TCFL5 CASP1 TXNDC PIK3C3 CLIC5 CD44 IGFBP4 CRMP1 GPX7 RAG1 ACTN1 PHYH GNG11 SCARB1 SORD TSPYL5 HLA-DOB IQGAP2 FLNB C10ORF26 MYC KCNN1 FCHSD2 PTP4A3 FYB FUCA1 NDRG1 PDLIM1 MYO10 SMAD1 SDC1 ARHGEF4 TERF2 IRF8 DDIT4 CD9 PRPS2 KHDRBS3 PTPRK AP1S2 ITGB2 LSP1 ITPR3

YEOH_DAV_BCRABL na ABL1 OLFML2A S100A13 PON2 COL6A3 IGFBP4 ECM1 CAP2 CDC42EP3 PHF15 GADD45A CTDSPL TRAM2 TUBA1 SLC2A5 CASP10 IL2RA

YEOH_DAV_E2APBX1 na KIAA0922 FLI1 PBX1 HCAP-D3 MAGED1 NP MYBPH KIAA0802 SLC27A2 PRKCZ HIP1R FAT BUB3 IL1B KIAA0247 CD72 AMOT MERTK BLK C1ORF121 ANKRD15 AKR1B1 PARP1 CRYM MAPKAPK2 NFKB1 NID2

YEOH_DAV_HYPERDIP50 na UBE2J1 USP9X MPP1 TCEAL4 ZBED1 ACTN1 SH3BP5 SOD1 PLP2 DAAM1 OFD1 KIAA0179 PSMD10 BLNK ARHGEF6 UBE2G2 /// TAX1BP3 SCML2 PDLIM1 HMGN1 RAB9A IL10RB ATP6AP2 CD9 SUMO3 FLT3

YEOH_DAV_MLL na RHOBTB3 PCDHGC3 /// PCDHGB4 TUFM THRAP2 EIF4B SLC25A3 CD44 NR3C1 PABPC4 MEIS1 CCNA1 ADAM10 KLRK1 IGFBP7 HOXA10 TPD52L2 HOXA5 EIF3S7 LGALS1 CAPN3 MBNL1 MAP3K5 PLXNC1 C20ORF47 MEF2A TNFRSF14 DAD1 CAPG

YEOH_DAV_NOVEL na MONDOA PCDH9 SCHIP1 LTA4H CDH11 LIMK2 /// PPP1R14BP1 CCND2 PTPRM DNTT DKFZP586A0522 PRDX1 TOP2B FSCN1 MME ENG LGR5 CD99 LOC90925 DDHD2 RNPC1 CTDSP2 GPR18 TCF3 SATB2 LASP1 TLE4 MSN PKIG CHST2 XBP1 QPRT APOBEC3G

YEOH_DAV_TALL na SH2D1A CD3E TRBV21-1 /// TRBV19 HLA-DRA NUCB2 TRBV21-1 /// TRBV5-4 HLA-DPB1 HLA-DPA1 PTP4A2 LCK USP20 MAL ITM2A CD3D SEPW1 PRKCQ TCF7

YEOH_DAV_TELAML1 na EDEM1 TNFRSF7 TCFL5 LAIR1 VAV1 ARHGEF18 CLSTN1 PIK3C3 IDI1 CLIC5 PRKCB1 RAG1 LBA1 FKBP1A SCARB1 INSR ZNF91 HLA-DOB C10ORF26 FCHSD2 KCNN1 PTP4A3 FOXO1A ITPR1 FUCA1 PIM1 CHD9 SMAD1 AP1B1 ARHGEF4 TERF2 CBFA2T3 POU2AF1 SMARCA4 LTB PTPRK SPTA1 MDK

YEOH_RELAPSE_HYPERDIP50 na CD3Z RPS4Y1 RNF11 STAT5B NR2C1 STCH DDX3Y IMPA1 MAN2B2 CHSY1 LOC255458 TAF12 CALCOCO1 TMEM41B HHLA1 HAGH PIP3-E CRY1

YEOH_RELAPSE_OTHERS na ELAC2 MAN1A1 BAHD1 SRP46 THOC1 GPR161 TOPORS STAT1 P29 HIST1H1C CBX1 MVP RASSF7 GTPBP6 KIAA0339 SEPT11 CACNB3 ZNF193

YEOH_RELAPSE_HYPERDIP_PERM na CD3Z RPS4Y1 RNF11 STAT5B NR2C1 STCH DDX3Y IMPA1 MAN2B2 CHSY1 LOC255458 TAF12 CALCOCO1 TMEM41B HHLA1 HAGH PIP3-E CRY1

ROZOVSKAIA_T(4_11)_VS_ALL na UCK2 LMO4 PLTP OBSL1 ATP8A1 MEIS1 EXT1 ENOSF1 PIK3C2B RYK TAF11 C21ORF25 ITGA6 SERPINB6 FLT3LG AIM1 LILRA1 TBC1D8 GNA12 HOXA4 LMNB1 TLE4 BLK ALDH3B2 PROC PTPN6 DHFR LY6H QPRT GLT8D1 RECK TRFP ITGB3BP CD44 GCSH CCND2 SPRY1 CCNA1 KLRK1 SH2D1A PTP4A3 CAT MYL6 GDI2 SC65 KIAA0992 CDC2 ASAHL CTNS S100A10 EVPL ME2 ZNF467 ABLIM1 NXT2 PROM1 YES1 MAD2L1 CLIC4 VLDLR PLOD1 MAC30 IL3RA CECR7 RAD23A TNFAIP2 KIAA0685 CAP2 IPO7 AKAP12 ALOX5 CSPG4 TNFRSF7 TBL1X IGFBP4 IER3 ADAM8 ARL6IP5 RAB4A IGFBP3 KCTD17 SH3BP5 GALC IGJ PPP3CB RPP40 MAST4 CD52 FADD CD69 GADD45G PDCD2 LILRB4 PCNXL2 USP13 POLE RHOBTB3 DAPK1 IMP-3 IGFBP7 PCK2 MME C6ORF32 MYC GPM6B PPM1H GNAI1 TMSL8 PFKP PTPRD CCR7 FEZ1 EEF1E1 LTB TNFRSF14 LSP1 NAP1L1 ANXA2P1 GATA3 DARS JUNB TRA@ /// TRD@ UBXD6 FLT1 POU4F1 SCP2 STK39 PXDN NCOA1 BCAT1 LGALS1 SMARCD2 CDKN1B CD72 KCNAB2 APP PVRL2 SPOCK2 PKIG LRMP FHIT ODC1 MDK FUT4 TRBV21-1 /// TRBV5-4 EVI2B FOLR2 ANXA2 IL17R ANXA5 MYBL2 SEC23A PDGFRB NP ADCY9 GGT1 /// GGTL4 SUPT4H1 PTGER4 HDAC9 HOXA5 CDKN3 PALM2-AKAP2 PPP2R5C CNN3 MAP7 RRS1 SMAD1 DIAPH2 PRDX3 PDE4B SPIB HLA-F LOC54103 GRIK5 DPYSL3 ATP2A3 ATXN10 MAP3K5 SERPINB8 RRM2 BLMH NT5E SDHD FKBP9 CSRP2 NMT2 EVI2A GSTM1 /// GSTM2 /// JUN MAF LMO2 WEE1 GSTA2 STX1A HOXA10 SC4MOL WFS1 MGST3 MX2 DPEP1 PPP5C GNA11 CDKN2C DDX1 BCL11A CDH4 MX1 ANPEP HOXA9 ITM2A LOC57228 LARGE ITPR3

ROZOVSKAIA_T(4_11)_AND_CD10NEG_VS_ALL na YWHAZ PABPC1 HKR3 SMARCA2 CENTB1 CUGBP1 PRPF4B AMD1 PTPRC GTF2I /// GTF2IP1 RARRES3 CALM1 ITGA4 P2RX4 RECQL TOP2B LST1 TOP2A GCHFR TAF11 ACTR2 ZNFN1A1 BIN1 TAF2 PRKDC STK38 WASF1 PRKCBP1 IFITM1 HSPA4 IRF8 KIAA0685 IPO7 GNA13 TFRC DDX3X DNCI2 EIF2S3 ILF3 ERG GNL1 NAP1L1 TNFRSF7 ATP5A1 PITPNB ARFRP1 H41 SCAP2 FLOT1 CD164 MAP2K1 VPS4B UPP1 RSN CCNC SERBP1 PDE8A RBL2 CCS MRE11A STK11 VASP JMJD1C PKP4 GNAS RB1 SFRS3 MAT2A WDR45L FLT3

ROZOVSKAIA_MLL_VS_BOTH_CD10NEG_AND_OTHER_ALL na GATA3 RECK FUT4 DAPK1 CD44 JUN CCND2 MEIS1 VLDLR PLOD1 ARL6IP5 RYK FLT1 GSTA2 POU4F1 HOXA10 WFS1 CECR7 C21ORF25 ITGA6 MYC LGALS1 MAST4 PPP2R5C CDKN2C CNN3 SMAD1 FLT3LG CD72 CD69 TNFAIP2 CDH4 SERPINB8 HOXA9 ALOX5 TNFRSF14 FHIT ITPR3 QPRT LILRB4 PCNXL2

ROZOVSKAIA_COMBINED_ALL_VS_CD10NEG na OGT SLC16A3 CRIP1 PRKCB1 SERPINB9 MAP4K5 RARRES3 ZNF85 SDCCAG1 CD34 SFRS2 THOC2 P2RX4 REL ZNF91 ITGA6 DUSP1 ETFB JMJD2B

ROZOVSKAIA_DOWN_IN_T(4_11) na C10ORF38 ENPP2 ID3 CNN3 PON2 EPB41L3 CPVL KRT18 COL6A3 CHN2 SATB2 ID1 NT5E POU4F1 CD1D LAPTM4B MME HOXB2 LPL MYO1B EFNA1 FGFR1 ABLIM1

ROZOVSKAIA_DOWN_IN_CELL_LINES_VS_TUMOUR na KLF1 ATF3 HIST1H2BM CXORF9 HIST1H2BJ MAP3K8 ZFP36 HIST2H2AA PF4 IGHG1 /// IGHM /// L CDC42 KLHL9 GABARAPL1 MAPK8IP3 HBA1 /// HBA2 GYPB NALP1 IGLC1 HIST1H1C RHAG NFIL3 LSP1 HIST1H2BL HBG1 /// HBG2 TFR2 HBD HIST1H3D JUNB IRF1 KRTHA5 PTDSR SPRY1 TSC22D3 DCBLD2 ZFP36L2 NCOA1 PRKAA1 CXCL12 BTG1 DUSP10 HIST1H2BE EPB42 TAF6L MYL6 MAFF HIST1H2AC STK17B IGHA1 /// IGHA2 /// HIST1H2BK CA2 STK17A HBB MYL4 FSCN2 KLF6 SLC6A8 TMCC2 PLK2 IGKC /// IGKV1-5 RGS1 IGL@ /// IGLC1 /// I PPP1R15A HIST1H2BI HIST1H2AL BPGM HMBS SPTB PDE4B KIAA0247 SELENBP1 HIST2H2BE PNRC1 TAL1 HIST1H2BF SNF1LK DUSP5 IDS IGH@ /// IGHG1 /// I MGC14376 MYT1 RAP1GA1 GYPA NFKBIA FOSB HIST1H2AI IER3 JUN SLC4A1 EPB49 OAT RHCE HIST1H2AE BLVRB DUSP1 HIST1H3H XK SELL SLC2A3 MOBP HIST1H2BN RASGRP2 PRDX2 KCNH2 EIF4A1 PER1 LOC91316 TNFAIP3 RBP3 GMPR KEL ALAS2 FOS SPTA1

ROZOVSKAIA_MLLPOS_AML_VS_OTHER_AML na KIAA0241 PCCA KLF6 ZMPSTE24 CEACAM8 TUBGCP3 ICK ACOX1 LCN2 PF4 DEFA1 /// DEFA3 DDX17 PPP2R5C PCTK3 MLH1 ZNF318 RAD51C LMNB1 SELENBP1 EIF2AK2 MAL SERPINH1 SNX13 PNRC1 EXOSC8 CSTF2T ATM PPBP COL1A1 MTMR6 NPAT UBE3C SLC4A7 JUNB CIT SKIV2L2 HLA-DRB4 EPB49 FLJ10534 TCN1 ACP5 INSR RARS RGS10 KIAA0261 LTF PARP4 BPI RASA1 DDX1 TAF6L COG5 DKFZP564G2022 AP1S1 CD96 DEFA3 ARG1 SON FHIT MYL4

ROZOVSKAIA_AML_MLLPARTIAL_DUP_VS_TRANSLOCATION na BLM C19ORF7 SRP68 KRIT1 HOXB5 PRR3 C4A /// C4B TIMP1 TOP2A TUSC2 MAZ MYC CDC42 MCM4 ARL4 LMNB1 ZHX2 TP53 EEF1E1 C20ORF47 EIF2S3 ILF3 ATBF1 RCC1 NAP1L1 MXD4 CD302 MT ANKRD17 TSC22D3 ACP5 MTF2 RGS10 STAB1 PRKAA1 AP2A2 KIAA0543 EDG5 TES KIF2 ASF1A CCNA2 GBP2 STAT1 CDC2 PROSC PTK2B NFIX CCNB1 ARG1 AK2 BIK CSTA SFPQ SLC16A5 KLF6 SLC29A1 CEACAM8 MMP9 MYBL2 NP ADCY9 RAD23A ZNFN1A1 SMOX BCL2 MPHOSPH9 EDD1 HSPA4 ATXN10 TFRC G1P2 HIST1H4C AIF1 TNFSF12 MFAP1 TFDP1 CIT LOC440118 FSTL3 FLJ10534 WEE1 ZNF263 SEC23IP TIPARP MARCH2 PPP5C HOXB7 TNIP1 PRPF4 EWSR1 MSH6 FADD MRE11A DEFA3 GPR3 FUSIP1 SON WDR45

ROZOVSKAIA_AML_ALL_COMBINED_MLLPOS_VS_NEG na PSMD14 TRBV21-1 /// TRBV5-4 SLC4A7 JUNB TXNDC SFRS1 SKIV2L2 ITCH WBSCR22 ACOX1 CBX1 TXNDC9 FAM20B KIAA0261 CD24 TAF11 WRB HLA-DRB1 PRKDC PPP2R5C CD52 DDX1 SLC39A6 TTF1 COG5 HPS5 HMGCR MLH1 MCM4 PRKCBP1 LMNB1 RAD51C GRIK5 SPCS2 B4GALT4 ANAPC13 IPO7 OASL DDR1 DUSP5 PKP4 EXOSC8 PSMA3 CLASP2 FHIT PREPL USP13

HOFFMAN_HOX11 na MAP3K10 GALNS BLVRA SLC6A8 SLC5A12 SLC15A1 OLFML2B ZBTB20 PMS2L4 RASA3 TSPAN8 INSR TOB1 CDC42EP4 FRMPD1 GNAL HOXB13 HPGD MAGI2 MTMR7 HIST1H2AK L3MBTL NFKB2 CBX7 STX3A AMHR2 RAPGEF4 MS4A2 EPHB2 MAGI1 BCL2 RORA GRIA3 TRPC1 SMARCD3 NR4A3 TRIM25 ABAT ATP2B2 PRKCD PNRC1 DHX34 PCK1 TRO TNPO3 FHIT FYN LILRB4

MEDH_DEX_INDUCED_IN_SENSITIVE_CEM na BCL2L11 RHOBTB3 NFKBIA NR3C1 CD53 GRAP2 BTG2 FHL1 PTPRM DSCR1 SLA DKFZP586A0522 IL7R WFS1 SLC18A2 TUBA1 NBEAL2 SOCS1 C6ORF32 MGC17330 PALM2-AKAP2 RNPC1 BTG1 BIRC3 MAP1A TXNIP AP1G2 JAM3 AIM1 CD69 TGFBR2 DFNA5 PLXND1 CCR4 PTK2B INPP1 PRG1 NFIL3

MEDH_REPRESSED_IN_SENSITIVE_CEM na MGC2574 HRMT1L3 PPP3CB HSU79274 EIF2C2 TYRO3 HES1 ARMC6 RAG1 NOC2L TLE4 FLJ10534 RAD17 ITGA4 CELSR3 LTB PPAT HMGCS1 HSPC111 KIAA0830 MYC

MEDH_COMBINED_DEX_RESPONSE_IN_SENS_CEM na RHOBTB3 ARMC6 GRAP2 RAG1 BTG2 FHL1 PTPRM SLA ITGA4 DKFZP586A0522 CELSR3 NBEAL2 SLC18A2 SOCS1 HSPC111 C6ORF32 KIAA0830 PALM2-AKAP2 MYC RNPC1 MGC2574 HSU79274 BIRC3 HES1 AP1G2 AIM1 TGFBR2 DFNA5 TLE4 PLXND1 NOC2L INPP1 PPAT LTB PRG1 NFIL3 BCL2L11 HRMT1L3 NFKBIA NR3C1 CD53 DSCR1 RAD17 FLJ10534 IL7R WFS1 HMGCS1 TUBA1 MGC17330 BTG1 PPP3CB MAP1A TYRO3 EIF2C2 TXNIP JAM3 CD69 CCR4 PTK2B

TSUTSUMI_MLL_60_GENE_PROFILE na PABPC1 WBP2 SNRPG MDH1 PSMA1 SF3A1 CSK UFD1L TRAM1 KIAA0372 SMARCC2 BAT3 HMGN4 SNX4 UQCRB CUTL1 TAPBP SEP15 TYK2 C1D DPM1 NDUFA5 ARHGAP1 ATP6V0C TLN1 POP4 ARHGDIA POLR2K TGFB1 INDO ATXN2L RAC2 TRIP3 COPA PSMA4 UNC84B COX6C RNF187 GMFB ZFP36L2 NDUFS4 CHD3 CALM2 DVL3 CEBPZ C14ORF2 DULLARD USF2 SART1 CFL1 ZYX C9ORF16 SIPA1 PSMA2 DNM2 HAT1 HDCMA18P PFDN4 GBAS

YOCUM_PROTEINS_IN_MLL_CELL_LINES_VS_CD34 na FUBP1 SFPQ GSTP1 HNRPA1 STIP1 NME1 PGAM1 ANXA2 ENO1 EIF4E HSPA9B HNRPH1 HSPA1A GLUD1 HSPA5 COPS4 DDX17 KHSRP PKM2 TCP1 ACTG1 GRB2 TUBB HNRPA0 HNRPK DDX5 STMN1 MAPK1 EEF1B2 EEF2 CAPG

STAUNTON_740_MTX na PON2 LOXL2 NME4 GLO1 MT1H SPARC BTG3 UQCRC2 LGALS3BP COX17 PGD HLA-B /// HLA-C KIAA0114 SERPINE1 PRDX4 LDHA CTGF SNRPC ID3 EIF5A /// LOC143243 COL6A2 PRDX3 IFITM3 /// IFITM2 TGFBI CYC1 PDIA6 PSAP ESD PGRMC1 DPM1 SAP18 CDKN2A SPP1 POLR2F RRAS PRG1 P4HB CYR61 COMT DDT COL1A1 MYH10 NFKBIA MFGE8 ECH1 AP2M1 PTTG1IP CYB5R3 S100A2 IGFBP3 HRMT1L1 CCND1 PRAME SLC7A5 UPP1 COL11A1 CTSC DXS9879E NDRG1 IL8 SRPX CSRP1 ACLY AKR1C3 G6PD S100A10 CAPG IDH3G

STAUNTON_749 na SRM RAB7 COL1A1 TXNIP MFGE8 COL6A2 BST2 TST IER3 TGFBI HLA-F ID2B ZYX ALDH1A1 PLOD1 SERPINH1 G1P2 HLA-A MT2A KRT8 TPM2 PXDN HLA-B /// HLA-C CLU

STAUNTON_4114 na IGFBP3 GPX1 COMT NME4 PFKP KRT18 MT1E TPM1

STAUNTON_4280 na FLNA CRABP2 MT1H FN1 S100A11 HLA-A HSPA1A COX17 IFITM3 /// IFITM2 // CD99 MGLL KRT7 NNMT PFKP HLA-F TGFBI TAX1BP3 AKR1B1 /// EIF3S9 LASP1 CSRP1 CAV1 SFN C10ORF116 TPM1 S100A4 MDK

STAUNTON_26980_MITOMYCINC na SRM MFGE8 KRT18 UQCRH CPNE1 S100A2 ATP6V0B PLP2 CTSL CTPS ARF3 FH LGMN CLU PRDX4 MYC EXOSC7 CDC20 HADH2 SSR4 CRIP1 SLC39A6 HRAS VCL CAP1 EBNA1BP2 CYC1 AKR1B1 /// EIF3S9 NDUFS8 RHOC ZYX LASP1 SPCS2 PBEF1 COX7B PIN1 PGM1 SERPINA1 UCP2 LTB4DH KRT8 MT2A /// LOC441019 LITAF CYBA TPM1 UBE1

STAUNTON_27640 na PON2 FNTA FHL1 FN1 PLOD1 CAST PLP2 HLA-A YWHAH CAPN2 MYL9 HLA-B /// HLA-C CD99 TUBB3 HADH2 CTGF PYGB CRIP1 COL5A2 TGFBI NDUFS8 GPX1 SPCS2 TIMP2 PSAP CAV1 CST3 LTB4DH MCAM ATP6V0C TPM2 DSTN MYH10 TXN DUT MFGE8 THY1 /// LOC94105 TPM4 IER3 FKBP1A ATP6V0B CYB5R3 GNG11 CCND1 PMP22 TUBA1 TAGLN2 ATP6V1F TNFSF7 LGALS1 GPNMB ID2B CAP1 EBNA1BP2 ALDH1A1 ZYX SULT1A3 /// SULT1A4 AKR1B1 /// EIF3S9 LASP1 CSRP1 CDH1 AKR1C3 MSN PGM1 CD9 LITAF CTSH COL6A1 XBP1 ASNS MDK CAPG

STAUNTON_38535 na DSTN FLNA MFGE8 IDI1 KRT18 PCBD1 IGFBP3 ACTN1 OAT TARS LGALS3 /// GALIG EIF3S9 TUBB3 KYNU NDRG1 PDLIM1 COL6A2 FADD COL6A3 SNRPB AKR1B1 /// EIF3S9 PRDX2 TIMP2 CAV1 AKR1A1 MT2A SEMA3C MT2A /// LOC441019 MCAM TPM2 MT1E TPM1 COL6A1 ASNS MDK PHLDA2

STAUNTON_56410 na MAPK6 CSDA ALCAM HMGA1 KRT18 IDH2 MT1H CCNH ANXA5 ITGB1 UFD1L NARS HLA-A SORD TXNRD1 LGALS3BP PGD HLA-B /// HLA-C CD99 KIAA0114 CD81 ETFB CTGF ID3 SNRPC HSPB1 EIF5A /// LOC143243 GRN JUND HRAS IFITM3 /// IFITM2 VCL HLA-F SPINT2 MAGEA3 TAX1BP1 SERPINH1 CAV1 TFRC ITGB5 MCAM PRG1 MT1E TPM1 S100A4 CRIP2 COMT TXN MFGE8 SQSTM1 GNAI2 UQCRH CD151 COX6C PTMS PTTG1IP IGFBP3 CYB5R3 G0S2 GNG11 PRAME CTSL IFITM3 /// IFITM2 // ZNF91 SLC7A5 POLR2J TKT CD59 TXNIP SLC39A6 FAM38A EBNA1BP2 ID2B RHOC AKR1B1 /// EIF3S9 SULT1A3 /// SULT1A4 HEXB ARF5 LASP1 CSRP1 PBEF1 STAT1 G6PD GDF15 PYCR1 EIF3S8 GPI ARF4 MT2A /// LOC441019 LITAF S100A10 NBL1 SEPW1 MDK IFI16 CAPG

STAUNTON_63878_ARAC na CTGF GDF15 SDHA H2AFX EEF1A2 CLU PTTG1IP

STAUNTON_65104 na PABPC1 GUK1 COL1A1 TXNIP S100A10 CD99 ARF5 SERPINE1 PRDX2

STAUNTON_67586 na DCI HMGA1 BZRP NPC2 ATF4 PON2 GSTP1 NME4 IDI1 KRT18 TALDO1 AARS MT1H PDHA1 SPARC EXT1 IRAK1 PGD HLA-B /// HLA-C CLU RBBP7 CBFB COX5B TGFBI SPINT2 EEF1A2 ARPC1B DMPK DDX11 MT1E PHLDA2 B2M RER1 CRABP2 DUT EMP3 IGFBP4 PDXK TM4SF4 FKBP1A IGFBP3 SLC1A5 MAPRE1 CCND1 HSPA1A NQO1 SLC7A5 SNRPB2 BLVRB TKT TMSB4X FAM38A ARMET ALDH1A1 AKR1C1 APOBEC3B AKR1B1 /// EIF3S9 PRDX2 CSRP1 AKR1C3 C20ORF24 PGM1 STMN1 MT2A SFN MT2A /// LOC441019 CYBA COL6A1 ASNS MDK

STAUNTON_76455 na COL1A1 TPM4 STIP1 NDUFV3 IGFBP3 F3 PAFAH1B3 FBL DAP3 NMU CKMT1B UCHL1 C5ORF13 BOP1 HSPB1 SREBF1 M-RIP HLA-F ATP6AP1 SULT1A3 /// SULT1A4 G1P2 ITGB5 TAGLN SLC4A2 MDK

STAUNTON_76627 na PPIF DCI CSDA CLTC GLO1 TIMM17A JTV1 CSK S100A11 NEFL ITGB1 SCARB1 CEBPD TRAP1 CD99 APRT CKMT1B CLU BOP1 TM4SF1 SREBF1 JUP CYC1 ATP6AP1 SSX2 /// SSX3 GPX1 SPCS2 CAV1 HNRPU ESD MAGEA12 NT5E G1P2 ATP5G2 IGFBP2 S100A4 MCM2 CRIP2 CRABP2 DHCR24 EMP3 MFGE8 TPM4 GNAI2 COX6C PTMS PTTG1IP IGFBP3 S100A2 PRAME FKBP4 SIAHBP1 SKB1 DXS9879E CCT2 INPPL1 NIFUN SEC24C PSITPTE22 PPIB G6PD ATP2A2 MT2A UROD MDK CAPG MAGEA2 /// MAGEA2B

STAUNTON_77830 na COL4A2 HLA-F PTMS

STAUNTON_80466 na ECH1 IGFBP4 MEST MT1H CD63 S100A2 IGFBP3 VIM MMP1 HLA-A FSCN1 CAPN2 ALDH1A3 KIAA0114 LGALS3 /// GALIG LGALS1 TM4SF1 CRIP1 FABP5 ID2B TGFBI HLA-F AKR1B1 /// EIF3S9 ZYX CAV1 ITGB5 MT2A /// LOC441019 CYBA S100A4

STAUNTON_90636 na TUBA3 PPGB CTAG1A /// CTAG1B // EXT1 PRDX1 CBX1 CTSD MYL9 HLA-B /// HLA-C CIRBP SEPT7 CYP24A1 CRYAB ID3 MGST1 NNMT TST EEF1A2 AKR1A1 MAGEA12 SMS UCP2 ANXA1 CST3 MCAM MT1E NR2F6 MFGE8 EMP3 SQSTM1 TPM4 PRKAR1A CCND1 CTSL CTAG1B /// CTAG2 /// PMP22 TUBA1 TAGLN2 LGALS3 /// GALIG LGALS1 CTSC GPR143 KIAA0251 IL8 GDI2 M-RIP AKR1B1 /// EIF3S9 CSRP1 S100A10 ACOT7 NBL1 MDK MAD2L1BP CAPG GALE ALCAM NPC2 QSCN6 PON2 MT1H TMED10 FN1 ANXA5 ITGB1 SORD HLA-A SERPINE1 GNB2 CTGF EIF5A /// LOC143243 CDC25B FABP5 HLA-F TGFBI PTDSS1 IL32 KRT8 TPM2 TPM1 DSTN CIB1 COMT COL1A1 DHCR24 LRPAP1 NFKBIA PSMA4 IGFBP4 MGST2 IGFBP3 ATP6V0B S100A2 SNRPN /// SNURF MSLN IFITM3 /// IFITM2 // DUSP1 DXS9879E XRCC5 PRDX2 PPIB GDF15 SFN LITAF CYBA

STAUNTON_94600 na USP14 TOMM20 HMGA1 PSMD7 QSCN6 TUBA3 IDH2 MT1H PCBD1 ANXA2 PTTG1IP IGFBP3 OAT CCND1 UQCRC2 RHOD KIAA0174 LGALS3BP SHC1 SLC7A5 KIAA0114 ALDH1A3 LGMN CLU ETFB CTGF HMGN1 COL5A2 IFITM3 /// IFITM2 VCL TGFBI SULT1A3 /// SULT1A4 RHOC CKAP4 RAB13 G6PD ESD CD9 GPI ARF4 MT2A /// LOC441019 TPM2 MT1E PRG1 TPM1 ARL6IP

STAUNTON_94889 na COMT HMGA1 U2AF1 MFGE8 KRT18 TNNC1 IDH2 SNRPN /// SNURF FBL ITPK1 MYL9 SLC7A5 BLVRB SERPINE1 LGALS3 /// GALIG DXS9879E S100A13 ID3 LY6E EEF1A2 TACSTD2 GUSB GDF15 SFN ATP6V0C MT1E CYBA CTSH

STAUNTON_103704 na FLNA FHL2 GSTP1 CLTC FNTA PPP2CB UFD1L TXNRD1 YWHAH PGD MYL9 CAPN2 CTSD HLA-B /// HLA-C TYMS TUBB3 KARS HK1 SNRPC ID3 NNMT CRIP1 PFKP JUND HRAS COL5A2 TST VCL CBFB GGH TAX1BP3 MAGEA3 CKAP4 SPCS2 GPX1 SUMO2 CAV1 PPM1F RNPEP ANXA1 MCAM ATP6V0C POLD4 TXN MFGE8 EMP3 DUT TPM4 AP2M1 CD63 PRAME CTSL SLC7A5 TAGLN2 LGALS3 /// GALIG EEF1D CD47 LY6E ARMET AKR1B1 /// EIF3S9 SULT1A3 /// SULT1A4 CSRP1 MRCL3 RAB13 G6PD MSN PYCR1 ARF4 MT2A PEA15 MDK CAPG QSCN6 LOXL2 H3F3A /// LOC440926 KRT18 KRT19 MT1H IDH2 ASS ITGB1 ANXA5 HLA-A COL4A2 CD24 SERPINE1 ETFB LEPROT CTGF GRN EIF5A /// LOC143243 IFITM3 /// IFITM2 FTL PTS SND1 HLA-F CYC1 HNRPK ARPC1B LGALS9 TMSB10 TPM2 PRG1 TPM1 COMT COL1A1 TOMM20 DDT HADHB MYH9 GARS IGFBP4 IER3 GNAI2 CD151 UQCRH PTTG1IP CYB5R3 S100A2 ATP6V0B OAT GNG11 HSPA1A ATP6V1F KRT7 ACTG1 ANXA11 EBNA1BP2 RHOC AKR1C1 PRDX2 LASP1 ACLY GAS6 CDH1 AKR1C3 PGM1 GNAS PLEC1 EIF3S2 ANXA4 LITAF LSM1 CYBA CTSH

STAUNTON_105132 na IGFBP3 G6PD GNG11 RHOD LGALS3BP KRT19 ECH1 PRG1 MT1E SLC7A5

STAUNTON_122750 na CYR61 DSTN MFGE8 KRT19 TUBA3 NDUFV3 RHOA MSLN FSCN1 CTSD SLC7A5 BLVRB UCHL1 LGALS3 /// GALIG TUBB3 MCM7 ID3 SREBF1 TGFBI AKR1B1 /// EIF3S9 PPIB PSITPTE22 MSN STMN1 CD9 CDKN2A TPM2 MT1E TPM1 ACOT7 MDK

STAUNTON_127716 na CD74 LGALS1 NDRG1 CRABP2 SRI BST2 TGFBI HLA-F RBM3 GPX1 GUK1 HLA-A SPP1 IFITM3 /// IFITM2 // HMGA2 CD99 S100A10 MDK

STAUNTON_132483 na CYR61 DDT ALCAM SLC6A8 MFGE8 PLOD1 IGFBP3 PRAME HLA-A LGALS3BP PMP22 HLA-B /// HLA-C SKB1 HADH2 DXS9879E CTGF TXNIP LY6E EIF5A /// LOC143243 SND1 ECM1 HLA-F RHOC PRDX2 PSAP SERPINH1 MAGEA12 LGALS9 CST3 IL32 KRT8 MT1E CYBA ODC1 S100A4 P4HB

STAUNTON_135758 na ANP32B NPC2 IDH2 MT1H FN1 ATP6V0B OAT CCND1 CTSL RHOD COL4A2 IFITM3 /// IFITM2 // HLA-B /// HLA-C SLC7A5 CD81 LGMN CLU ETFB MGLL LEPROT GRN FABP5 IFITM3 /// IFITM2 VCL HLA-F CAP1 M-RIP EBNA1BP2 ARPC2 AKR1A1 CD9 ARF4 G1P2 MT2A /// LOC441019 LITAF TPM1

STAUNTON_135962 na EIF2S1 NPC2 HMGA1 FHL2 UBE2L3 NME4 TUBA3 KRT19 IFI30 SFRS11 IDH2 ITGB1 MCM5 PLP2 HLA-A RHOD MYL9 H2AFX LGMN CTGF HK1 SNRPC ID3 SREBF1 HRAS LENG4 ANXA1 CST3 KIAA0101 MT1E BMP4 TPM1 TSPAN7 ALG3 CRABP2 PSMA4 GARS ATIC AP2M1 YWHAE PRKAR1A PTTG1IP ATP6V0B IGFBP3 SNRPN /// SNURF CCND1 HSPA1A NQO1 PXDN CSTB BLVRB CD59 KRT7 MLLT11 BCAP31 SLC39A6 BST2 FAM38A SDHA EBNA1BP2 PCNA AKR1B1 /// EIF3S9 LASP1 PRDX2 MX1 PYCR1 GDF15 PGM1 SLC4A2 LITAF ARL6IP

STAUNTON_136722 na NDRG1 ALCAM MGST1 LOXL2 HLA-B /// HLA-C ID2B TPM1 TGFBI ZYX

STAUNTON_139105 na FHL2 UQCRFS1 GLO1 VIL2 MTHFD2 PDHA1 PHB IMPDH1 HSPD1 CIRBP HLA-B /// HLA-C CTNNB1 TYMS TARS EIF3S6 MYC SHFM1 NNMT RANBP1 ATP6AP1 SUMO2 CARS POLR2F P4HB CYR61 ADSL ATIC BNIP3 ACTN1 DAP3 CTSL RARS PTP4A1 PXDN ALDH1A3 PSMC2 HSPE1 NEU1 C1QBP ECM1 HEXB COX7B RAB13 PSITPTE22 GPI MT2A MT2A /// LOC441019 ASNS RAE1 LOXL2 PON2 IDI1 KRT18 MT1H STIP1 PDHB FN1 HADHA PIR FBL MRPL12 SORD HLA-A COL4A2 FSCN1 SERPINE1 ETFB BOP1 CTGF SREBF1 EIF5A /// LOC143243 IFITM3 /// IFITM2 HLA-F COX5B CYC1 IGFBP6 ARPC1B SAP18 SPP1 ITGB5 IL32 MITF IMMT DDT COL1A1 GSTO1 DHCR24 NDUFS1 H1FX UQCRH ARPC1A S100A2 NQO1 BLVRB POLR2J DXS9879E ERCC1 STRA13 XRCC5 PCNA SDHA RHOC PLSCR1 SFN IMPDH2 BRD2 MAGEA2 /// MAGEA2B

STAUNTON_140701 na HMGA1 NPC2 GSTP1 PON2 SLC6A8 KRT18 PPGB MT1H CTAG1A /// CTAG1B // SPARC FN1 PIR ANXA5 PRDX1 HLA-A LGALS3BP COX17 CD99 SERPINE1 TM4SF1 EIF5A /// LOC143243 IFITM3 /// IFITM2 VCL TGFBI HLA-F SPINT2 MAGEA3 TAX1BP3 GPX1 ARPC1B SERPINH1 CAV1 AKR1A1 MAGEA12 PTDSS1 SMS ANXA1 CST3 MT1E TPM1 ATP6V1E1 DDT DHCR24 NFKBIA LRPAP1 MFGE8 IGFBP3 ATP6V0B GNG11 PRAME CCND1 CTAG1B /// CTAG2 /// IFITM3 /// IFITM2 // SHC1 DUSP1 CXCL1 LGALS3 /// GALIG SLC39A6 IL8 GPR143 FAM38A ECM1 CAP1 PRDX2 C20ORF24 PGM1 CD9 MT2A SFN MT2A /// LOC441019 LITAF CYBA NBL1 MDK MAGEA2 /// MAGEA2B CAPG MAGEA1

STAUNTON_141540_ETOPOSIDE na LGALS1 CRIP1 MFGE8 KRT18 DIPA VIL2 TPM4 CALD1 PTMS SPINT2 PSITPTE22 SEC61B PRSS11 DDX11 PRG1 CD99 B2M

STAUNTON_143095 na FHL2 CLTC NME4 RPN2 LAMB1 PPGB SPARC EXT1 NOMO1 /// NOMO2 /// CEBPD APLP2 PRDX1 UQCRC2 PSMA6 YWHAH CTSD PGD HLA-B /// HLA-C CLTA CD99 SEPT7 CYP24A1 C1S UCHL1 KYNU PDLIM1 ALDH9A1 HK1 MGST1 CRYAB ALDH2 PFKP COL5A2 SOD2 GGH GPX4 TMED9 TAX1BP3 EEF1A2 TIMP2 TAX1BP1 CAV1 SERPINH1 CST3 TAGLN KDELR2 TSPAN3 CCNG1 EMP3 ECH1 TPM4 PCBD1 CD63 COX6C PRKAR1A LAMC1 GADD45A PRAME CCND1 CTSL PMP22 PXDN SLC7A5 LGALS3 /// GALIG FSTL1 NR4A1 TKT CTSC MCM7 TI-227H KIAA0251 PTP4A2 IL8 TMSB4X GDI2 ECM1 ID2B PTGES3 APP HEXB AKR1B1 /// EIF3S9 G6PD GPI MT2A MT2A /// LOC441019 NBL1 XBP1 PEA15 MDK CAPG GLG1 NPC2 PSMD7 QSCN6 LOXL2 PON2 IDH2 STIP1 TPBG FN1 ASS MIF PLOD1 ITGB1 ANXA5 CCT6A BTG3 HLA-A COL4A2 LAMP1 CLU GCLM PRDX4 CTDSP2 ABL1 CTGF LOX UGCG SREBF1 PAX8 COL6A2 FABP5 TGFBI HLA-F SPINT2 PHC2 IFITM1 ARPC1B PSAP NT5E G1P2 C1R HSPG2 CDKN2A ATP5G2 TPM2 DDX11 GNB1 TPM1 P4HA1 SLC3A2 B2M COMT TOMM20 DDT COL1A1 PLEKHC1 PLAUR LRPAP1 MYH9 NFKBIA GARS C1ORF16 IGFBP4 NDUFV3 PTTG1IP S100A2 IGFBP3 HSPA1A LDHB IFITM3 /// IFITM2 // BLVRB LOC23117 /// DKFZP54 DXS9879E TXNIP SLC39A6 STRA13 CRYZ VDAC2 SDHA NDUFA4 RHOC SRPX PRDX2 ACLY GAS6 PGM1 GDF15 SFN MATN2 ANXA4 COL6A1

STAUNTON_145668 na FHL2 TUBA3 IFI30 FNTA PHB MCM5 ARF3 CAPN2 PGD CD99 CTNNB1 SEPT7 TYMS H2AFX LGMN MYC HK1 ID3 MGST1 NNMT SRI TST GPX4 TAX1BP3 NDUFS8 EEF1A2 TAX1BP1 SERPINH1 AKR1A1 PRSS11 MCAM MT1E CYR61 MFGE8 SQSTM1 ARHGDIB PTMS GOT2 ACTN1 HRMT1L1 PAFAH1B3 CCND1 PXDN TUBA1 MGLL HMGB2 C1QBP TMSB4X ID2B CAP1 TACSTD1 AKR1B1 /// EIF3S9 ZYX ALDH1A1 CSRP1 COX7B G6PD PYCR1 MT2A /// LOC441019 S100A10 MDK CAPG YWHAZ HMGA1 LOXL2 KRT18 KRT19 MT1H IDH2 TMED10 FN1 PLOD1 ITGB1 FBL PLP2 FSCN1 BAT3 CLU CTGF HPRT1 SREBF1 PAX8 GRN IFITM3 /// IFITM2 FABP5 SPINT2 TFRC G1P2 CDKN2A ITGB5 KRT8 KIAA0101 TPM2 TPM1 TSPAN7 ALG3 B2M SRM PSMA4 IER3 CD151 IGFBP3 ATP6V0B IFITM3 /// IFITM2 // CSTB C12ORF8 POLR2J SIAHBP1 CDC20 MLLT11 NDRG1 TXNIP CD7 SLC39A6 BCAP31 FAM38A EBNA1BP2 SDHA RHOC LASP1 ARPC2 STMN1 PGM1 DDX48 LITAF SLC4A2 CCND3 COL6A1 ARL6IP BRD2

STAUNTON_146268 na CSDA PON2 KRT18 TUBA3 MT1H JTV1 GTF2I /// GTF2IP1 PLOD1 HLA-A LAMP1 FSCN1 CTSD PPAP2B MYL9 LDHA CTGF ALDH2 COL6A2 COL5A2 VCL HLA-F TGFBI ARPC1B SERPINH1 PGRMC1 AKAP12 PTDSS1 CDKN2A SPP1 CST3 AP3S1 IL32 MCAM CDA MT1E POLD4 COMT DSTN MYH10 DDT CCNG1 PLEKHC1 MFGE8 IGFBP4 BNIP3 IGFBP3 CYB5R3 GNG11 CCND1 IFITM3 /// IFITM2 // NQO1 SLC7A5 ALDH1A3 UPP1 LGALS3 /// GALIG TNFSF7 CTSC MLLT11 COPB2 ID2B PTGES3 SRPX ARF5 G6PD GDF15 CD9 MT2A /// LOC441019 CTSH COL6A1 NBL1 CAPG SEMA3B MAGEA1

STAUNTON_166641 na EIF2S1 GSTP1 TUBA3 IFI30 CTAG1A /// CTAG1B // ANXA2 TMED10 PLP2 PRDX1 RHOD FH MST1R LGMN MYC PRDX4 SERPINB6 EXOSC7 HK1 CTGF CRYAB UGCG HPRT1 RBBP7 CRIP1 HRAS VCL TGFBI SPINT2 NDUFS8 EMD GYPC SPP1 UCP2 ANXA1 CST3 MT1E TPM1 NR2F6 TSPAN7 CETN2 CYR61 GSTO1 ECH1 IER3 IGFBP4 PCBD1 YWHAE ACTN1 S100A2 ATP6V0B VIM SLC1A5 CCND1 CTAG1B /// CTAG2 /// CTPS NQO1 TUBA1 MLLT11 NDRG1 TXNIP SSR4 FADD TMSB4X ECM1 EBNA1BP2 ID2B ZYX SRPX RHOC PGM1 LITAF CYBA CCND3 ACOT7 UBE1

STAUNTON_174121 na COMT HLA-A CST3 CTSD MYL9 IGFBP4 CYBA

STAUNTON_177382 na DCI KRT18 IDH2 MT1H TRAP1 HLA-A CD24 PGD ITPK1 HLA-B /// HLA-C CD99 CLU HIST2H2AA KARS MGST1 ID3 CRIP1 JUP FABP5 ATP6AP1 EEF1A2 HSPA4 CAV1 ANXA1 TMSB10 KRT8 MCAM ALDOC POLD4 MT1E UBE2S TPM1 S100A4 PHLDA2 CRIP2 CIB1 COMT ECH1 ATP6V0B VIM SSX2 CCND1 PRAME ACADVL IFITM3 /// IFITM2 // TAGLN2 BLVRB G6PD MT2A MT2A /// LOC441019 LITAF AES XBP1

STAUNTON_184692 na DSTN DDT NPC2 MFGE8 SLPI IGFBP4 TMED10 OAT NOMO1 /// NOMO2 /// GNG11 LGALS3BP PPAP2B CTSD TUBA1 CD99 UCHL1 LGALS3 /// GALIG CTGF COPB2 AKR1B1 /// EIF3S9 ARPC1B PGM1 CST3 TPM1 IGFBP2 ABLIM1 B2M

STAUNTON_208652 na CSDA GLO1 TUBA3 PPGB ATP5J SPARC EXT1 FLII NOMO1 /// NOMO2 /// LGALS3BP CAPN2 CTSD HLA-B /// HLA-C CD99 TYMS LGMN C1S UCHL1 HADH2 PDLIM1 CRYAB TM4SF1 NNMT JUP PLAU TAX1BP3 NDUFS8 EEF1A2 PAM TAX1BP1 TIMP2 SERPINH1 TEGT CST3 MCAM TAGLN MT1E NR2F6 CRIP2 CYR61 TSPAN3 MYH10 CRABP2 MFGE8 ECH1 SLPI TPM4 ATIC FKBP1A RBBP8 LAMC1 GADD45A SSX2 COX4I1 HOXB2 EEF1D LGALS3 /// GALIG FSTL1 PSMC2 COL4A1 DIPA KIAA0251 COPB2 M-RIP APP PLOD2 HEXB ARF5 CSRP1 NEDD8 G6PD RAB13 MSN S100A10 NBL1 XBP1 SEPW1 ODC1 PEA15 TFPI2 GLG1 NPC2 HMGA1 LOXL2 H3F3A /// LOC440926 KRT18 KRT19 IDH2 STIP1 TPBG TMED10 ASS FN1 MIF PLOD1 FBL SCARB1 HLA-A COL4A2 LAMP1 COX17 CD24 TUBG1 PPAP2B CLU PRDX4 CTDSP2 GNB2 CTGF BOP1 S100A13 HSPB1 DNAJA1 SREBF1 COL6A2 CALD1 FABP5 TGFBI HLA-F CYC1 SPINT2 IFITM1 LOXL1 HSPA4 ARPC1B C1R CDKN2A HSPG2 ITGB5 IGFBP5 TMSB10 KRT8 IL32 ACOT2 TPM1 P4HA1 CETN2 B2M COMT DSTN COL1A1 PLAUR GNAI2 IGFBP4 H1FX ARPC1A PTTG1IP CYB5R3 IGFBP3 S100A2 OAT HSF1 MSLN HSPA1A IFITM3 /// IFITM2 // NQO1 SHC1 HLA-DRB1 DUSP1 C5ORF13 CD59 KRT7 DXS9879E NDRG1 SSR4 SURF1 XRCC5 PCNA SDHA SRPX PRDX2 GAS6 PPIB GDF15 PGM1 MATN2 COL6A1

STAUNTON_209835 na CSDA FLNA FHL2 CLTC FNTA LTA4H RHEB SPARC YWHAH CTSD MYL9 ITPK1 HLA-B /// HLA-C CD99 ID3 NNMT VCL CKAP4 SUMO2 SERPINH1 ANXA1 TAGLN MT1E P4HB CYR61 NEDD5 U2AF1 EMP3 PRAME KIAA0152 CTSL SLC7A5 TAGLN2 FSTL1 LGALS3 /// GALIG RAB2 LGALS1 COL4A1 KRT10 LY6E IL8 TMSB4X ECM1 AKR1B1 /// EIF3S9 CSRP1 MSN INHBA TFPI2 MAP3K10 LOXL2 HSPH1 KRT18 MT1H IDH2 SNRPF FN1 FBL SORD COL4A2 FSCN1 SERPINE1 LEPROT CTGF S100A13 RBBP7 COL6A2 FABP5 IFITM3 /// IFITM2 TGFBI HLA-F SPINT2 HNRPK PDIA6 DDX5 PGRMC1 ESD GUSB KPNA2 IL32 KRT8 KIAA0101 CDA TPM2 PRG1 TPM1 SRM TOMM20 COL1A1 IGFBP4 CD151 PTTG1IP S100A2 IGFBP3 CYB5R3 VIM MSLN GNG11 AHCY UBE2V2 KRT7 COL6A3 GAS6 ACLY GDF15 CD9 LITAF CYBA COL6A1 FGFR1

STAUNTON_219734 na S100A2 CD9 IFITM3 /// IFITM2 // TUBA3 FABP5 CYBA HLA-F FN1 ZYX

STAUNTON_224131 na CSDA ATF4 TALDO1 PPGB SPARC RPL36AL BENE DDX39 FLII XRCC6 NOMO1 /// NOMO2 /// PRDX1 APLP2 RHOD PSMA6 GPC1 CTSD PGD MYL9 TOP2A HLA-B /// HLA-C CIRBP SEPT7 CD81 LGMN C1S UCHL1 CPSF1 SNRPC CRYAB MGST1 NNMT TST GPX4 TAX1BP3 ATP6AP1 EEF1A2 PAM SPCS2 TAX1BP1 GPX1 SERPINH1 MAGEA12 TEGT SMS CST3 MCAM MT1E KDELR2 S100A4 CRIP2 CYR61 EMP3 SQSTM1 ECH1 TPM4 ARHGDIB PSMC3 RAC1 PTMS PRKAR1A BNIP3 LAMC1 CCND1 KIAA0152 FKBP4 SLC7A5 ALDH1A3 TAGLN2 LGALS3 /// GALIG FSTL1 SKB1 LGALS1 KIAA0251 PTP4A2 IL8 GNG10 /// LOC552891 TMSB4X M-RIP TACSTD1 ALDH1A1 APP HEXB ARF5 AKR1B1 /// EIF3S9 ZYX G6PD MSN MT2A /// LOC441019 S100A10 ACOT7 SEPW1 LTBR GLG1 NPC2 IDI1 KRT18 KRT19 STIP1 MT1H IDH2 TPBG ASS FAM50A TMED10 PLOD1 CCT6A HLA-A LAMP1 PPAP2B ADAM15 BCL3 SERPINE1 CLU PRDX4 MXI1 CD74 ACO2 GNB2 CTDSP2 HSPB1 UGCG TNC COL6A2 IFITM3 /// IFITM2 CALD1 HLA-F SPINT2 IFITM1 PDIA6 PSAP TFRC NT5E G1P2 AKAP12 CDKN2A HSPG2 ITGB5 KRT8 ACOT2 KIAA0101 TPM2 TPM1 DSTN COMT COL1A1 RPS4Y1 MYH9 CELP IGFBP4 ARPC1A PTTG1IP MGST2 CYB5R3 IGFBP3 S100A2 OAT HSPA1A LDHB NQO1 MIA UPP1 BLVRB PLP1 LOC23117 /// DKFZP54 MLLT11 CD59 NDRG1 CCT2 DXS9879E SURF1 BCAP31 FADD EIF2B2 RHOC PRDX2 SLC25A1 AKR1C3 PGM1 GDF15 CD9 PLEC1 SCNN1A COL6A1

STAUNTON_249992 na GSTP1 NFKBIA SQSTM1 PTMS ANXA2 ARPC1A IGFBP3 UCHL1 CLU DUSP1 MGLL ID3 CRIP1 DIPA GRN PFKP FABP5 FAM38A AKR1B1 /// EIF3S9 LASP1 MSN AKR1A1 CD9 SFN TPM1

STAUNTON_279836 na DHCR24 MFGE8 SFN MT1H EBNA1BP2 FN1 BLVRB LGALS3 /// GALIG TFPI2

STAUNTON_295501 na NPC2 ALCAM NME4 SLC6A8 MT1H SPARC TMED10 LGALS3BP RHOD YWHAH CD24 FSCN1 KIAA0114 SERPINE1 ACO2 TUBB3 SNRPC MGST1 UGCG JUP HLA-F SPINT2 TAX1BP3 MAGEA3 EEF1A2 IGFBP6 PSAP SERPINH1 TFRC MAGEA12 CST3 ITGB5 MT1E TPM1 DSTN COMT DDT TSPAN3 DHCR24 MFGE8 ECH1 IGFBP4 IER3 FKBP1A IGFBP3 CCND1 MVP IFITM3 /// IFITM2 // SHC1 UPP1 TAGLN2 CXCL1 LGALS3 /// GALIG TSC22D1 DXS9879E IL8 FADD PCNA HEXB AKR1B1 /// EIF3S9 GAS6 INHBA C20ORF24 CD9 MT2A /// LOC441019 CYBA S100A10 DAD1 CAPG MAGEA1

STAUNTON_299117 na SERPINB6 SRM COMT IFITM3 /// IFITM2 IDH2 MT1H HLA-F SPARC OAT CAV1 SERPINH1 HLA-A TPM1 UPP1 NBL1 ODC1 LGALS3 /// GALIG

STAUNTON_301460 na CYR61 NPC2 ALCAM BZRP GSTP1 KRT18 MT1H FKBP1A S100A2 APLP2 COL4A2 HYOU1 CD99 H2AFX BLVRB FSTL1 LGALS1 MGST1 NNMT HSPB1 SREBF1 FAM38A FABP5 VCL TGFBI HLA-F PRDX2 GPX1 PSITPTE22 PRSS11 MT2A ANXA1 MT2A /// LOC441019 TPM2 TAGLN

STAUNTON_301477 na HMGA1 NPC2 NME4 KRT18 STIP1 FN1 ILK PLP2 HLA-A TXNRD1 YWHAH CD99 CLU IQGAP1 KARS ID3 PFKP CDC25B HRAS TST HLA-F TGFBI TIMP2 SPCS2 CAV1 CDKN2A ITGB5 ATP6V0C MCAM TPM2 BMP4 TPM1 POLR2G S100A4 HDLBP DSTN COMT NFKBIA MFGE8 SQSTM1 IGFBP4 PTMS UQCRH FKBP1A PTTG1IP ATP6V0B GNG11 CCND1 TUBA1 CDC20 LGALS1 FUCA1 CD59 SLC39A6 FAM38A EBNA1BP2 RHOC AKR1B1 /// EIF3S9 SULT1A3 /// SULT1A4 LASP1 CSRP1 G6PD PGM1 SFN LITAF CYBA SFRS3 S100A10 XBP1 ASNS UBB MDK CAPG

STAUNTON_302325 na SNRPG HMGA1 LOXL2 QSCN6 KRT18 TIMP3 IDH2 SPARC FN1 ADA ILK CCT6A HLA-A TRAP1 LGALS3BP COL4A2 HMGA2 HLA-B /// HLA-C SERPINE1 CLU PRDX4 TUBB3 CTGF NNMT JUP FABP5 IFITM3 /// IFITM2 HLA-F TMED9 LOXL1 TOP1 CKAP4 PRSS11 AKAP12 SPP1 CDKN2A ANXA1 IL32 KRT8 TAGLN TPM2 MT1E TPM1 P4HB CETN2 CYR61 COMT DSTN COL1A1 TSPAN3 TXN DHCR24 MYH9 ECH1 IGFBP4 PTTG1IP IGFBP3 ACTN1 ATP6V0B SLC1A5 GADD45A NPPB SSRP1 LDHB IFITM3 /// IFITM2 // NQO1 PXDN TUBA1 TAGLN2 CXCL1 FSTL1 DUSP1 COL4A1 KIAA0251 TMSB4X CSRP1 GDF15 PGM1 CD9 PARP1 MT2A SFN PLEC1 PSMA3 NUTF2 CYBA S100A10 COL6A1 NBL1 CAPG

STAUNTON_303812 na EIF2S1 COMT NFKBIA DUT ANXA2 CPNE1 IGFBP3 ATP6V0B OAT IRAK1 CCND1 IFITM3 /// IFITM2 // PGD ITPK1 SLC7A5 BAT3 CLU HPRT1 PFKP IFITM3 /// IFITM2 GDI2 VCL HLA-F TGFBI PCNA CYC1 TACSTD1 CSRP1 TAX1BP1 ARPC2 DCTD AKR1A1 G1P2 CD9 CDKN2A LITAF

STAUNTON_305222 na COMT DCI COL1A1 GSTP1 LOXL2 HSPH1 SPARC IGFBP3 MAPRE1 COL4A2 SHC1 SERPINE1 LGALS3 /// GALIG FSTL1 RAB2 KRT7 CTGF KRT10 TM4SF1 S100A13 BCAP31 COL6A2 IL8 COL6A3 TGFBI CSRP1 FXYD3 AKR1C3 G6PD ESD GDF15 SAP18 CDKN2A SFN TPM2 COL6A1 P4HB

STAUNTON_309401 na FLNA FBN1 PPGB CTAG1A /// CTAG1B // SPARC FHL1 LGALS3BP AHNAK ITPK1 CTSD MYL9 PGD HLA-B /// HLA-C CD99 LGMN UCHL1 ELF3 SHFM1 MGST1 TM4SF1 CRYAB NNMT ALDH2 PFKP COL5A2 TST GGH SNRPB CAV1 SERPINH1 AKR1A1 CST3 AP3S1 TAGLN MT1E NCOR2 S100A4 P4HB CRIP2 CYR61 VDAC1 MYH10 EMP3 POLR2H SQSTM1 THY1 /// LOC94105 TPM4 ECH1 PDXK PTMS ACTN1 GADD45A CCND1 PRAME CTAG1B /// CTAG2 /// HDAC1 SLC7A5 TUBA1 TAGLN2 EEF1D LGALS3 /// GALIG FSTL1 CSPG2 MGLL COL4A1 LY6E DGCR6 CAP1 ID2B M-RIP AKR1B1 /// EIF3S9 ZYX ARF5 MSN S100A10 NBL1 ACOT7 ODC1 MDK ABLIM1 RAE1 GLG1 POSTN CAPG HMGA1 LOXL2 PON2 FXYD2 IDH2 MT1H ASS FN1 TMED10 PLOD1 FBL PLP2 SCARB1 MRPL12 HLA-A COL4A2 FH PPAP2B KIAA0114 SERPINE1 CLU LDHA MXI1 CD74 LEPROT ABL1 BOP1 CTGF UGCG SDC1 TNC RBBP7 COL6A2 GRN CDC25B CALD1 FABP5 SND1 HLA-F TGFBI AKAP12 SPP1 CDKN2A ITGB5 RRAS IL32 KRT8 TPM1 SLC3A2 CES2 CIB1 COMT DSTN COL1A1 MCM3 PLEKHC1 PLAUR DHCR24 NFKBIA MYH9 IGFBP4 UQCRH IGFBP3 ATP6V0B CYB5R3 SLC1A5 SNRPN /// SNURF IFITM3 /// IFITM2 // LDHB MVP NQO1 MIA SLC35B1 UPP1 IARS PLP1 TSC22D1 C5ORF13 MLLT11 CD59 KRT7 TXNIP BCAP31 AKR1C1 RHOC LASP1 AKR1C3 GDF15 PGM1 C20ORF24 CD9 UBE2I LITAF CYBA COL6A1 MAGEA1

STAUNTON_314622 na EIF2S1 MAPK6 FLNA GLO1 KRT19 FXYD2 STIP1 FLII FN1 ANXA2 CPNE1 CAST PLP2 TRAP1 RHOD COL4A2 BAT3 ETFB CTGF ID3 SNRPC HPRT1 GRN PFKP VCL TGFBI NDUFS8 MAGEA3 SPCS2 CAV1 DCTD SPP1 CDKN2A TPM1 CYR61 COMT DUT IER3 GNAI2 IGFBP4 H1FX PTTG1IP ATP6V0B LAMC1 PAFAH1B3 OAT SNRPN /// SNURF GNG11 CCND1 CTSL CTPS COX4I1 TUBA1 POLR2B MGLL LOC23117 /// DKFZP54 KRT7 CD59 TXNIP ID2B PSMA5 ALDH1A1 RHOC SULT1A3 /// SULT1A4 PLD3 CSRP1 STMN1 GDF15 ARF4 LITAF PEA15 MDK

STAUNTON_318807 na MAP3K10 MAPK6 HMGA1 LOXL2 LTA4H PPGB AARS MT1H SPARC ASS CPNE1 CAST PRKACG FBL PRDX1 PLP2 HLA-A LGALS3BP CIRBP HLA-B /// HLA-C CD99 CLU IQGAP1 CTGF PDLIM1 TM4SF1 S100A13 MGST1 CRIP1 PLAU HRAS IFITM3 /// IFITM2 SPINT2 EEF1A2 TAX1BP1 TIMP2 GPX1 SERPINH1 CAV1 PSAP HSPG2 ANXA1 KRT8 TPM2 MT1E TPM1 PHLDA2 CRIP2 B2M SRM TSPAN3 COL1A1 CRABP2 MYH9 EMP3 SQSTM1 TPM4 PSMC3 IGFBP4 CD63 PTTG1IP IGFBP3 S100A2 TNFSF9 VIM IFITM3 /// IFITM2 // PXDN TUBA1 SHC1 SLC7A5 NCSTN FSTL1 TKT LGALS1 CTSC NDRG1 TXNIP ID2B SRPX ZYX AKR1B1 /// EIF3S9 G6PD SLC2A1 MSN SEC61B DDR1 CD9 MT2A /// LOC441019 PLEC1 SEPW1 NBL1 MDK

STAUNTON_319725 na CD74 S100A2 BOP1 CST3 FTL CD99 HLA-F HLA-DRB1

STAUNTON_322921 na ALCAM LOXL2 MYH9 KRT18 ECH1 IGFBP4 MT1H NDUFV3 ASS FKBP1A ANXA5 GADD45A CTSL HLA-A COL4A2 IFITM3 /// IFITM2 // TUBG1 CAPN2 HLA-B /// HLA-C CD99 HCCS SERPINE1 FSTL1 LGALS1 TUBB3 PDLIM1 S100A13 TXNIP NNMT SREBF1 ID2B TGFBI PRDX2 SLC25A1 CAV1 SMS CDKN2A MT2A SEMA3C ANXA1 KRT8 MT2A /// LOC441019 TAGLN B2M

STAUNTON_323241 na RAB7 GALE TXN FHL2 MYH9 EMP3 DUT NME4 IGFBP4 SPARC SNRPN /// SNURF LGALS3BP HLA-B /// HLA-C BLVRB LGALS3 /// GALIG ACO2 LGALS1 TST PTS TGFBI ID2B GGH TACSTD1 SPINT2 AKR1B1 /// EIF3S9 TAX1BP1 ARPC1B MSN CST3 MT1E LSM1 MDK PHLDA2

STAUNTON_324979 na RAB7 NPC2 PGK1 PPGB SPARC EXT1 ANXA5 NOMO1 /// NOMO2 /// HLA-A LGALS3BP PMM1 LAMP1 CTSD PPAP2B CD99 LGMN CD74 MXI1 HLA-DRA TM4SF1 NNMT TNC TGFBI HLA-F PHC2 PAM EEF1A2 TIMP2 GPX1 TAX1BP1 PSAP NT5E HSPG2 CST3 ITGB5 MITF MCAM TPM1 B2M CYR61 COMT DDT TSPAN3 COPA CRABP2 DUT EMP3 IGFBP4 CDC37 CD63 FKBP1A IGFBP3 LAMC1 CYB5R3 OAT IFITM3 /// IFITM2 // NQO1 MIA PMP22 HLA-DRB1 SNRPB2 PLP1 LGALS3 /// GALIG LGALS1 ATP1B3 CTSC ATOX1 KIAA0251 APOBEC3B APP SRPX HEXB CSRP1 MSN C20ORF24 PGM1 MATN2 LITAF NBL1 PEA15 RFC4 MDK

STAUNTON_325319 na DRAP1 CRABP2 QSCN6 TUBA3 PFN2 MGST2 IGFBP3 APEX1 CCND1 APLP2 HSPA1A HLA-B /// HLA-C SEPT7 SIAHBP1 LOC23117 /// DKFZP54 GNB2 ALDH9A1 TXNIP CRIP1 HLA-F AKR1B1 /// EIF3S9 EEF1A2 TIMP2 ARPC1B IL32 ATP6V0C IGFBP2 MDK CAPG

STAUNTON_328130 na PPIF HMGA1 CKS2 SLC6A8 GLO1 KRT18 PGK1 TIMM17A MT1H IDH2 AARS PRDX6 NEFL TTC3 CBX3 CEBPD SSR2 FH CTSD RAD23A PRDX4 CPSF1 TUBB3 ALDH9A1 CRYAB M11S1 HPRT1 GRN SRI COL5A2 SND1 RRM1 HLA-F CYC1 PHC2 EIF1AX PAM PSMD4 ARPC1B PGRMC1 SMS FXR1 KRT8 GNPDA1 MT1E P4HA1 ALG3 MFGE8 JUNB AP3D1 ATP6V0B IGFBP3 HRMT1L1 HSF1 GUK1 SSRP1 IFITM3 /// IFITM2 // MRPL23 PXDN FSTL1 POLR2J CSNK1E RRAGA C5ORF13 CD47 CD59 DXS9879E SSR4 ALAS1 DIPA STOM ID2B PRDX2 PBEF1 COX7B C20ORF24 MT2A /// LOC441019 SLC4A2 SEPW1 NBL1 DAD1

STAUNTON_328784 na PON2 LOXL2 KRT19 LAMB1 FN1 ITGB1 CEBPD HLA-A LAMP1 COX17 TOP2A BCL3 CPSF1 TUBB3 TM4SF1 ID3 NNMT TST FTL HLA-F CKAP4 CAV1 PRSS11 NT5E CDKN2A CST3 LTB4DH ATP5G2 TAGLN TPM1 JUNB EMP3 SQSTM1 IGFBP4 FKBP1A PTTG1IP S100A2 NQO1 PSMB10 SHC1 GAS1 RRAGA NIFUN ID2B AKR1C1 HEXB LASP1 PRDX2 CSRP1 STAT1 ACLY AKR1C3 PSITPTE22

STAUNTON_335791 na SNRPN /// SNURF CTGF ID3 HLA-A HSPA1A MT2A MT2A /// LOC441019 PPGB MT1H SDHA

STAUNTON_335794 na BZRP NME4 TIMP3 PDHA1 RPL36AL SPARC EXT1 JTV1 APEX1 PRKACG APLP2 PRDX1 RHOD LGALS3BP NDUFA12 YWHAH CTSD CIRBP SERPINB6 MGST1 TM4SF1 SNRPC KHSRP NNMT PLAU JUND TMED9 SNRPB EEF1A2 CKAP4 GPX1 SUMO2 SERPINH1 CAV1 PTPRF ATP6V0C MCAM TAGLN MT1E UBE2S IGFBP2 TRA1 PHLDA2 CRIP2 CYR61 MYH10 TSPAN3 MFGE8 EMP3 DUT POLR2H TPM4 PSMC3 ARHGDIB RAC1 AP2M1 RBBP8 BNIP3 ACTN1 LAMC1 GADD45A CTSL HDAC1 ACAT1 TUBA1 SLC7A5 ALDH1A3 NCSTN LGALS3 /// GALIG LGALS1 TKT CTSC COL4A1 DVL3 PRNP KIAA0251 PTP4A2 ID2B M-RIP SULT1A3 /// SULT1A4 ZYX HEXB MGP COX7B G6PD SLC2A1 RAB13 DDR1 MT2A MT2A /// LOC441019 PSMD6 NBL1 PEA15 CAPG MAP3K10 GALE LOXL2 QSCN6 IDI1 MT1H IDH2 TPBG TMED10 FN1 ANXA5 CAST HLA-A SORD COL4A2 COX17 FSCN1 CD24 ADAM15 CLU LOX CTGF S100A13 HSPB1 UGCG SREBF1 RBBP7 IFITM3 /// IFITM2 COX5B TGFBI HLA-F HNRPK HSPG2 SPP1 ITGB5 KIAA0101 TPM1 SLC3A2 SRM COMT DDT COL1A1 PLAUR PSMA4 MYH9 IGFBP4 GNAI2 ARPC1A PTTG1IP IGFBP3 CYB5R3 OAT SNRPN /// SNURF NPPB RPL34 LDHB SHC1 BLVRB SNRPB2 NDRG1 SSR4 SURF1 EIF2B2 XRCC5 AP2B1 PRDX2 CD9 PLEC1 SCNN1A PSMA3 COL6A1

STAUNTON_348977 na NME4 FBN1 TUBA3 H3F3A SPARC JTV1 ILK ISG20 NARS PRDX1 TXNRD1 LGALS3BP MYL9 HSPA5 CIRBP HLA-B /// HLA-C CTNNB1 C1S UCHL1 SERPINB6 TUBB3 ALDH9A1 TM4SF1 CRYAB SNRPC NNMT CRIP1 TST SNRPB MAGEA3 PAM TOP1 TEGT SMS CST3 ANXA1 TAGLN POLD4 MT1E S100A4 CRIP2 CYR61 NEDD5 TSPAN3 POLR2H SLPI TPM4 PDXK AP2M1 PTMS BNIP3 LAMC1 HRMT1L1 CTSL COX4I1 ALDH1A3 FSTL1 LGALS3 /// GALIG COL4A1 HMGB2 CTSC TMSB4X ALDH1A1 ZYX AKR1B1 /// EIF3S9 STAT1 G6PD PSITPTE22 SLC2A1 GPI MT2A NUTF2 S100A10 ACOT7 ODC1 MDK CAPG PCDHGC3 /// PCDHGB4 H3F3A /// LOC440926 PON2 KRT18 FXYD2 IDH2 FN1 PDHB TMED10 PLOD1 HLA-A COL4A2 FSCN1 FH PPAP2B BCL3 SERPINE1 GCLM SREBF1 RBBP7 EIF5A /// LOC143243 FABP5 HLA-F CYC1 IFITM1 LOXL1 DDX5 AKAP12 CDKN2A SPP1 KRT8 TPM2 TPM1 P4HA1 HDLBP COMT DHCR24 NFKBIA IGFBP4 GNAI2 IER3 IGFBP3 ATP6V0B SNRPN /// SNURF IFITM3 /// IFITM2 // SSRP1 BLVRB LOC23117 /// DKFZP54 FUCA1 CD59 DXS9879E TXNIP ANXA11 CRYZ SDHA AKR1C1 PRDX2 AKR1C3 GDF15 PARP1 PLEC1 ANXA4 COL6A1 MAGEA2 /// MAGEA2B BRD2 MAGEA1

STAUNTON_349622 na MAPK6 LRRC14 FLNA GSTP1 NME4 CLTC TUBA3 ATP5J PDHA1 SPARC JTV1 GTF2I /// GTF2IP1 UFD1L NARS UQCRC2 LGALS3BP AHNAK SSR2 ARF3 MYL9 HLA-B /// HLA-C MAZ CIRBP H2AFX TARS COX5A DLD MYC EIF3S9 SNRPC EIF1AX MAGEA3 ATP6AP1 EEF1A2 SUMO2 GPX1 TIMP2 SERPINH1 HNRPU CAV1 AKR1A1 PPM1F MAGEA12 RNPEP MCAM POLD4 NCOR2 TRA1 FARSLA S100A4 P4HB VDAC1 USP14 PHB2 RPL39 DUT EMP3 MFGE8 SQSTM1 TPM4 RAC1 PTMS COX6C RABGGTB HNRPF PRAME CTSL PTP4A1 MORF4L2 ALDH1A3 LGALS3 /// GALIG RRAGA MGLL SKB1 LGALS1 MCM7 KRT10 C1QBP TCP1 XPO1 TMSB4X FASN NIFUN ID2B ARF5 COX7B G6PD MSN PYCR1 GPI MT2A SET MT2A /// LOC441019 NBL1 ASNS MDK CAPG ALCAM HMGA1 SLC6A8 IDI1 KRT18 PGK1 MT1H PCCB TMED10 FN1 HNRPA2B1 HADHA PLOD1 HSPA9B HAX1 CBX3 POLD2 SORD TRAP1 HLA-A COL4A2 FSCN1 KIAA0114 UQCRB PRDX4 CTGF BOP1 UGCG HSPB1 SREBF1 FABP5 CALD1 TGFBI HLA-F SPINT2 SLC39A14 CYC1 HNRPK BCLAF1 PSAP DDX5 ESD GUSB SPP1 ITGB5 KIAA0101 KRT8 IMMT DSTN ANP32B COL1A1 ATP5A1 EIF4B GARS IER3 GNAI2 SLC25A5 AP3D1 UQCRH PTTG1IP IGFBP3 CYB5R3 GNG11 HSPA1A MRPL23 AHCY UPP1 BLVRB C12ORF8 POLR2J MLLT11 DXS9879E TXNIP SURF1 INPPL1 SLC39A6 FAM38A ECHS1 SDHA NDUFA4 PLCD1 PRDX2 ACLY DDX48 NDUFV2 SFRS5 EIF3S2 PSMA3 SLC4A2 CYBA IMPDH2 MAGEA2 /// MAGEA2B

STAUNTON_351521 na COMT UBE2L3 DHCR24 MYH9 SQSTM1 FXYD2 IGFBP4 SNRPF PTMS TMED10 S100A2 ATP6V0B ISG20 CCND1 PRAME HLA-A KIAA0152 LDHB CTPS CTSD HMGA2 HLA-B /// HLA-C TUBA1 PDIA3 SEPT7 TAGLN2 TKT KYNU TCP1 FABP5 TST TMSB4X DGCR6 HLA-F TAX1BP3 AKAP12 NT5E SPP1 ANXA1 CST3 CYBA CAPG B2M

STAUNTON_355457 na H3F3A /// LOC440926 GLO1 RPN2 PLOD1 NOMO1 /// NOMO2 /// LGALS3BP PSMA6 YWHAH SERPINE1 UCHL1 CLU GCLM PRDX4 MGST1 CRYAB GRN TGFBI SPINT2 PHC2 G1P2 MT1E P4HB SLC3A2 CIB1 COMT CCNG1 PSMA4 EMP3 NFKBIA MFGE8 PSMC3 PTMS NPIP /// LOC23117 // PTTG1IP ACAT1 TUBA1 NCSTN BLVRB LGALS3 /// GALIG LOC23117 /// DKFZP54 C5ORF13 EIF2B2 PTP4A2 KIAA0251 GNG10 /// LOC552891 SRP14 NDUFA4 AKR1B1 /// EIF3S9 HEXB PSITPTE22 PGM1 PLEC1 PSMA3 CYBA DAD1 COL6A1 GLG1

STAUNTON_356885 na PPIF FLNA ALCAM CRABP2 MFGE8 PDXK PDHA1 NDUFV3 PTMS HSF1 COL4A2 HSPA1A PTP4A1 ARF3 HSPD1 BIRC5 KIAA0114 CKMT1B POLR2J MGLL MCM7 KRT7 BOP1 DXS9879E NNMT SREBF1 FABP5 LAMB3 NIFUN HLA-F CYC1 AKR1B1 /// EIF3S9 TAX1BP3 LASP1 GPX1 CAV1 AKR1A1 RNPEP ANXA1 SFN MCAM MT2A /// LOC441019 SLC4A2 TPM1 FARSLA MDK

STAUNTON_357885 na HMGA1 BZRP UBE2L3 GSTP1 TUBA3 MT1H IDH2 ASS FN1 TMED10 HLA-A LGALS3BP FKBP2 MYL9 CTSD HLA-B /// HLA-C H2AFX SERPINE1 CDKN3 UCHL1 MYC KYNU NNMT GRN FABP5 TGFBI SPINT2 NDUFS8 ARPC1B SERPINH1 CST3 ANXA1 IL32 MT1E COMT DSTN COL1A1 ATP6V0B IGFBP3 HSPA1A IFITM3 /// IFITM2 // SLC7A5 BLVRB DXS9879E SSR4 CD7 BST2 ARMET PRDX2 G6PD PPIB AKR1C3 STMN1 CD9 SFN MT2A /// LOC441019 MDK CAPG

STAUNTON_363979 na MAPK6 NPC2 HMGA1 FHL2 LOXL2 H3F3A /// LOC440926 QSCN6 NME4 KRT18 FNTA LAMB1 IDH2 FN1 ASS FLII PLOD1 CAST HLA-A COL4A2 CTSD HLA-B /// HLA-C MMP2 UCHL1 TUBB3 SNRPC UGCG RBBP7 COL6A2 PFKP COL5A2 TGFBI HLA-F PHC2 TAX1BP3 EEF1A2 PAM CKAP4 SERPINH1 PGRMC1 TACSTD2 TEGT G1P2 CDKN2A CST3 IL32 TPM1 P4HA1 CRIP2 CYR61 COL1A1 CRABP2 MFGE8 GARS IGFBP4 NDUFV3 FKBP1A RHOA S100A2 IGFBP3 OAT PAFAH1B3 SNRPN /// SNURF MSLN HSPA1A PXDN IARS LOC23117 /// DKFZP54 MGLL KRT7 MCM7 DXS9879E TXNIP SLC39A6 FAM38A ECM1 NIFUN ID2B AKR1B1 /// EIF3S9 PLOD2 ZYX PRDX2 MSN STMN1 SEC61B SFN PSMD6 XBP1 COL6A1 CKB MDK

STAUNTON_364830 na MGLL MLLT11 CCND1 GPI MT1H MT1E

STAUNTON_368390 na TUBA3 TALDO1 XRCC6 CCNH PRKACG NOMO1 /// NOMO2 /// APLP2 LGALS3BP RHOD PSMA6 GPC1 TOP2A CTSD SEPT7 LGMN C1S TUBB3 HADH2 CRYAB MGST1 SNRPC GPX4 TMED9 TAX1BP3 EEF1A2 PAM CKAP4 TAX1BP1 ANXA1 CST3 MCAM MT1E PHLDA2 CYR61 DUT MFGE8 EMP3 SQSTM1 TPM4 ARHGDIB PRKAR1A LAMC1 HRMT1L1 CCND1 KIAA0152 TUBA1 SLC7A5 TAGLN2 LGALS3 /// GALIG LGALS1 TI-227H IL8 KIAA0251 TMSB4X ID2B TACSTD1 ZYX APP ALDH1A1 HEXB ARF5 MDK LTBR GLG1 TTC1 GALE HMGA1 NPC2 PON2 QSCN6 SLC6A8 PGK1 MT1H IDH2 TMED10 FAM50A PLOD1 ANXA5 SORD HLA-A BECN1 PPAP2B SERPINE1 GCLM GNB2 S100A13 UGCG RBBP7 COL6A2 CALD1 HLA-F SPINT2 IFITM1 ARPC1B PSAP PGRMC1 TFRC HSPG2 ITGB5 KIAA0101 TPM2 DSTN COMT DDT MYH9 IER3 CA9 ARPC1A PTTG1IP IGFBP3 S100A2 NQO1 SHC1 BLVRB LOC23117 /// DKFZP54 CD59 DXS9879E TXNIP BCAP31 FADD EIF2B2 SRPX RHOC SLC25A1 AKR1C3 GDF15 TCEA1 SCNN1A COL6A1

STAUNTON_376254 na HMGA1 GSTP1 KRT18 TUBA3 IDH2 MT1H FN1 PHB PLOD1 ANXA5 PRDX1 HLA-A ITPK1 PGD HMGA2 CTSD CD99 H2AFX EIF3S9 SNRPC TM4SF1 NNMT PYGB SREBF1 IFITM3 /// IFITM2 TGFBI HLA-F MAGEA3 TAX1BP3 CAV1 KPNA2 CDKN2A POLR2F ANXA1 MCAM KRT8 TPM1 COMT COL1A1 DDT NFKBIA MFGE8 ATP6V0B GNG11 CCND1 PMP22 TUBA1 UPP1 CXCL1 LGALS3 /// GALIG LGALS1 CTSC CD59 CXCL2 SLC39A6 IL8 AKR1B1 /// EIF3S9 HEXB ALDH1A1 PGM1 LITAF S100A10 CYBA NBL1 ACOT7 UBB MDK CAPG MAGEA2 /// MAGEA2B

STAUNTON_376266 na FLNA GSTP1 GLO1 TUBA3 PDHA1 XRCC6 ISG20 IRAK1 CAPN2 HLA-B /// HLA-C CD99 C1S TUBB3 SNRPC TM4SF1 NNMT PFKP JUND TST TMED9 EEF1A2 GPX1 TIMP2 TAX1BP1 CAV1 SERPINH1 AKR1A1 CST3 MCAM ATP6V0C TAGLN MT1E IGFBP2 S100A4 CRIP2 CYR61 TSPAN3 EMP3 MFGE8 ATIC ACTN1 PAFAH1B3 GADD45A CTSL HDAC1 PXDN ALDH1A3 POLR2B EEF1D LGALS1 ATP1B3 COL4A1 TMSB4X ID2B AKR1B1 /// EIF3S9 ZYX SULT1A3 /// SULT1A4 CSRP1 PSITPTE22 G6PD RAB13 MSN ARF4 GPI MT2A DHPS MT2A /// LOC441019 NBL1 PSMD6 PEA15 CKB MDK CAPG GALE NPC2 LOXL2 SLC6A8 KRT18 MT1H AARS ANXA2 ASS PIR S100A11 PLP2 HLA-A SORD COL4A2 PMM1 COX17 TUBG1 FSCN1 SERPINE1 CKMT1B CLU PRDX4 HPRT1 FTL CALD1 IFITM3 /// IFITM2 TGFBI CYC1 LOXL1 ITGB5 IGFBP5 KRT8 IL32 TPM2 TPM1 B2M DSTN PLAUR DHCR24 MYH9 IER3 GNAI2 UQCRH CYB5R3 IGFBP3 OAT SNRPN /// SNURF GUK1 GNG11 NPPB HSPA1A BLVRB SIAHBP1 DXS9879E TXNIP FADD SDHA RHOC PLD3 PRDX2 SLC25A1 GDF15 CD9 DDX48 LITAF COL6A1

STAUNTON_382034 na LOXL2 MFGE8 MYH9 TALDO1 MT1H FN1 IGFBP3 ANXA5 GADD45A PRDX1 SCARB1 COL4A2 FSCN1 ETFB TKT CD59 CTSC CTGF ID3 MGST1 NNMT UGCG COL5A2 KIAA0251 COX5B GGH NR0B1 TIMP2 PRSS11 CD9 MT2A SPP1 MCAM MT2A /// LOC441019 LITAF CAPG

STAUNTON_382035 na CYR61 COL1A1 ECH1 STIP1 SPARC IGFBP3 GADD45A CCND1 HLA-A COL4A2 PXDN SERPINE1 FSTL1 COL4A1 CTGF NNMT HLA-F TAX1BP1 PSAP SERPINH1 PGM1 SFN MATN2 TPM1 COL6A1 MDK TFPI2

STAUNTON_382046 na CTGF NT5E HLA-A CST3 HLA-F C1S PTTG1IP

STAUNTON_600032 na FLNA FHL2 GSTP1 APOE GLO1 RPN2 NOMO1 /// NOMO2 /// CTSD CAPN2 MYL9 HSPD1 HLA-B /// HLA-C CD99 TYMS H2AFX MYC SHFM1 MGST1 NNMT CRIP1 VCL TAX1BP3 EEF1A2 GPX1 CAV1 CST3 TAGLN IGFBP2 PHB2 EMP3 JUNB MFGE8 TPM4 COX6C CLNS1A /// C3ORF4 CCND1 SLC7A5 TAGLN2 LGALS3 /// GALIG LGALS1 HMGB2 HSPE1 TMSB4X AKR1B1 /// EIF3S9 ZYX PLOD2 CSRP1 STAT1 PYCR1 ARF4 MT2A MT2A /// LOC441019 S100A10 ODC1 PEA15 MDK TFPI2 CAPG GLG1 PABPC4 MT1H FN1 HADHA ANXA5 ITGB1 FBL PLP2 HLA-A COL4A2 LAMP1 FSCN1 CD24 TUBG1 PPAP2B BCL3 CLU S100A13 SREBF1 IFITM3 /// IFITM2 CALD1 SLC39A14 IFITM1 ESD IGFBP5 IL32 TPM1 B2M COMT DSTN TOMM20 COL1A1 DDT PLEKHC1 DHCR24 ATP5A1 IGFBP4 PTTG1IP ATP6V0B IGFBP3 VIM SNRPN /// SNURF GUK1 GNG11 IFITM3 /// IFITM2 // C12ORF8 FUCA1 CD59 KRT7 PKM2 SLC39A6 RHOC PLSCR1 LASP1 SLC25A1 CD9 PLEC1 LITAF ATP5D

STAUNTON_600391 na COMT DDT LRPAP1 EMP3 KRT18 TMED10 FHL1 PLOD1 IGFBP3 COL4A2 ACAT1 IFITM3 /// IFITM2 // GPC1 NQO1 CTSD PMP22 MYL9 HLA-B /// HLA-C TUBA1 LGALS3 /// GALIG UCHL1 SHFM1 TUBB3 CD59 BOP1 NNMT PFKP TGFBI AKR1B1 /// EIF3S9 HEXB MT1E MDK

STAUNTON_602022 na CAST BZRP NME4 LGALS3BP ZNF91 CD63 COX6C SPINT2

STAUNTON_603071 na DCI CSDA GSTP1 TUBA3 FNTA SPARC PHB CCNH UFD1L LGALS3BP RHOD ARF3 MYL9 CAPN2 ITPK1 HLA-B /// HLA-C CD99 H2AFX LGMN MYC TUBB3 MGST1 ID3 NNMT VCL GPX4 TAX1BP3 MAGEA3 GPX1 SERPINH1 AKR1A1 MAGEA12 CST3 ANXA1 MCAM MT1E PSMB4 UBE2S S100A4 NEDD5 MYH10 PPP1CC MFGE8 TPM4 GAL FKBP1A CTSL PMP22 SLC7A5 LGALS3 /// GALIG MGLL ATP1B3 LGALS1 HMGB2 MCM7 IL8 M-RIP CAP1 ID2B AKR1B1 /// EIF3S9 INHBA MSN MT2A /// LOC441019 MLF2 NBL1 MDK CAPG NPC2 DRAP1 PON2 FOSL1 SLC6A8 KRT18 IDH2 MT1H ASS FN1 PLOD1 FBL PLP2 HLA-A COL4A2 FSCN1 SERPINE1 CDKN3 PRDX4 LEPROT S100A13 IFITM3 /// IFITM2 TGFBI CYC1 ITGB5 KRT8 IL32 RRAS TPM2 PRG1 DDX11 TPM1 DSTN COMT CIB1 DDT DHCR24 MYH9 NDUFV3 CYB5R3 ATP6V0B IGFBP3 IFITM3 /// IFITM2 // UPP1 BLVRB SNRPB2 C12ORF8 CXCL1 CDC20 CD59 DXS9879E SLC39A6 FAM38A RHOC LASP1 PRDX2 UBE2C STMN1 PGM1 GDF15 CD9 LITAF CYBA MAGEA1

STAUNTON_603553 na FLNA COL1A1 CRABP2 PLAUR LOXL2 GSTP1 IGFBP3 ACTN1 PAFAH1B3 SORD PDIA3 SERPINE1 LGALS1 DXS9879E NDRG1 CRIP1 JUP KIAA0251 ECHS1 TMSB4X TGFBI ZYX MSN TFRC SEMA3C COL6A1 POLR2G MDK PHLDA2

STAUNTON_606307 na CRABP2 SNRPN /// SNURF PSMB10 TAGLN2 CKMT1B CLU POLR2J SIAHBP1 HADH2 TFF3 DXS9879E SSR4 HSPB1 JUP NIFUN IFITM1 ZYX ATP6AP1 PSITPTE22 G6PD G1P2 NT5E IGFBP5 ATP5G2 MT1E IGFBP2

STAUNTON_606497 na FLNA LOXL2 GSTP1 FNTA MT1H IDH2 FN1 ILK HLA-A COL4A2 YWHAH FSCN1 ITPK1 ETFB IQGAP1 CTGF ID3 SNRPC NNMT PFKP COL5A2 IFITM3 /// IFITM2 HLA-F SPINT2 NDUFS8 EEF1A2 TIMP2 SERPINH1 AKR1A1 ITGB5 MCAM ATP6V0C MT1E TPM1 UBE2S CIB1 MFGE8 MYH9 DUT TPM4 UQCRH PTMS ATP6V0B CYB5R3 IGFBP3 GADD45A GNG11 CCND1 IFITM3 /// IFITM2 // SLC7A5 POLR2J MGLL FUCA1 CD59 TXNIP SLC39A6 FAM38A M-RIP EBNA1BP2 CAP1 ARF5 ALDH1A1 ZYX RHOC AKR1B1 /// EIF3S9 LASP1 CSRP1 STAT1 PGM1 CD9 ARF4 MT2A /// LOC441019 LITAF SFRS3 CYBA NBL1 UBB CAPG

STAUNTON_606499 na PPIF HMGA1 NPC2 PLAUR GSTP1 MFGE8 SLC6A8 SQSTM1 TUBA3 S100A2 ATP6V0B PRDX1 RARS CTSL LGALS3BP CTPS IFITM3 /// IFITM2 // ALDH3B1 HLA-B /// HLA-C SHC1 CDKN3 POLR2J EXOSC7 ID3 NNMT SSR4 UGCG CRIP1 HMGN1 PFKP FABP5 TMSB4X ANXA11 HLA-F AKR1B1 /// EIF3S9 RHOC CSRP1 MRCL3 CAV1 UCP2 TCEA1 LTB4DH MT1E LITAF TPM1 UBE1

STAUNTON_606698 na TIMP2 NPC2 CTGF CRABP2 ALDH2 HLA-F MDK PRDX2 TFPI2

STAUNTON_606699 na COMT TSPAN3 PLEKHC1 NME4 KRT18 TPM4 LAMB1 IDH2 NPIP /// LOC23117 // ITGB1 APLP2 LGALS3BP HSPA1A MVP CD99 CD81 SERPINE1 BLVRB DUSP1 KYNU DXS9879E SNRPC TM4SF1 CRYAB MGST1 COL6A2 JUND IFITM3 /// IFITM2 CALD1 ID2B CYC1 ZYX GAS6 ACLY RAB13 ANXA1 KRT8 RRAS TPM2 TAGLN XBP1 SEPW1 B2M TFPI2 CAPG

STAUNTON_608984 na PGK1 STIP1 MT1H PDHA1 FN1 FBL HLA-A COX17 CD24 HSPD1 CLU ETFB MYC KYNU GNB2 BOP1 TFF3 HLA-F CYC1 EMD GPX1 CARS TFF1 NT5E SMS MCAM MT1E C10ORF116 FARSLA ANP32B CRABP2 MFGE8 ECH1 TNNC1 SLC25A5 PXN PCBD1 COX6C IGFBP3 OAT HSF1 HSPA1A IFITM3 /// IFITM2 // MORF4L2 BIRC5 C12ORF8 POLR2J MCM7 NDRG1 DXS9879E NIFUN CYP11B1 /// CYP11B2 ALDH1A1 PRDX2 COX7B MT2A SFN MT2A /// LOC441019 SLC4A2 CYBA ASNS MDK

STAUNTON_609699 na NPC2 DDT FKBP1A PRKAR1A IGFBP3 OAT CCND1 CTSL SHC1 LGMN CLU LGALS3 /// GALIG CXCL1 S100A13 IFITM3 /// IFITM2 PCNA RHOC G6PD PGM1 ITGB5 MCAM LITAF S100A10 TPM1 CAPG

STAUNTON_610457 na MAGEA4 COMT DDT MT1H FN1 IGFBP3 ANXA5 CCND1 COL4A2 FSCN1 YWHAH IQGAP1 FUCA1 ID3 EIF5A /// LOC143243 IFITM3 /// IFITM2 HLA-F CAP1 STAT1 G6PD GDF15 CD9 SPP1 MCAM MT2A /// LOC441019 LITAF CYBA NBL1 UBB MAGEA2 /// MAGEA2B MAGEA1 CAPG

STAUNTON_610459 na DCI FLNA GSTP1 LOXL2 IDI1 TUBA3 TIMP3 IDH2 FN1 FBL FSCN1 ITPK1 CD99 SERPINE1 CLU IQGAP1 SNRPC ID3 NNMT RBBP7 COL5A2 IFITM3 /// IFITM2 COX5B NDUFS8 EEF1A2 TIMP2 SERPINH1 AKR1A1 ITGB5 MCAM KRT8 UBE2S PHLDA2 CIB1 PLAUR MFGE8 EMP3 DUT PTMS CYB5R3 ACTN1 IGFBP3 ATP6V0B GADD45A GNG11 IFITM3 /// IFITM2 // POLR2J MGLL FUCA1 ATP1B3 PRNP TXNIP SLC39A6 TMSB4X FAM38A CAP1 ALDH1A1 RHOC CSRP1 CD9 ARF4 MT2A MT2A /// LOC441019 LITAF CYBA COL6A1

STAUNTON_615441 na GSTP1 CLTC APOE TUBA3 PPGB SPARC NARS APLP2 LGALS3BP RHOD ARF3 CAPN2 CTSD MYL9 PGD ITPK1 HLA-B /// HLA-C CIRBP CLTA CD99 HCCS TUBB3 TM4SF1 MGST1 ID3 NNMT SOD2 VCL GPX4 TAX1BP3 EEF1A2 GPX1 ATP5G3 SERPINH1 CAV1 AKR1A1 SMS ANXA1 CST3 MCAM MT1E CYR61 EMP3 ECH1 PRKAR1A FKBP1A CCND1 CTSL PXDN PMP22 TUBA1 TAGLN2 LGALS3 /// GALIG LGALS1 MCM7 C1QBP IL8 ECM1 GDI2 CAP1 AKR1B1 /// EIF3S9 PSITPTE22 MSN SEMA3C MT2A MT2A /// LOC441019 S100A10 NBL1 ACOT7 UBB MDK CAPG HMGA1 NPC2 ALCAM UBE2L3 PON2 DRAP1 KRT18 KRT19 MT1H ASS FN1 ITGB1 ANXA5 HLA-A COL4A2 COX17 CD24 FSCN1 SERPINE1 CLU CTGF S100A13 UGCG DNAJA1 SREBF1 PAX8 IFITM3 /// IFITM2 FABP5 TGFBI HLA-F SPINT2 PTDSS1 CDKN2A IL32 KRT8 TPM2 TPM1 B2M DSTN CIB1 COMT COL1A1 DDT DHCR24 NFKBIA IGFBP4 NDUFV3 SDHB IGFBP3 ATP6V0B SNRPN /// SNURF IFITM3 /// IFITM2 // CTPS DUSP1 CXCL1 KRT7 SLC39A6 FAM38A PCNA PLD3 RHOC PRDX2 GAS6 GDF15 PGM1 CD9 SFN LITAF CYBA MAGEA2 /// MAGEA2B

STAUNTON_620256 na ATP6V0B CTGF ID3 CBX1 RAC1 MT1E TPM1 MDK

STAUNTON_623520 na AKR1C3 NPC2 HSPB1 IDI1 CDKN2A MT1E IGFBP2 SRPX PAM PLOD1

STAUNTON_623794 na CYR61 CRIP1 MFGE8 CD24 CYBA S100A4 LGALS3 /// GALIG

STAUNTON_625492 na COMT GSTP1 IGFBP4 S100A11 GOT2 S100A2 ACTN1 ANXA5 F3 PAFAH1B3 KLF5 HLA-A LGALS3BP HLA-B /// HLA-C LGALS1 DXS9879E S100A13 SREBF1 CRIP1 MRPS12 TGFBI ID2B SNRPB G6PD PSITPTE22 C20ORF24 CD9 LTB4DH MDK B2M

STAUNTON_625498 na MGLL MGST1 DHCR24 HSPA1A LITAF TGFBI MDK

STAUNTON_625499 na COMT CIB1 NPC2 TNNC1 IGFBP4 IDH2 ANXA5 LGALS3BP TM4SF1 MGST1 PKM2 JUP TGFBI SNRPB CSRP1 PSITPTE22 CDKN2A KRT8 LTB4DH TPM2 MT1E TPM1 ABLIM1 MDK ATP5D

STAUNTON_627505 na C5ORF13 LGALS1 COMT CTSC HMGA1 MGST1 PON2 ID2B SPARC SPINT2 UQCRH IGFBP3 SORD TMSB10 MYL9 CYBA CD99 TAGLN2 S100A4

STAUNTON_629971 na CSDA DCI KRT18 IDH2 ANXA2 FN1 FHL1 PHB PLOD1 CTSD CAPN2 ITPK1 HLA-B /// HLA-C CD99 LGMN SERPINE1 EIF3S9 LEPROT CTGF SNRPC NNMT HPRT1 CALD1 CYC1 TAX1BP3 HSPA4 AKR1A1 ITGB5 KRT8 MCAM RRAS MCL1 MT1E TPM1 PSMB4 CIB1 COMT COL1A1 MFGE8 DUT SLC25A5 CD151 NDUFV3 CYB5R3 ATP6V0B IGFBP3 GNG11 NQO1 PXDN PMP22 UPP1 CXCL1 LGALS3 /// GALIG MGLL ATP1B3 CD59 SLC39A6 CXCL2 IL8 EBNA1BP2 CAP1 AKR1B1 /// EIF3S9 RHOC INHBA PGM1 GDF15 LITAF CYBA COL6A1 MDK CAPG

STAUNTON_630307 na COMT NPC2 MYH9 CELP TPM4 GNAI2 IFITM3 /// IFITM2 MT1H HLA-F FLII CPNE1 PRDX2 CSRP1 TIMP2 IGFBP3

STAUNTON_633713 na HMGA1 NPC2 GSTP1 NME4 KRT18 IFI30 TUBA3 KRT19 ADA TMED10 ANXA2 FAM50A HLA-A LGALS3BP COL4A2 CTSD HLA-B /// HLA-C KIAA0114 SERPINE1 LGMN CD74 IQGAP1 TUBB3 HLA-DRA HK1 CTGF CRYAB JUP FABP5 RANBP1 HLA-F TGFBI SPINT2 EEF1A2 TAX1BP1 CKAP4 PSAP PTPRF ANXA1 CST3 KRT8 MT1E TPM1 S100A4 CETN2 B2M CYR61 DSTN COL1A1 TSPAN3 MYH9 GARS SQSTM1 ECH1 IGFBP4 PFN2 PTMS PTTG1IP IGFBP3 SNRPN /// SNURF GADD45A CCND1 HSPA1A IFITM3 /// IFITM2 // NQO1 TUBA1 CSTB TAGLN2 CXCL1 MGLL SKB1 CTSC COL4A1 KRT7 TXNIP SSR4 TMSB4X TACSTD1 LASP1 CSRP1 GAS6 PPIB G6PD MSN PGM1 CD9 MATN2 LITAF CYBA S100A10

STAUNTON_634724 na CCND1 DHCR24 SPP1 EIF5A /// LOC143243 MT1E TGFBI TPM1 UPP1 CAPG

STAUNTON_635448 na ALCAM FHL2 LOXL2 KRT18 VIL2 TUBA3 KRT19 MT1H IDH2 SPARC HLA-A COL4A2 AHNAK ITPK1 CTSD SERPINE1 CLU TUBB3 S100A13 SNRPC HSPB1 NNMT IFITM3 /// IFITM2 TGFBI GPX4 EEF1A2 IGFBP6 TAX1BP1 CAV1 DDX5 AKR1A1 SAP18 CDKN2A AP3S1 ANXA1 IL32 KRT8 MT1E TPM1 S100A4 B2M CIB1 DSTN COL1A1 MYH9 MFGE8 ECH1 FKBP1A IGFBP3 PAFAH1B3 GADD45A KLF5 HSPA1A IFITM3 /// IFITM2 // CSTB LGALS3 /// GALIG FSTL1 MGLL LGALS1 COL4A1 KRT7 TXNIP PKM2 DIPA TMSB4X FAM38A ID2B ARF5 AKR1B1 /// EIF3S9 PRDX2 SLC25A1 GAS6 PSITPTE22 MT2A MT2A /// LOC441019 NBL1 ACOT7 MDK

STAUNTON_636092 na CYR61 COL1A1 HMGA1 CAPZA1 IGFBP3 PRDX1 HNRPH1 CCND1 CTSD HLA-B /// HLA-C BAT3 ETFB SIAHBP1 BOP1 CTGF DXS9879E SNRPC ID3 SSR4 GRN FABP5 HLA-F ZYX PRDX2 NEDD8 GPI CDKN2A CAPG

STAUNTON_637140 na GSTP1 CLTC TUBA3 PPGB SPARC PRDX1 CEBPD APLP2 LGALS3BP RHOD ARF3 ITPK1 MYL9 HLA-B /// HLA-C CLTA CD99 UCHL1 KYNU MGST1 TM4SF1 ID3 NNMT SOD2 VCL GGH GPX4 TAX1BP3 EEF1A2 GPX1 ATP5G3 CAV1 SERPINH1 AKR1A1 TEGT SMS CST3 ANXA1 MCAM MT1E POLD4 CYR61 CCNG1 MFGE8 ECH1 TPM4 PRKAR1A CCND1 PRAME CTSL HOXB2 TUBA1 TAGLN2 MTHFD1 FSTL1 LGALS3 /// GALIG NR4A1 RAB2 LGALS1 MCM7 TI-227H KIAA0251 IL8 TMSB4X GDI2 CAP1 AKR1B1 /// EIF3S9 PSITPTE22 MSN GPI MT2A MT2A /// LOC441019 PSMD1 NBL1 MDK CAPG ALCAM NPC2 HMGA1 UBE2L3 QSCN6 PON2 KRT18 MT1H IDH2 FN1 ENO1 ITGB1 HLA-A SORD COL4A2 LAMP1 COX17 FSCN1 SERPINE1 PRDX4 CTGF S100A13 UGCG BZW1 /// LOC151579 DNAJA1 SREBF1 PAX8 IFITM3 /// IFITM2 FTL FABP5 TGFBI SPINT2 ARPC1B PSAP PTDSS1 G1P2 CDKN2A RRAS ATP5G2 IL32 TPM2 DDX11 TPM1 B2M COMT CIB1 COL1A1 DDT DHCR24 NFKBIA LRPAP1 C1ORF16 IGFBP4 NDUFV3 PTTG1IP MGST2 IGFBP3 ATP6V0B S100A2 IFITM3 /// IFITM2 // SHC1 BLVRB CXCL1 DUSP1 DXS9879E SSR4 SLC39A6 FAM38A XRCC5 RHOC LASP1 PRDX2 GAS6 ACP1 GDF15 PGM1 CD9 SFN TCEA1 LITAF CYBA MAGEA2 /// MAGEA2B

STAUNTON_637651 na DCI COL1A1 FOSL1 MFGE8 PAX8 VIL2 ID2B HLA-F SPARC SPINT2 AKR1B1 /// EIF3S9 LASP1 IGFBP3 S100A2 GUK1 TFRC COL4A2 MYL9 CD99

STAUNTON_639831 na MAPK6 LRRC14 FLNA BZRP FHL2 GSTP1 NME4 UQCRFS1 TUBA3 FNTA SFRS11 TALDO1 SPARC USP11 RPL36AL DDX39 NOMO1 /// NOMO2 /// PRDX1 PSMA6 LGALS3BP YWHAH ARF3 MYL9 PGD ITPK1 HLA-B /// HLA-C CTNNB1 SEPT7 UCHL1 IQGAP1 HK1 SNRPC TM4SF1 ID3 NNMT SRI PFKP TST VCL GPX4 GGH TAX1BP3 TAX1BP1 SPCS2 CAV1 SERPINH1 CST3 MCAM POLD4 MT1E UBE2S CRIP2 CYR61 TXN MFGE8 SQSTM1 POLR2H TPM4 FKBP1A PRAME KIAA0152 ACAT1 PSMB10 TUBA1 SLC7A5 NCSTN TAGLN2 MTHFD1 LGALS3 /// GALIG MGLL LGALS1 HSPE1 DIPA KIAA0251 PTP4A2 ID2B HEXB ZYX AKR1B1 /// EIF3S9 ARF5 SFRS10 MSN SEC61B GPI SFRS3 SEPW1 UBB MDK CAPG TFPI2 PCDHGC3 /// PCDHGB4 ALCAM HMGA1 UBE2L3 PON2 STIP1 IDH2 TMED10 FN1 ENO1 ANXA5 HAX1 HLA-A COL4A2 LAMP1 FSCN1 BAT3 SERPINE1 CLU PRDX4 ACO2 RBBP7 GRN CDC25B FTL HLA-F TGFBI SPINT2 ARPC1B TFRC CDKN2A IL32 RRAS GNB1 TPM1 SLC3A2 B2M DSTN CIB1 COMT COL1A1 DDT PLAUR DHCR24 LRPAP1 NFKBIA PSMA4 MYH9 C1ORF16 IER3 UQCRH ARPC1A CYB5R3 ATP6V0B S100A2 IGFBP3 SNRPN /// SNURF MSLN IFITM3 /// IFITM2 // UPP1 DUSP1 POLR2J SIAHBP1 LOC23117 /// DKFZP54 LMNA CD59 DXS9879E EBP TXNIP SLC39A6 CXCL2 EIF2B2 FAM38A XRCC5 EBNA1BP2 SRP14 SRPX LASP1 PGM1 GDF15 CD9 SFN PLEC1 PSMA3 LITAF ANXA4 CYBA UBE1

STAUNTON_641296 na HMGA1 NME4 LTA4H IDH2 FN1 MIF HMGN3 LGALS3BP CD24 MYL9 CTSD TOP2A CIRBP TYMS UCHL1 CD74 IQGAP1 CPSF1 TM4SF1 ID3 CRYAB S100A13 JUP PFKP TST CALD1 SND1 IFITM1 ADK CAV1 TACSTD2 IGFBP5 ATP5G2 TAGLN C10ORF116 IGFBP2 CIB1 TSPAN3 COL1A1 RPL39 GSTO1 CRABP2 RPS4Y1 SQSTM1 GNAI2 TNNC1 FTH1 S100A2 VIM SNRPN /// SNURF NQO1 SIAHBP1 DXS9879E C1QBP DIPA TMSB4X ID2B ZYX HEXB AKR1B1 /// EIF3S9 PSITPTE22 GDF15 PSMD1 XBP1 ODC1

STAUNTON_641297 na NPC2 MYH10 PON2 EMP3 MFGE8 KRT18 ARHGDIB SPARC PLOD1 S100A2 CTSL HLA-A HDAC1 NQO1 ALDH1A3 LGALS1 PSMC2 SHFM1 ID2B CYC1 ZYX CAV1 PSITPTE22 PSAP CST3 S100A4

STAUNTON_643248 na ANP32B MFGE8 TPM4 TUBA3 IDH2 NDUFV3 FN1 ARPC1A CCND1 HMGA2 POLR2J C5ORF13 CD47 GNB2 DXS9879E CTGF ID3 HSPB1 COL6A2 COPS6 SULT1A3 /// SULT1A4 ARF5 PRDX2 RAB13 SFN CYBA IFI16 MDK CAPG

STAUNTON_644211 na CYR61 NPC2 COL1A1 GNAI2 FN1 S100A2 PAFAH1B3 PLP2 HLA-A NQO1 PXDN SERPINE1 UCHL1 HK1 CCT2 ID3 SNRPC PFKP HLA-F TGFBI PRDX2 GPX1 SPCS2 MRPL49 MT2A /// LOC441019 MT1E ODC1

STAUNTON_644945 na ANP32B NFKBIA TUBA3 IFI30 PABPC4 IDH2 FN1 PSMD2 FBL FKBP2 LGMN CLU MGLL SIAHBP1 TXNIP CRIP1 GRN DIPA COPS6 HLA-F SPINT2 LASP1 AKR1A1 CD9 G1P2 MT2A MT2A /// LOC441019 TCEA1 TPM1

STAUNTON_645017 na DDT UBE2L3 MFGE8 EBNA1BP2 MAGEA3 TIMP2 ATP6V0B GYPC OAT PSAP MAGEA12 PRAME CD9 CDKN2A UCP2 LITAF CRIP2

STAUNTON_645018 na MFGE8 KRT19 FNTA MT1H GNG11 CCND1 RHOD LGALS3BP ARF3 PGD TUBA1 LGALS3 /// GALIG CTSC HLA-F EBNA1BP2 TGFBI SPINT2 ALDH1A1 PGM1 POLR2F SFN LITAF CYBA AES UBB MDK

STAUNTON_645665 na PON2 KRT18 PDHA1 CSK SORD CBX1 HLA-B /// HLA-C TYMS SERPINE1 UCHL1 ETFB HADH2 CTGF SNRPC UGCG SNAPC1 EIF5A /// LOC143243 PFKP GRN IFITM3 /// IFITM2 HLA-F EEF1A2 CAV1 CDKN2A CST3 FARSLA ALG3 CYR61 DSTN CAPZA1 LRPAP1 HNRPA1 GNAI2 PFN2 PTTG1IP MSLN HNRPH1 CCND1 RARS IFITM3 /// IFITM2 // LGALS3 /// GALIG MGLL KRT7 NDRG1 TXNIP ALDH1A1 PRDX2 COX7B PPIB GDF15 GPI PSMA3 MDK

STAUNTON_645668 na DCI RPS3 CKS1B GLO1 FLII EXT1 PSMA6 PGD CD99 MYC KYNU HADH2 SNRPC CRIP1 PFKP COL5A2 TAX1BP3 NDUFS8 MAGEA3 EEF1A2 TAX1BP1 CST3 MCAM MT1E S100A4 SQLE PHLDA2 CRIP2 CYR61 CRABP2 EMP3 DUT SQSTM1 ARHGDIB PAFAH1B3 CCND1 RARS PXDN SLC7A5 ALDH1A3 LGALS3 /// GALIG MGLL TKT HMGB2 DIPA TMSB4X ID2B ZYX AKR1B1 /// EIF3S9 CSRP1 PYCR1 GPI MT2A /// LOC441019 CKB MDK PPIF ALCAM LOXL2 KRT18 MT1H PRDX6 ANXA2 PLOD1 HLA-A COL4A2 BECN1 TUBG1 BAT3 SERPINE1 CLU PRDX4 EXOSC7 BOP1 HPRT1 SREBF1 GRN HLA-F TGFBI SPINT2 DCTD TFRC SERPINA1 CDKN2A KRT8 CDA TPM1 COL1A1 ANP32B GSTO1 MYH9 NFKBIA RPS4Y1 HNRPA1 GNAI2 H1FX PTTG1IP IGFBP3 S100A2 OAT SNRPN /// SNURF MAPRE1 MMP1 HNRPH1 IFITM3 /// IFITM2 // NQO1 SHC1 UPP1 HLA-DRB1 NDRG1 TXNIP ANXA11 ECHS1 PLCD1 AKR1C1 SEC24C PAICS GDF15 SFN RDBP UROD COL6A1

STAUNTON_649677 na GSTP1 MFGE8 LRPAP1 KRT18 ANXA5 HLA-A CTSL LGALS3BP PGD SLC7A5 ATP6V1F TM4SF1 NNMT CDC25B HLA-F TGFBI ZYX LASP1 CAV1 GDF15 CD9 MCAM MT1E CYBA TPM1 NBL1 MDK CAPG TFPI2

STAUNTON_651694 na NME4 TALDO1 EXT1 RAD21 EEF1A1 CEBPD APLP2 LGALS3BP HLA-B /// HLA-C SEPT7 RPL24 /// SLC36A2 COX5A MYC SRI ACY1 SOD2 TMED9 SUMO2 SERPINH1 CAV1 SMS MCAM PHLDA2 CYR61 MFGE8 ADSL ACTN1 CTSL LGALS3 /// GALIG CSNK1E SKB1 TKT HSPE1 CTSC KIAA0251 TMSB4X TSC2 ID2B SULT1A3 /// SULT1A4 AKR1B1 /// EIF3S9 COX7B SFRS10 HNRPL GPI MT2A MT2A /// LOC441019 TK1 RAE1 MDK CAPG ALCAM HMGA1 QSCN6 LOXL2 HSPH1 MT1H PCCB FAM50A FN1 PIR ANXA5 CCT6A COL4A2 TUBG1 FSCN1 CLU PRDX4 ETFB UGCG CDC25B HLA-F ATP5O NT5E SPP1 IMMT TPM1 P4HA1 DDT COL1A1 PLAUR ATP5A1 GNAI2 SLC25A5 FTH1 IGFBP3 S100A2 MTHFS MRPL23 LDHB UPP1 SIAHBP1 CD59 KRT7 EWSR1 HNRPM PRDX2 PAICS PSMA2 GDF15 PLEC1 LITAF SLC4A2 UBE1 ATP5D BRD2

STAUNTON_652176 na FLNA BZRP LOXL2 CKS2 LTA4H SPARC JTV1 FN1 TMED10 CPNE1 SCARB1 SORD HLA-A COL4A2 AHNAK HMGA2 CD99 NMU KIAA0114 SSBP1 PRDX4 ETFB PDLIM1 CTGF KHSRP UGCG HSPB1 SNAPC1 PSME1 EIF5A /// LOC143243 SND1 HLA-F SPINT2 SNRPB TOP1 PSAP CAV1 SERPINH1 G1P2 CDKN2A SPP1 MCAM TPM1 PHLDA2 SLC3A2 OS9 CYR61 COMT EIF4B NFKBIA MYH9 GNAI2 PDXK CD151 UQCRH YWHAE H1FX ALDOA LAMC1 IGFBP3 S100A2 ACTN1 CCND1 IFITM3 /// IFITM2 // SLC7A5 CSTB SNRPB2 MGLL PSMC2 KRT7 CD59 DXS9879E NDRG1 DIPA TMSB4X GDI2 M-RIP PCNA CYP11B1 /// CYP11B2 ALDH1A1 PRDX2 STAT1 PSITPTE22 RAB13 CD9 MT2A SFN RDBP S100A10 MDK CAPG

STAUNTON_652287 na MAPK6 DCI NPC2 GSTP1 GLO1 KRT19 JTV1 IL13RA2 BTG3 UQCRC2 ARF3 PGD CLU ETFB MGST1 SNRPC HSPB1 IFITM3 /// IFITM2 FABP5 TGFBI TMED9 CYC1 EEF1A2 PSMD4 CAV1 DDX5 ESD SAP18 SPP1 MCAM TPM2 PRG1 IGFBP2 CIB1 MYH9 MFGE8 GARS PFN2 UQCRH PRKAR1A PTTG1IP IGFBP3 S100A2 CCND1 HSPA1A IFITM3 /// IFITM2 // PDIA3 TUBA1 SLC7A5 POLR2B BLVRB CCT2 HMGN1 SLC39A6 TMSB4X NDUFA4 ARMET SRPX LASP1 ACLY AKR1C3 PYCR1 ARF4 MT2A /// LOC441019 CYBA CAPG

STAUNTON_654830 na CYR61 NPC2 HMGA1 MFGE8 TUBA3 IDH2 MT1H FAM50A ASS FN1 PLOD1 ISG20 TTC3 HLA-A LGALS3BP FSCN1 CTSD CSTB COL11A1 LGMN SERPINE1 LGALS3 /// GALIG CTGF TXNIP SSR4 BST2 COL5A2 HLA-F SRPX EEF1A2 PSAP CAV1 PRSS11 CD9 G1P2 CDKN2A CST3 MT1E IFI16 B2M ALG3

STAUNTON_657822 na GALE NPC2 KRT19 FXYD2 TALDO1 SOX4 JTV1 S100A11 RHOD LGALS3BP NDUFA12 CTSD PGD SDC4 HLA-B /// HLA-C PRDX4 TUBB3 MGST1 SNRPC RBBP7 JUND HLA-F SPINT2 CYC1 SNRPB SERPINH1 SNRPD3 SPP1 MCAM MT1E POLR2G PHLDA2 B2M CYR61 DSTN COMT MYH10 EMP3 MFGE8 ECH1 PSMC3 IER3 ARHGDIB UQCRH ALDOA S100A2 IGFBP3 KIAA0152 HDAC1 LDHB NQO1 SLC7A5 COL11A1 UPP1 BLVRB TKT LGALS1 MLLT11 CTSC BCAP31 KIAA0251 RBP1 SULT1A3 /// SULT1A4 AKR1C1 AP2B1 FXYD3 G6PD AKR1C3 CD9 SEMA3C MT2A /// LOC441019 S100A10 CYBA ACOT7 LTBR MDK

STAUNTON_658247 na DRAP1 TXNIP FXYD2 GNAI2 MT1H HLA-F GUK1 NT5E HLA-A LGALS3BP LDHB HLA-B /// HLA-C CYBA COL6A1 LGALS3 /// GALIG CAPG TNK2

STAUNTON_659948 na UBE2L3 FHL2 PON2 GSTP1 NME4 KRT18 KRT19 MT1H IDH2 SPARC ASS CPNE1 PLOD1 HLA-A LGALS3BP ARF3 MYL9 HLA-B /// HLA-C CD99 UCHL1 TUBB3 SNRPC JUND VCL HLA-F TGFBI SPINT2 TAX1BP3 EEF1A2 ARPC1B CAV1 PTDSS1 MCAM MT1E TPM1 CETN2 B2M ALG3 COMT DDT COL1A1 EMP3 MYH9 MFGE8 GARS TPM4 PDXK UQCRH NDUFV3 IGFBP3 GNG11 CCND1 PRAME HSPA1A IFITM3 /// IFITM2 // TUBA1 LGALS3 /// GALIG TKT CTSC TXNIP FAM38A AKR1B1 /// EIF3S9 SRPX PRDX2 CSRP1 GDF15 PGM1 ARF4 CD9 MT2A /// LOC441019 CYBA TFPI2 CAPG

STAUNTON_663790 na HMGA1 NPC2 SLC6A8 TUBA3 PGK1 VIL2 PPGB ATP5J TPBG TMED10 UFD1L NOMO1 /// NOMO2 /// SCARB1 PLP2 PSMA6 CTSD MYL9 GCHFR CD99 CLU HADH2 CTGF RBBP7 FTL TMED9 CYC1 MAGEA3 IFITM1 PSAP MAGEA12 CST3 ANXA1 LTB4DH ACOT2 MT1E KDELR2 S100A4 B2M DSTN COMT DDT TXN MFGE8 DUT GARS IER3 BNIP3 ATP6V0B SSX2 GNG11 CCND1 HSPA1A IFITM3 /// IFITM2 // PDIA3 LGALS3 /// GALIG ATP1B3 CD59 SSR4 COPB2 FADD TMSB4X PCNA ALDH1A1 RHOC PPIB AKR1C3 PGM1 GPI MT2A /// LOC441019 LITAF CCND3 S100A10 RFC4 MDK MAGEA1

STAUNTON_663791 na HADH2 CAV1 GNG11 CTGF CRIP1 KRT19 CD99 MDK

STAUNTON_663792 na CYR61 CLTC SLC6A8 MFGE8 PGK1 IER3 PTTG1IP ACTN1 ATP6V0B UFD1L NOMO1 /// NOMO2 /// GNG11 PLP2 HLA-A HSPA1A CTSD PGD CD99 BLVRB LGALS3 /// GALIG LGALS1 CD59 CTGF SNRPC SSR4 CD7 CRIP1 HLA-F EBNA1BP2 SPINT2 RHOC ALDH1A1 PPIB AKR1C3 PGM1 GPI MT2A CST3 ANXA1 LTB4DH CCND3 KDELR2 XBP1 S100A4 MDK B2M CAPG

STAUNTON_664181 na RHEB LAMB1 SPARC FLII CEBPD SSR2 CTSD HSPA5 PGD HLA-B /// HLA-C TUBB3 TFF3 TM4SF1 NNMT CRIP1 PLAU JUP COL5A2 TMED9 ATP6AP1 CKAP4 SMS CST3 ATP6V0C MT1E IGFBP2 S100A4 P4HB CRABP2 TPM4 AP2M1 PCBD1 CCND1 PXDN FSTL1 RAB2 MGLL TKT PTP4A2 TMSB4X NIFUN PLOD2 ZYX APP AKR1B1 /// EIF3S9 PSITPTE22 XBP1 CAPG GLG1 LOXL2 QSCN6 H3F3A /// LOC440926 HSPH1 IDH2 PLOD1 CAST HLA-A LAMP1 CD24 SERPINE1 PRDX4 RBBP7 COL6A2 FABP5 IFITM3 /// IFITM2 HLA-F TGFBI PHC2 HNRPK ADK PDIA6 PGRMC1 TPM2 TPM1 P4HA1 CTNNA1 OS9 COMT COL1A1 GSTO1 NFKBIA HNRPA1 IGFBP4 PXN IGFBP3 HSF1 GNG11 MAPRE1 HSPA1A IFITM3 /// IFITM2 // LDHB UPP1 HLA-DRB1 PARP4 POLR2J TNK2 TSC22D1 LOC23117 /// DKFZP54 KRT7 DXS9879E SERPINB5 TXNIP SSR4 SURF1 SRP9 LASP1 AKR1C3 SFN COL6A1

STAUNTON_665076 na NPC2 ERH CLTC TALDO1 MT1H TMED10 PLOD1 HLA-A TXNRD1 FSCN1 HLA-B /// HLA-C HCCS KIAA0114 SERPINE1 SNRPC ID3 MGST1 S100A13 RBBP7 COL6A2 HLA-F TIMP2 MCAM TPM2 MT1E DDX11 DSTN PLAUR SQSTM1 IER3 FKBP1A CYB5R3 IGFBP3 OAT CCND1 NQO1 SHC1 TKT DXS9879E BCAP31 IL8 TMSB4X ALDH1A1 G6PD PGM1 MT2A /// LOC441019 COL6A1 ASNS MDK CAPG TFPI2

STAUNTON_665622 na CTGF FLNA NDRG1 COL1A1 LOXL2 NME4 GARS PFKP TGFBI ID2B SPARC GTF2I /// GTF2IP1 HNRPF IGFBP3 HLA-B /// HLA-C TPM1 COL6A1 S100A4 MDK

STAUNTON_666783 na RPS3 LOXL2 KRT18 KRT19 RHEB IDH2 ASS CCNH HLA-A LGALS3BP COL4A2 LAMP1 ITPK1 CTSD MYL9 PPAP2B LGMN C1S CTGF CRYAB HSPB1 NNMT JUND IFITM3 /// IFITM2 CALD1 VCL HLA-F LOXL1 TAX1BP1 ARPC1B PSAP SERPINH1 PGRMC1 AKR1A1 CDKN2A MCAM TAGLN MT1E IGFBP2 S100A4 CYR61 COMT COL1A1 PLEKHC1 PLAUR CRABP2 MFGE8 SQSTM1 CELP SLPI PTTG1IP IGFBP3 HRMT1L1 GADD45A MSLN CSTB TAGLN2 DUSP1 LGALS3 /// GALIG COL4A1 SSR4 ZYX CSRP1 SLC25A1 CD9 MATN2 PEA15 MDK

STAUNTON_666787 na CTGF COL4A2 VIL2 HLA-F

STAUNTON_667876 na LGALS1 COL4A1 ALCAM NNMT NME4 ECH1 FTL HLA-F SPARC IGFBP6 ALDOA STMN1 HLA-A COL4A2 NQO1 FSCN1 CTSD COL6A1 FSTL1 B2M

STAUNTON_668281 na EIF2S1 CYR61 NPC2 PLEKHC1 IGFBP4 ANXA2 ATP6V0B PLP2 CCND1 CTPS PPAP2B LGMN MLLT11 CTGF CRYAB FADD HRAS VCL EBNA1BP2 RHOC NDUFS8 TFAP2C SERPINH1 PGM1 GDF15 LITAF TSPAN7 ALG3

STAUNTON_668297 na CSDA ALCAM LOXL2 IFI30 MT1H IDH2 SPARC PLOD1 LGALS3BP CTSD CD99 MYC CTGF BOP1 CALD1 FTL COX5B HLA-F TAX1BP1 ARPC1B CAV1 PIN1 SERPINA1 ITGB5 CST3 ATP5G2 MCAM TPM1 MYH10 MFGE8 SQSTM1 ARHGDIB H1FX NPIP /// LOC23117 // ALDOA G0S2 S100A2 CYB5R3 OAT CTSL NQO1 PXDN BIRC5 LGALS3 /// GALIG POLR2J MGLL ATP1B3 CD59 TXNIP FAM38A HEXB PRDX2 CSRP1 PSITPTE22 NBL1

STAUNTON_668323 na CYR61 BZRP NPC2 ALCAM LOXL2 PLAUR LRPAP1 DUT MYH9 SPARC PTTG1IP S100A2 CLNS1A /// C3ORF4 COL4A2 IFITM3 /// IFITM2 // CD99 H2AFX RAB2 DXS9879E NDRG1 BOP1 MGST1 TM4SF1 NNMT HSPB1 TXNIP SREBF1 UBC ALDH2 FAM38A FTL NIFUN COX5B HEXB PRDX2 CKAP4 DDX5 ARF4 CDKN2A KIAA0101 LTB4DH ATP5G2 TAGLN SLC4A2 TPM1 PHLDA2

STAUNTON_668324 na PFKM PPIF NPC2 LOXL2 PON2 CKS1B SLC6A8 IDI1 TUBA3 TIMP3 IDH2 PRDX6 SPARC PLOD1 ITGB1 COL4A2 LGALS3BP MYL9 HLA-B /// HLA-C H2AFX PRDX4 TUBB3 EXOSC7 CTGF MGST1 SNRPC NNMT HSPB1 CALD1 FABP5 COX5B HLA-F TGFBI SPINT2 TAX1BP3 EEF1A2 EMD TIMP2 SERPINH1 CAV1 PTDSS1 KPNA2 ANXA1 MCAM TPM2 MT1E TPM1 S100A4 SQLE PHLDA2 DHCR24 PLAUR TPM4 PDXK PFN2 YWHAE PTMS GOT2 ATP6V0B IGFBP3 CYB5R3 S100A2 CST6 NPPB GNG11 CTSL ACADVL HSPA1A IFITM3 /// IFITM2 // CTPS PMP22 PXDN POLR2J MGLL KRT7 COL4A1 DXS9879E NDRG1 TXNIP ANXA11 ID2B ZYX PRDX2 CSRP1 COX7B RAB13 PPIB SLC4A2 SEPW1 COL6A1 LDLR

STAUNTON_668325 na PFKM NPC2 FLNA GSTP1 SLC6A8 KRT18 TUBA3 IDH2 FLII S100A11 PLOD1 ITGB1 APLP2 PLP2 COL4A2 CTNNB1 H2AFX CD81 MYC MGST1 NNMT PFKP SRI TST CALD1 FTL VCL HLA-F TGFBI EMD TIMP2 DDX5 SERPINH1 IL32 TPM2 MT1E TPM1 S100A4 PHLDA2 B2M CRIP2 ANXA7 TPM4 IER3 PFN2 PDXK IGFBP3 HSPA1A PMP22 TAGLN2 ALDH1A3 SIAHBP1 MGLL KRT7 ATOX1 NDRG1 DXS9879E TXNIP KIAA0251 TMSB4X ZYX PRDX2 CSRP1 COX7B RAB13 G6PD MSN SFN S100A10 COL6A1 CKB MDK

STAUNTON_668327 na AKR1C3 LOXL2 TXNIP H2AFX MDK MYC

STAUNTON_668328 na PFKM CSDA LOXL2 LTA4H TALDO1 MT1H PDHA1 MAP1B S100A11 CAST TRAP1 HLA-A LGALS3BP PGD CTSD CTNNB1 H2AFX UCHL1 CLU MYC BOP1 CTGF MGST1 ID3 PYGB SREBF1 PFKP COL5A2 IFITM3 /// IFITM2 FTL HLA-F TGFBI COX5B DPYSL3 AKAP12 CDKN2A ITGB5 IL32 MT1E C10ORF116 CYR61 COL1A1 CRABP2 IGFBP3 ACTN1 HSF1 SNRPN /// SNURF GNG11 PXDN SHC1 PSME2 BLVRB POLR2J SIAHBP1 MGLL DXS9879E TXNIP BCAP31 NIFUN ALDH1A1 AKR1B1 /// EIF3S9 LASP1 PRDX2 PPIB G6PD AKR1C3 GDF15 CD9 S100A10 CYBA COL6A1 PSMD6 CKB

STAUNTON_668329 na S100A2 CYB5R3 HLA-A MFGE8 CST3 NQO1 TAGLN HLA-F CD99

STAUNTON_668331 na CSDA FLNA FHL2 LOXL2 APOE SLC6A8 HSPH1 TUBA3 TALDO1 PDHA1 CPNE1 PMM1 FSCN1 MYL9 HLA-B /// HLA-C UCHL1 SERPINB6 BOP1 CTGF NNMT UGCG COL5A2 CALD1 GGH NDUFS8 SPCS2 CKAP4 IL32 MCAM TPM2 TAGLN MT1E TPM1 S100A4 DSTN DUT LRPAP1 MFGE8 TNNC1 H1FX S100A2 IGFBP3 ACTN1 CTSL AIP PXDN BLVRB LGALS3 /// GALIG EEF1D RAB2 SIAHBP1 TXNIP ID2B ZYX PLD3 PRDX2 CSRP1 GDF15 CYBA FGFR1 ABLIM1 TFPI2

STAUNTON_668332 na CYR61 CSDA BZRP NPC2 MYH10 MYH9 EMP3 MFGE8 TIMP3 SPARC FN1 CYB5R3 OAT HLA-A TRAP1 LGALS3BP CTSD CD99 H2AFX LGALS3 /// GALIG CLU MYC SIAHBP1 LGALS1 DXS9879E SREBF1 FAM38A FTL IFITM3 /// IFITM2 TGFBI HLA-F PRDX2 EMD TIMP2 AKAP12 ITGB5 CST3 MCAM IL32 TPM1 NBL1 MDK CAPG

STAUNTON_668333 na CSDA NPC2 MYH10 DUT MFGE8 SPARC FN1 OAT APLP2 IFITM3 /// IFITM2 // CD99 H2AFX LGALS3 /// GALIG SIAHBP1 BOP1 HSPB1 SREBF1 COL5A2 FTL IFITM3 /// IFITM2 HEXB ALDH1A1 EMD CKAP4 AKR1A1 PRSS11 CAPG TFPI2

STAUNTON_668334 na DXS9879E TM4SF1 TRAP1 HLA-A IFITM3 /// IFITM2 // FTL TAGLN CIRBP

STAUNTON_668336 na CSDA FLNA NME4 TUBA3 TALDO1 PDHA1 CPNE1 UQCRC2 CTSD MYL9 PGD HLA-B /// HLA-C CD99 H2AFX UCHL1 MYC HADH2 PDLIM1 TM4SF1 MGST1 ALDH2 PFKP TST CBFB NDUFS8 GPX1 CKAP4 SERPINH1 CAV1 SMS DPM1 LTB4DH TAGLN S100A4 ANXA7 MYH10 CCNG1 CRABP2 DUT MFGE8 EMP3 SQSTM1 PDXK FKBP1A CLNS1A /// C3ORF4 CTSL HDAC1 AIP PMP22 PXDN EEF1D LGALS3 /// GALIG NR4A1 TETRAN MGLL LGALS1 PSMC2 GCLC ID2B NIFUN AKR1B1 /// EIF3S9 ZYX SILV CSRP1 G6PD NEDD8 MSN NUTF2 XBP1 PEA15 ABLIM1 MDK TFPI2 CAPG NPC2 PON2 LOXL2 QSCN6 SLC6A8 HSPH1 MT1H SNRPF FBL BTG3 HMGN3 FH SERPINE1 LEPROT BOP1 S100A13 SREBF1 RBBP7 FTL IFITM3 /// IFITM2 FABP5 CYC1 ADK ARPC1B DDX5 ESD CDKN2A RRAS ATP5G2 KIAA0101 IL32 MCL1 TPM2 CDA TPM1 P4HA1 CIB1 DSTN TOMM20 LRPAP1 IGFBP4 H1FX ARPC1A S100A2 IGFBP3 HSF1 IFITM3 /// IFITM2 // NQO1 BLVRB SIAHBP1 KRT7 TXNIP FAM38A AKR1C1 ACLY AKR1C3 ACP1 GDF15 CD9 GNAS CTSH COL6A1 FGFR1

STAUNTON_669615 na MTHFD2 ATP5J FBL UQCRC2 RHOD YWHAH MYL9 CTNNB1 CD81 H2AFX CLU LDHA EIF3S6 KYNU TUBB3 CTGF BOP1 MGST1 SRI FABP5 RANBP1 MCM4 COX5B TAX1BP1 ICT1 SPP1 LUM FXR1 S100A4 COMT TXN MFGE8 ADSL ACTN1 ATP6V0B SNRPN /// SNURF CCND1 CTSL MVP PTP4A1 UBE2D3 HNRPD CD47 ALAS1 LY6E TCP1 GNG10 /// LOC552891 TMSB4X STOM PLOD2 SET SFN CYBA NBL1 ASNS MDK CAPG

STAUNTON_669727 na HMGA1 NPC2 NME4 KRT18 TUBA3 MT1H ANXA2 CCNH PRDX1 LGALS3BP PGD ITPK1 HLA-B /// HLA-C CD99 KIAA0114 SERPINE1 CLU CDKN3 ETFB CTGF ID3 NNMT GRN JUND HRAS VCL TIMP2 CAV1 SERPINH1 TFRC SNRPD3 PTPRF ITGB5 KRT8 MT1E TPM1 CYR61 TXN CAPZA1 MFGE8 SQSTM1 ATIC GNAI2 PTTG1IP IGFBP3 CCND1 RARS ZNF91 SLC7A5 POLR2J MGLL CD59 SSR4 DIPA SLC39A6 FAM38A EBNA1BP2 PCNA PSMA5 RHOC AKR1B1 /// EIF3S9 LASP1 CSRP1 GAS6 PGM1 CD9 ARF4 MT2A /// LOC441019 LITAF SFRS3 PEA15 CAPG

STAUNTON_670653 na IGFBP3 ITGB1 APOBEC3B PTTG1IP

STAUNTON_670779 na MGLL CSDA FLNA TM4SF1 CCNG1 CRABP2 ECH1 FTL PDHA1 NDUFV3 TAX1BP3 FLII CSRP1 IGFBP3 ITGB1 CAV1 COX17 MDK MYC

STAUNTON_670783 na FHL2 LOXL2 KRT18 TALDO1 MT1H PDHA1 SNRPF FN1 PLOD1 BTG3 CEBPD TRAP1 CD24 CTSD CD99 H2AFX SERPINE1 MYC PRDX4 KARS LEPROT BOP1 MGST1 HPRT1 NNMT RBBP7 ALDH2 COL6A2 FTL IFITM3 /// IFITM2 VCL EMD ADK CKAP4 DDX5 CAV1 AKR1A1 PRSS11 SMS DPM1 CST3 ATP5G2 MCAM TAGLN PRG1 DSTN MYH10 TOMM20 COL1A1 CCNG1 CRABP2 DUT MFGE8 ECH1 PCBD1 NPIP /// LOC23117 // S100A2 IGFBP3 CTSL HDAC1 IFITM3 /// IFITM2 // NQO1 PMP22 PXDN UBE2V2 BIRC5 ALDH1A3 LGALS3 /// GALIG NR4A1 POLR2J TETRAN MGLL SIAHBP1 TXNIP FAM38A HEXB PRDX2 GAS6 PSITPTE22 GDF15 DDX48 NUTF2 XBP1 FGFR1 CAPG

STAUNTON_670784 na BZRP NPC2 FHL2 HSPH1 BTG3 KIAA0174 CD24 CD99 H2AFX SERPINE1 UCHL1 IQGAP1 CTGF BOP1 ID3 CRYAB RBBP7 SREBF1 FTL IFITM3 /// IFITM2 TST CBFB TGFBI HLA-F CKAP4 ARPC1B ESD SMS UCP2 CDKN2A ATP5G2 TAGLN TPM1 CIB1 CRABP2 LRPAP1 NFKBIA MFGE8 PDXK OAT HSPA1A IFITM3 /// IFITM2 // NQO1 UBE2V2 BIRC5 SLC7A5 UPP1 LGALS3 /// GALIG FSTL1 TETRAN MGLL SIAHBP1 LGALS1 FUCA1 DXS9879E TXNIP FAM38A ZYX SRPX AKR1B1 /// EIF3S9 HEXB APOBEC3B SLC25A1 SFN ODC1 FGFR1

STAUNTON_670785 na MGLL COMT CSDA CTGF LOXL2 HSPH1 CALD1 PDHA1 PRDX6 UQCRH CSRP1 S100A2 IGFBP3 G6PD NEDD8 DPM1 AKAP12 TRAP1 IFITM3 /// IFITM2 // TAGLN TPM1 BLVRB UCHL1 MYC

STAUNTON_670786 na GSTP1 TUBA3 PDHA1 PSMA6 MYL9 CAPN2 PGD HLA-B /// HLA-C CD99 MYC MGST1 NNMT CRIP1 PFKP COL5A2 TST VCL EMD CKAP4 GPX1 CAV1 AKR1A1 TACSTD2 UCP2 TAGLN MT1E UBE2S S100A4 CRIP2 CYR61 CCNG1 MFGE8 ATIC TPM4 PDXK PCBD1 GADD45A PXDN LGALS3 /// GALIG MGLL LGALS1 LY6E TMSB4X GDI2 TACSTD1 AKR1B1 /// EIF3S9 ZYX CSRP1 PSITPTE22 NEDD8 RAB13 MSN MT2A MT2A /// LOC441019 CKB MDK KRT18 PABPC4 MT1H IDH2 ASS PLOD1 ITGB1 PLP2 HLA-A COX17 FSCN1 TUBG1 SERPINE1 LEPROT FTL TGFBI SPINT2 CDKN2A IL32 KRT8 TPM2 C10ORF116 TPM1 B2M CIB1 HNRPA1 UQCRH IGFBP3 ATP6V0B CYB5R3 CST6 SNRPN /// SNURF HSF1 GUK1 HSPA1A BIRC5 BLVRB NDRG1 DXS9879E TXNIP FAM38A RHOC PRDX2 LASP1 SLC25A1 GDF15 CD9 DDX48 SFN LITAF COL6A1

STAUNTON_670788 na CYR61 NPC2 CRABP2 MYH9 HLA-B /// HLA-C TPM1 MDK PTTG1IP

STAUNTON_671136 na CKAP4 HLA-A MT2A HLA-B /// HLA-C SPINT2 FSTL1

STAUNTON_671314 na MAGEA12 SPP1 PDHA1 TYMS MAGEA3 PRDX2 MAGEA2 /// MAGEA2B

STAUNTON_671456 na GALE ATF4 PON2 GSTP1 KRT18 TALDO1 SORD LGALS3BP COX17 CTSD HLA-B /// HLA-C KIAA0114 SERPINE1 CTGF ID3 TM4SF1 MGST1 JUP CDC25B TGFBI TMED9 IFITM1 NDUFS8 SPP1 CDKN2A CST3 MCL1 TPM2 S100A4 EEF2 PHLDA2 CYR61 CRABP2 MYH9 DUT SHMT2 PTMS CCND1 IFITM3 /// IFITM2 // FSTL1 LGALS3 /// GALIG RAB2 TNFSF7 SIAHBP1 TKT KRT7 CTSC XRCC5 TMSB4X AKR1B1 /// EIF3S9 SRPX GAS6 PSITPTE22 CD9 MDK CAPG

STAUNTON_672141 na NPC2 GSTP1 PON2 NME4 KRT19 TUBA3 ADA ASS CSK PLOD1 HLA-A LGALS3BP TXNRD1 COX17 FSCN1 PGD CTSD HLA-B /// HLA-C CD99 CLU ETFB ID3 PYGB CDC25B IFITM3 /// IFITM2 HLA-F TMED9 EEF1A2 ARPC1B PSAP TACSTD2 PRSS11 LGALS9 ANXA1 CST3 AP3S1 ATP6V0C TPM2 TPM1 SLC3A2 B2M OS9 CYR61 COMT CRABP2 MYH9 MFGE8 RPS4Y1 GARS ECH1 IGFBP4 PFN2 ARPC1A MGST2 PTTG1IP S100A2 OAT CTSL IFITM3 /// IFITM2 // BLVRB CXCL1 TNK2 LGALS1 FUCA1 KRT7 ATOX1 PRNP SSR4 IL8 KIAA0251 BST2 TMSB4X ALDH1A1 AKR1B1 /// EIF3S9 SULT1A3 /// SULT1A4 SRPX ZYX CSRP1 G6PD GPI CD9 CYBA S100A10 COL6A1 NBL1 PEA15 CAPG

STAUNTON_673104 na COL1A1 CTGF FHL2 MGST1 LOXL2 SPARC ALDH1A1 IGFBP3 TIMP2 ARPC1B HRMT1L1 SERPINH1 GNG11 NPPB PRAME CD9 COL4A2 HLA-B /// HLA-C COL6A1 S100A4

STAUNTON_673267 na ID3 TXNIP CRIP1 RBBP7 MFGE8 SQSTM1 COL5A2 IER3 PDHA1 HLA-F CDKN2A IFITM3 /// IFITM2 // PMP22 BIRC5 SHC1 H2AFX

STAUNTON_673652 na GALE ALCAM HMGA1 NPC2 PON2 HSPH1 KRT19 TALDO1 MT1H ATP5J CTAG1A /// CTAG1B // PCCB EXT1 FAM50A ASS TTC3 IL13RA2 MRPL12 SCARB1 HLA-A LGALS3BP GPC1 BIRC2 FSCN1 CAPN2 TARS SERPINE1 ETFB KYNU SNAPC1 SRI GRN CUL1 TST RPL35A ST3GAL4 TAX1BP3 IFITM1 PAM ARPC1B CAV1 ATP5O PGRMC1 PRSS11 NT5E SPP1 ACOT2 TPM2 MT1E TPM1 IGFBP2 ALG3 MYH9 SLC25A5 PTMS ARPC1A ATP6V0B OAT HRMT1L1 CCND1 CTAG1B /// CTAG2 /// CTPS NQO1 TAGLN2 PSME2 LGALS3 /// GALIG PLP1 TKT CD47 HSPE1 NDRG1 SURF1 TMSB4X HEXB PLOD2 SRPX ARF5 CSRP1 COX7B PSITPTE22 CD9 MT2A SET MT2A /// LOC441019 SLC4A2 CYBA CAPG

STAUNTON_673828 na IGFBP3 CALD1 LITAF MT1H MT1E ID2B CYC1 COL11A1 H1FX ALDH1A1

STAUNTON_673829 na NPC2 LOXL2 KRT18 KRT19 SPARC FN1 ASS PLOD1 ITGB1 FBL HLA-A TRAP1 PSMA6 PMM1 CD24 HLA-B /// HLA-C CD99 LGMN MYC CTGF ID3 CRIP1 COL6A2 PFKP FTL TST TGFBI HLA-F CYC1 EEF1A2 EMD TAX1BP1 PSAP CAV1 NT5E HSPG2 ITGB5 CST3 KRT8 MCAM TPM2 TPM1 S100A4 CRIP2 CYR61 MYH10 COL1A1 CRABP2 DHCR24 MYH9 EMP3 SQSTM1 ECH1 CD63 FKBP1A PTTG1IP DAZAP2 S100A2 CYB5R3 ACTN1 VIM OAT CTSL CTPS NQO1 LGALS3 /// GALIG CXCL1 MGLL ATP1B3 ATOX1 DXS9879E IL8 PLD3 HEXB LASP1 COX7B MT2A TCEA1 COL6A1 MDK CAPG

STAUNTON_673841 na LRRC14 BZRP MDH1 GSTP1 CLTC IFI30 APEX1 ISG20 MCM5 APLP2 UQCRC2 TXNRD1 LGALS3BP ITPK1 CAPN2 CTSD HSPA5 PGD HLA-B /// HLA-C CIRBP CD99 TYMS TARS LGMN SERPINB6 HK1 ID3 SNRPC MGST1 PFKP SRI JUND MCM4 VCL GPX4 NDUFS8 ATP6AP1 EEF1A2 PSMD4 SPCS2 GPX1 CARS HNRPU CAV1 SMS RNPEP ANXA1 MCAM ATP6V0C MT1E S100A4 P4HB PHLDA2 CRIP2 CYR61 TSPAN3 EMP3 SQSTM1 ADAR ATIC PTMS YWHAE CD63 FKBP1A HNRPF NDUFA9 PRAME CTSL PTP4A1 PSMB10 FKBP4 SLC7A5 WARS LGALS3 /// GALIG EEF1D CSNK1E SKB1 CD47 LGALS1 LY6E H2AFZ FASN NIFUN ARMET RBM3 HEXB AKR1B1 /// EIF3S9 RAB13 G6PD MSN ATP2A2 MT2A PCBP1 MT2A /// LOC441019 MLF2 PSMD1 S100A10 ASNS PEA15 MDK NPC2 ALCAM HMGA1 QSCN6 KRT18 TIMM17A PCCB ANXA2 FN1 HSPA9B PLP2 SORD TRAP1 HLA-A KIAA0114 APRT CKMT1B SERPINE1 CLU ETFB BOP1 S100A13 SREBF1 COL6A2 GRN CDC25B IFITM3 /// IFITM2 MLH1 TGFBI CYC1 SPINT2 HSPA4 SNRPD3 KRT8 ACOT2 TPM2 EIF4G2 COL1A1 PLAUR PSMA4 IGFBP3 SLC25A6 GNG11 HSPA1A LDHB MRPL23 NQO1 UPP1 NFE2L2 BLVRB SIAHBP1 DXS9879E TXNIP DDX1 SURF1 INPPL1 FAM38A ECHS1 XRCC5 PCNA SRPX RHOC LASP1 SLC25A1 PPIB PGM1 GDF15 DDX48 CD9 CDK4 SLC4A2 CTSH CYBA COL6A1

STAUNTON_674182 na CSDA GLO1 TUBA3 IFI30 SOX4 ADA CPNE1 NOMO1 /// NOMO2 /// MCM5 CTSD HLA-B /// HLA-C H2AFX LGMN MYC SNRPC MGST1 CRIP1 HRAS EEF1A2 CKAP4 CST3 ATP6V0C LTB4DH MT1E S100A4 P4HB CYR61 MYH10 TXN MFGE8 SQSTM1 ATIC FKBP1A PXDN SLC7A5 WARS LGALS1 LY6E BST2 IL8 ID2B AKR1B1 /// EIF3S9 G6PD MT2A /// LOC441019 S100A10 ACOT7 XBP1 ASNS CAPG NPC2 PON2 KRT18 KRT19 IDH2 ASS PLP2 HLA-A BAMBI CLU PRDX4 CTGF S100A13 SREBF1 GRN IFITM3 /// IFITM2 FTL HLA-F IFITM1 ARPC1B ESD LGALS9 KRT8 TPM2 SRM DSTN IER3 PTTG1IP DAZAP2 IGFBP3 SNRPN /// SNURF GNG11 ZNF91 CSTB COL11A1 BLVRB C5ORF13 CDC20 SSR4 CD7 FAM38A PRDX2 LASP1 PPIB AKR1C3 PGM1 CCND3 CTSH

STAUNTON_675278 na FLNA CLTC RPN2 SPARC KIAA0174 HYOU1 ARF3 HSPA5 HLA-B /// HLA-C CIRBP CTNNB1 CD99 SEPT7 H2AFX GLA TUBB3 TM4SF1 NNMT PFKP GGH GPX4 TAX1BP3 ATP6AP1 EEF1A2 CARS CAV1 MAP2K2 MCAM TAGLN ARCN1 CRIP2 CYR61 DUT MFGE8 THY1 /// LOC94105 PCBD1 COX6C GOT2 ACTN1 MAP4 CCND1 TUBA1 MTHFD1 FSTL1 CSPG2 RAB2 TKT CD47 LGALS1 LY6E TMSB4X ECM1 FASN PTGES3 RBM3 ARF5 MSN PYCR1 DDR1 GPI NBL1 XBP1 PSMD6 PEA15 UBB MDK CKB CAPG POLR2E ALCAM LOXL2 QSCN6 IDI1 KRT18 KRT19 MT1H IDH2 FN1 ITGB1 FBL PLP2 HLA-A COL4A2 COX17 LAMP1 SERPINE1 LEPROT CTGF COX6B1 SREBF1 PAX8 IFITM3 /// IFITM2 PTS COX5B TGFBI EIF2S2 CYC1 SPINT2 KRT8 ARHGDIA TPM2 PRG1 TPM1 P4HA1 B2M COMT COL1A1 CALR LRPAP1 PSMA4 IER3 NDUFV3 IGFBP3 ATP6V0B SLC1A5 VIM SNRPN /// SNURF GUK1 HSPA1A NQO1 UBE2V2 SNRPB2 BLVRB POLR2J KRT7 SSR4 SURF1 SLC39A6 INPPL1 STOM RHOC PRDX2 SLC25A1 GAS6 AKR1C3 GDF15 CD9 SLC4A2 EEF1B2

STAUNTON_676345 na EIF2S1 ALCAM NPC2 QSCN6 VIL2 KRT19 LAMB1 MT1H IDH2 CTAG1A /// CTAG1B // FN1 ITGB1 PLP2 APLP2 HMGN3 HLA-A RHOD COX17 TUBG1 CD24 MYL9 SDC4 CIRBP C1S UCHL1 CPSF1 SNRPC MGST1 TM4SF1 PYGB CRIP1 JUP PFKP TST HLA-F COX5B SPINT2 CYC1 ATP6AP1 NDUFS8 EEF1A2 GPX1 ARPC1B PSAP AKR1A1 MCAM KRT8 TPM1 S100A4 CRIP2 TSPAN3 DHCR24 EMP3 NFKBIA ECH1 GNAI2 SLC25A5 FTH1 PTTG1IP IGFBP3 ATP6V0B HRMT1L1 SNRPN /// SNURF SFRS9 CTAG1B /// CTAG2 /// CTPS PMP22 CSTB ALDH1A3 BLVRB LGALS1 NDRG1 C1QBP TXNIP APOBEC3B PLD3 CSRP1 ACLY MT2A MT2A /// LOC441019 MDK CKB

STAUNTON_676495 na GSTP1 MFGE8 KRT18 ITGB1 PLP2 CTSL LGALS3BP NQO1 HCCS BLVRB DUSP1 LGALS3 /// GALIG ETFB MGLL KRT7 TGFBI SPINT2 TAX1BP3 PRDX2 EEF1A2 CSRP1 ACLY CAV1 SFN KRT8 TPM1 ACOT7

STAUNTON_676497 na GSTP1 KRT18 KRT19 AARS ASS PPP2CB ITGB1 PLP2 LGALS3BP CD24 HLA-B /// HLA-C CD99 TARS ETFB MYC KARS BOP1 FABP5 TST VCL HLA-F TGFBI CYC1 SPINT2 TAX1BP3 EEF1A2 CKAP4 CAV1 ANXA1 KRT8 TPM1 S100A4 B2M LRPAP1 MFGE8 HNRPA1 EMP3 ECH1 PDXK PCBD1 CTSL HSPA1A IFITM3 /// IFITM2 // NQO1 ZNF91 BIRC5 FKBP4 SLC7A5 LGALS3 /// GALIG DUSP1 POLR2J MGLL KRT7 TXNIP SSR4 TMSB4X ID2B APOBEC3B ZYX AKR1B1 /// EIF3S9 PRDX2 CSRP1 ACLY MT2A SFN MDK

STAUNTON_676498 na MGLL KRT7 PLAUR UGCG SNAPC1 RPS4Y1 KRT18 COL5A2 TGFBI ID2B ARF5 PRDX2 CSRP1 ADAM9 KRT8 SERPINE1 MDK MYC

STAUNTON_676914 na BZRP APOE GLO1 RPN2 PPGB NEFL ILK C5ORF18 PRDX1 APLP2 RHOD PSMA6 ARF3 HLA-B /// HLA-C TYMS UCHL1 MYC SHFM1 HK1 SNRPC MGST1 NNMT CRIP1 SRI ATP6AP1 EEF1A2 AKR1A1 SMS RNPEP UCP2 GNPDA1 UBE2S CYR61 CCNG1 DUT EMP3 SQSTM1 ATIC ECH1 CCND1 ALDH3B1 TUBA1 TAGLN2 MGLL DIPA KIAA0251 ZYX CSRP1 PSITPTE22 ASNS CAPG MAD2L1BP PPIF PON2 KRT18 IDH2 TPBG HNRPA2B1 ITGB1 TTC3 HLA-A COL4A2 BECN1 LAMP1 CD24 SERPINE1 CLU CTDSP2 LEPROT BOP1 FABP5 HLA-F CYC1 SPINT2 KRT8 TPM1 CIB1 COMT DSTN NFKBIA C1ORF16 PXN HSF1 ACADVL BIRC5 SHC1 BLVRB POLR2J DXS9879E SURF1 SRP14 SEC24C AP2B1 TCEA1 LITAF CYBA CTSH

STAUNTON_676916 na LRRC14 GSTP1 APOE VIL2 RPL36AL NARS C5ORF18 PRDX1 LGALS3BP RHOD ARF3 PGD CTSD HLA-B /// HLA-C MST1R HCCS TARS LGMN MYC CPSF1 SERPINB6 MGST1 TM4SF1 NNMT CRIP1 PFKP JUP JUND TST MAGEA3 ATP6AP1 NDUFS8 EEF1A2 SPCS2 CAV1 SERPINH1 NELL2 MAGEA12 SMS POLR2F UCP2 ANXA1 MCAM MT1E UBE2S S100A4 PHLDA2 CRIP2 CYR61 ANXA7 PHB2 EMP3 MFGE8 SQSTM1 ECH1 RAC1 PDXK PAFAH1B3 CCND1 CTSL PMP22 ALDH3B1 TUBA1 ALDH1A3 LGALS3 /// GALIG MGLL LGALS1 TKT CTSC C1QBP KIAA0251 GPR143 TMSB4X GDI2 M-RIP ALDH1A1 ZYX HEXB AKR1B1 /// EIF3S9 MRCL3 PSITPTE22 SEMA3C MT2A /// LOC441019 S100A10 ACOT7 RFC4 UBB CKB MDK CAPG NPC2 HMGA1 IDI1 KRT18 KRT19 MT1H IDH2 AARS TPBG TMED10 FN1 ITGB1 ANXA5 HLA-A COL4A2 PMM1 CD24 CLU LEPROT CTGF BOP1 S100A13 HPRT1 UGCG CDC25B FABP5 HLA-F TGFBI SPINT2 SERPINA1 NT5E HSPG2 CDKN2A ACOT2 KRT8 TPM1 OS9 COMT DSTN DHCR24 NFKBIA HNRPA1 GARS C1ORF16 GNAI2 IER3 IGFBP4 SLC25A5 MGST2 S100A2 ATP6V0B OAT SNRPN /// SNURF GNG11 HSPA1A MVP IFITM3 /// IFITM2 // NQO1 BIRC5 SLC35B1 UPP1 BLVRB LOC23117 /// DKFZP54 CD59 KRT7 NDRG1 TXNIP SURF1 FADD PCMT1 ANXA11 EBNA1BP2 RHOC APOBEC3B SEC24C PLSCR1 PRDX2 GDF15 CD9 SFN EIF3S2 LITAF RGS2 CYBA MAGEA2 /// MAGEA2B MAGEA1

STAUNTON_678401 na PON2 NME4 FXYD2 SPARC FLII NARS PLP2 TRAP1 HLA-A COX17 MYL9 HMGA2 CLTA SERPINE1 CLU PRDX4 GNB2 TUBB3 DDOST CTGF SNRPC NNMT COX5B TGFBI SPINT2 EEF1A2 HSPA4 CAV1 PSAP SERPINH1 AKAP12 SPP1 ANXA1 MCAM TAGLN MT1E NCOR2 S100A4 CYR61 COL1A1 DDT MYH9 AP2M1 UQCRH YWHAE PTTG1IP IGFBP3 ACTN1 NPPB CCND1 HSPA1A MVP COX4I1 LDHB SSRP1 PXDN PMP22 SLC7A5 POLR2B CTSC TXNIP TMSB4X ALDH1A1 AKR1B1 /// EIF3S9 RHOC GAS6 INHBA MT2A ANXA4 CYBA S100A10 NBL1 MDK RAE1

STAUNTON_678402 na CYR61 NPC2 HMGA1 FHL2 PLAUR PON2 VIL2 PFN2 SPARC ACTN1 GADD45A MSLN CCND1 SORD IFITM3 /// IFITM2 // SLC7A5 SERPINE1 UCHL1 ETFB SIAHBP1 CTGF SNAPC1 UGCG PCOLCE AKR1B1 /// EIF3S9 ZYX ALDH1A1 EEF1A2 STAT1 PSITPTE22 GDF15 MT2A SPP1 CST3 CDA SLC4A2 ANXA4 NBL1 ODC1

STAUNTON_678880 na DSTN COL1A1 GSTP1 KRT18 ECH1 TALDO1 CD151 IGFBP3 HLA-A HSPA1A NQO1 HLA-B /// HLA-C CD99 H2AFX BLVRB CKMT1B C1S CLU LGALS1 CTGF NDRG1 HSPB1 TGFBI ID2B PRDX2 ARPC1B SERPINH1 PSITPTE22 CD9 MT1E TPM1 COL6A1 MDK CAPG

STAUNTON_678882 na BZRP ATF4 SLC6A8 KRT18 MT1H FN1 ANXA2 PLOD1 MRPL12 APLP2 PMM1 COX17 ARF3 FSCN1 CD99 H2AFX CKMT1B C1S TUBB3 LEPROT SNRPC HSPB1 HPRT1 COL5A2 IFITM3 /// IFITM2 CALD1 NDUFS8 SUMO2 CKAP4 GPX1 SERPINH1 DDX5 CAV1 SMS RNPEP ATP5G2 MCAM ATP6V0C TPM2 TPM1 POLR2G PHLDA2 CYR61 DSTN DUT MYH9 GOT2 IGFBP3 BLVRB LGALS3 /// GALIG TETRAN HMGB2 NDRG1 PRNP TXNIP SRP9 FADD STRA13 TMSB4X EBNA1BP2 ID2B ALDH1A1 ZYX PRDX2 LASP1 CSRP1 STAT1 ACLY SFRS10 PGM1 GDF15 CD9 DDX48 SEMA3C EIF3S2 NUTF2 XBP1 COL6A1 MDK CAPG

STAUNTON_679108 na TKT MGST1 PLAUR ANXA1 NQO1 CTSD UCHL1

STAUNTON_680417 na GSTP1 FLII UFD1L TXNRD1 LGALS3BP PGD CD99 TARS MYC KYNU DDOST HADH2 MGST1 ID3 NNMT VCL MAGEA3 NDUFS8 EEF1A2 SPCS2 CAV1 MAGEA12 PIN1 CST3 ANXA1 LTB4DH MT1E CRIP2 CYR61 MFGE8 EMP3 SQSTM1 TPM4 RARS CTSL SLC7A5 TAGLN2 LGALS3 /// GALIG MGLL TKT MCM7 DIPA TMSB4X ZYX HEXB AKR1B1 /// EIF3S9 CSRP1 G6PD GPI MT2A /// LOC441019 S100A10 MDK CAPG NPC2 SLC6A8 KRT18 FXYD2 MT1H IDH2 TMED10 ANXA2 TRAP1 HLA-A SERPINE1 CTGF S100A13 FABP5 FTL IFITM3 /// IFITM2 HLA-F TGFBI SPINT2 CYC1 CDKN2A TPM2 TPM1 SLC3A2 SRM COMT PLAUR HNRPA1 LRPAP1 IER3 NDUFV3 PTTG1IP DAZAP2 ATP6V0B S100A2 IGFBP3 OAT GNG11 GUK1 HSPA1A SSRP1 CTPS IFITM3 /// IFITM2 // NQO1 BIRC5 SHC1 UPP1 POLR2J CD59 DXS9879E ATOX1 SSR4 TXNIP SLC39A6 FAM38A ANXA11 EBNA1BP2 PSMA5 RHOC PRDX2 ACLY PPIB GDF15 CYBA COL6A1 MAGEA1 BRD2

STAUNTON_680418 na CSDA HMGA1 FLNA LOXL2 GSTP1 SLC6A8 IDI1 KRT18 FBN1 KRT19 LTA4H TUBA3 EXT1 ASS ANXA5 HLA-A RHOD FSCN1 MYL9 CTSD HLA-B /// HLA-C CD99 MMP2 C1S TUBB3 CTGF NNMT COL6A2 COL5A2 IFITM3 /// IFITM2 TST HLA-F TGFBI SERPINH1 ANXA1 KRT8 LTB4DH IL32 MCAM TPM2 MT1E TPM1 CYR61 SRM USP14 MFGE8 EMP3 DUT ECH1 IER3 GNAI2 CD151 CYB5R3 ATP6V0B IGFBP3 ACTN1 GNG11 NQO1 PXDN BLVRB POLR2J ATP1B3 CD59 NDRG1 TXNIP SLC39A6 TMSB4X ALDH1A1 SULT1A3 /// SULT1A4 AKR1B1 /// EIF3S9 RHOC PRDX2 CSRP1 MRCL3 PSITPTE22 GDF15 PGM1 GPI MT2A /// LOC441019 GNAS LITAF XBP1 COL6A1 ARL6IP UBE1 CAPG

STAUNTON_680420 na HMGA1 GSTP1 NME4 IDI1 PGK1 ANXA2 TMED10 CCNH NOMO1 /// NOMO2 /// FBL PLP2 TXNRD1 LGALS3BP CD99 LGMN MGST1 SNRPC HLA-F NDUFS8 HSPA4 SNRPD3 ANXA1 CST3 ITGB5 LTB4DH KRT8 TPM2 TPM1 S100A4 SQLE CYR61 COMT PLAUR MFGE8 DUT EMP3 IGFBP4 SLC25A5 FKBP1A PTTG1IP S100A2 ATP6V0B DAZAP2 GNG11 IFITM3 /// IFITM2 // BLVRB MGLL ATP1B3 DIPA TMSB4X EBNA1BP2 TACSTD1 PSMA5 AKR1B1 /// EIF3S9 RHOC ALDH1A1 SULT1A3 /// SULT1A4 CDH1 MSN PGM1 DDX48 GNAS LITAF SEPW1 COL6A1 ASNS CAPG

STAUNTON_680781 na SRM DDT ALCAM PON2 MFGE8 MYH9 FNTA MT1H FLII IGFBP3 S100A2 HRMT1L1 ANXA5 GNG11 CCND1 HLA-A HLA-B /// HLA-C CD99 THBS2 CTSC NDRG1 SNRPC PYGB EIF5A /// LOC143243 HRAS FASN HLA-F TGFBI PRDX2 TIMP2 ARPC1B MAGEA12 CDKN2A MCAM ATP6V0C IL32 RDBP MT1E CYBA MDK CAPG

STAUNTON_681104 na LRRC14 GSTP1 TUBA3 PDHA1 FLII GTF2I /// GTF2IP1 MCM5 SNRP70 IMPDH1 UQCRC2 LGALS3BP RHOD CIRBP CTNNB1 CD81 TUBB3 PBP SRI JUND SNRPB ATP6AP1 SPCS2 CKAP4 SUMO2 CARS ATP6V0C TRA1 S100A4 P4HB CCNG1 ARHGDIB CD63 YWHAE COX6C LAMC1 NDUFA9 CCND1 CTSL ACAT1 PTP4A1 COX4I1 WBSCR1 EEF1D PSMC2 HSPE1 CTSC C1QBP FASN NIFUN AKR1B1 /// EIF3S9 HEXB SULT1A3 /// SULT1A4 CSRP1 COX7B RAB13 MT2A MT2A /// LOC441019 PSMD1 MAP3K10 IDI1 MT1H SNRPF PCCB ANXA2 PDHB HSPA9B CCT6A PLP2 CLU ETFB CTGF CDC25B FTL FABP5 IFITM3 /// IFITM2 COX5B CYC1 ESD ATP5G2 KIAA0101 IMMT HDLBP COMT DDT ATP5A1 PSMA4 EIF4B GARS UQCRH TNFSF9 OAT MRPL23 SSRP1 LOC23117 /// DKFZP54 KRT7 TXNIP SLC39A6 SDHA NDUFA4 RHOC SLC25A1 DDX48 SLC4A2 CTSH BRD2

STAUNTON_681634 na CSDA DCI ALCAM FOSL1 PON2 KRT18 MT1H IDH2 SPARC FN1 PLOD1 COL4A2 RHOD COX17 FSCN1 ITPK1 MYL9 CD99 H2AFX SERPINE1 TUBB3 LEPROT S100A13 MGST1 NNMT SREBF1 EIF5A /// LOC143243 IFITM3 /// IFITM2 VCL TGFBI MAGEA3 TAX1BP3 GPX1 SERPINH1 AKR1A1 TEGT ITGB5 CST3 MCAM IL32 KRT8 ATP6V0C RRAS MCL1 TPM2 MT1E PRG1 TPM1 CIB1 DSTN COMT DDT DHCR24 MFGE8 FKBP1A IGFBP3 ATP6V0B CYB5R3 CTSL IFITM3 /// IFITM2 // PMP22 UPP1 CXCL1 LGALS3 /// GALIG CDC20 FUCA1 LGALS1 CD59 SLC39A6 IL8 KIAA0251 FAM38A M-RIP CAP1 AKR1B1 /// EIF3S9 RHOC LASP1 PRDX2 INHBA MSN GDF15 PGM1 CD9 LITAF CYBA NBL1 MDK MAGEA2 /// MAGEA2B CAPG

STAUNTON_681635 na CSDA DCI ALCAM FOSL1 PON2 KRT18 MT1H IDH2 SPARC FN1 PLOD1 COL4A2 RHOD COX17 FSCN1 ITPK1 MYL9 CD99 H2AFX SERPINE1 TUBB3 LEPROT S100A13 MGST1 NNMT SREBF1 EIF5A /// LOC143243 IFITM3 /// IFITM2 VCL TGFBI MAGEA3 TAX1BP3 GPX1 SERPINH1 AKR1A1 TEGT ITGB5 CST3 MCAM IL32 KRT8 ATP6V0C RRAS MCL1 TPM2 MT1E PRG1 TPM1 CIB1 DSTN COMT DDT DHCR24 MFGE8 FKBP1A IGFBP3 ATP6V0B CYB5R3 CTSL IFITM3 /// IFITM2 // PMP22 UPP1 CXCL1 LGALS3 /// GALIG CDC20 FUCA1 LGALS1 CD59 SLC39A6 IL8 KIAA0251 FAM38A M-RIP CAP1 AKR1B1 /// EIF3S9 RHOC LASP1 PRDX2 INHBA MSN GDF15 PGM1 CD9 LITAF CYBA NBL1 MDK MAGEA2 /// MAGEA2B CAPG

STAUNTON_681636 na GPX1 FUCA1 MFGE8 MT2A /// LOC441019 MT1E CAP1 CYBA FN1 CAPG

STAUNTON_681638 na DCI HMGA1 NPC2 GSTP1 IDI1 SLC6A8 KRT18 KRT19 MT1H SPARC EXT1 ANXA5 CBX1 SORD RHOD COL4A2 LGALS3BP CD24 ARF3 FSCN1 CTSD MYL9 TRAM2 TYMS SERPINE1 CLU CDKN3 TUBB3 LEPROT SNRPC UGCG JUP EIF5A /// LOC143243 COL5A2 TGFBI MAGEA3 SERPINH1 CAV1 AKR1A1 TFRC UCP2 ITGB5 MCAM KRT8 IL32 ACOT2 TPM2 MT1E UBE2S TPM1 SQLE CYR61 MAGEA4 CIB1 COMT DUT MFGE8 IGFBP4 GNAI2 IER3 IGFBP3 CYB5R3 GNG11 CTSL IFITM3 /// IFITM2 // SRP19 HOXB2 SHC1 MGLL COL4A1 DXS9879E SURF1 GDI2 ECM1 CAP1 HEXB RHOC AKR1B1 /// EIF3S9 MRCL3 STAT1 STMN1 MT2A /// LOC441019 LITAF CAPG

STAUNTON_681639 na DCI HMGA1 FLNA GSTP1 LOXL2 CKS1B SLC6A8 IDI1 TUBA3 EXT1 FBL LGALS3BP FSCN1 ITPK1 HLA-B /// HLA-C CD99 SERPINE1 CLU CDKN3 SNRPC NNMT HPRT1 RBBP7 PFKP COL5A2 TGFBI TIMP2 SERPINH1 CAV1 AKR1A1 ITGB5 KRT8 MCAM MT1E TPM1 UBE2S VDAC1 CYR61 CIB1 PLAUR DUT EMP3 MFGE8 GNAI2 IER3 CYB5R3 IGFBP3 ATP6V0B ACTN1 GADD45A GNG11 CTSL IFITM3 /// IFITM2 // SRP19 POLR2J MGLL ATP1B3 SLC39A6 TMSB4X GDI2 CAP1 EBNA1BP2 RHOC AKR1B1 /// EIF3S9 LASP1 CSRP1 ARF4 MT2A /// LOC441019 LITAF CYBA COL6A1 UBE1

STAUNTON_681643 na MFGE8 EMP3 HRAS EBNA1BP2 PSMA5 RHOC ANXA2 AKR1B1 /// EIF3S9 LASP1 CSRP1 ATP6V0B AKR1A1 PLP2 LITAF TPM1 CD99 CAPG

STAUNTON_681644 na NPC2 GSTP1 KRT18 KRT19 MT1H CCNH ANXA5 NARS HLA-A RHOD COL4A2 FSCN1 HLA-B /// HLA-C CDKN3 ETFB LEPROT CTGF SNRPC ID3 EIF5A /// LOC143243 VCL HLA-F MAGEA3 CAV1 ITGB5 MCAM PRG1 MT1E TPM1 COMT CIB1 MFGE8 IGFBP4 PRKAR1A FKBP1A PTTG1IP ATP6V0B IGFBP3 CYB5R3 GNG11 CCND1 CTSL ZNF91 LGALS3 /// GALIG CD59 DXS9879E SLC39A6 FADD FAM38A EBNA1BP2 SULT1A3 /// SULT1A4 AKR1B1 /// EIF3S9 RHOC LASP1 CSRP1 STAT1 G6PD PGM1 GDF15 ARF4 MT2A /// LOC441019 LITAF CYBA CAPG

STAUNTON_681645 na TTC1 DCI FLNA CKS1B IDI1 KRT18 FN1 ANXA2 FHL1 TMED10 PLOD1 UFD1L PSMA6 COL4A2 CTSD ITPK1 PGD CD99 TYMS CDKN3 LEPROT BOP1 NNMT CRIP1 PFKP COL5A2 TGFBI SERPINH1 AKR1A1 UCP2 ITGB5 KRT8 MT1E TPM1 SLC3A2 CIB1 COMT EMP3 MFGE8 DUT POLR2H IER3 IGFBP3 CYB5R3 ATP6V0B RARS IFITM3 /// IFITM2 // SRP19 SLC7A5 MGLL SSR4 SURF1 PTP4A2 GDI2 CAP1 M-RIP HEXB AKR1B1 /// EIF3S9 RHOC LASP1 CSRP1 MSN LITAF CAPG UBE1

STAUNTON_682306 na GSTP1 NME4 VIL2 IFI30 TUBA3 SPARC LGALS3BP CTSD NMU CD81 DDOST HADH2 MGST1 SNRPC COL5A2 TAX1BP3 EEF1A2 TAX1BP1 CAV1 SMS CST3 MCAM MT1E S100A4 TSPAN3 MYH10 SQSTM1 ECH1 ITGB4 RBBP8 CCND1 CTSL PMP22 TAGLN2 POLR2B SKB1 CTSC TMSB4X ID2B MSN GPI S100A10 MDK CKB NPC2 LOXL2 QSCN6 KRT19 ANXA2 TMED10 FN1 PLP2 HMGN3 HLA-A SERPINE1 CD74 CTGF SNAPC1 UGCG SREBF1 SDC1 FABP5 IFITM3 /// IFITM2 LENG4 TGFBI SPINT2 PSAP TFRC KPNA2 G1P2 IL32 KRT8 B2M OS9 COMT DSTN COL1A1 IGFBP4 UQCRH IGFBP3 ATP6V0B S100A2 VIM SNRPN /// SNURF HSPA1A IFITM3 /// IFITM2 // NQO1 CSTB TSC22D1 C5ORF13 KRT7 SSR4 TXNIP BCAP31 SDHA PSMA5 RHOC LASP1 GAS6 MX1 PPIB CD9 SFN MATN2 LSM1 CYBA

STAUNTON_682769 na FLNA GSTP1 CLTC NME4 ATP5J SPARC ADA DDX39 EXT1 FLII NOMO1 /// NOMO2 /// PSMA6 LGALS3BP ARF3 CAPN2 MYL9 CLTA PIM1 HK1 CRYAB PSME1 CRIP1 VCL TAX1BP3 EEF1A2 SSX2 /// SSX3 GPX1 SPCS2 TAX1BP1 SERPINH1 CAV1 MCAM TAGLN MT1E S100A4 SQLE P4HB CYR61 MFGE8 SQSTM1 POLR2H ECH1 ARHGDIB PCBD1 PRKAR1A GOT2 ACTN1 RARS KIAA0152 HOXB2 PSMB10 TUBA1 ALDH1A3 PSME2 LGALS3 /// GALIG SKB1 LGALS1 IL8 KIAA0251 MRPL3 M-RIP ZYX ALDH1A1 HEXB ARF5 AKR1B1 /// EIF3S9 PSITPTE22 GPI MT2A LTBR POSTN GALE HMGA1 RGS3 FOSL1 KRT18 AARS IDH2 FN1 MRPL12 SCARB1 SORD SERPINE1 CLU ETFB CD74 MXI1 ACO2 ABL1 S100A13 UGCG COL6A2 CALD1 RPL35A SPINT2 ST3GAL4 ARPC1B PSAP SAP18 CDKN2A KRT8 MITF TPM2 CDA CIB1 DDT COL1A1 PLEKHC1 PLAUR GSTO1 MYH9 LRPAP1 CDC37 MGST2 IGFBP3 OAT HSF1 GNG11 IFITM3 /// IFITM2 // SNRPB2 PARP4 MLLT11 DXS9879E SSR4 BCAP31 STRA13 XRCC5 APOBEC3B PRDX2 UBE2C ACP1 AKR1C3 GDF15 C20ORF24 ANXA4 LITAF CYBA COL6A1

STAUNTON_683039 na HMGA1 NPC2 NME4 GLO1 TUBA3 SUMO1 IDH2 MT1H PPGB AARS CPNE1 PLOD1 ANXA5 CAPN2 HCCS BCL3 GCLM MGST1 ID3 TM4SF1 RBBP7 SRI IFITM3 /// IFITM2 HLA-F TGFBI GGH EEF1A2 ARPC1B PSAP CDKN2A ANXA1 LTB4DH DDX11 MT1E GNB1 POLR2G PHLDA2 OS9 B2M ALG3 DSTN CRABP2 MFGE8 EIF4B IGFBP4 PFN2 PCBD1 FKBP1A PTTG1IP HNRPF IGFBP3 MAPRE1 CCND1 IFITM3 /// IFITM2 // AHCY NQO1 PMP22 SHC1 LGALS3 /// GALIG LGALS1 MCM7 TXNIP FAM38A ID2B RBP1 APP AKR1B1 /// EIF3S9 ALDH1A1 APOBEC3B SLC25A1 MSN PGM1 GPI DDR1 MT2A MT2A /// LOC441019 COL6A1 MDK

STAUNTON_683140 na MAPK6 HMGA1 GSTP1 GLO1 KRT18 TUBA3 AARS IDH2 ATP5J ADA PLOD1 HLA-A TXNRD1 PGD TFF3 TM4SF1 SREBF1 VCL HLA-F SPINT2 MAGEA3 TAX1BP3 CKAP4 ARPC1B CAV1 SERPINH1 MAGEA12 SPP1 CDKN2A KRT8 IL32 ATP5G2 TPM1 C10ORF116 PHLDA2 DDT MYH9 HNRPA1 MFGE8 IGFBP4 ATP6V0B GNG11 NQO1 SLC35B1 TUBA1 SHC1 SLC7A5 LGALS3 /// GALIG DXS9879E NDRG1 TXNIP SLC39A6 SDHA ZYX AKR1B1 /// EIF3S9 ACLY PGM1 SFN XBP1 COL6A1 PSMD6 MAGEA1

STAUNTON_683414 na ACTN1 TXNIP CDKN2A MT2A TUBA3 MT2A /// LOC441019 MT1H HLA-F CLU

STAUNTON_683555 na DCI GSTP1 CLTC TUBA3 TIMP3 PDHA1 SPARC CEBPD UQCRC2 PSMA6 ITPK1 HLA-B /// HLA-C CD99 H2AFX MYC MGST1 NNMT PFKP COL5A2 CAV2 VCL TAX1BP3 EEF1A2 EMD CKAP4 TIMP2 CAV1 ADAM9 AKR1A1 PRSS11 SMS POLR2F RNPEP ANXA1 ATP6V0C LTB4DH MT1E UBE2S FARSLA P4HB PHLDA2 CYR61 MYH10 CRABP2 MFGE8 TPM4 ECH1 PCBD1 COX6C CCND1 CTSL PMP22 FKBP4 ALDH1A3 LGALS3 /// GALIG FSTL1 TETRAN RAB2 NR4A1 MGLL LGALS1 PRNP KIAA0251 IL8 TMSB4X CAP1 AKR1B1 /// EIF3S9 ZYX ALDH1A1 COX7B G6PD NBL1 MDK CAPG ALCAM NPC2 PON2 DRAP1 LOXL2 QSCN6 KRT18 HSPH1 KRT19 PGK1 IDH2 MT1H SNRPF FN1 PLOD1 ITGB1 TTC3 TRAP1 HLA-A COX17 FSCN1 FH SERPINE1 CLU PRDX4 LEPROT RBBP7 EIF5A /// LOC143243 COL6A2 IFITM3 /// IFITM2 TGFBI COX5B HLA-F ADK PSAP PGRMC1 SAP18 CDKN2A SPP1 KRT8 RRAS TPM2 PRG1 GNB1 TPM1 CTNNA1 COL1A1 PLAUR NFKBIA LRPAP1 RPS4Y1 MYH9 HNRPA1 PTTG1IP IGFBP3 CYB5R3 S100A2 DAZAP2 OAT GUK1 HSPA1A IFITM3 /// IFITM2 // NQO1 SHC1 UPP1 POLR2J ATP6V1F FUCA1 CD59 MLLT11 DXS9879E ATOX1 TXNIP SLC39A6 FAM38A RHOC SRPX LASP1 ACLY CD9 SFN COL6A1

STAUNTON_683556 na FHL2 CKS1B GSTP1 CLTC TUBA3 SPARC CPNE1 PSMA6 LGALS3BP RHOD KIAA0174 MYL9 CTSD PGD CAPN2 CD99 H2AFX CD81 LGMN UCHL1 EIF3S6 MYC CRYAB ID3 NNMT COL5A2 VCL GPX4 TAX1BP3 EEF1A2 TIMP2 TAX1BP1 CKAP4 CAV1 SERPINH1 UCP2 POLR2F CST3 ANXA1 ATP6V0C MCAM TAGLN UBE2S P4HB CRIP2 MYH10 MFGE8 TPM4 PCBD1 YWHAE FKBP1A GAL GOT2 PSMD2 GADD45A CCND1 PRAME CTSL ACAT1 SLC7A5 LGALS3 /// GALIG TETRAN MGLL TKT CD47 C3 IL8 TMSB4X PTGES3 CAP1 M-RIP ID2B ZYX AKR1B1 /// EIF3S9 SULT1A3 /// SULT1A4 APP CSRP1 COX7B G6PD RAB13 EIF3S8 ARF4 DHPS CYFIP1 MLF2 NBL1 SEPW1 ABLIM1 MDK TFPI2 CAPG EIF2S1 ALCAM PON2 KRT19 IDH2 SNRPF FN1 PLOD1 ITGB1 HAX1 MRPL12 HLA-A COL4A2 LAMP1 COX17 CD24 FSCN1 SERPINE1 CLU PRDX4 CTGF S100A13 RBBP7 EIF5A /// LOC143243 PAX8 COL6A2 FTL CALD1 TGFBI SPINT2 ARPC1B PGRMC1 SAP18 PTDSS1 IL32 RRAS TPM2 PRG1 DDX11 TPM1 CIB1 COMT DSTN COL1A1 DDT MYH9 NFKBIA GARS IER3 SLC25A5 CD151 NDUFV3 PTTG1IP DAZAP2 IGFBP3 ATP6V0B CYB5R3 VIM OAT MAPRE1 GNG11 HSPA1A UBE2V2 UPP1 MLLT11 CD59 TXNIP ACTG1 CXCL2 SLC39A6 FAM38A EBNA1BP2 RHOC PRDX2 ACLY GAS6 AKR1C3 VAT1 PGM1 CD9 PLEC1 LITAF CYBA ARL6IP

STAUNTON_684043 na GSTP1 KRT19 SNRPF CTSD SERPINE1 FSTL1 FUCA1 NNMT TCP1 COL6A2 TGFBI PTGES3 TMED9 SPINT2 CYP11B1 /// CYP11B2 HEXB TGFBR2 CAV1 GDF15 SAP18 NT5E MCAM ANXA4 COL6A1 POLR2G MDK CAPG

STAUNTON_684047 na MAPK6 COMT MFGE8 PSMA4 SQSTM1 ATIC IDH2 SPARC UQCRH DDX39 NPIP /// LOC23117 // PHB PTTG1IP PLOD1 MGST2 IGFBP3 PRAME HMGA2 MTHFD1 SERPINE1 NR4A1 CTDSP2 FUCA1 TI-227H S100A13 SNRPC TXNIP EIF2B2 KIAA0251 BST2 ID2B COX5B TGFBI CYP11B1 /// CYP11B2 SRPX SLC25A1 ARPC1B CAV1 PGM1 GPI NT5E ITGB5 CST3 MCAM MT1E CYBA CAPG

STAUNTON_684836 na PFN2 UQCRH CTSL UQCRC2 SORD HLA-A KIAA0152 CD99 KIAA0114 NDRG1 HK1 TXNIP EIF5A /// LOC143243 DIPA VCL HLA-F TGFBI AKR1B1 /// EIF3S9 MAGEA3 PRDX2 GYPC GDF15 MAGEA12 SPP1 S100A4

STAUNTON_684845 na FNTA AARS FLII GTF2I /// GTF2IP1 SORD HLA-A FSCN1 HLA-B /// HLA-C CD99 KIAA0114 SERPINE1 ETFB TUBB3 SNRPC GRN HRAS FABP5 IFITM3 /// IFITM2 VCL HLA-F CKAP4 CAV1 AKR1A1 MAGEA12 TFRC MCAM PRG1 S100A4 MYH9 TPM4 GNAI2 PFN2 PTMS UQCRH S100A2 SNRPN /// SNURF CCND1 PRAME CTSL CTPS IFITM3 /// IFITM2 // LGALS3 /// GALIG MGLL NDRG1 TXNIP LY6E ARMET ALDH1A1 LASP1 PRDX2 CSRP1 PBEF1 GDF15 GPI MT2A EIF3S2 RDBP S100A10 MDK CAPG

STAUNTON_684901 na DSTN COL1A1 PCDHGC3 /// PCDHGB4 DHCR24 QSCN6 KRT18 MT1H FN1 HLA-A LGALS3BP ACAT1 IFITM3 /// IFITM2 // HMGA2 CTSD HLA-B /// HLA-C CD99 TAGLN2 LGALS3 /// GALIG KRT7 CD59 ATOX1 NNMT PFKP KIAA0251 TST TGFBI HLA-F ZYX AKR1B1 /// EIF3S9 AKAP12 CD9 SAP18 HSPG2 CST3 MT2A /// LOC441019 PLEC1 IL32 SLC4A2 NCOR2 S100A10 CYBA RAE1 MDK CAPG

STAUNTON_684906 na PCDHGC3 /// PCDHGB4 NPC2 BZRP PON2 FOSL1 GLO1 VIL2 MT1H FN1 EXT1 PDHB FBL HLA-A LGALS3BP PGD HMGA2 CTSD HCCS CD99 SERPINE1 MGST1 NNMT PYGB SREBF1 PFKP SRI PAX8 CDC25B TST TGFBI HLA-F MAGEA3 EEF1A2 CAV1 SMS HSPG2 CST3 ANXA1 IL32 CDA MT1E TPM1 NCOR2 B2M DSTN DHCR24 PLAUR EMP3 SQSTM1 IGFBP4 SLC25A5 FKBP1A ALDOA IGFBP3 GNG11 CTSL IFITM3 /// IFITM2 // ACAT1 NQO1 MFAP5 TAGLN2 PARP4 LGALS3 /// GALIG LMNA CD59 CTSC KRT7 HMGB2 DXS9879E ATOX1 PRNP BST2 KIAA0251 STOM EBNA1BP2 ID2B AKR1B1 /// EIF3S9 SULT1A3 /// SULT1A4 PPIB GDF15 CD9 SEMA3C MT2A /// LOC441019 PLEC1 LSM1 SLC4A2 CYBA NBL1 RAE1 MDK BRD2 CAPG

STAUNTON_685687 na ALCAM APOE KRT18 GLO1 KRT19 FNTA IDH2 MT1H FN1 PHB GTF2I /// GTF2IP1 IRAK1 APLP2 HLA-A CBX1 CD24 ITPK1 PGD HMGA2 HLA-B /// HLA-C CD99 HCCS TYMS CPSF1 TUBB3 CTGF SNRPC MGST1 ID3 CRIP1 IFITM3 /// IFITM2 VCL TGFBI CYC1 ATP6AP1 EEF1A2 GPX1 ARPC1B TFRC ATP6V0C RRAS MT1E TPM1 P4HB CYR61 DSTN COL1A1 MYH10 MFGE8 H1FX ARPC1A BNIP3 PAFAH1B3 HRMT1L1 SNRPN /// SNURF CTPS MVP NQO1 HOXB2 PXDN PDIA3 COL11A1 TAGLN2 LGALS1 CTSC DXS9879E IL8 ID2B PRDX2 MT2A /// LOC441019 S100A10 CYBA ODC1 CAPG

STAUNTON_685981 na ALCAM TUBA3 MT1H AARS ANXA2 ADA FN1 FLII CPNE1 PPP2CB PHB ANXA5 FBL APLP2 COL4A2 LGALS3BP PGD MYL9 HLA-B /// HLA-C CD99 KIAA0114 GNB2 NNMT CDC25B TST VCL TGFBI HLA-F COX5B SPINT2 CYC1 GPX1 ARPC1B CAV1 SPP1 KRT8 PRG1 MT1E DDX11 TPM1 S100A4 P4HB B2M DSTN DDT MFGE8 ECH1 IGFBP4 AP2M1 CD63 H1FX S100A2 ACTN1 CTPS SLC7A5 LGALS3 /// GALIG LGALS1 CTSC TXNIP SSR4 LY6E ID2B ZYX LASP1 PRDX2 CSRP1 PSITPTE22 GDF15 SFN MT2A /// LOC441019 ANXA4 CCND3

STAUNTON_685989 na CSDA BZRP GSTP1 APOE TUBA3 PPGB PDHA1 BENE DDX39 FLII PRDX1 LGALS3BP PSMA6 TXNRD1 YWHAH SDC4 CIRBP HLA-B /// HLA-C MST1R CD99 CD81 SEPT7 MYC IQGAP1 HADH2 SNRPC MGST1 ID3 CRIP1 SRI HRAS TMED9 GPX4 ATP6AP1 EEF1A2 EMD GPX1 TAX1BP1 CAV1 TACSTD2 TEGT NELL2 SMS ANXA1 MCAM MT1E SRPK1 POLR2G S100A4 PHLDA2 CRIP2 MYH10 COPA EMP3 MFGE8 SQSTM1 ATIC TPM4 ECH1 ARHGDIB PCBD1 PTMS PRKAR1A PRAME HDAC1 FKBP4 NCSTN ALDH1A3 LGALS3 /// GALIG NR4A1 LGALS1 HSPE1 CTSC LY6E KIAA0251 PTP4A2 TMSB4X NIFUN TACSTD1 HEXB AKR1B1 /// EIF3S9 ZYX ALDH1A1 PSITPTE22 HNRPL RAB13 G6PD MSN GPI SEMA3C MT2A MT2A /// LOC441019 S100A10 ACOT7 ODC1 CKB MDK LTBR CAPG PPIF ALCAM NPC2 QSCN6 PON2 KRT18 PGK1 IDH2 MT1H PRDX6 S100A11 PLOD1 TTC3 HLA-A BECN1 FSCN1 SERPINE1 CLU HSPB1 FTL HLA-F TGFBI CYC1 SPINT2 ADK ARPC1B DDX5 G1P2 AKAP12 NT5E CDKN2A ITGB5 RRAS TPM2 GNB1 TPM1 C10ORF116 SRM DSTN COMT CIB1 DDT COL1A1 PLAUR GSTO1 LRPAP1 NFKBIA UQCRH MGST2 PTTG1IP DAZAP2 IGFBP3 S100A2 ATP6V0B OAT SNRPN /// SNURF CST6 ACADVL HSPA1A IFITM3 /// IFITM2 // NQO1 BIRC5 SHC1 CSTB UPP1 BLVRB LOC23117 /// DKFZP54 SIAHBP1 CD59 DXS9879E TXNIP SSR4 EIF2B2 STRA13 XRCC5 CYP11B1 /// CYP11B2 SEC24C AP2B1 ACLY GDF15 PARP1 CD9 DDX48 SFN TCEA1 SCNN1A LITAF CYBA COL6A1

STAUNTON_688021 na TUBA3 RHEB CPNE1 PPP2CB PRDX1 LGALS3BP YWHAH PGD CD99 CD81 LGMN UCHL1 MYC TUBB3 HADH2 NNMT CRIP1 SRI COL5A2 GGH SNRPB TCTEL1 NDUFS8 TAX1BP1 GPX1 SERPINH1 AKR1A1 MCAM MT1E S100A4 VDAC1 U2AF1 TXN RPL39 CAPZA1 DUT JUNB EMP3 MFGE8 SQSTM1 PSMC3 GAL CCND1 CTSL SLC7A5 ATP1B3 DIPA CAP1 HEXB AKR1B1 /// EIF3S9 CSRP1 MSN SEC61B MT2A /// LOC441019 SFRS3 PABPC1 ALCAM HMGA1 SLC6A8 KRT18 FN1 ANXA2 HSPA9B HLA-A FSCN1 KIAA0114 AK3L1 CLU PRDX4 FABP5 IFITM3 /// IFITM2 HLA-F TGFBI CYC1 SPINT2 PSAP DDX5 DCTD SNRPD3 G1P2 SERPINA1 CDKN2A ITGB5 KRT8 TPM1 SRM COMT NFKBIA IER3 CDC37 UQCRH ARPC1A ATP6V0B IGFBP3 OAT IFITM3 /// IFITM2 // KIF2C SHC1 CSTB C12ORF8 TNFSF7 SSR4 TXNIP BCAP31 PCNA SDHA RHOC GDF15 CD9 TCEA1 RDBP LITAF CYBA

STAUNTON_689531 na CSDA ALCAM FHL2 LOXL2 NME4 PLOD1 ITGB1 FSCN1 MYL9 MMP2 LGMN UCHL1 TUBB3 KYNU CTGF TM4SF1 SNRPC HSPB1 UGCG COL6A2 FTL TST TGFBI IFITM1 TAX1BP3 EEF1A2 SUMO2 TAX1BP1 TFRC G1P2 SMS HSPG2 CDKN2A CST3 ITGB5 ATP5G2 LTB4DH ATP6V0C TAGLN TPM1 PHLDA2 DSTN TXN PPP1CC GARS SQSTM1 IGFBP4 NDUFV3 MGST2 IGFBP3 S100A2 PXDN COL11A1 LGALS3 /// GALIG SKB1 SIAHBP1 DXS9879E TXNIP KIAA0251 TMSB4X FASN AKR1B1 /// EIF3S9 PRDX2 CSRP1 GAS6 PSITPTE22 AKR1C3 VAT1 DDX48 SEMA3C GNAS COL6A1 UBB CAPG

STAUNTON_689533 na COL4A1 NME4 COL4A2 COL6A2 CST3 IFI30 TPM1 LGALS3 /// GALIG UCHL1

STAUNTON_689540 na CSDA GSTP1 NME4 LTA4H IFI30 TALDO1 PDHA1 EXT1 DDX39 CPNE1 PRDX1 APLP2 LGALS3BP CTSD ITPK1 CD99 SEPT7 RPL24 /// SLC36A2 GLA UCHL1 HK1 MGST1 KHSRP SNRPC ID3 NNMT CRIP1 PFKP SRI TST TMED9 CKAP4 SPCS2 MT1E PHLDA2 P4HB CYR61 TXN CRABP2 MFGE8 EMP3 SQSTM1 TPM4 ACTN1 CTSL NCSTN LGALS3 /// GALIG SKB1 PSMC2 LGALS1 CTSC LY6E DIPA KIAA0251 TMSB4X ID2B ALDH1A1 AKR1B1 /// EIF3S9 ARF5 ZYX HEXB SULT1A3 /// SULT1A4 COX7B G6PD RAB13 MSN GPI MT2A MT2A /// LOC441019 UBB MDK RAE1 CAPG GALE HMGA1 LOXL2 QSCN6 MT1H STIP1 FN1 ANXA5 PLP2 SORD COL4A2 FSCN1 SERPINE1 GNB2 S100A13 UGCG HSPB1 FABP5 IFITM3 /// IFITM2 SND1 HLA-F TGFBI IGFBP6 ARPC1B TFRC LGALS9 AKAP12 IL32 ALG3 DDT COL1A1 MYH9 EIF4B GNAI2 SLC25A5 H1FX ALDOA S100A2 IGFBP3 SNRPN /// SNURF IFITM3 /// IFITM2 // UPP1 BLVRB PARP4 KRT7 NDRG1 DXS9879E SSR4 PLSCR1 PRDX2 SLC25A1 CD9 PLEC1 LSM1 LITAF IDH3G

STAUNTON_691243 na PCDHGC3 /// PCDHGB4 HMGA1 GLO1 IDH2 FN1 TMED10 PPP2CB IL13RA2 CBX1 HLA-A COL4A2 HLA-B /// HLA-C BAT3 KIAA0114 SERPINE1 UCHL1 C1S ETFB CTGF SNRPC HSPB1 NNMT IFITM3 /// IFITM2 TGFBI HLA-F EEF1A2 SERPINH1 PGRMC1 PRSS11 SNRPD3 CST3 TPM2 TPM1 SRPK1 IGFBP2 CYR61 COL1A1 PLEKHC1 MFGE8 IGFBP4 PFN2 H1FX LAMC1 IGFBP3 CYB5R3 OAT PAFAH1B3 SNRPN /// SNURF PRAME CCND1 CTSL IFITM3 /// IFITM2 // PXDN TUBA1 TAGLN2 POLR2B LGALS3 /// GALIG TMSB4X ARF5 AKR1C1 CSRP1 PPIB RDBP CYBA SEPW1 ACOT7 MDK

STAUNTON_691519 na FLNA FBN1 MTHFD2 TIMP3 ATP5J PDHA1 RPL36AL JTV1 EXT1 NEFL ILK APLP2 PRDX1 GPC1 CTSD MYL9 HSPD1 CIRBP HLA-B /// HLA-C CTNNB1 CD99 TYMS EIF3S6 MYC EIF3S9 ELF3 TM4SF1 MGST1 NNMT ALDH2 TST GGH TCTEL1 TAX1BP3 SUMO2 CAV1 TACSTD2 MAGEA12 SMS CCT7 GNPDA1 NCOR2 FARSLA PHLDA2 NAP1L1 CYR61 USP14 G3BP EMP3 PPP1CC MFGE8 ADSL SLPI ECH1 PFN2 PDXK GSN YWHAE GAL PRKAR1A RABGGTB BNIP3 ACTN1 HNRPF NDUFA9 HDAC1 RPS3A PMP22 COX7C TAGLN2 CSPG2 MGLL SKB1 COL4A1 HSPE1 HMGB2 C1QBP TCP1 HEXB PLOD2 CSRP1 COX7B SLC2A1 MT2A NUTF2 S100A10 ACOT7 PEA15 ODC1 RAE1 CAPG PPIF POLR2E SLC6A8 KRT18 HSPH1 ACO1 IDH2 STIP1 SNRPF ASS FN1 HNRPA2B1 PIR FBL MRPL12 TRAP1 HMGN3 HLA-A C2F COL4A2 CD24 BCL3 GCLM EXOSC7 GNB2 CTGF BOP1 CALD1 FABP5 HLA-F COX5B SLC39A14 CYC1 AKAP12 CDKN2A SPP1 FXR1 MITF KIAA0101 KRT8 IL32 TPM1 OS9 CIB1 COMT PLAUR DHCR24 PSMA4 MYH9 LRPAP1 IER3 IGFBP4 UQCRH S100A2 IGFBP3 HSF1 HNRPH1 RPL34 MRPL23 MVP SSRP1 LDHB BIRC5 OXA1L DUSP1 TSC22D1 SIAHBP1 KRT7 DXS9879E CCT2 HNRPM INPPL1 NCL NDUFA4 AKR1C1 PRDX2 LASP1 AKR1C3 PARP1 CD9 SFN CYBA MAGEA2 /// MAGEA2B UBE1 BRD2 IMPDH2

STAUNTON_693117 na SSR4 HMGN1 EIF5A /// LOC143243 IGFBP4 CAV2 ID2B TMED9 ANXA2 EEF1A2 PPARG MRCL3 OAT APLP2 GDF15 NT5E ATP5G2 SLC7A5 CTNNA1 MDK LGALS3 /// GALIG

STAUNTON_693167 na GSTP1 TUBA3 FLII TXNRD1 LGALS3BP CAPN2 ITPK1 HLA-B /// HLA-C CD99 LGMN TUBB3 ID3 MGST1 VCL EEF1A2 CST3 ANXA1 MCAM MT1E NCOR2 POLR2G S100A4 CRIP2 CYR61 MYH10 DUT MFGE8 PRKAR1A PAFAH1B3 CCND1 CTSL PXDN PMP22 SLC7A5 POLR2B TAGLN2 LGALS3 /// GALIG MGLL TKT LGALS1 CTSC TMSB4X ID2B ARF5 G6PD SEMA3C S100A10 NBL1 MDK CAPG HMGA1 NPC2 DRAP1 KRT18 KRT19 IDH2 FN1 FAM50A MIF CAST HLA-A FSCN1 SERPINE1 CLU CTGF S100A13 SREBF1 COL6A2 IFITM3 /// IFITM2 HLA-F TGFBI SLC39A14 SNRPD3 CDKN2A ITGB5 TPM2 PRG1 TPM1 COMT LRPAP1 RPS4Y1 NDUFV3 PTTG1IP DAZAP2 IGFBP3 OAT HSPA1A IFITM3 /// IFITM2 // NQO1 UPP1 TNFSF7 CD59 DXS9879E EBNA1BP2 PSMA5 RHOC PPIB PGM1 CD9 CYBA

STAUNTON_693622 na FLNA RPS3 GSTP1 NME4 TUBA3 FNTA TALDO1 XRCC6 CBX1 MYL9 CAPN2 HLA-B /// HLA-C CD99 UCHL1 TUBB3 TM4SF1 ID3 CRYAB MGST1 NNMT CRIP1 PFKP VCL GGH TAX1BP3 GPX1 SERPINH1 AKR1A1 SMS MCAM TAGLN MT1E S100A4 P4HB CYR61 CRABP2 MFGE8 ECH1 PDXK ACTN1 CCND1 CTSL PXDN LGALS3 /// GALIG FSTL1 MGLL ATP1B3 LGALS1 HMGB2 LY6E TMSB4X ECM1 ID2B AKR1B1 /// EIF3S9 SULT1A3 /// SULT1A4 PSITPTE22 NEDD8 MSN GPI MT2A MT2A /// LOC441019 NUTF2 PSMD6 PEA15 MDK ABLIM1 CAPG ALCAM LOXL2 PON2 SLC6A8 KRT18 KRT19 MT1H IDH2 AARS TMED10 ASS HLA-A SORD COL4A2 COX17 FSCN1 SERPINE1 CTGF S100A13 UGCG DNAJA1 HSPB1 SREBF1 EIF5A /// LOC143243 FTL PTS TGFBI ARPC1B DDX5 CDKN2A ITGB5 ATP5G2 TPM2 TPM1 COL1A1 PLAUR DHCR24 MYH9 GNAI2 H1FX NDUFV3 PTTG1IP IGFBP3 ATP6V0B VIM GNG11 CTPS IFITM3 /// IFITM2 // BLVRB CD59 KRT7 NDRG1 TXNIP SSR4 EBNA1BP2 RHOC PRDX2 GAS6 SLC25A1 GDF15 STMN1 CD9 LITAF CYBA CTSH

STAUNTON_693627 na DSTN NPC2 NME4 GARS TALDO1 ARHGDIB H1FX LGALS3BP COL4A2 IFITM3 /// IFITM2 // COX17 UCHL1 LGALS3 /// GALIG TKT NDRG1 CRYAB MGST1 BCAP31 AKR1B1 /// EIF3S9 TAX1BP1 CKAP4 SLC25A1 C20ORF24 TFRC CST3 CDA TPM1 B2M

STAUNTON_693635 na DCI FLNA FHL2 GSTP1 NME4 GLO1 FNTA TUBA3 TALDO1 PDHA1 CTAG1A /// CTAG1B // SPARC DDX39 XRCC6 CCNH LGALS3BP CTSD MYL9 HLA-B /// HLA-C HCCS CD99 UCHL1 KARS TUBB3 HADH2 HK1 PDLIM1 ID3 TM4SF1 SNRPC CRYAB MGST1 CRIP1 ALDH2 PFKP JUP TST VCL MAGEA3 TAX1BP3 TAX1BP1 CKAP4 TIMP2 CAV1 MAGEA12 CST3 MT1E POLR2G NR2F6 P4HB PHLDA2 SQLE CRIP2 CYR61 CRABP2 MFGE8 SQSTM1 POLR2H ECH1 ARHGDIB PFN2 GSN ACTN1 HRMT1L1 PRAME CCND1 CTSL CTAG1B /// CTAG2 /// LGALS3 /// GALIG EEF1D SKB1 TKT PSMC2 LGALS1 HMGB2 UBC TMSB4X ECM1 ARF5 AKR1B1 /// EIF3S9 ZYX CSRP1 PSITPTE22 G6PD RAB13 MSN EIF3S8 PYCR1 GPI ARF4 MT2A MT2A /// LOC441019 NUTF2 ACOT7 UBB ABLIM1 MDK CAPG HMGA1 NPC2 LOXL2 IDI1 SLC6A8 RPL28 KRT18 KRT19 MT1H IDH2 FN1 TMED10 PLOD1 ANXA5 POLD2 SORD HLA-A COL4A2 COX17 ZBTB12 KIAA0114 SERPINE1 ACO2 GNB2 CTGF S100A13 HSPB1 UGCG SNAPC1 RBBP7 EIF5A /// LOC143243 FABP5 FTL IFITM3 /// IFITM2 SND1 SPINT2 ARPC1B PGRMC1 SNRPD3 TFRC CDKN2A ITGB5 TPM2 CDA TPM1 B2M ALG3 RER1 COMT DSTN CIB1 COL1A1 PLAUR GARS IGFBP4 GNAI2 IER3 TNNC1 H1FX ARPC1A PTTG1IP ALDOA CYB5R3 IGFBP3 S100A2 ATP6V0B OAT MSLN IFITM3 /// IFITM2 // BLVRB SNRPB2 POLR2J CD59 KRT7 CCT2 NDRG1 SSR4 BCAP31 ANXA11 SDHA APOBEC3B PRDX2 SLC25A1 GDF15 STMN1 C20ORF24 CD9 LITAF CYBA CTSH

STAUNTON_694456 na BZRP PON2 KRT18 IDH2 ADA CPNE1 ANXA5 APLP2 HLA-A COL4A2 LGALS3BP CIRBP BAT3 LGMN MGST1 CRIP1 PFKP HRAS TGFBI HLA-F PSMD4 ARPC1B SPP1 CST3 POLR2G DDT SQSTM1 S100A2 PAFAH1B3 MAPRE1 MMP1 NQO1 PMP22 TAGLN2 LGALS3 /// GALIG MGLL HMGB2 DIPA PTP4A2 HEXB AKR1B1 /// EIF3S9 TFAP2C PPIB STMN1 GDF15 GPI CD9 SFN EIF3S5 ANXA4 CYBA S100A10 NBL1 RFC4

STAUNTON_694483 na NPC2 PON2 IFI30 KRT19 SPARC ASS ANXA5 APLP2 HLA-A LGALS3BP CD24 FSCN1 CAPN2 HLA-B /// HLA-C ADAM15 LGMN TUBB3 CTGF FABP5 VCL TGFBI HLA-F KPNB1 TAX1BP1 TIMP2 ARPC1B CDKN2A MT1E CRIP2 B2M COL1A1 TSPAN3 SQSTM1 PTMS PTTG1IP IGFBP3 HSPA1A PSMB10 CSTB LGALS3 /// GALIG MGLL SKB1 CRYZ HEXB AKR1B1 /// EIF3S9 GAS6 PPIB PSITPTE22 MSN CD9 MDK TFPI2

STAUNTON_694484 na HADH2 CTGF NPC2 SSR4 CRIP1 FAM38A TMSB4X CYC1 PTMS IGFBP6 PTTG1IP GPX1 LGALS3BP KRT8 PRG1 CD99 PEA15 MDK

STAUNTON_695047 na CSDA GSTP1 NME4 NME1 JTV1 FHL1 CPNE1 NARS SNRP70 APLP2 RHOD LGALS3BP BIRC2 CTSD CD99 SEPT7 UCHL1 SERPINB6 MGST1 NNMT JUND TST TMED9 SNRPB EIF1AX EEF1A2 TIMP2 SERPINH1 AKR1A1 MAP2K2 MCAM MT1E S100A4 MYH10 DUT EMP3 THY1 /// LOC94105 TPM4 PSMC3 ARHGDIB PFN2 AP2M1 PTMS BNIP3 LAMC1 ACTN1 SLC7A5 TUBA1 NCSTN ALDH1A3 MTHFD1 LGALS3 /// GALIG TKT ATP1B3 HSPE1 CTSC DIPA CAP1 ID2B ALDH1A1 ZYX CSRP1 RAB13 HNRPL MT2A MT2A /// LOC441019 PSMD6 MDK MAP3K10 HMGA1 LOXL2 FXYD2 CAST ITGB1 FBL PLP2 HLA-A COL4A2 SERPINE1 CLU ETFB PRDX4 LDHA UGCG HSPB1 CDC25B IFITM3 /// IFITM2 HLA-F COX5B AKAP12 NT5E ITGB5 TPM2 TPM1 SRM COL1A1 GSTO1 HNRPA1 GNAI2 ARPC1A S100A2 IGFBP3 CYB5R3 OAT KIF2C PARP4 CD59 NDRG1 TXNIP SSR4 XRCC5 PAICS CD9 PLEC1 CYBA UBE1

STAUNTON_695267 na HMGA1 NFKBIA JUNB SLPI PTMS ASS TMED10 PTTG1IP CTSL HLA-A PSMA6 IFITM3 /// IFITM2 // CTSD CD99 HLA-DRB1 CLU CD74 CD47 HLA-DRA CTGF MGST1 PSME1 HLA-F ID2B GGH PLD3 RHOC TAX1BP3 TAX1BP1 PSITPTE22 CST3 NBL1 S100A4 GLG1 B2M

STAUNTON_695588 na DRAP1 APOE KRT19 MT1H MIB1 ATP5J FN1 RAD21 TTC3 CCT6A C5ORF18 MRPL12 APLP2 CEBPD TPMT CTSD CD99 HMGN4 TARS TYMS CLU UCHL1 EIF3S6 TM4SF1 MGST1 SDC1 EIF5A /// LOC143243 FABP5 TST HLA-F RRM1 SLC39A14 ATP5O TEGT NT5E SMS SPP1 ITGB5 CST3 CCT7 TMSB10 MT1E IMMT P4HA1 FARSLA S100A4 B2M COMT CLPP PHB2 CRABP2 TPM4 IGFBP4 SLC25A5 CD63 SDCBP MGST2 ADM IGFBP3 CCND1 IFITM3 /// IFITM2 // LDHB ACAT1 SLC35B1 SLC7A5 TAGLN2 BLVRB TNK2 FUCA1 TKT CD47 DXS9879E NDRG1 HMGN1 KIAA0251 TMSB4X AKR1B1 /// EIF3S9 ALDH1A1 LASP1 CSRP1 MRCL3 COX7B PSITPTE22 VAT1 GDF15 MT2A SFN MT2A /// LOC441019 EIF3S2 SLC4A2 CYBA CTSH ASNS FGFR1 MDK CAPG

STAUNTON_695937 na NPC2 DDT MFGE8 SOX4 UQCRH TMED10 IGFBP3 CCND1 HLA-A HLA-B /// HLA-C SLC7A5 CD99 SNRPC HSPB1 CDC25B IFITM3 /// IFITM2 BSG GDI2 PRDX2 ARPC1B AKR1A1 CD9 CAPNS1 KPNA2 MT1E MDK

STAUNTON_695945 na GSTP1 FBN1 TUBA3 TIMP3 RPL36AL EXT1 APLP2 NDUFA12 ARF3 ITPK1 CAPN2 HLA-B /// HLA-C CD99 CTNNB1 TUBB3 KHSRP NNMT COL5A2 VCL EEF1A2 GPX1 CAV1 SERPINH1 PRSS11 CST3 ANXA1 TAGLN MT1E S100A4 CRIP2 CYR61 MFGE8 DUT ATIC ECH1 COX6C YWHAE FKBP1A BNIP3 PAFAH1B3 GADD45A CCND1 CTSL PXDN FSTL1 LGALS3 /// GALIG CTSC COL4A1 TMSB4X ID2B CSRP1 MT2A /// LOC441019 NBL1 SNRPG ALCAM LOXL2 ASS FN1 ANXA2 CAST PLP2 TRAP1 COL4A2 SERPINE1 CLU PRDX4 GNB2 CTGF FTL IFITM3 /// IFITM2 COX5B LENG4 TGFBI SLC39A14 LOXL1 COL3A1 PSAP KRT8 TPM2 TPM1 ATXN2L COL1A1 GARS GNAI2 CYB5R3 IGFBP3 VIM GUK1 IFITM3 /// IFITM2 // C5ORF13 DXS9879E TXNIP PCNA SDHA LASP1 PRDX2 CD9 PSMA3

STAUNTON_696125 na NME4 IFI30 ISG20 LGALS3BP FKBP2 HLA-B /// HLA-C CD99 UCHL1 TUBB3 HK1 ID3 TM4SF1 SNRPC CRIP1 COL5A2 TST GGH EEF1A2 DPM1 UCP2 ANXA1 ATP6V0C LTB4DH MCAM MT1E POLD4 S100A4 P4HB NEDD5 TXN JUNB MFGE8 TPM4 GAL PRAME PSMB10 LGALS3 /// GALIG MGLL CTSC CAP1 ID2B ALDH1A1 CSRP1 G6PD PYCR1 MT2A /// LOC441019 UBB LTBR MDK YWHAZ PABPC1 UBE2L3 PON2 KRT18 SNRPF PRDX6 ITGB1 MRPL12 SORD HLA-A COL4A2 LAMP1 FH KIAA0114 CLU LEPROT CTGF HSPB1 EIF5A /// LOC143243 GRN FABP5 HLA-F PGRMC1 SAP18 CDKN2A ATP5G2 TPM2 PRG1 COMT COL1A1 TOMM20 NFKBIA UQCRH CD151 DAZAP2 SNRPN /// SNURF MSLN GNG11 HSPA1A IFITM3 /// IFITM2 // CTPS MLLT11 ANXA11 AKR1C1 LASP1 ACLY AKR1C3 PGM1 CD9 SFN ANXA4

STAUNTON_696559 na NPC2 KRT18 IDH2 MT1H FLII ADA FAM50A ANXA2 TMED10 JTV1 BAT3 CLU TUBB3 HLA-DRA CTGF ID3 SNRPC SREBF1 RBBP7 COL6A2 FABP5 CYC1 FDPS TCTEL1 EEF1A2 PSAP TFRC SERPINA1 LGALS9 AKAP12 CST3 MCAM PHLDA2 CYR61 DSTN COMT ANP32B MCM3 MYH9 NFKBIA RPS4Y1 IGFBP4 IER3 PTTG1IP S100A2 MAPRE1 CCND1 CTPS PDIA3 HLA-DRB1 HMGB2 CASP4 TXNIP IL8 TMSB4X ID2B PCNA AKR1B1 /// EIF3S9 RHOC LASP1 STMN1 SERPINB2 MT2A /// LOC441019 RDBP DAD1 PEA15 TFPI2 CAPG

STAUNTON_696660 na CYR61 COMT DDT FLNA TXN PLAUR MYH9 IER3 PFN2 SPARC SCARB1 CTSL TRAP1 COX4I1 SERPINE1 LGALS3 /// GALIG HSPE1 CTGF NDRG1 PFKP BST2 TMSB4X AKR1B1 /// EIF3S9 LOXL1 CSRP1 PPIB SERPINB2 SPP1 PLEC1 TPM2 TAGLN RDBP PEA15 CETN2 ATP5D

STAUNTON_696661 na FLNA FHL2 FBN1 TIMP3 APPBP1 NME1 SPARC EXT1 PHB CCNH XRCC6 PRDX1 LGALS3BP MYL9 ITPK1 CLTA H2AFX LGMN GLA C1S SHFM1 HLA-DRA MGST1 ID3 SNRPC JUP PFKP COL5A2 HRAS TMED9 MAGEA3 TAX1BP3 TOP1 CKAP4 TAX1BP1 SERPINH1 UCP2 CST3 ANXA1 MCAM TAGLN UBE2S NCOR2 KDELR2 NR2F6 IGFBP2 S100A4 CRIP2 CYR61 TXN SQSTM1 RAC1 PFN2 HSPCA AP2M1 GAL PRKAR1A GOT2 ACTN1 LAMC1 GADD45A PRAME CCND1 CTSL ACAT1 PMP22 SLC7A5 TUBA1 COX7C TAGLN2 FSTL1 EEF1D SKB1 MGLL HSPE1 CTSC LY6E IL8 BST2 TMSB4X AKR1B1 /// EIF3S9 ZYX SULT1A3 /// SULT1A4 HEXB COX7B PSITPTE22 NEDD8 ARF4 GPI MT2A GTF2A2 CYFIP1 S100A10 DAD1 UBB RAE1 MDK TFPI2 MAD2L1BP CAPG NPC2 SLC6A8 KRT19 MT1H FAM50A ANXA2 PLOD1 FBL TRAP1 COX17 FSCN1 HMGA2 KIAA0114 SERPINE1 CLU PRDX4 CD74 LEPROT CTGF UGCG HSPB1 SNAPC1 TNC RBBP7 COL6A2 CALD1 IFITM3 /// IFITM2 SND1 HLA-F TGFBI HNRPK HSPA4 DPYSL3 PSAP G1P2 AKAP12 CDKN2A SPP1 KRT8 RRAS IL32 TPM2 PRG1 TPM1 CETN2 B2M COMT COL1A1 DDT DHCR24 NFKBIA MYH9 EIF4B IER3 UQCRH H1FX ALDOA PTTG1IP S100A2 ATP6V0B VIM OAT HSPA1A IFITM3 /// IFITM2 // SSRP1 HLA-DRB1 KRT7 ATOX1 NDRG1 PCNA PRDX2 PPIB C20ORF24 CD9 SFN PLEC1 PSMA3 LSM1 LITAF COL6A1 ATP5D

STAUNTON_696662 na CYR61 PHB2 DHCR24 MYH9 IER3 AP2M1 ARPC1A S100A2 MCM5 HLA-A TRAP1 LGALS3BP ALDH1A3 SERPINE1 LDHA SERPINB6 CTGF NDRG1 SNRPC TXNIP PLAU TACSTD2 AKAP12 MCAM IGFBP2 PEA15 CETN2 CAPG

STAUNTON_696860 na KYNU TUBB3 COMT C1QBP TCP1 PFKP PRDX3 STRA13 PTMS AKR1B1 /// EIF3S9 JTV1 GTF2I /// GTF2IP1 PLSCR1 GOT2 MSN SCARB1 MT2A KIAA0101 DUSP1

STAUNTON_697912 na CSDA COMT DDT NPC2 LRPAP1 MFGE8 EMP3 SLC6A8 PPGB TMED10 PTTG1IP IGFBP3 UFD1L GNG11 PRAME LGALS3BP CLU LGALS3 /// GALIG HIST2H2AA PIK4CA CTGF FADD TMSB4X EBNA1BP2 HEXB MAGEA3 RHOC SSX2 /// SSX3 ARPC1B PSAP MAGEA12 PGM1 NT5E MT2A HSPG2 CST3

RAMASWAMY_METASTASIS_128_GENE_CLASSIFIER na VPS13B GREM1 FBLN2 UBN1 PSEN2 RNASE4 AGRN NDUFA1 RNASE1 MT3 CA12 SOX18 DKFZP564F0522 P2RX1 PCNT2 LMNB1 KIAA1102 RAVER1 FXN PDE6A EIF3S12 RBM5 P4HB COX7A2L HR44 C19ORF2 RORB NOTCH3 SRGAP2 MYL2 ROR1 MYLK NUP62 FAM36A PBX2 SLC10A3 ACADSB CHRNA5 NR4A1 CDK2AP1 FALZ SPA17 ITPA MGC23401 FASN MYH11 HRMT1L2 BRRN1 BDH PTN NR2F2 COX7B JMJD1C GPI RAB30 DHPS LOC340318 PTTG1 ABLIM1 DLG3 ELF4 MSX2 KCNAB1 AGTRL1 NOS2A MAD2L1 PGK1 SNRPF STAC PCDH11X SIL ZNF646 COX6B1 POM121 /// LOC340318 ARPP-19 CPM CAND1 SPEN TAL1 WDR37 XAB1 VAV2 NOLA2 NIFIE14 CPN1 COL1A1 HLA-DPB1 POLS ASPA LOC440118 RUNX1 C3ORF6 GP1BA UBADC1 TDG CNN1 BIRC5 RANBP3 MAOB PLP1 MAPRE3 UPF2 TRIM37 FCAR STRA13 ZNF148 INPP5E AKAP8 COL1A2 ZNF212 EIF4E2 F10 PRKG2 REL ACTG2

RAMASWAMY_METASTASIS_17_GENE_CLASSIFIER na COL1A1 HLA-DPB1 MYH11 COL1A2 RUNX1 SNRPF MYLK DHPS CNN1 ACTG2 MT3 RBM5 NR4A1

RAMASWAMY_GCM_TUMOUR_MARKERS na GNA15 C3ORF23 PRC1 FEN1 CKS1B GSTP1 RPN2 MEST PCNX ATF2 ALG1 TSHR ILF2 CD5 IL17 CCL17 PLAGL2 CDK9 TOP2B AGRN NMU PLAC4 RPS2 RPS6KB1 FYTTD1 ADD2 PDK1 HADH2 GSK3B CKAP2 EXTL1 RPLP0 RANBP1 BRF2 MLL5 S100G ABL2 C9 CUL7 LRRC16 OPRK1 LYK5 EGR4 ARHGAP4 CHML SMCX DGKQ ERG CCL19 ALDH5A1 ACTR1B VPS4A P2RY4 SLC6A4 ITGB3BP DUT PCTK1 PSMC3 LAMC2 C1ORF164 PME-1 TMF1 PRIM1 RCN1 CXCL10 HDAC1 PLXNA1 RYBP PRKAA1 GAPDH PARP14 UBL7 TETRAN ARID4A PLOD3 ZBTB17 HIST1H2BE PTP4A2 CD96 FNBP3 PRO0149 ELN GATA4 CKLFSF3 ERAL1 FLJ14681 LIFR RB1 ZFP106 TK1 CASP2 FLJ11021 SNRPD1 NOS2A TUFM CKS2 KRT18 CYP2U1 TMPO TWIST1 LCK PPP4C TRAF4 LRP8 MRPL45 CDCA5 MGC16037 CECR5 MAP3K11 ZNFN1A1 AQP4 CBLB BOP1 HMBS MPHOSPH9 CD1B MYB GCN5L2 KLHL24 HIST1H2BG WDR36 JARID2 IQWD1 SMARCA4 HPCA THOP1 RPS5 ALG3 SRM NUPL1 IL2 STXBP2 COL17A1 PUS1 TH1L DNAH12 GATA2 CCT3 GCNT1 TPX2 CMA1 PLA2G7 ZNF266 CGGBP1 BRCA2 RBBP6 LUC7A RCC2 CRYBA4 CDC23 SEC10L1 MKLN1 CHRNB1 KIF2C NPPA IFNA5 ZNF462 CRYBB2 FLJ20308 NUCB2 FLJ20232 ERCC1 CD7 RHOB SLC17A1 ZNF148 KIAA1219 PAICS CCDC22 ERVK6 RPSA TMEFF1 PTE1 ZFR DVL1 KNTC1 CXCL9 AES CDC2L5 CSDA HLA-DOA KLF1 CHRNA3 NME1 ZNF621 KIAA0999 JARID1A SPTLC2 CCDC25 CA1 ARR3 CPT1A ITGB8 PSMA6 PA2G4 FLJ20758 DSG2 SNX12 MAZ RAG2 UBTF EIF3S9 TNF HLF KIAA0648 KAL1 RPL12 PSMB2 MAGEA3 BF GJA1 TCOF1 MHC2TA BGN EPB41 FAS FLJ10726 LASS6 TIAF1 /// MYO18A CPSF2 AP1S2 CCNE2 TBCD PDYN PSG7 HIST1H4E ARRDC3 LTK ITGB4 TRIM14 SLC12A8 U2AF2 HLA-G TBX2 LSM7 CASP8 PBX2 SNRPA ABCB10 ING3 GPT2 MCM7 ARTS-1 C21ORF91 PRNP C13ORF1 NEK2 CAP350 CRKL RAD51AP1 ANXA8 RASGRP1 STAT1 CDCA7L SLC2A1 RPSA /// LOC387867 / RAB8A AP3B2 LOC340318 SLC7A1 RAB11B MGC33302 ABCE1 BMP7 FLJ35348 MDK RDX HIST1H2BC /// H2BFS ROS1 FOXM1 NR1H2 DLG5 C21ORF66 SNRPF DCP1B GNL3 MIF POLD2 MLLT6 CDKN3 SRY IL2RA MSX2 /// MSX2P LZTFL1 PRKDC TCF3 MED25 EIF5A /// LOC143243 NFKBIL2 ORC6L NR5A1 IFNB1 OSBP TGFBI MMP12 ABAT VHL SSTR1 CDKN2A CNAP1 CRBN RBM33 LMNB2 PEX6 MCM3 HNRPH3 LOC440118 RPS7 PTPN2 LOC285749 COL10A1 C6ORF110 NKG7 MMP1 MAP4K4 CIB2 BIRC5 CD3D NSUN4 COL11A1 SLC11A2 SERPINA3 TDRD9 GBA2 RNASE2 OPN1MW /// OPN1LW NUDT4 CDKN2C DXS9879E RIOK3 NUP88 TUBB ZNF192 76P WDR68 IL17RC DNAJB6 CLCN7 ASXL1 BARD1 GATS FAM84A C9ORF40 SMG1 MAPKAPK2 GNB4 GNAS THSD3 RPS21 EIF4A2 CCND3 DHX37 TNFRSF25 WDR3 ADCY8 SPFH1 SGCG NUCB1 RUNX2 CDK6 VIL2 PKN1 TIA1 SOX4 BAT1 TRIO BCL11B ABCC1 FBXW9 CPSF6 IRX4 KRT6A ZNF154 DNTT CWF19L1 MGC5306 TOP2A LOC257407 TYMS BRCA1 TXK PTPRCAP FBXW5 BAK1 TFDP2 PDAP1 GPX1 DNA2L C6ORF4 RPS26 /// RPS26L /// MPO ILF3 AKAP13 ZEP-2 TYR ERCC2 GALNT2 ASCL1 SH3GL1 ABCF2 FLJ20625 BCL7A FLJ11785 CHAF1A LOC221362 OTUB1 PAPOLA CHRNG ZYG11BL LOC83693 EZH2 APBA2 CCNA1 EYA1 PRAME VPREB1 ETS2 TBL1XR1 LOC283454 CD47 LOC90806 CBARA1 OGT CENPF CBX5 PMS2L5 PRPS1 HRMT1L2 OTUD4 PDE6B SC65 NNT RPS19 HUWE1 TERF1 NUP133 LOC221955 CYP2A7 MYL5 ATF1 SLC38A1 FZD2 NUTF2 HIP1 SFRS3 FLJ14668 PITPNC1 PPARA PABPC1 SFPQ HMGA1 SCML1 ICAM2 TGFB2 PRDM2 KRT19 PRO2730 IDH2 ITGA4 NFKBIB MRPL12 RAD54L TIMD4 ZBTB12 CD6 STX16 RNPC1 PANK3 GYPE FSHB ZNF313 PTMA EYA3 ZNF160 CD2 TRIM15 DHODH MGC72104 ANP32A RPS26 /// LOC338611 SNRPD2 CDCA4 EIF4EBP2 PRTN3 SCOTIN CSNK2A1 KIAA0101 GNB1 APOA4 C6ORF106 TCF7 PIK3R2 CALCB MAGEA4 CAMK2D TRBV21-1 ZNF74 GYPA FGF3 GLRA1 SCYE1 KIAA1155 SHMT2 CA9 TIF1 SIM2 H1FX DLX4 S100A2 HTR6 SLC25A6 PLCG1 SCGF N4BP3 C1ORF59 RPS10 LOC113386 /// ZNF8 HAPLN1 PRKCQ CHPF LOC23117 /// DKFZP54 TBC1D5 KRT7 RPL18A CCT2 FLJ10287 KIF11 RANGAP1 RBMX SEPT6 YARS PCNA MPG BTK KLHDC3 RAP2B DNAJC9 GAPD SAC3D1 CHRNB3 WDR48 PGF KIR3DL2 CYP2F1 PLEC1 MDM4 RDBP SMYD2 SMU1 EEF1B2 XIST GOLGA4 GPLD1 CTAGE5 FLT3 MAGED4 PIP5K2B TOP3A SFRS11 SRP68 ADA ABCB7 CPNE1 IMP-3 APEX1 SULT1E1 HERC2 /// LOC283755 ZWILCH GUCY2D AZU1 NAV1 VAMP1 MC1R TNIK TRBV19 /// TRBC1 RPL35 CCR9 MCM6 H2AFX SERPINB4 EIF3S6 ZNF22 KBTBD4 CPSF1 TMSL8 NNMT JUP CORO2A PRKCBP1 DNAL4 SMYD5 GOSR2 TP53 ARD1A GTF3C5 AMH RASSF7 MCAM FMOD IGHD UBE2S IGFBP2 MCM2 RUVBL2 FOXG1B NSEP1 C1ORF128 ITGB3 RBM10 G3BP DPPA4 GRPR ZRF1 NUP93 SOX11 ZFP36L2 KCNQ1 NUP62 GABRR2 SLC7A5 UCP1 DGKA ZNF37A LIG1 MMP11 LRRC6 RALY PSITPTE22 INHBA JMJD1C LOC81691 KIAA1644 CCNB1 FAD158 TRA@ /// TRDV2 /// T RFC4 MAT2A PDE7A SERPINC1 WSB1 TRBV21-1 /// TRBV5-4 GNB2L1 POU3F1 EXOSC2 PNRC2 ART1 USP39 FBL CBX3 MLLT3 WNT2 ABI2 CD200 ZNF345 UBE3A FANCC ATAD3B /// ATAD3A SEMA3F MEN1 NAPRT1 SPTBN1 RAD9A AK3L1 LDHA KSR NOL10 SLIT1 H2AFV ELAVL2 SSX2 /// SSX5 /// SS SRP72 GNL2 CDC25B WASPIP ADH6 FUT5 CAND1 ZMAT2 RAB11FIP4 RNU60 KIAA0748 CDKN2B TTC7A WDR23 EFNB2 ATAD2 HTR1A EIF4B HIST1H2AI DKFZP434B0335 /// GA MAP2 BAT2 MSLN FLJ12529 FOXJ3 HMGCS1 DLST C6ORF48 FANCG C12ORF8 NGFB DRIM EIF5A FLJ13910 PRSS7 INPP4A ADAR /// ADRBK2 /// MRE11A KRTHB5 ASPH CRYBA1 LOC284702 REST STMN1 ADD1 MYEF2 COMP MAP2K5 HSPA14 DCUN1D1 BRD2 IMPDH2

RAMASWAMY_GCM_NORMAL_TISSUE_MARKERS na PELO TANK SORBS3 LOC91689 SPFH2 WT1 YSG2 PPP2CB PCOLCE2 PRKACG C5ORF18 ACTA2 NPFF AMOTL2 TGFBR1 ACADS DGAT2 MSRB2 SNED1 CA12 BDKRB1 RNF111 CNIH4 ZCCHC2 GABARAPL1 NGFRAP1 CLDN5 LPP MGC14288 AK3 A2M ZSWIM3 ASAH1 MR-1 FBXW4 TNS CAV1 CKLFSF6 SH3BGRL2 DNASE1L1 MICAL-L1 BMPR1A CORO1C MT1E ZNF652 SAT2 CYR61 SLC26A6 C9ORF19 NRK SLC38A2 UBE2E2 RECK TLOC1 TRIM44 ARHGEF15 MAP3K3 STAP2 TNXB GATM SH3GLB2 PTGER3 LRRC4 INSR PODN MGC3123 LOC125150 ITGA7 PAFAH1B1 TFAP2A IHPK2 LOC146712 PECI PRKAA1 ISLR PPCDC CXCL12 RRAGA ANKRD40 ME1 LOC144871 MRPS9 GPR124 DNAJC13 SLC8A1 AXL FMR1 C2ORF30 CTSF ABCD3 MYH11 BTN2A1 IFNAR1 USP48 PER3 BDH FAHD1 IGHA1 /// IGHA2 /// CEL LIG3 MARK3 DPYD ZFP106 UCK1 IGLC2 DES LOC339263 SMARCA2 MGC11332 DHRS6 PURA DICER1 PBX1 MICA /// MICB C7 TGFBR3 DKFZP434D0215 RBM6 TGFB1I1 IPO8 LTBP1 KIAA1458 ORF1-FL49 PCDH1 GNAQ SMARCAL1 KIAA1191 SYNPO2 SORBS1 TSPYL1 CYCS ZBTB38 SLC39A13 GAPVD1 PDIA6 CCL14 /// CCL15 IGF1 TAZ SLC43A1 KIAA1033 SBLF TLN1 SNX9 TPM2 TUBB6 SRC RRAGC LOC171220 /// DSTN FLJ20920 DPT LOC90557 /// DKFZP43 VPS16 C14ORF154 PNLIP EPLIN ALDOB KLF9 ACAS2 C6ORF211 NDUFB3 PRELP LMOD1 CHMP2A CNOT2 LEPR C7ORF27 LPPR2 MOV10 USP36 COL21A1 NS5ATP13TP2 GFER NDUFB1 DAB2IP STX17 REV3L KIAA0859 TRPC1 STOM POP7 ADH1B FLJ22386 XPNPEP1 FLJ11286 FAM26B LOC286044 EDG1 ASMTL ARG99 XLKD1 PER2 MRPS31 RGS2 MGC17624 C20ORF194 PDZRN3 ANGPTL2 LOC126669 RWDD1 JDP2 FBLN2 GK001 F11 FHL1 ARV1 ZNF576 KLF3 RAMP1 TUSC2 JMJD1B ADIPOQ SPAG16 PPP1R12A TESK1 LOC399884 HPS5 FLJ21742 GOSR1 VCL RGS5 EDNRA C6ORF75 TFPI E2IG5 SHBG PCK1 KIAA1272 C22ORF13 PAFAH1B2 TFF2 ADARB1 ECHDC1 LOC285550 COL15A1 CX3CL1 SNX27 GPS2 HCFC1R1 NUDT22 PDLIM5 MYLK NDUFS4 ZFYVE21 CHD3 SERPING1 HAND2 SCP2 RAC3 INHA MGC34646 SCUBE2 C1ORF109 PIP5K1B DCXR PRNP FLJ22269 KIAA1128 IL12RB1 SF3B3 RBL2 NIFUN ALDH1A1 BBOX1 GATA6 IDH3B APEG1 P29 LOC115294 DYRK1A PKIG FLJ21062 CHRND LOC112869 FBXL3 PARP16 ELOVL1 FLJ11200 ABCA10 D2LIC NTAN1 CLIC5 BET1L SLC22A6 ACO1 RPS6KA3 KCNJ8 MT1H IL4 HT007 FNDC3B TBRG1 DNAJB4 SEC23A ABO DMD GPD1 HNMT MXI1 ACTN4 PIGV SUCLG1 JMJD3 MRPL33 SET8 KIAA1280 EPS15 EI24 PNRC1 MAOA HSPG2 MFAP4 CISH TULP2 C10ORF116 RBPMS POU1F1 HTN1 PLEKHC1 FLJ39370 SLC35D2 MSTP9 LOC389203 DHRS3 PRSS23 DUSP3 NDUFA8 FMO2 POLR3G OAT MFN2 AKR7A2 HSPA1A C1ORF31 SLIT2 DUSP1 EPB41L4B FGF8 ARID5A DECR2 SH3YL1 MAP2K4 CAMK2G KIAA0256 EGLN2 NDUFA4 NR4A3 SRPX BAG3 GAS6 INPP5A KCTD2 LOC255458 ANPEP MRPS21 GPT KCNMA1 C2ORF12 SEPT10 LRRC54 ACTG2 AFTIPHILIN EDF1 ADH1C /// ADH1A /// PTRF ISG20L1 ARRDC1 TM4SF10 SDFR1 MPZ FLNA CRY2 PGBD3 MGC16028 LOC89894 WTIP OGN ZNF605 PCP4 H17 GABARAPL2 FAM11A CYP4F3 /// CYP4F2 APLP2 ARHGEF12 EGR1 MYL9 RNF167 PAPD4 SORBS2 GEM NRP1 PPP2R3A ALDH2 C6ORF119 C20ORF35 C14ORF28 CAV2 FLJ20273 GRK1 TATDN1 FLJ20209 SSB1 B4GALT4 NDUFB10 LOC57149 FY KCNMB1 DCUN1D4 TRA1 PLCB3 PACSIN2 NR3C2 SOD3 FLJ11785 C14ORF159 EFHA1 SLC15A1 TACC1 SUPT3H PRKAA2 AKT3 DKFZP564J0863 AGTR1 FBXL5 CGNL1 NFE2L1 CIDEB GSTM3 SNTA1 DHX32 CBX7 ESAM EGFL7 TES SEC24D SPCS1 CDK2AP1 CALB1 GPX3 SLC24A6 BAZ2A ARL1 TBC1D1 SMN1 PJA2 MGP ANKRD15 ZNF434 FLJ21963 EPO PNPLA2 ATP6V1C1 MGC11335 INMT UAP1 EHD2 RBP5 DLC1 NPR2 BRP44L PIP3AP PCGF5 NDUFB6 UBE4B SPARCL1 AOX1 PSG6 PRRG2 MT1X GYPE BOK KLHL21 LOC127262 KIAA1160 /// RAB43 SCO1 TFEB SELENBP1 TGFBR2 ATXN1 RASL12 PREP EPS8 DKFZP434K1421 CTDSPL HIBADH CRYL1 YIPF6 MGC32124 SH3BGR BHMT2 C1ORF24 MRPL18 PRKAR2A DSTN HADHB SNTB2 ITGA8 KCNJ1 PITPNB IGFBP4 ANTXR2 PLAC9 GRK5 TOB2 COL7A1 RHOBTB1 CMYA5 FBXO38 PPP1R1A REXO2 VILL ITPKB LOC146517 GZMH VPS24 FCER1A ALDH4A1 NUDT10 FLJ20254 GNPTG SVIL NOTCH2 CDC42SE1 COL6A3 RGN FEZ2 KLF4 ECE1 SDC2 MGC5987 IGH@ KCTD14 RABEPK DKFZP434L142 KNG1 PER1 SEPP1 ATXN7 KIAA0367 LRRFIP1 RBMS3 GOLGA4 DNAJB14 SEL1L SLC17A5 ARFIP1 TWSG1 FLJ12438 C9ORF5 SFRP1 RNF185 KRAS TIMP3 ADH1A /// ADH1B /// DDR2 CPXM2 SMTN PRLR GSTM2 /// GSTM1 /// DKFZP586A0522 LRP16 C9ORF3 FBLN5 FLJ22709 LOC92305 RNF103 GPR146 AGL MGC27165 RAP1A AQP1 SITPEC DHRS4L2 /// DHRS4 COL2A1 CORO2A COX7A1 SCYL1 NDUFB9 BRS3 ZAK DKFZP686L21136 SCNM1 AIG1 COL4A6 ZNF207 ANKS1 MCAM PEF TAGLN LRP1 TDGF1 ARHGAP10 PHLDB2 KIAA0446 PLN MMRN2 FLJ45445 /// LOC3491 BNIP2 DPPA4 CD302 GSN SRGAP2 TMBIM1 PLEKHH2 CLEC3B KIAA0556 LOC90826 MRPL34 BIRC4 CTTNBP2NL PMP22 LOC90624 MTCH1 TRAF3IP1 PRM1 MSRA STRN3 MGC50853 BTBD15 CCDC2 OPTN SLC37A4 MXRA7 C10ORF72 ZBTB4 GMEB1 MAP3K2 KIAA0143 APP OSBPL9 CLOCK CSRP1 DMN TPST2 NR2F2 FLJ20487 PQLC3 AGMAT NPY1R LOC120224 EIF2S1 CYP4A11 EHD1 TEF KCNE1L PDGFRA NCOA3 PRODH2 SUMF1 FLJ13111 LOC153222 FLJ44635 RPS6KA2 LOC92689 PPP1R14A HMOX2 TP53AP1 WFDC1 SMAD2 PPAP2B PRKACA UQCR KARCA1 GENX-3414 PTGER1 TRIM40 SLC25A28 FAM62A PCYOX1 CNN3 DKFZP761A132 CRISPLD2 NDUFC1 AOC3 UMOD PPP1R12C KIF12 SUCLG2 DBT CRIM1 WWP1 CD79A NDUFA5 C5ORF4 RRAS ACOT2 PEX5 XAB1 TPM1 FUT3 HSA272196 C1ORF166 SLC25A24 ECHDC2 HDHD3 PDLIM2 MAF FOXP1 PHF3 RNF13 DCTN3 C1ORF54 CNN1 UBE2V2 DF TMEM16K FLJ20699 ANKRD25 MAOB RSN TM9SF1 ADAMTS5 ITPR1 DKFZP564M082 DKFZP566O084 FCGRT ARL6IP2 PPP1R12B HAAO SART1 DAB2 APOBEC3B C10ORF9 FBLIM1 WDR25 LOC339745 FLJ12886 ATP1B1 SMPDL3A PDCD8 BCL2L2 RPS6KA5 ADAMTS1 PELI3 LOC255512 ITSN2 FOSL2 SLC25A4 SGCE PTEN

RAMASWAMY_GCM_BLADDER na CENPE KIR2DL3 RREB1 WT1 KHK CCL1 ALG1 C15ORF31 C8G PHYH NAT2 DRD3 ATP8A2 PPM1M TCP10 ACTA2 GPX2 ACADS DGAT2 YLPM1 RAPSN BDKRB1 KLHDC4 DAF CHRM1 ZNF593 GOLGA1 CTF1 KRT23 SPRR1A VGLL1 AGRP RNF113A C9 LRRC16 CDC27 PROC ARHGEF5 B4GALT1 KRTAP5-9 DNASE1L1 ACTC GLRA2 QPRT CACNB3 ABHD2 GHRH THBD IRF5 CHRM5 CALML3 STAP2 ACR LAMC2 TDRD3 GGTL3 TCF19 PTGER3 C4ORF10 GC INSR TFAP2A PLXNA1 REN ZNF84 CALM2 PRB3 C4ORF9 BAGE KRT13 SLC8A3 RBM35A SLC8A1 SLC25A13 TRIM25 PRO0149 MLF1 TMEM49 EIF2AK4 CCKAR THRB RARG MYH2 GABRA3 MUC15 PDE1C SLC27A4 FLJ23186 IGLC2 LIPF SNCG CSTA SMARCA2 YAF2 S100A12 CYLC1 CTSB ZNF143 PIR MAC30 PKIA PPARG SH3MD2 DOC2B FRK NPR3 EPHA5 ELF5 FLT4 TGFBR3 NTRK3 OR1E1 RIN1 IFI35 PTPRR OR3A1 SLC39A11 SELE RILP PCDH1 TRIB1 FLJ25222 WIT-1 SDC1 GPR19 COL6A2 KCNJ15 ITGA3 UPK2 USP28 KRT16 SLC43A1 FOXF2 TPM2 TFAP4 MGC14376 CHD7 POU3F4 TCEA2 PVALB CDH12 SP1 IGFBP3 LMOD1 KLF5 HNF4A DNAH14 LOC92691 SUOX MAGEA5 IGLC1 /// IGLC2 /// COL21A1 ETV3 BHMT KCNJ4 NDRG1 TCN2 ERCC1 SERPINB5 IL17B H1F0 SH3BP2 DGCR11 KRT2B BCL2L14 ALB TRIMP1 FLJ21839 NAGA NPAL3 AKR1C3 RGR ERVK6 SFN ZFR DVL1 DCC RGS2 HSPB3 HAS2 KCNJ11 SEPT8 TIMP4 HLA-DQB1 CHRNA3 MAGEA9 RELA F11 GAGE8 /// GAGE4 /// MAPK11 AHSG GPR109B FAM12A S100A7 ABHD6 CRYGA SLC39A3 ELF3 CLCN5 MGST1 TESK1 HES1 IL1B CAMP HRAS MAGEA3 GJA1 SERPINA7 ID1 IFNA14 LOC166994 /// LOC340 E2IG5 FAS SHBG FXN FPGS KIAA1272 POLA2 VAMP2 PHLDA2 RPL39 TNNI1 IL8RB CHD5 CLUL1 PLGLB1 ERBB2 CXCR3 DPF1 POU4F1 SLC20A1 AMELX /// AMELY RAB3A CASP8 RTTN CNOT7 SALL1 SCUBE2 COLEC11 ADRA2B ADH4 UBC MYL3 LOC400581 CRKL APOL1 SILV IDH3B CSNK2A2 SLC2A1 ANGPTL4 DYRK1A SLC12A1 IL11 FOXA1 TMEM51 IL6 RAB25 KRT5 FLJ35348 MDK PSG1 AADACL2 GALE MECR ESM1 ZNF35 ATP4A SMPDL3B RPS6KA4 GRIN2B EMP2 HMOX1 CART1 DMD XG RND2 ITGB6 RYR2 GPATC3 FGFBP1 HMGCS2 IHH JAG1 C6ORF152 TCF1 IFNB1 IGKC SPRR2E ZNF609 MC4R FABP4 FOXC1 MT1F FLJ37440 SMAD3 MAOA VNN3 RPL23A /// LOC130773 C10ORF116 PEX11A NMB SSTR3 CFDP1 KCTD4 FABP6 MSTP9 DHRS3 PRSS23 UMPS CCL13 MMP1 SLC6A14 GPR89 FAM3B GPR4 OCA2 CYP1A2 ZXDC COL11A1 PYGO2 FUT2 GPR6 OAS1 KRTHB6 BRD1 GUCA2A PERP CCKBR MAPRE2 IL9R /// LOC400481 SCNN1B AKR1C1 NR4A3 AR HCG9 MOSC1 FAM84A LOC348938 KRTHA3B MAPKAPK2 HOOK2 ARMCX4 ACTG2 LSM1 EIF4A2 H19 GTPBP1 S100P SPRR1B GALNS MPZ PGBD3 WNT5A PRPSAP1 RUNX2 RBMY1A1 PCP4 NFYA PTPRM MAP3K7IP1 RAB6B SOX3 GPC3 FGF7 MT1B MAGEA10 NARG1 SHFM1 CEACAM4 P2RY10 PTGS2 KRT20 EGF MGC4677 CYP3A5 ZNF278 KRT12 TACSTD2 RLN1 ATN1 GSTM5 SLC19A1 LAD1 HRK DTNA ASCL1 SOD3 CLDN4 MCRS1 MLLT1 OMD NRCAM APBA2 EYA1 SGCA CCND1 AGTR1 GSTM3 HLA-DQB2 GP5 GABRA6 ZFYVE16 KRT14 LY6D CALB1 ATPBD1B KIAA0251 HBEGF PTX3 GNB3 GJB2 TGFA OIP106 FXYD3 ASAHL NFRKB MYF6 GALNT4 EVPL PMS2L1 RBM35B DST IGSF9 SELP PDCD1 TRPC3 KRT19 NPR2 ZNF662 PIAS3 PLA2R1 NTRK2 TMEFF2 GHRHR IL3RA RAB22A AOX1 DLX2 CGB /// CGB5 /// CGB IVL SSH3 DUSP26 FAM13A1 PHKG2 MYOM1 SELENBP1 TNFAIP2 KLK3 ICAM4 KIFC2 NPAS2 SETMAR SH3BGR MGC32124 NF2 CYP4B1 CD1A DNASE1L2 PLAUR C1ORF91 ALAD NRG1 IER3 CA9 ESR2 PLAT PAX3 SIM2 TNFAIP6 S100A2 KIAA0664 CST6 FLJ90586 NPPB BMP5 PGA5 EPPB9 KRT17 FOXF1 XK KRT7 ALDH4A1 CAPS COL6A3 MGAT5 CD4 IL3 MDFI PPP2R2B BBS4 FLJ21439 GCNT2 CHRNB3 GDF15 CPN2 HTN3 KNG1 OR3A2 /// OR3A3 CCL20 TAF15 MAS1L MAGEA1 LILRB4 CXORF12 IGF2 CD36 STC1 ASGR2 SLC2A4 RNF185 FGFR3 SMPD1 TALDO1 ADH1A /// ADH1B /// DDR2 DUX4 /// LOC399839 / IGKV1D-13 MPHOSPH1 GSTM2 /// GSTM1 /// SULT1E1 ZWILCH HIST2H4 SCN2A2 CLEC1A ZNF167 MT3 MPP3 IRS1 CYP24A1 ADAM2 XDH TUSC3 MGC27165 PSCA COL2A1 CFH /// CFHL1 LAMB3 UGT1A10 /// UGT1A8 / STARD8 C1ORF61 PRKCBP1 MAP3K14 UGT2B15 ZAK CKM DEFA6 IGLC1 CCNE1 GOSR2 F25965 TBX5 ICT1 C9ORF12 COL4A6 CHRNA6 INPP1 PLA2G5 HNF4G PIK3R3 DIO3 MGC23280 IL10 ERCC8 FLJ45445 /// LOC3491 ACADL GATA3 CASP9 ITGB3 G3BP ERBB3 DPPA4 CRYGB CYP4F12 MYOG CLEC10A ATXN7L3 UGT8 DNAJB12 LYZ CP FLT1 SDS DGCR2 CHGA ITGA2B SFT2D3 HLA-DQA1 G1P3 PRM1 SLC7A2 MAP1A EPB42 KRT15 ALPK3 EDNRB HSD17B2 DNM1L PSITPTE22 RBL1 GRB7 GDF10 ELP3 SERPINB3 CREBL1 PTHLH NFKBIL1 SMPD3 AP3M2 MYL4 NPY1R C1ORF86 ACVR1B HIST1H4K /// HIST1H4 PSPH PDZK10 GRB14 CCR2 SPRR2D ZNF672 TP53AP1 ASH2L IL8RA SEMA3F GRM8 CRH C8ORF72 LOC58489 GUCA2B EDN2 SHC3 GLRB GP2 PEX19 FABP5 AOC3 LDHC UMOD TP73 CLN5 DLG4 CMKOR1 MT1G CXADR SYT5 ASIP CD79A BDKRB2 SYCP1 CNNM4 ZNF294 KRTHB3 CREM CYP2D6 EGLN3 SPINK1 IFNA10 ZNF193 FBP1 PTK6 WDR23 LOC143381 NR5A2 AFF2 GLP1R KRT6B MMP13 GSTM1 /// GSTM2 /// DKFZP434B0335 /// GA MYO7A NELL1 GRP TNNI2 PKP1 PAH EVI1 RDH5 TGM2 CNN1 TMEM16K SHC1 HPGD DRD2 ZNF42 MPL DUSP4 E2F1 PCYT2 PRSS7 TG SLC35D1 ADAR /// ADRBK2 /// GLI2 HOXB1 DLGAP1 CDH1 LARP6 SLC2A12 ADAMTS1 QPCT RBP3 CRAT TRDN PRPH DHRS2 PTD015

RAMASWAMY_GCM_BREAST na PYGM MAN1A1 AVPR1B CENPE MUC5AC KHK CCL1 ARHGAP26 PHYH DRD3 NAT2 ATP8A2 NTF3 TCP10 AGRN COG2 SLC18A2 AFP YLPM1 KCNB1 CA12 RAPSN LOC83690 PDK1 FLJ30990 POLR1D ZNF593 GOLGA1 COL5A2 MYH6 /// MYH7 PITX2 SPRR1A PRPSAP2 C9 ZNF187 LRRC16 CDC27 KRTAP5-9 FAM40B GLRA2 DIO2 CACNB3 ENPP1 CRIP2 TUBA2 /// LOC112714 GHRH PAXIP1L C10ORF10 P2RY4 CRABP2 ACRV1 ACR TNXB TDRD3 GGTL3 PTGER3 LRRC4 C4ORF10 GC P2RY2 SCN5A CD1D FKBP4 FOLH1 CASP10 PRB3 C4ORF9 BAGE TMEM33 ALPP SLC25A13 TRIM25 LOC219854 EIF2AK4 IRX5 RARG CYP2A6 PDE1C CEBPE CALB2 RAB40B SNCG SMARCA2 F8 CYLC1 CTSB EFEMP1 SH3MD2 DOC2B DNAJC4 BMP6 EPHA5 FLT4 HMGA2 SERPINE1 GRM1 WIT-1 KIAA0133 KCNJ15 G1P2 HPCA FOXF2 TFAP4 AURKAIP1 P2RX7 LOC90557 /// DKFZP43 ESR1 TCEA2 NAT1 CDH12 SP1 DNAH12 HSD3B2 LFNG TAF4B CPB1 FLJ90834 CRYBA4 HNF4A PDGFRL MAGEA5 LTF NFKB2 TRIM16 ETV3 PRL BCR SDF2 SPUF KIAA0859 SH3BP2 IL17B DGCR11 KRT2B BCL2L14 POP7 THOC1 XPNPEP1 MAPT SMOC2 RGR ERVK6 DCC PDCD2 HSPB3 C11ORF30 HAS2 MN1 IL13 KCNJ11 RNF3 HLA-DOA LOC126669 CHRNA3 KIAA0826 CYP2B6 F11 MAPK11 C4A /// C4B FAM12A S100A7 PIGR GPR81 MESDC2 MB CLCN5 HLF CXORF45 EFHD1 KAL1 FLJ21742 HFE BF SERPINA7 MHC2TA LOC166994 /// LOC340 LRRN1 TTN FAS SHBG SYN2 CCDC28B EFNA5 BCHE CRK C16ORF9 B7 CHD5 TCP11L1 MGC17943 ADAM17 TAF1A FGF4 CXCR3 TRAF7 DPF1 PCSK7 POU4F1 NOTCH2NL STC2 RAB3A TNK1 PDE4C CHRNA5 PPIL2 SCUBE2 LOC123722 ADRA2B TSPY1 TJP3 DCXR LOC400581 ANXA8 APBA2BP CHGB IDH3B OCM SLC9A3R2 IDE DYRK1A NEK4 FOXA1 LOC112869 FLJ35348 CXCL6 POSTN FMNL2 FUT4 SULT1C1 MECR GIT2 ATP4A ZNF35 CYBRD1 SMPDL3B RPS6KA4 HMOX1 CA14 FNDC3B CRISP1 ABO DMD XG BAMBI RND2 ZNF10 ITGB6 RYR2 EZH1 INPP4B SMA3 /// SMA5 DNAH3 IFNB1 IGKC TEAD1 HIST2H2BE CD80 DMPK C10ORF116 DCPS GNL1 GAL3ST1 AZGP1 POU1F1 CFDP1 ZNF250 HLA-E HAB1 HTN1 COL1A1 MAG MSTP9 FST PRSS8 LOC285749 ARHGDIG FMO2 DSCR1 RAB31 E2F4 AMT GPR4 C15ORF29 CYP1A2 ARSB COL11A1 SERPINA3 SLC6A12 VAC14 HNRPD MARCH1 TNFSF7 D4S234E KRTHB6 BRD1 RAI14 CCL4 CCKBR HDC IL15RA TNNI3 NR4A3 AR NR0B1 SFRP2 FDXR C22ORF3 SARM1 KIAA0683 TNFRSF25 ISG20L1 STK6 NID2 MYCNOS MPZ PGBD3 RUNX2 RBMY1A1 MRPL48 ZNF79 GBA /// GBAP WDR79 MAP3K7IP1 SOX3 TAF1 GPC3 BDNF MLLT10 FGF7 MT1B KIR2DL4 NARG1 IL24 PRB2 CD164L1 KRT20 CRIP1 GNAT1 MEG3 BCAP29 SPAG6 DUSP16 FLJ22175 PCDHB11 TACSTD2 ALDH3B2 HK3 ATN1 GSTM5 IFIT1 HRK GNGT1 MLLT1 SKIV2L SAA4 COX6C NRCAM CRABP1 EYA1 MYH3 CCND1 AGTR1 HSPA6 HLA-DQB2 GP5 C14ORF168 GABRR1 CALB1 LOC92799 KCNK1 MEP1B SP100 C10ORF57 MGP OIP106 MAP3K4 LOC221955 NFRKB CYP2A7 MYF6 ARG1 GALNT4 SERPINB10 SELP TGFB2 TRPC3 FAP ARHGAP23 KRT19 ARF4L CNGB1 NPR2 CASP6 PIAS3 RAD51L1 NTRK2 MCC PCGF5 CYP19A1 UBE4B SLC27A2 T2BP LOC90925 PRRG2 TAC1 ZNF436 SCGB2A2 ZNF313 DUSP26 ZBTB1 FOXK2 PHKG2 RAPGEF1 MYOM1 SELENBP1 SH3GLP2 /// SH3GLP1 LOXL1 PPARD IFI44L SARS NPAS2 F12 LRRC17 TNFRSF7 LONRF2 NRG1 IER3 CA9 RFC2 HR PAX3 HTR6 RHOBTB1 KLF8 CMYA5 AMELY /// AMELX GP1BA TCN1 PGA5 HLA-DRB1 HERC3 PON3 MYOZ1 PPP3CA LOC146517 KRT7 PGK2 ETEA PDIA5 KIAA0690 PRG4 COL6A3 MGAT5 CD4 IL3 SDC2 TMED5 C16ORF30 KCTD14 LOC153277 TPD52 CHRNB3 RIN2 CPN2 TOP3B OR3A2 /// OR3A3 PCNXL2 ROR2 MAGEA1 ZNF141 PHCA CXORF12 KCNA4 SLC2A4 RNF185 C1ORF21 SMPD1 HAS1 OLFML2B DUX4 /// LOC399839 / DPH5 IGKV1D-13 PRLR MPHOSPH1 IMP-3 ZWILCH MC1R CRHR1 ZNF167 CYP24A1 IRS1 MT3 TSPAN13 VBP1 ZNF239 TFF3 XPOT GUCY2F AMELY COL2A1 MAP3K14 EIF1AX CKM SATB1 /// SEMA3F IGLC1 GOSR2 TBX5 SCNM1 GALT C9ORF12 COL4A6 CHRNA6 PIK3R3 CART RIT2 RABEP1 CDH8 ERCC8 FLJ45445 /// LOC3491 GATA3 CASP9 CACNA1C CRYGB MYOG ATXN7L3 ITIH4 TTC10 UGT8 FMO5 LYZ SNX17 CP FLT1 MCFP SDS ITGA2B CYP2B7P1 ELL3 HLA-DQA1 TRBV5-4 /// TRBV3-1 PRM1 G1P3 RARB SLC7A2 VTN MAP1A HUMPPA CA4 PTGIR KRT15 ZBTB4 APOA2 ALPK3 PSITPTE22 PYCR1 SLC13A2 SERPINB3 MYL4 NPY1R CSF2RB MAP3K7 RSNL2 C10ORF68 KRTHB1 ERCC6 FN1 ART1 PDZK10 CGA GML IL8RA KARCA1 GABPA NDP CCL7 EFNA1 EDN2 GENX-3414 SSTR2 PTGER1 DNCH2 SREBF1 PEX19 AXIN1 MUC1 UMOD SEPHS1 TFF1 MT1G RYR1 PLCB4 PEX5 RARRES1 PIPPIN CYP2D6 NOS1 JMJD2B BGLAP C5R1 NR5A2 SPAR LUC7L AFF2 GLP1R DKFZP434B0335 /// GA NELL1 GRP ABCA4 PIP CYP3A4 RDH5 DRD2 SLC2A4RG DUSP4 LOC146346 TEAD4 FCAR SLC39A6 HMGCR COL1A2 ADAR /// ADRBK2 /// CYP11B1 /// CYP11B2 GLI2 HOXB1 MX1 CSN1S1 CRYBA1 FLJ20296 STMN1 COMP PPOX VTCN1

RAMASWAMY_GCM_CNS na HSBP1 C3ORF23 FZR1 MSI1 ARNT2 CTNNBIP1 PCNX NDRG4 EVI5L BLES03 FLJ10979 GGA1 DCHS1 ATP9A MAP1LC3B ADD2 GSK3B LAMA5 MGC10854 FAM82C KIAA1737 YIPF5 NUDT3 MAP3K7IP2 RAI17 GAP43 ARL2 MGC11266 GBL LYK5 TSEN34 PCSK1N RECQL5 SCAMP5 ATP6V1B2 ACTR1B MXD4 GLT8D1 VPS4A SESN3 SPPL2B PRPF8 NXF1 BAIAP1 GGTL3 WHSC1 DDAH1 PME-1 C14ORF92 URG4 FLJ13868 SRRM2 ARID3B IHPK2 ZFYVE1 ZNF84 GAPDH TXLNA APG4B HERC2 MYST2 GABBR1 PMP2 SCARB2 ANKRD40 ZNF512 PLOD3 SLC25A29 SEZ6L2 C9ORF25 CS RSBN1 CTSF PPIG ARHGEF11 GARNL1 SLC9A8 PRO0149 PHYHIPL MTMR3 KIAA0376 MECP2 ZFP106 APC2 RAB14 UCK1 RNUXA SNX15 LOC339263 AGTRL1 LOC56930 PCDHGC3 /// PCDHGB4 USP52 CIC KIAA2013 PTAR1 MYR8 TNPO1 TRIM9 FBXO9 PBX1 HIP2 ZNF677 KIAA0495 NCAM1 FADS1 KLF7 ZA20D2 C10ORF58 UNC84A REPIN1 GNAQ MGC4268 NAPG KIAA1191 SPAG9 RAPH1 GPR19 IMPAD1 C7ORF2 GCN5L2 STK11IP MGC4172 NEK11 FLJ10707 RIPK5 PSORS1C1 PREI3 ZNF161 WDR59 SSA2 ICMT PAPSS1 C20ORF4 THTPA DENND2A RALGPS1 IDS UBL3 ALS2CR4 LANCL1 VAV2 KIAA1602 AP1M1 NUPL1 ACPL2 DBN1 DDEF2 DKFZP434K1323 /// MG USP19 TRPC4AP ELMO2 C20ORF98 KIAA1450 PTPRZ1 UBQLN1 PARVB KIAA0674 RCC2 CDC42EP4 CDC23 WDR19 SEC10L1 FLJ20422 NAGLU LPPR2 IL17D DKFZP547K1113 POLDIP3 NAV2 PEG10 TRIM3 MID1 REV1L D4ST1 FLJ20308 SLC22A17 DAB2IP TTL CHD9 CNIH2 MGC4859 KLHL17 KIAA1219 GFM2 MRPL49 TNKS ZFR SCD4 VPS26B ZNF286 CLASP2 ARL6IP DNCLI2 RNF3 C14ORF100 KIAA1212 MGAT4A MAP1B MTMR9 POGK CCDC25 HKR1 FNTB TEX27 FLJ20758 RBM9 JMJD1B KIF1B C6ORF134 LOC148490 ACSL3 C20ORF43 GPR107 TRAF2 BCKDK SS18L1 KAL1 ASCC2 MAPK8IP3 B3GALT6 GTF3C4 DCTN4 EST1B TIMP2 LASS6 FLJ10826 MAGEH1 DNCI2 PAFAH1B2 CPSF2 CXXC5 NKIRAS2 TBCD PPIE STX12 UBXD1 MTCH2 MCOLN1 TMPIT AP2A1 SNX27 ZNF496 LOC152485 ANKRD12 C16ORF9 KIAA0523 ZNF692 GPSN2 LRRC49 NFAT5 STRN4 MAPK8IP1 TRAF7 C7ORF20 KIAA1193 RAC3 FLJ11273 ATP6V1A GPT2 ASB3 STAG2 MDH2 STARD7 NLGN4X C21ORF86 CRKL SF3B3 DENR FBXO21 GTF2A1 SH3BP5L DIRAS1 LASS1 /// GDF1 ANKFY1 FLJ36874 WDR6 LOC340318 MGC33302 IGSF4 FLJ35348 BMP7 KCNQ2 YWHAG USP22 RDX WBP2 TMEM43 FMNL2 DEAF1 DLG5 SH2B MSTO1 TMEM30A RASL10B MBD3 RAB18 TUSC1 DPY19L1 WSB2 MLLT6 CLU NEUGRIN MYO9A FLJ10925 COG4 SPTBN2 TBC1D10B C20ORF14 SET8 MBD1 TRIM31 TEAD1 KLHL12 C5ORF19 RAB1B CCDC3 CSRP2 CKLFSF4 SEC14L2 CES2 ARIH2 FN3KRP RABL2B SH3PX3 KIAA0515 GOLPH2 C1ORF33 FAM59A TTYH1 SDC3 DHRS3 ZNF12 ZNF404 THRA C6ORF110 MFN2 LRDD GRINA KIF13B FLJ10815 GPSM1 MAP4K4 RPS6KC1 USP24 SMUG1 DNCH1 TM4SF9 GBA2 PHF2 GLCCI1 SCN3B GDF1 /// LASS1 CCL4 DKFZP564D166 TIGD5 WDR5 MLLT4 TUBB 76P WDR68 LARP1 DKFZP564D172 GATS DHX29 CLUAP1 PGRMC2 ATG9A VPS33B TRO DHX37 CRSP6 IDS /// AFMID STMN2 NLGN2 WDR3 MUS81 TCEB3 MLL4 OBSL1 DRD4 KCTD10 WTIP TIA1 LCHN TRIO LOC338799 LOC130074 PEX14 SLC35B2 FAM11A SEC61A1 GABARAPL2 CALM1 ZNF592 RHBDL7 CAMK2N1 CPSF4 ZNF275 PHF13 TSNAX DIP13B DDX17 MYO10 PYGB PYCR2 FLJ12592 MGC9913 NUMA1 RTN4 ZNF273 TBC1D14 C16ORF49 PDXP EVI5 TU3A SIDT2 ILF3 AP3S2 ELLS1 KLHDC5 TMED7 TBRG4 ZZZ3 SFRS1 CTBP1 FLJ38984 OSBPL2 ZYG11BL NOTCH3 LOC83693 B4GALT2 MRC2 HGS HIPK2 PEG3 ARFGAP1 C14ORF35 FBXL5 FARP2 KIAA0350 COL11A2 UBE2E3 FLJ10099 CDK2AP1 THADA AAAS FBXW11 PRAF2 TNRC4 C10ORF57 PJA2 PTN PHF10 LOC339287 HUWE1 NUP133 PTPRS STK17A SLC8A2 KIAA1545 ATP13A1 PITPNC1 USP33 SLC6A8 ZNF358 NDRG2 PRO2730 SPPL3 MGC12966 P66ALPHA RERE CORO2B IL6ST NUCKS1 LDLRAP1 LOC145758 LTBP4 GPR153 KBTBD6 BTBD9 SEPN1 CCNL2 NFIB RBM21 SCAMP2 CRNKL1 POM121 /// LOC340318 MGRN1 BMPR2 DUSP26 NUDCD3 CHES1 IL13RA1 KIBRA THEM4 C3F EIF4EBP2 SCOTIN RBM8A IPO7 PGRMC1 SALL2 CIZ1 FLJ13154 NAB1 RAB3GAP2 SPRED2 C20ORF3 KIAA1018 FNDC4 NF2 PAIP1 FLJ25477 MRVI1 FLJ22471 GRIA2 RALA ZNF651 CRMP1 CHST10 SRR DHX40 KLHL18 XPO6 DKFZP434F0318 PLCG1 MAGEE1 CSNK1D IPO9 FOXO3A NEIL2 DVL2 VASH1 LOC285636 VPS24 ZNF384 WBSCR20C DERPC SPHK2 TRIM47 ARHGAP8 TREM2 VPS52 RNF41 MGC35048 GDPD3 PARVA UBQLN4 TSPYL4 GAPD MTMR2 BRD9 WDR48 NOPE SLC44A2 EP400 ZNF302 DKFZP434H1419 CA11 CLIPR-59 FLJ21128 HOXB6 RPRC1 GBAS DZIP3 MAGED4 C12ORF22 C9ORF5 PIP5K2B FLJ14566 SRP68 SPRY2 SPTLC1 FLJ21616 TENC1 RGMA YTHDF1 MTPN RHOD PPP1R16A QKI TNIK TTC17 ZNF135 LOC348262 C16ORF45 MT3 THRAP3 KIAA1559 MAGEF1 XPOT KLHL9 APCDD1 ZNF580 LPPR4 DPAGT1 GPM6A DNAL4 GOSR2 WASF3 TAF9L PLXNB2 FKRP KIAA0582 SIRT2 FOXG1B GOLGA7 APBB3 C1ORF128 ARL2BP THRAP1 SLC35E2 BCAR1 ATXN7L3 NDFIP1 COPS7A COPG ZBED1 LOC92249 FAM65A GPRASP2 VEZATIN SOX11 TNPO2 WDR33 ZNF629 WRB KLHL11 AP2A2 FSTL1 GSPT1 KIAA0431 PTDSS2 ZNF37A C21ORF33 SERBP1 C10ORF22 DNAJC11 PERQ1 MRPL10 RAN C19ORF6 KIDINS220 CRAMP1L NISCH HBLD2 RPL7L1 PHC1 KIAA1946 KIAA0310 CROT E2-230K CCNB1 MTMR4 PHACTR4 POGZ PRDM4 PFKM MGC26690 MPZL1 WSB1 DCX MCF2L MACF1 CARM1 LOC283761 DALRD3 VCP SUMF2 GSTA4 RNF26 KIAA0652 CLCN3 FLJ13111 BEX2 NCKAP1 KPNA6 SMO TMEM50B ABI2 LRRN6A UBE3A UBPH TPD52L2 ZDHHC4 USP46 FLJ10781 STMN3 IKBKAP C14ORF1 ARMCX3 PCBP4 H2AFV MGC16169 CDC25B KIAA2002 CDIPT MORF4L1 PPP1R12C B4GALT5 SPEN CAND1 GRPEL2 SELI RAB11FIP4 LOC283378 ASCC3L1 FLJ20345 GFAP PREPL ATAD3B PISD C16ORF55 ITM2C TRIM33 SAFB CPT1C TMEM29 CIT BAHD1 LOC285705 RANBP5 KIF3B FLJ10074 MAP2 MGC3207 GPR51 BRD4 FLJ37562 APPBP2 HMGCS1 DLST SIN3A PTPRG OAZ2 C16ORF34 MAPRE3 NS3TP1 C2ORF17 PIGS TRIM37 RNF11 SPIRE1 GAS2L1 NFIA EXOSC6 SDHA MAP6 COBRA1 PACS2 CAMK2B CIAPIN1 KIF5C SSBP4 PRNPIP UPF3A MFN1 SGCE TEX261 ACTB LDLR

RAMASWAMY_GCM_COLORECTAL na PRSS3 CCL11 CKS1B GSTP1 WDR43 IRF3 HCAP-H2 GPX2 AFP ADRM1 MMP2 ACADS CRSP9 CDCA3 HADH2 GSK3B ZNF593 POLR1D KRT23 RPLP0 LLGL2 HSD17B12 SH3BGRL2 MGC13170 P4HB STOML2 P2RY4 STAP2 BXDC1 GGTL3 PRKAA1 TXLNA THBS2 HSPE1 ME1 CEACAM1 RBM35A MYH11 TACSTD1 MEP1A BDH IGHA1 /// IGHA2 /// HCA112 TMEM49 CEL DNM2 S100A10 LIMK2 LTBR DES KLK10 YWHAZ SMR3A TUFM LOXL2 WBSCR16 KIAA2013 MUC13 DKFZP434D0215 MRPS35 TGFB1I1 ORF1-FL49 PPP1R1B CXORF39 BOP1 SDC1 CMAS DKFZP686O24166 KRT8 CDA FLJ20920 CDH17 INDO ALG3 CEACAM5 CCT3 CD14 S100A3 DDX56 KLF5 LISCH7 MMP7 DNTTIP1 SSRP1 HNF4A SIM1 CSE1L PLS1 NS5ATP13TP2 COX7A2 STRA13 TRIP10 LOC91461 C20ORF24 CD9 SFN HLA-DOA SEPT8 DCI FKSG24 C20ORF18 SPARC HOXB5 FLII CCNB2 CDX2 DSG2 SDC4 IMP-2 MYH14 ADIPOQ KIAA0103 SLC35C2 CDC16 COX5A EIF3S9 FLJ21742 NDUFS8 ETHE1 IL6R ATP5G3 CARS PCK1 KDELR2 PHLDA2 COL15A1 NEO1 HM13 SLC26A3 LHFPL2 SQSTM1 PCBD1 PPIC DNAJC10 MYLK COX4I1 STC2 PSMB10 PSME2 SUGT1 NEK2 PVRL3 STAT1 CDK10 SCAP DLG3 POSTN LOC113655 ASL ABCC3 LGP1 ANXA2 PTPN23 FNDC3B GPR160 IGL@ /// IGLC1 /// I TRAP1 DMD IHH COX6B1 ACTN4 TCF1 RPL41 IFITM3 /// IFITM2 C20ORF14 TGFBI MMP12 COL3A1 FLJ10769 IL32 RPS6KA1 IGH@ /// IGHG1 /// I DCPS PPBP PDZK1IP1 GRPEL1 LOC89944 CXCL3 UMPS SLC6A14 ARSB NFE2L3 DECR2 COX8A OAS1 WDR24 PERP ENTPD6 SLC25A1 GAS6 PRDX5 SEPT10 LSM1 RPS21 SLC39A7 MMP3 FLJ21908 RYK LGALS3BP SLC18A3 GEM KRT20 MUC2 C20ORF35 SNRPB CYP3A5 POLR1C DUSP16 BMS1L ADAM9 DSP DPM1 PTPRK SRPK1 GDPD5 TRA1 PLCB3 PITRM1 SOD3 ATIC NCOA6IP C20ORF52 MCP ZNF511 SEC24D TES PSMC2 KIAA0792 SDBCAG84 TIMM50 MELK XTP3TPA SH3GLB1 MGC10993 FLJ14668 CLDN3 CMIP C13ORF7 MC5R RGS3 DCBLD1 NOL5A PTPRO KLK1 EPS8L3 PFKFB1 PSG6 LCN2 ESRRA LOC127262 ZBTB1 QIL1 COX5B SELENBP1 CTSK TGFBR2 EPS8 GGCX RCV1 NPAS2 F12 MRPL18 DSTN PRKAR2A SNTB2 IGFBP4 CA9 COQ2 RHOBTB1 AMELY /// AMELX CEACAM6 MVP ETV4 FOXF1 COL12A1 C13ORF12 FADD COL6A3 GDF15 KNG1 NFATC4 SEPP1 RDBP LRRFIP1 UQCRC1 FCGBP FCGR1A ATP5D UTP15 PSEN1 FNTA PX19 TRIM29 GRLF1 HSPD1 HLA-B /// HLA-C EIF2B5 CLTA TFF3 PSMB1 MGC61571 SRI LOC129293 LAMB3 ZAK CCNE1 SERPINH1 PLA2G2A COL18A1 RASSF7 LUM DSC2 STRAP ABCB1 GTF2IRD1 POLR2H GUCY2C PDXK CYP27A1 DERA CDX1 ACTN1 PAFAH1B3 ELL3 VIL1 NEU1 SERBP1 PSMB9 MAPK12 MTMR11 TMSB4X MMP11 GBP2 CLOCK FUCA2 CTSZ CCR4 EIF3S8 PHGDHL1 IGLL1 /// LOC91316 / AP3M2 HTR7 WDSUB1 CYP4A11 GZMB ME3 CD24 TUBG1 CCT5 WFDC1 EFNA1 LDHA PRDX4 COASY GENX-3414 DSCR2 CDC25B LOC51337 SPINT2 CYC1 IFITM1 AGPAT2 CYB561D2 MT1G BDKRB2 PROL5 PLCB4 RRAS MGC:13379 TPM1 FAM3A RPS15 /// LOC440733 ANXA3 ILVBL SLC25A24 HDHD3 TRIM2 NPTX2 NQO1 FLJ20699 MYO15B MAPKAPK5 GTF2E2 MIPEP DKFZP564M082 FCAR COL1A2 FBLIM1 CDH1 EIF4E2 ATP1B1 PI3 DDX48 SEPT11 ITPR3 ACTB MYO5B PELO BZRP POLD3 CEACAM7 BENE YSG2 ACTA2 HSPC009 SLC39A1 FLNB ALDH1B1 DGAT2 SNRPC PLAU COL5A2 PITX2 NPM3 A2M LAMA3 PKLR ARHGEF5 AKAP1 LPGAT1 VDAC1 SLC26A6 TXN LOC150223 LAMC2 PTK9 MRPS18A RCN1 SLN CXCL10 CXCL14 NDUFA9 ANXA13 APOA1 /// LOC440837 GABRE EPHA1 SLC25A13 MGC61598 PON2 HSPH1 KRT18 ZFYVE19 MAC30 MET POFUT1 NEBL RPL8 CDCA5 IFI35 LTBP1 NDUFAB1 VDR HSPC111 RPS18 HEYL PCDH1 FBXO18 COL6A2 C20ORF22 CYCS MRPL11 ZBTB38 TCIRG1 TPM2 VAV2 KIAA1429 TFB1M WISP1 SP1 PSMD13 MRPL15 TPX2 CLIC1 LMOD1 UCHL3 BBC3 IGLC1 /// IGLC2 /// MOV10 USP36 HOXB7 SERPINB5 PLK3 GPNMB BCL2L14 CHCHD8 DRP2 EFTUD2 AKR1C3 PDCD2 CD2AP C20ORF149 PPGB GPR172A PHB CAPN1 GPR109B RARRES2 MST1R C1S ELF3 GBP3 MGC11242 ACY1 TST TAX1BP3 C6ORF75 NOLC1 TNNC2 E2IG5 PLVAP IFRD1 CYB5-M RORB AEBP1 RAC1 ITGB4 FGFR4 GOT2 NUDT22 GPSN2 FLJ38725 TUBA1 LGALS3 /// GALIG PIP5K1B TJP3 IARS2 GSS ARSE ARMET GATA6 OCM CGREF1 TAP1 SLC7A1 ABP1 PSMD1 RAB25 BMP7 FLJ35348 GALE ELOVL1 FUT4 CLIC5 ASS FAM50A EMP2 DPP3 GTF3C2 OLFM4 PVR SIL FH IFI27 ITGB4BP HMGCS2 SUCLG1 FTL ETFA YIF1 TFRC HSPG2 CTNNA1 COL1A1 SLC35D2 GARS FLJ10986 PRSS23 LOC440118 PRSS8 COL10A1 EFNB1 SDHB CCL13 C14ORF173 CFTR MMP1 CCBL1 EPB41L4B PARP4 CXCL1 FABP2 SH3YL1 TNIP1 EBP DDX1 PTPRH FAM38A TCTA CDK5RAP1 SCNN1B PKP2 RPA3 FAM84A LOC91316 KIAA0683 ACTG2 MRPL39 STK6 ADORA2B S100P C9ORF140 FBN1 PDE3A IFI30 MGAT1 CPSF6 PPM2C GPA33 MYL9 RNASE1 ITGA6 SERPINB6 RGS19IP1 PYGB SAP30L RPL37A ALDH2 WDR51B GGH SSB1 LOC51035 MC2R SLC25A26 LAD1 CTTN CLDN4 MTIF2 SLC35A2 ETV6 CCND1 D21S2056E NBR1 KIAA0152 PLSCR3 AMMECR1 /// LOC28650 CDK2AP1 MGLL GLB1 HEXB MGP FXYD3 MAP3K4 FBXO34 C9ORF127 EVPL ACOT7 NBL1 RBM35B FUT3 /// FUT6 C1ORF93 NOC4L SSSCA1 FAP GIPR KRT19 LEREPO4 IGKC /// IGKV1-5 S100A11 GFPT1 CAST TSPAN8 ZNF646 CTGF C1QB BOK NOSIP GPKOW IL13RA1 EIF2S2 PURB MPST NYREN18 ANTXR1 PLAUR IER3 ANTXR2 RORC ALDOA HEPH EPPB9 CSTB P4HA2 REXO2 XK BACE2 PCOLCE RANGAP1 CCNF RHOC ECE1 POLRMT IGH@ RPL31 THBS1 CLDN1 PDCD6IP F10 JRK CR2 C20ORF27 CCL20 COL6A1 FHL2 KIAA0241 FABP1 OLFML2B SLC12A2 JTV1 SMTN YTHDF1 PP PCK2 MCM8 LGMN CDW92 TSPAN13 ZNF239 PDHA2 NMES1 PRKCBP1 PTPN12 SCYL1 IGLC1 SMYD5 ICT1 BMP1 FAM57A FCGR3B POLR2F TAP2 MCAM TAGLN MRPL17 TDGF1 LGALS4 PLN CYP3A7 /// CYP3A4 // CLCNKB ERBB3 DPPA4 MTF1 MAL2 GAL TMBIM1 SNX17 RARS VWF DGCR2 RGAG4 FLJ10094 HLA-DQA1 LOC151162 GPRC5A GSPT1 NEK3 NSDHL AHCTF1 SATB2 PSITPTE22 INHBA CCNB1 HRASLS3 AGMAT CKB RAE1 EIF2S1 LOC120224 PCCA KIAA0963 KRTHB1 BLOC1S1 FN1 ITGB1 COL4A2 MICAL2 CKMT1B HSD11B2 LOC58489 CDH3 C2 LRRC32 FLJ13576 HLA-F AOC3 CYP51A1 LOC112714 TFF1 SEC13L1 STK38L CXADR C1R KPNA2 ITGB5 ACVRL1 FUT3 FAAH CIB1 TJP2 TRIB3 IL20RA MYH9 BIT1 C13ORF25 DDC PIP EVI1 DPEP1 DRD2 DOCK6 NGFB RXRA TEAD4 SAH DAB2 PSMA5 HOXB1 CKMT2 QPCT COMP

RAMASWAMY_GCM_LEUKEMIA na HCLS1 GNA15 AVPR1B AGER CENTB1 PRKACG CD5 PSMD11 TOP2B RENT1 ADRM1 RPS2 PIK4CA NFATC1 RPLP0 P2RX1 RANBP1 SPRR1A HSU79275 CHKB /// CPT1B SUMO2 TLE4 PROC SPN IKBKE CSH1 B4GALT1 POLR2K ELA2 MFGE8 DUT PRKCB1 TPM4 NXF1 PPP2R5A AIP SRP19 MYD88 RPS25 GAPDH PRKAA1 ARID4A EFNA3 TRIM32 BTN2A1 MRCL3 IK SYMPK SFRS2 SNRPE LIG3 MECP2 NUDT1 YWHAZ HMMR RAB7 RPL14 NOS2A SLBP TWIST1 PTGIS MPP1 ZNF43 HHEX ENO1 HMGA2 RPL3 RBM6 RAD23A TMED2 MYB NRAS RPL18 RPS16 MADD ADK UPK2 GUSB TGFB1 ANP32B NASP HNRPA1 CD53 NR3C1 SLC25A5 RPL13A PNN P2RX5 SEC10L1 BCR FLJ20232 KCNJ4 HNRPM HSF2 FES CD69 VASP C20ORF24 GAMT NPM1 RPL32 HLA-DOA CCR3 MIB1 USP11 DDX39 CCNH MCM5 TUB CHD1 AANAT PSMB2 LOC132556 TCOF1 PRKAG1 PSG7 VAV1 C14ORF166 SATB1 PSMB6 AKT1 GPS2 RBBP8 SP2 GCDH ZNF136 DSC3 SNRPA HSD17B3 BNIP1 PPIL2 PLD1 SMARCD2 C1QBP PRKCG CDCA7L COX10 RPSA /// LOC387867 / DYRK1A MATK TLN2 PIK3CG FUBP1 DRAP1 RPL28 NR1H2 GIT2 PDE4A SUPT4H1 DHX9 PFN1 TH GPSM3 TNP1 TPP2 OSBP PSG11 VHL ELF1 LGALS9 BLMH BCL7B RPS29 MYLPF RBM33 CPN1 DRD1 RPS7 HTR1B TPT1 ITSN1 GJA5 FUT2 RIT1 NUP88 MAPRE2 GCGR TUBB RSU1 CLCN7 KIF5B NQO2 RENBP HNRPA3P1 /// HNRPA3 SMG1 GNAS EIF3S5 UROD CCND3 RPS21 RPS3 JMJD2A MLN SUMO1 H3F3A SH3BGRL3 BAT1 TLK2 ZNF592 HTATSF1 CCL2 NDUFA12 TRH ATP5C1 TRIP13 MYCL1 JUND CKAP1 SNRPB BAK1 GPX1 PRH1 /// PRH2 PPP1R10 ARHGEF2 WTAP POLG ALPI TNR EIF5 MYST3 CHAF1A PAPOLA IL2RG RHOA SUI1 TIAL1 CDKN2D RGS19 WBSCR1 COL11A2 TAGLN2 SKB1 RPS17 GTF2B CAT H2AFZ KHDRBS1 PCM1 GDI2 USP7 GNB3 HNRPL TERF1 DDX21 MYO5A FXYD5 CUGBP2 TTC1 SFPQ SFRS2IP ZNF142 KLF6 IDH2 PSMD8 ITGA4 HCAP-D3 PLP2 RPL19 PFAAP5 ADRB3 CGB /// CGB5 /// CGB TRIM15 CCL22 STAT5B RPL9 HBA2 SMC1L1 CHERP ANP32A THRSP MGC20235 CDCA4 RPL38 ALPPL2 CSNK2A1 FXR1 KIAA0101 SNRPA1 ARHGDIA NTHL1 H2AFB3 /// LOC442472 ZNF74 PIK4CB C1ORF16 H1FX ELK3 CRYAA VIM STX4A EEF1G RPL34 PLCG1 SFRS9 RSC1A1 DEF6 S100A9 TBC1D5 PNMT RPL18A ZNF306 CCT2 MPP2 ZNF8 SEPT6 BRAF CTCF MPG TRHR CYP2F1 TCEA1 RPLP1 EEF1B2 AQP2 PHKA2 SLC2A4 SFRS11 ADA CPNE1 EEF1A1 SYBL1 MYLIP VAMP1 NMI HLA-B /// HLA-C CCR9 GLA CPSF1 UBAP2L MYH7 MDC1 PSME1 RB1CC1 JAK3 BCAT2 NAP1L1 RAC2 COPA ZFP36L2 NUP62 MAP1A DVL3 HNRPC TMSB4X LRRC6 RALY HEM1 KIAA0143 WAS AAMP RPL11 DHPS RET HINT1 PDE7A F2RL1 BRPF1 UBE2L3 TEF ZNF132 RPL27 RPS24 FBL HLA-A FANCC SMARCD1 SCNN1G AMHR2 RPL7A FKBP8 DLST /// DLSTP MRPS12 PPM1A FUT5 BCLAF1 RPS6 ATXN10 ESD CLK2 ADORA2A GPR30 EEF2 LCP1 RPS15 /// LOC440733 ARRB2 TRIP3 INPP5D EIF4B RPS28 CELP RPL6 RPL23 PIK3CD PDGFB RAB32 C10ORF7 RNPS1 PLG E2F1 RPA2 ACTG1 RBM34 EIF4A1 MVK GPR3 STMN1 ADD1 GABPB2 PSMA3 MAP2K5 HSPA14 ACTB BRD2 PTEN TSTA3 H3F3B SSR1 LILRA2 ILF2 CDK9 MMP19 ITK PRPF38B RPL21 COL16A1 M11S1 MLL5 RNF113A PKLR HIPK3 ARHGAP4 ASMT SFRS4 ALDH5A1 PLCB2 ITGB3BP CALML3 EMP3 PIB5PA ERBP IRF1 PSMC3 KRTHA5 RPS23 H2AFY NKX2-5 FRAP1 PRIM1 HDAC1 UBE2D3 TKT TCP1 STIM1 FNBP3 MSN SF1 SET RB1 TK1 UBB TACR2 H3F3A /// LOC440926 L1CAM TMPO PPP4C RPL10A ARF1 NFATC3 RAB33A RBM15B PTCH TRA2A RPS18 AQP4 NAPG UBE1L TTF1 HSF4 DUSP9 HIST1H2BG ARHGEF1 ZNF76 SMARCC1 SNRPD3 ATP5F1 TMSB10 IDS RPS5 SRM STXBP2 MTMR1 BFSP1 MAPK3 ZNF266 SERTAD2 TBXA2R HMGN2 CLIC1 KIAA0195 P2RX3 MATR3 RING1 NUCB2 GTF2F1 PAICS RPLP2 OAS2 RPSA SFRS5 AGPS CDC2L5 ZNF211 CSDA RPS11 RELA ENTH CXORF40 /// LOC54157 RPS27 /// ZFPL /// R ST3GAL1 NEFL FNTB UFD1L PSMA6 ITGB8 CLCNKA KIAA0226 GLTSCR2 MAZ MST1R ACTR1A MARS TP53BP2 DOCK2 CUTL1 HLF AMPD2 STK38 MGC11242 GRB2 RPL12 EMD SLC38A3 SP110 DRG2 FXN PIN1 NACA PLAC8 ZP2 RPL39 ST14 LTK AQP7 U2AF2 FGF4 PMS2L11 RPS3A CASP8 GSTZ1 MCM7 GCK DGCR6 RPL36A SFRS10 SLC29A2 RAB8A NUP188 PCBP1 SLC7A1 LAT1-3TM MFNG APOC3 SIRPB1 EBI3 RPS27A KRTHA2 EVI2B NR2C1 MAPK14 DDB2 /// LHX3 TIMM17A SNRPF HSPA9B NGFR UBE2N BCL3 RNF12 CDKN3 MSX2 /// MSX2P FAH TCF3 ZNF131 GPR31 EIF5A /// LOC143243 MYO1F DDX5 RAB35 TRIM28 SSTR1 OAZ1 RPS9 B2M EIF4G2 LMNB2 GRIK3 MCM3 CSNK2B SLC25A3 RUNX1 PTPN5 ERV3 NOS3 HNRPH1 LDHB CSN3 HNRPD EBP INPP5E NCL ATP5G1 ATF7 KCNMA1 HA-1 LAPTM5 PDE3A PKN1 RPS15A LYST MRPS27 ABCC1 HTR2C TAF1 TAF11 TYMS PLK4 FCGR2B FUS DHX38 MNT TYK2 CHKB HCFC1 PTPN6 RPS26 /// RPS26L /// AKAP13 GPR68 KIAA0082 PHB2 MYH10 ANXA6 RPL27A BCL7A ADSL CHRNG EZH2 RABGGTB C4ORF8 PMS2L3 PPIA DDO ETS2 HMGB2 PRPS1 HRMT1L2 RBM3 PDE6B WWOX DYRK2 RPS19 HBB /// HBD HUWE1 STK10 NUTF2 SFRS3 SERPINB10 PSMD6 PABPC1 HMGA1 RPL23A /// RPL23AP7 ICAM2 QARS NCF4 TCF20 BAP1 TRAM1 MRPL12 SIGIRR BECN1 STX16 GYPE ODF1 RPL4 PTMA ATP5B SNRPD2 C3F AMFR DDX3X SAP18 RABGGTA GNB1 RPL7 ATXN2L RPIA ATM PIK3R2 TRBV21-1 DEK HADHB SCYE1 SHMT2 TNNC1 AMPD3 IL1A DLX4 DNAJC7 SLC25A6 MYBPC2 RPS10 OXA1L RPL17 LOC23117 /// DKFZP54 TAF9 KIF11 HMGN1 ETV2 RBMX YARS PCNA HNRPA0 KLHDC3 CFL1 GZMM GMFG GAPD SAC3D1 FEV RPL31 TXN2 THBS1 UBE2I GOLGA4 ELK1 SUMO3 CD36 PIP5K2B COL14A1 SLC16A1 ZNF177 APEX1 HERC2 /// LOC283755 FAM53B FSHPRH1 MPV17 ACHE OPRS1 PCK2 RPL35 GFI1 MCM6 RPL29 RPL24 /// SLC36A2 LYL1 EIF3S6 EP300 CBFB DPF2 GCM1 IGLC1 PSMD9 OMP HNRPU DR1 CCT7 PDE6G BMP4 PLK1 NSEP1 CLCNKB U2AF1 TBCC ZNF137 NONO ARHGDIB ZRF1 NUP93 HIVEP1 TSC22D3 HAS3 ARAF RAF1 ZNF37A ATP1B3 CXCR4 PTGES3 JAK1 JMJD1C PTK2B FAD158 GRM4 RPS14 RPS8 GNB2L1 PSMA1 PSCD2L LOC440836 CBX3 NP DNMT1 SEMA3F MEN1 TFE3 APRT CLCN1 HSPCB H2AFV HPRT1 RRS1 SMN1 /// SMN2 RPL35A HTR1E ADH6 PTCRA WNT2B ATP5O HD MICB DHX34 AIF1 PEX5 ZNF134 GSTO1 C14ORF11 MGC2803 UBA52 PCBP2 PTPN1 MAPRE1 TP53BP1 MT1A IDUA ARF6 DGKZ SLC6A11 SRP14 PSMA5 CSNK1G2 UBE2C RAB5A CREB1 CYBA ITGAL

RAMASWAMY_GCM_LUNG na TACC2 MOXD1 AGER EPHX1 WDR43 ALOX15B BENE C1QR1 SOCS3 PAK3 LOC55831 DRD3 CDKL1 HSPC009 FOXE1 GPX2 CTSD SLC39A1 RENT1 MMP2 LMO3 CRSP9 HYAL1 RAPSN KYNU DAF KLHDC4 CNIH4 ZNF593 PLAU PITX2 CLDN5 CLIC3 YIPF5 A2M ZSWIM3 ANAPC11 CDC27 CAV1 KIAA0368 HMGB3 EXT2 C6ORF109 HLA-DRB1 /// HLA-DRB ARMC9 ABCA3 TUBB4Q SLC26A6 KCNJ6 RAB27A P2RY4 TXNL5 C9ORF88 ARHGEF15 ACRV1 STAP2 CD63 LOC390660 GGTL3 GPR116 ERO1L MRPS18A C14ORF92 C4ORF10 LRRC4 PODN LOC125150 PLXNA1 ALDH3B1 GAPDH PRKAA1 PPCDC HSDL2 ME1 SFTPA1 RBM35A SLC8A1 CDK2AP2 G6PD IGHA1 /// IGHA2 /// MRC1 TMEM49 FLJ34443 LIG3 PGC FLJ12443 DUSP7 CEBPE SLC27A4 LPL FLJ23186 ABLIM1 LOC283501 NOS2A PON2 LOH11CR2A MRPS16 ZNF143 DHRS6 PIR BMP6 ISYNA1 FLJ43339 ELF5 DKFZP434D0215 TGFB1I1 LTBP1 VDR LOC154761 GRM1 SMARCAL1 SCIN SNAPC1 GPR19 CYB5 PSORS1C1 SLC7A11 NEDD4L ADK KRT16 DKFZP686O24166 MGC17299 LOC90557 /// DKFZP43 VPS16 IL2 WISP1 CLEC14A PAOX ARC FGF2 CNOT2 DNTTIP1 HNF4A C7ORF27 DKFZP547K1113 GP1BB IGLC1 /// IGLC2 /// MOV10 USP36 COL21A1 CCPG1 GCKR FGG COL19A1 AGA TCN2 SPUF SLC17A4 C8B GPNMB STOM FLJ22386 ADH1B XPNPEP1 IER3IP1 CNTFR AKR1C3 LOC286044 ALDH3A1 C20ORF24 ERVK6 ARG99 GNRHR2 ZFR SFN MRPS31 SCNN1A MGC17624 EGR2 ANGPTL2 LOC126669 JDP2 CYP2B6 MTUS1 HTRA3 C14ORF125 C18ORF1 PFKFB3 DSG2 SDC4 SLC39A3 C1S PPP1R12A MGST1 TAPBP MGC11242 LOC399884 HPS5 TCTEL1 TAX1BP3 EDNRA TFPI KCNC4 SHBG ZNFN1A4 SFTPA1 /// SFTPA2 KIAA0828 PHLDA2 MMP10 C14ORF147 IFNA16 TITF1 AGGF1 CX3CL1 CES1 APBB2 TNFRSF1A MLPH C16ORF9 DNAJC10 GPS2 ERBB2 BCL6B ACP5 CHD3 PCSK7 RCE1 NEDD9 CBR1 MGC34646 LGALS3 /// GALIG CHST9 ADH4 F2RL2 OPCML FLJ40432 APBA2BP SFTPC APOC1 DYRK1A PHACTR3 ABP1 LOC112869 RAB25 KRT5 FLJ35348 POSTN LOC113655 ABCC3 AADACL2 TARBP2 ABCA10 KCNJ8 FOLR2 SMPDL3B IL4 SLURP1 PCCB PTPN23 EMP2 FNDC3B MSTO1 C14ORF24 IGL@ /// IGLC1 /// I ABO KIAA0040 TSPY1 /// TSPY2 /// IFI27 TMEM4 RYR2 FGFBP1 FLJ13352 PIGV FTL IFNB1 LOC54103 MMP12 EI24 FLJ37440 SERPINA1 ADH7 MAOA CISH IGH@ /// IGHG1 /// I C14ORF149 POU1F1 HLA-E COL1A1 BLVRA SOSTDC1 MSTP9 PRSS23 GRPEL1 UBE3B FST COL10A1 ARHGDIG FMO2 CNKSR1 POLR3G RAB31 CCL13 MMP1 SLC6A14 GPR4 ZXDC SLIT2 COL11A1 C18ORF25 DUSP1 VAC14 FGF8 KIAA1295 HNRPD ARID5A CYBB RAI14 DJ222E13.1 PERP MAPRE2 SCNN1B AKR1C1 MOSC1 BAG3 NR0B1 SLCO1A2 LOC255458 SFRP2 MRPS21 C2ORF12 PRKAB1 CGN LOC91316 CCR1 CTSH ISG20L1 EDF1 ARRDC1 STK6 SPRR1B MPZ CA5BL PGBD3 LLGL1 LOC89894 FER1L3 IFI30 GOLGA2 H17 PRKG1 FKBP3 KRT6A CEBPD NKAP HESX1 RNASE1 FLJ32810 ORMDL2 FUT1 C1ORF116 TXK NRP1 RGS19IP1 MGC42090 ALDH2 SFTPA2 CAV2 FLJ20273 TATDN1 GRK1 GGH B4GALT4 NTS CRYBB1 DERL3 GDPD5 AKAP13 UNC13B HAPLN3 CLDN4 MYL7 MCRS1 FLJ11785 CCL18 EYA1 SLC25A14 HSPA6 CENTA2 DHX32 WIPI49 EGFL7 SEC24D ESAM ZBTB33 LOC90806 SLC24A6 GCLC BAZ2A SDBCAG84 SC65 GJB2 ASAHL ABLIM2 LOC221955 SENP2 HABP2 FAM46C RBM35B DST MGC11335 INMT ALCAM UAP1 RPE65 RGS3 SLC6A8 GIPR DLC1 PLA2G10 ALDH3A2 IGKC /// IGKV1-5 FLJ20160 MAN2B2 MRPL12 UBE4B METTL1 SFTPB PSG6 EDG2 KIF1A SERPINF2 GCLM PRRG2 RGS14 ZNF436 BOK PHOX2B POLR3F RAPGEF1 FOXK2 PHKG2 SELENBP1 PPARD GLS ICAM4 GGCX SPIN2 SH3BGR NF2 CYP4B1 ADSSL1 DNASE1L2 ITGA2 PLAUR HPD C1ORF91 ITGA8 GRK5 DKFZP566N034 PRSS16 SULT2B1 COL7A1 CMYA5 TBXAS1 CEACAM6 REXO2 MYOZ1 PON3 LOC146517 CHPF SCGB1A1 COL12A1 C4BPA FLJ20035 PHF20L1 COL6A3 MGAT5 C6ORF89 ECE1 MDFI MGC5987 MGC25181 IGH@ C16ORF30 KCTD14 GAPD LOC153277 ZP3 /// POMZP3 CHRNB3 HTN3 SEPP1 LRRFIP1 RBMS3 FCGR1A PHCA ARFIP1 PRB1 /// PRB2 PSEN1 MBL2 CXORF12 SLC45A4 SMPD1 TALDO1 OLFML2B ADH1A /// ADH1B /// IGL@ GRLF1 LRP16 TXNRD1 CAPN2 CLEC1A LOC92305 CYP24A1 LGMN UCHL1 C17ORF27 TSPAN13 DDOST ZNF239 SITPEC NNMT CFH /// CFHL1 UGT1A10 /// UGT1A8 / LAMB3 LOC283345 SCYL1 SCNM1 DKFZP686L21136 C20ORF17 BMP1 C9ORF12 FAM57A CHRNA6 LUM MCAM IGHD IGFBP2 MRPS14 PHLDB2 MMRN2 AYP1P1 FLJ45445 /// LOC3491 DPPA4 MTF1 DERA TMBIM1 PLEKHH2 KIAA0556 FMO5 VWF DGCR2 CYP2B7P1 PMP22 LOC90624 GPRC5A PRM1 GSPT1 MNAT1 LOC255743 C10ORF72 KRT15 COLEC12 GMEB1 ALPK3 FUCA2 TXNDC5 INHBA FLJ20487 ICAM1 PTHLH C20ORF44 IGLL1 /// LOC91316 / NPY1R LOC120224 NPC2 UNG2 ACVR1B GM2A PRODH2 FN1 SUMF1 FLJ13111 GRB14 RPS6KA2 LOC92689 GML TP53AP1 MICAL2 HGD YIPF2 MYOD1 C1ORF56 TTC15 FOLR1 PTGER1 MAPK13 C2 BCL2 DKFZP761A132 CELSR1 LOC51337 MUC1 ABCC6 AOC3 CPM PHF7 DBT PITX1 HD C1R ACOT2 C9ORF58 IL1RL2 JAG2 ACVRL1 GULP1 FUT3 ZNF193 FBP1 RABIF C5R1 NR5A2 LAMP3 TNRC15 DHCR24 CABIN1 ATP11A VPS39 KRT6B MMP13 NELL1 FLJ37562 IGHM MSLN PKP1 EVI1 RDH5 NQO1 FMO3 IDUA C16ORF34 SLC2A4RG DOCK6 CYP1A1 RXRA CD59 FLJ14154 DKFZP566O084 FCAR SAH RAB6IP1 COL1A2 ARNT WDR25 ATP1B1 PELI3 PPOX ITPR3 SGCE ACTB

RAMASWAMY_GCM_LYMPHOMA na FIBP C3ORF23 FZR1 CWF19L2 LGTN CENTB1 CORO1A PCNX MDS032 ALG1 BLES03 LOC283578 LOC388962 PPM1M CD209L CPSF5 FOXE1 ITK RPS2 FADS3 NFATC1 HADH2 BUB3 CNIH4 FLJ10980 EML4 RAMP3 NDUFA11 BRF2 MGC14288 CD48 C15ORF20 KIAA2018 ARL2 CKLFSF6 COMMD2 KIAA0409 EIF2S3 HEATR1 SYK RECQL5 RNGTT CCL19 STOML2 C1QTNF6 VPS4A SNAPAP PTPN7 TXNL5 CNO UBL5 CR1 GGTL3 C6ORF149 CXCL10 MGC3123 SNX5 MRPL40 RPS25 NIN APOA1 /// LOC440837 GAPDH PDK3 AHSA1 PARP14 XRN1 MFSD1 VRK3 ATP5H DNASE1L3 TIMM10 ARMCX6 GRINL1A CDK2AP2 RPS27 C2ORF24 ARHGEF11 SIPA1 IQCB1 CKLFSF3 FLJ14681 C9ORF105 SSNA1 MUC15 PHF11 NECAP2 BIK SCAP1 RBX1 CXCR6 MRPL53 MEOX1 KIAA0922 SFMBT1 CYP2U1 C1ORF90 C10ORF45 NFATC3 NEK6 TCF21 CECR5 BATF NOD27 RPS18 CD74 SPRN CHAD FOXI1 EMID2 RPS16 WDR59 ICMT SH2D3C HSPC142 CD8A FLJ34077 OSBPL10 PARP9 RPS5 KIAA1463 VAV2 MOSPD2 CCL21 TFB1M IL2 PUS1 HLA-DPA1 TBL1X CD53 MSL3L1 CCDC17 PARVB NDUFB3 NUP37 SLC25A19 RCC2 PRKD3 HLA-DQA1 /// HLA-DQA IGJ C9ORF112 NDUFB1 MGC4859 VPS25 C19ORF24 EVER1 TRIM5 TXNL1 CYHR1 MGC42630 GFM2 RAFTLIN PPCS MRPL49 MGAT2 LOC284385 PTE1 RAP2C CXCL9 BCL2L12 PSMA7 TAOK2 RPS11 HLA-DQB1 MRPS15 SNX10 ATF5 PTPRC CCDC25 SLA AKNA UNQ467 ANKRD47 NDUFA1 SOCS1 MAP2K7 GPR18 HLA-DRA BIRC3 ARPC5 IFNG GRB2 FLJ21865 SELT CCR7 CD37 MGC2477 C3ORF1 MHC2TA IFNA14 RHOH FAS ALOX5AP ZNF581 EPB41 LIMS1 PI4KII C22ORF13 CCNE2 MRPS18C ADAM19 PPIE AZI2 UBXD1 MIS12 C14ORF166 ANKRD12 C6ORF49 COTL1 SEMA4A NUDT22 TFIP11 LSM7 SCAP2 FLJ22405 TRAF7 MASA MRPL35 C14ORF123 PRRX2 SF3B5 IL4R ITIH1 APOL3 LAP3 CCDC5 ASF1A TRAF5 C21ORF91 NBS1 CAP350 MCTS1 APOC1 STAT1 TNFAIP8L2 WDR50 LOC91526 LYPLA1 HSPC152 ALS4 PKIG HSPC196 LRMP MGC33302 MFNG ACTR6 ATP5J2 CHMP7 RPL28 SLC9A9 SLC22A6 ZMAT1 C9ORF46 HT007 GNL3 ZDHHC3 MAP4K1 CRISP1 MPEG1 TOR3A CD79B ETS1 MYO9A FAM96B APOBEC3C MED25 SPIB RPL41 C9ORF37 FTL ANAPC5 PSG11 LOC54103 HSPA4 SRCAP DTX1 TXNL4A SYTL2 FLJ37440 SDHD DCPS SECP43 B2M ARIH2 UBE2L6 CCNDBP1 HLA-E HBXIP C16ORF33 LOC389203 KIAA1949 SLC35B3 WIBG KIF13B LY64 SLC40A1 LYPD3 CIB2 NSUN4 C14ORF58 MMAB FUCA1 ARID5A LOC257106 FLJ21749 ATOX1 HLA-DMA CYBB NFKBIE WDR5 FLJ42117 STK4 NTNG1 GIMAP4 LOC255458 KIAA0476 FLJ10652 STX7 RPS21 CTSH DHX37 ADH1C /// ADH1A /// PNOC MS4A6A WDR45 BLM AMSH-LP MOBKL2A PLEKHA2 CD180 SYNGR2 ISG20 IRF4 NOL7 CORO1B CWF19L1 MKI67IP SIAT7D NKAP CPSF4 SARA2 HCCS PIM2 PAPD4 CUTC PTPRCAP PYCR2 FLJ33814 FBXW5 LMAN1L MEG3 C2ORF29 BAK1 AMICA1 IRF8 FLJ22318 DNA2L RFFL DTX3L ERCC2 ARL5 MS4A1 FLJ20625 TMBIM4 DUSP18 CCL18 ATIC LOC284336 OTUB1 WDR1 SNX1 OMD BTN3A2 BTN2A2 EYA1 PSARL IFNAR2 C17ORF42 SLC15A3 CDCA2 KRT1 CXCL11 SKB1 MGC4368 SUV39H1 CTSC CBARA1 RSAFD1 OGT LOC92799 ATPBD1B TNRC4 LOC51315 PSMB8 STK10 CRLF3 SLC38A1 SENP2 STK17A PTTG1 NPL CKIP-1 RBP5 SLC15A4 FLJ14981 MTP18 GGA2 IGKC /// IGKV1-5 MMP9 LDLRAP1 ZA20D3 MBP ATP6V0E LY9 TIMD4 MGC5508 LOC64744 SEPN1 VCX2 ACTR2 MBD4 GRHPR PARP12 PTGDS L3MBTL2 KRT2A SCAMP2 UBE2W RBBP7 CD2 BCL2A1 GA17 UBE2E1 IRF2 MGC72104 IL13RA1 FLJ12118 FAM13A1 TFEB GLS CCRL2 ARPC1B FLJ11171 MGC33488 KIAA0494 ABT1 RAB3GAP2 C20ORF3 C1ORF24 PDCD5 MADCAM1 POU2F2 CD19 MALT1 HLA-DPB1 ARHGAP25 NPAL2 CYB5R2 RABAC1 LILRB5 /// LILRB3 // CD1C DKFZP566N034 EDG4 RANBP2L1 KLHL18 C14ORF150 SH3BP5 HGFAC MGC3731 NAGK DPM3 DEF6 C2ORF25 RPS10 ADRB1 HLA-DRB1 COBLL1 LOC113386 /// ZNF8 PRKCQ KIAA0746 GZMH RPL18A CD52 WDR75 CTSS TREM2 CD4 CCNF MPG GZMM FAM49B TMED5 ZNF212 IGH@ GAPD VAMP5 TNFRSF9 ING1 ALG8 CHRNB3 RPP38 CR2 LOC51255 CSTF1 DAPP1 LILRB4 ZWINTAS LYPLA2 SNX11 SLC7A7 WNT10B CXORF9 NT5C2L1 PX19 EIF3S4 EEF1A1 PP ARPC5L SCYL3 DUSP22 STRBP HTATIP2 HLA-B /// HLA-C C20ORF100 GZMK PSG4 /// PSG7 THRAP3 EIF3S6 KBTBD4 MEF2B TINP1 SOX18 STYXL1 POLE4 CORO2A TOMM22 NDUFB9 GOSR2 C3ORF10 SERF2 SNX8 LTB SPG21 MRPS14 RUVBL2 C14ORF83 RASSF5 DDX26B G3BP DC13 CYP4F12 COPS7A PSME3 GABRR2 TMED3 HLA-DOB HLA-DQA1 MGC17330 PTDSS2 MGC50853 ACTR3 ANKRD13 NCBP1 IL2RB C15ORF12 PSMB9 DBNL MRPL10 NCF1 TMSB4X SGPP1 KIAA0342 ASF1B TXNDC5 CTSZ HLA-DMB KIAA1644 TRAF1 TNFAIP8L1 TRA@ /// TRDV2 /// T PRDM4 POLR3GL MGC26690 ERBB2IP EHD1 PI16 DENND2D TRAFD1 NCOA3 GM2A MDS010 NALP2 EXOSC2 PPIH USP39 SP140 KPNA6 GZMB BMP2K MGC21874 HLA-A SEC22L2 ZNF672 PEX11B RAB8B FLJ12903 ATAD3B /// ATAD3A MYBL1 MRPL28 SS18L2 FGL2 RNF34 AK3L1 OSBPL3 SEMA4F GEMIN7 MAPK13 KSR UGCG PORIMIN FAM96A MRPS12 MBNL1 MAT2B WASPIP HLA-F SUCLG2 MLLT7 RAD1 CD79A RAB11FIP4 ATP5G2 MTCP1 BLR1 FLJ20345 TXNDC10 EVI2A MGC20446 RABIF CIB1 FAM69B IL16 GIMAP5 E2F5 LOC91801 H41 MTHFS LSM6 C1ORF54 IGBP1 COMMD8 C9ORF123 FOXO1A NS3TP1 NHP2L1 HSPC138 ARPC3 FLJ10803 EIF2B2 BCL11A INPP4A PHF23 RIOK2 COBRA1 CCL5 IL18 CTPS2 DKFZP564J157 ZNRF2 APG12L BAG2 FAM72A ITSN2 NR1H3 MFN1 ACTB

RAMASWAMY_GCM_MELANOMA na RNPEPL1 LRRC14 AVPR1B APOE KIR2DL3 MDS032 LOC55831 C8G C19ORF28 CDKL1 RCBTB2 TROAP TGFBR1 CNIH4 ZNF593 DCT ANAPC11 CHKA SORCS1 ADCK1 C6ORF192 STOML2 MFGE8 NRP2 BAT4 BXDC1 TDRD3 CD63 GGTL3 SNX5 TFAP2A GAPDH THBS2 PLOD3 ME1 MGC90512 GPR143 PSMB7 TMEM49 LCN1 KLP1 MECP2 EMP1 ZFP106 DUSP7 SLC27A4 LTBR TGDS LOR LOC113251 KIAA2013 TWIST1 DOC2B FLJ43339 PDE4DIP MGC16037 MAP3K11 TGFB1I1 RAD23A NDUFB7 CHI3L1 GALK1 SMARCAL1 BOP1 TRIB1 MYO9B PAF1 RIPK5 TAZ PRKAR1B AZIN1 ETV5 CSPG4 RRAGC ALG3 TRPT1 IL2 NDN AGPAT6 LRPAP1 CCT3 GJB1 YTHDF2 DDX56 PARVB HSPC117 CHMP2A THAP7 HNF4A IFNA5 UPP1 NAV2 TRIM16 NS5ATP13TP2 NDUFV1 ADAMTS9 C9ORF112 CYB5R1 ERCC1 KIAA0859 TRIMP1 VAT1 ERVK6 GNRHR2 VARS2L ITGA9 HLA-DOA RBPSUH LHCGR DNAJB11 C6ORF206 SLC35C2 LOC148490 SLC39A3 DLEU1 G6PC3 FLJ21742 C16ORF5 CARS LOC166994 /// LOC340 PPM1F ZNF584 ADARB1 CCDC28B LHFPL2 TM7SF1 LCP2 MLPH GPS2 HEG1 SLC20A1 TFAP2B RAGE SALL1 APOL3 MGC34646 GPR37 GPAA1 MAD1L1 FLJ40432 LOC400581 MAPBPIP STAT1 CDK10 MRPL4 RXRB RPL10 IL6 TMEM51 LOC113655 TARBP2 LGP1 WDR13 NTAN1 ATP4A ANXA2 PTPN23 CGI-14 ABO COX17 FAM54B IHH S100A13 ZNF16 C20ORF14 TXNL4A SPP1 MGC3329 DCPS RUNX3 DDT MAG GRINA SERPINA3 FUT2 DXS9879E D4S234E NFKBIE WDR24 PES1 ENTPD6 NR0B1 LOC255458 MRPS21 COG6 AD-003 TBC1D16 SLC4A2 PNOC SPRR1B LLGL1 ABCA2 SH3BGRL3 IRF4 RHBDL7 LGALS3BP TLE3 MAGEA10 C6ORF125 ABCA6 POLR1C CNIH NDUFB10 ARHGEF2 LAG3 PITRM1 HAPLN3 MYL7 ICA1 P2RY5 C20ORF52 EIF3S6IP PARD6G C9ORF76 SPON2 KRT9 MGC4368 SLC27A3 SDBCAG84 OTUD4 SC65 LAMA4 MGC10993 MYO5A ELOF1 FXYD5 C13ORF7 RGS3 TGFB2 EHD2 MPDU1 MTP18 PLA2G10 NOL5A GHRHR CRELD1 POFUT2 SEPN1 CGB /// CGB5 /// CGB DLX2 LOC127262 OSTM1 MGRN1 QIL1 SCO1 CTSK FAM82B C19ORF25 ICAM4 MGC33488 MITF RRM2B LRRC17 TNFRSF7 ADSSL1 NIFIE14 HPD HR PLAC9 HPS4 CMYA5 TBXAS1 PPP1R1A DKFZP434E1822 GSTT2 DDEF1 PRG4 C6ORF89 MLANA MGC25181 GDF15 RDBP LRRFIP1 TOP3B RBMS3 FCGR1A ATP5D PSEN1 AGXT CSN2 FLJ12438 C11ORF31 PX19 GRLF1 EIF3S4 HIST2H4 CLTA ABCB4 ADAM2 RXRG ATP5I COL2A1 CPT1B TBX5 ZFYVE26 EIF3S12 MGC23280 S100A4 KIAA0446 CACNA1C CYP27A1 DERA PET112L COPS7A PS1D ST6GALNAC2 PTGIR NISCH OSBPL9 FUCA2 THRAP5 MGC16733 PROS1 RAP1GDS1 IGLL1 /// LOC91316 / AP3M2 CYP4A11 KIAA0831 GAS7 LZTS2 C2F HMG20B YIPF2 UQCR C14ORF1 GPATC4 IL12B UGCG DKFZP761A132 CDC25B WASPIP CYC1 VARS CYB561D2 C21ORF6 SLC25A32 BCL7C NOL3 CPSF3L JMJD2B C1ORF166 SLC11A1 S100B CDK5RAP2 DYRK3 LOC149705 TMEM16K SHC1 MIPEP CD59 ENPP2 DKFZP564M082 SLC39A6 GJA4 GLB1L LARP6 FMNL1 MRPL24 HSPA14 FOSL2 SGCE TRPM1 XPO5 KRTAP4-7 ALG1 ROBO1 PPM1M FBXO31 DGAT2 LRCH3 SNED1 FADS3 SHMT1 A2M SEC14L4 SSX2 /// SSX3 LYK5 CORO1C LOC388886 RECK RAB27A LOC150223 MAP3K3 MRPS18A MRPL40 PLXNA1 EEF1D PCQAP DNAJC13 SLC8A1 USP48 ZKSCAN1 SLC25A13 C17ORF41 C10ORF56 EIF2AK4 GK2 PDLIM4 C6ORF145 HIC2 RAB9P1 LOC51161 DNASE2 PLEKHM1 GTSE1 MRPL53 ZFYVE19 ACAS2L STIP1 MRPS16 H3F3A /// LOC347376 MET PLOD1 PIR ISYNA1 POFUT1 C21ORF2 SPATA7 NDUFAB1 NXT1 HSPC111 FBXO18 FLJ25222 COL6A2 KIAA0133 C20ORF22 GRIA3 PREI3 MAN2B1 OSBPL10 TLN1 H2AFJ DNAJB1 PANK4 ALS2CR4 KIAA1429 VPS16 SFXN3 GATA2 RPUSD3 LFNG CSF2 BBC3 IGLC1 /// IGLC2 /// USP36 PLP1 FLJ35725 NDUFB1 SPUF TM7SF3 GPNMB EFTUD2 CNTFR POLR3C BSCL2 LOC253981 POLE NFATC2IP C20ORF149 PSEN2 GPR172A GK001 ZC3H11A GAGE8 /// GAGE4 /// METRN PFKFB3 PLEKHG3 TAPBP HRAS MAGEA3 TIMP2 PLVAP AP1S2 TNPO3 VAMP2 C14ORF147 SHARPIN C20ORF81 ZNF697 NUDT22 TAF1A FGF4 CXCR3 S100A1 WDR34 LAP3 LGALS3 /// GALIG TYRO3 IARS2 SILV CRTAP CGREF1 TTC11 FLJ21062 BMP7 FLJ35348 HARS YIPF1 INHBB XPA MIF CA14 YY1AP1 SEC23A POLD2 PVR BAMBI MLLT6 RASA4 NDST1 IFI27 TMEM4 GPATC3 COG4 COMMD7 INPP4B C6ORF152 SMA3 /// SMA5 PIGV FLJ20850 SPRR2E SLC25A25 CISH RPL23A /// LOC130773 GAL3ST1 RHOT2 SGCD SLC35D2 GARS FLJ10986 ASPA SDC3 GPR89 OCA2 DPH2 MARCH1 SH3YL1 CYBB TMEM8 CDK5RAP1 SRPX BAG3 KCTD2 FAM84A LOC91316 VPS33B MAGEA2 /// MAGEA2B PRPSAP1 BRD7 PDE3A MGAT1 GBA /// GBAP KCNN4 PTPRM SCYL1BP1 FLJ13912 MAP3K7IP1 RNF167 RNASE1 LOC93343 IGHD /// IGHG1 /// I P2RY10 NRL KHSRP RGS19IP1 CDK2 APOD C14ORF28 SSB1 LOC51035 MC2R SLC25A26 LOC339229 CRYBB1 COMMD1 MICAL3 PACSIN2 TYR MYH10 LCMT1 DKFZP564K1964 SKIV2L MIDN OSTALPHA BTN3A2 LIMK1 FLJ36031 PRAME AKT3 CCND1 D21S2056E ZFYVE16 WIPI49 COMMD5 AMMECR1 /// LOC28650 RFXANK THAP10 KRT10 PRAF2 DHX30 C9ORF74 TSC2 SF4 RNUT1 FXYD3 MGP RAB13 MRPL27 SENP2 PAK2 PQBP1 TIMM13 NBL1 ACOT7 GUP1 C1ORF93 NOC4L SEMA3B POLR2J2 GIPR MMP9 CYP19A1 GOLT1A IGSF3 SACM1L PLA1A C1QB NOSIP KLHL21 NUDCD3 FOXK2 MYOM1 BC002942 ARPC1B DDX49 SAP18 CTDSPL C1ORF24 PPT2 PIK3R2 CAMK2D MTX2 PITPNB IER3 BFAR C3ORF6 PAX3 PLAT GAGE1 /// GAGE2 /// KLF8 TBC1D7 HGFAC SLC1A4 EPPB9 SERPINF1 ITPKB HAPLN1 HYPK STATIP1 DVL2 BACE2 PHF20L1 PCOLCE RANGAP1 ECE1 POLRMT IGH@ GAPD UBQLN4 C16ORF30 PGF WDR48 JRK KIAA0367 C20ORF27 RRAGD MAGEA1 PAPPA SFRP1 SMPD1 C1ORF21 HKR2 MGC14327 JTV1 RCD-8 EIF2C1 COQ4 DUSP22 CRHR1 CTNNB1 MCM8 MT3 PRKCBP1 SCYL1 ICT1 SCNM1 BMP1 FAM57A CHRNA6 MCAM MRPL17 POLG2 ELAC2 SRGAP2 UBXD6 TEX10 DGCR2 FAM20B SMAP1 PMP22 TMED3 HLA-DQA1 PL6 C21ORF33 CITED1 EDNRB PSITPTE22 ADRA2A FAM14A RAE1 OPRD1 VEGFB EHD1 KIAA0963 RNMTL1 GRB14 ZNF672 DKFZP547K054 ATAD3B /// ATAD3A MYOD1 STK25 NOL10 COMMD4 KIAA0020 TPP1 CHMP6 CLN5 ACE IGFBP5 EIF4G3 CACNA1A BGLAP RABIF CHD1L TRIB3 AFF2 MAGED2 BIT1 C13ORF25 POMGNT1 MGC11257 MIA PANX1 DRD2 RANBP3 RTN2 ZNF42 NGFB RXRA TYRP1 DUSP4 MRPS6 GAS2L1 PHF23 DAB2 CSNK1G2 LOC128439 PFKFB2 QPCT TCEB2 PAF53 PRNPIP NR1H3 C9ORF41 PTD015

RAMASWAMY_GCM_MESOTHELIOMA na PLXDC2 MYO5B SORBS3 FIBP APOE MEST CPZ WT1 C1QR1 SOCS3 STAU2 AGRN WDR41 MMP2 NMU IRF7 MSRB2 NFATC1 GSK3B ARHGEF3 LAMA5 CNIH4 MEIS2 ZNF593 COL5A2 TGM1 CLIC3 YIPF5 AK3 GOLPH4 CKAP4 LAMA3 CAV1 PRSS11 CORO1C MT1E C6ORF109 P4HB ENPP1 SLC38A2 NRK UBE2E2 TSPAN3 CRABP2 C9ORF88 DELGEF MAP3K3 RNPC2 ADCYAP1R1 TNXB AP2M1 C19ORF10 RCN1 PTGER3 SCN5A PODN PTPN9 ZNHIT1 FLJ34922 MFAP5 CALM2 GAPDH PARP14 THBS2 RAB2 CXCL12 DNAJC13 LY6E SLC25A29 GPC6 RPS27 SULT1A3 /// SULT1A4 ADCY3 TMEM49 HCA112 CKLFSF3 VAPA EIF2AK4 SEMA3C MT2A PDLIM4 KLP1 RRBP1 CALB2 S100A10 PRKCI LTBR LOC92558 KLK10 SLCO3A1 MEOX1 MGC2749 LOXL2 KRT18 PRICKLE1 PTGIS PHPT1 MRPS16 POU3F2 TPBG VLDLR SECTM1 EFEMP1 MET PROCR PBX1 ISYNA1 TOB1 MAP3K11 ITGA5 FSCN1 IFI35 TRAM2 TGFB1I1 NDUFAB1 SERPINE1 ORF1-FL49 FBXO18 COL6A2 SAA1 ITGA3 TGFB3 SLC39A13 MRPL11 PSAP HECTD2 TAZ KRT8 TLN1 TPM2 RRAGC PARP9 TUBB6 PPP2R4 VAV2 LOC90557 /// DKFZP43 ALG3 REC8L1 VPS16 AP1M1 WISP1 NDN EPLIN HSD3B2 CD14 CHMP2A LEPR PDGFRL TRIM16 GFPT2 SPUF KIAA0859 CDA08 STRA13 GPNMB C8B IF TRPC1 ADH1B GALNT10 ZNF395 /// FBXO16 LOC91461 FAM26B MRPL19 PPIB LOC286044 MGAT2 PGM1 ARL6IP KLK8 LOC253981 FGFR1 FLJ39378 FOXJ2 DCI BOMB LOC126669 OKL38 MRPS15 ARL6IP4 C20ORF149 RFK CCR3 LAMB1 LOC144347 MAP1B GK001 ZNF621 ATF5 SPARC MAN1B1 LRP2 C4A /// C4B KLF3 KIAA1333 LHCGR RARRES2 DSG2 IMP-2 GPR81 SLC35C2 PLEKHG3 C1S G6PC3 PPP1R12A MAP3K15 ARPC5 TM4SF1 TAPBP PDZK11 MGC11242 HRAS FLJ21742 GOSR1 PPL TAX1BP3 BF PLVAP TENS1 HM13 GPR NEO1 CYB5-M ZAP70 BCHE SQSTM1 LHFPL2 RORB SPATS2 TNFRSF1A FKBP9 /// FKBP9L SHARPIN ITGB4 SLC12A8 PPIC HCFC1R1 MCFD2 TAF1A PDLIM5 SERPING1 RERG STAB1 ALDH1A3 BCAT1 LAP3 URB GPR37 LGALS1 TSPY1 OPCML C3 ANXA8 APOL1 C2ORF18 GATA6 APEG1 CRTAP CDCA7L STN2 TTC11 MT2A /// LOC441019 KRT5 FLJ35348 ELOVL1 TCFL5 SPESP1 CLIC5 NTAN1 MT1H CYBRD1 RARA ANXA2 ASS FNDC3B LRP11 SEC23A TRAP1 ZD52F10 PVR IFI27 TMEM4 LOC63929 PTPLB LOX PDLIM7 ACTN4 PIGT HSPB1 MFAP2 IFITM3 /// IFITM2 TGFBI YIF1 SPRR2E IGFBP6 DPYSL3 TEAD1 COL3A1 C16ORF35 SPP1 AGPAT3 STEAP3 C10ORF116 SMURF2 ADAM12 COL1A1 PRO1855 EDIL3 DHRS3 PRSS23 LOC440118 IL15 GABRB1 ABCA1 PTTG1IP RAB31 C14ORF173 TIMM23 HP PPHLN1 KIAA0776 HSPA1A BIRC5 FZD6 SLC11A2 COL11A1 DUSP1 EPB41L4B HIATL1 KIAA1295 TNFSF7 DECR2 CYBB OAS1 PKM2 EGLN2 FLJ13231 SEC22L1 LAMB2 MOSC1 BAG3 GAS6 NTNG1 PRDX5 SFRP2 FDXR SARM1 C2ORF12 AD-003 SLC4A2 DPYS H19 EDF1 PTRF ADH1C /// ADH1A /// DIRC2 PPP1R14B JMJD2A FER1L3 OBSL1 PDE3A FBN1 VIL2 C8ORF13 OXTR OGN GOLGA2 RAB34 SH3BGRL3 ZNF154 SEC61A1 C18ORF54 CEBPD CD68 LGALS3BP CCDC23 DNAJC16 MYL9 SLC35A4 EFCAB1 IQGAP1 RGS19IP1 CRIP1 MGC4677 SEC61G TMEM76 RUNX1T1 B4GALT4 ALOX15 DSP RLN1 PTPRF CNIH NDUFB10 LOC57149 GDPD5 COMMD1 TRA1 AKAP13 PAK4 POLR2L TMED7 HIC1 TMBIM4 THY1 /// LOC94105 DKFZP564K1964 SLC35A2 MRC2 OSTALPHA C20ORF52 BTN3A2 ROR1 LAMC1 FLJ36031 DKFZP564J0863 MEG3 /// LOC440199 AGTR1 C2ORF23 CENTA2 AMMECR1 /// LOC28650 C10ORF26 TBL1XR1 ACD PFDN1 CDK2AP1 MGC4368 CD47 DC12 SLC27A3 LOC90806 SLC24A6 PRAF2 BAZ2A HEBP1 CBX5 ARL1 HSD3B7 TBC1D1 PHGDH OTUD4 PLOD2 ZYX SC65 RNUT1 RPN1 MGP MRPL27 DAP LAMA4 FZD2 TJP1 NBL1 IL1R1 FLJ14668 SSSCA1 SEMA3B IGSF9 INMT UAP1 EHD2 RGS3 FAP SHB LTBP2 TGFB2 DCBLD1 KRT19 RERE PLK2 RAD51L1 CLIC4 LY9 AOX1 VPS26 POFUT2 PARP12 EPHB2 C1QB TMEM39A ZNF160 DHODH SCN7A IL13RA1 SCO1 KIBRA SELENBP1 KIAA1040 LOXL1 GLS MDM2 POLDIP2 SAP18 CTDSPL ANTXR1 MMP23A 182-FIP RUSC2 PIK3R2 NIFIE14 HPD SNTB2 IGFBP4 CA9 C3ORF6 CDH11 FLJ10916 TOB2 CST6 HSPC176 CSF1 SEMA5A MVP P4HA2 COBLL1 HYPK HAPLN1 DDEF1 CHPF TMEM14C MAST4 KRT7 ETEA COL12A1 LMAN1 C4BPA FLJ20254 PRG4 NOTCH2 PCOLCE M6PRBP1 RAP2B RHOC IGH@ GAPD CLDN1 PGF SLC44A2 FLRT2 LRRFIP1 RBMS3 FCGBP COL6A1 FCGR1A EXTL2 ROR2 TWSG1 KIAA1404 RPP25 SLC7A7 HAS1 SRP68 TIMP3 COL14A1 ADH1A /// ADH1B /// PX19 SVEP1 SULT1E1 DPCD EEF1A1 MTPN C9ORF3 CLTA UCHL1 C17ORF27 AQP1 TNNT1 ATP5I MATN1 SITPEC SOX18 NNMT AMELY CFH /// CFHL1 HSPC023 LEPRE1 TNFSF4 TRIT1 SERPINH1 COL18A1 BMP1 SERF2 FCGR3B MRPL17 TAGLN FMOD SCCPDH EIF3S12 LRP1 IGFBP2 IL10 COPE PHLDB2 MMRN2 CACNA1C FAM38B ID4 ARL2BP SLPI C21ORF59 BCAR1 SPRR2B PDXK ATXN7L3 DKFZP586J0619 PTMS TMBIM1 SRGAP2 PLEKHH2 TNFRSF12A RFNG LXN KIRREL MKL1 FAM20B ELL3 TMED3 SV2B SEC11L1 GPRC5A PL6 FSTL1 G1P3 C1ORF160 C21ORF33 OPTN VTN LOC255743 COLEC12 FUCA2 INHBA KLK11 SPOCK2 ICAM1 PQLC3 GLG1 WDSUB1 EHD1 NPC2 HT008 MAP3K7 KIAA0963 HGF ADAMTS3 FN1 ITGB1 SOCS5 CCR2 FLJ44635 LGALS8 DHCR7 LOC92689 CD200 GIT1 SLC39A8 TP53AP1 MICAL2 ASH2L UBPH PLA2G12A YIPF2 UQCR NAPRT1 STK25 C14ORF1 C2 NOL10 DNCH2 FAM62A COMMD4 UGCG BCL2 KIAA2002 CDC25B CRISPLD2 ZBTB9 MXRA5 AXIN1 IFITM1 PMM2 TYROBP DEFB4 KIAA0980 DBT PSMC4 CRIM1 SEC13L1 NXN C1R KPNA2 C5ORF15 ITGB5 MMP15 RNU60 KIAA0339 RRAS EIF4G3 RARRES1 TPM1 TMOD3 ANXA3 BHLHB2 FGF9 C1ORF122 BIT1 FSTL3 MAF FOXP1 PCBP2 SLC22A5 MSLN PIK3CD MGC11257 SKP2 SHC1 GAS1 ADAMTS5 SLC2A4RG LMNA TEAD4 NDUFS5 COL1A2 RBP1 DAB2 USP31 FBLIM1 PODXL IL18 GLB1L EIF4E2 C12ORF10 FMO1 TCEB2 MRPL24 SRPRB COMP FOSL2 ITPR3 ACTB

RAMASWAMY_GCM_OVARY na CCL11 MEST RREB1 CCL1 WT1 EXT1 REGL PHYH ATP8A2 DCHS1 RENT1 MMP2 ACADS CTF1 KRT23 CHKB /// CPT1B ATP6AP1 ADCYAP1 CUL7 C9ORF95 UBE2H IKBKE B4GALT1 GNPDA1 CLDN10 FBN2 CRIP2 GHRH ELA2 CRABP2 MFGE8 NRP2 STAP2 PTGER3 TMEM41B MUM1 ADRA1A ANK1 LY6E RBM35A FMR1 GABRA1 TACSTD1 SLC10A1 BDH MLF1 MYH2 RARG LIMK2 SLC27A4 IGLC2 CYP2C9 KLK10 YWHAZ LOXL2 KMO GDF5 PTGIS ZNF143 SLC6A9 PBX1 SLC17A2 FLT4 OR1E1 HMGA2 CXORF39 UBE2B SNAPC1 WIT-1 MYB NFIC CHIC1 KRT16 FOXF2 HPCA CDA ITGA1 BCL9 ESR1 TCEA2 CTLA4 HSD3B2 GJB1 KLF5 LISCH7 MMP7 ELK4 HNF4A PDGFRL MAGEA5 IFNA5 TRIM16 KCNJ4 NDRG1 TCFL1 IL17B IF NAGA CYHR1 CNTN1 SMOC2 RGR CSTF2 ERVK6 SFN DVL1 GMPR ITGA9 KLK8 HAS2 FGFR2 CDKL5 TP53I11 CST1 CHRNA3 ZNF165 LAMB1 MAGEA9 MAPK11 HTR3A SDC4 PNMA3 TUB HES1 CAMP SAG IL6R AKR1D1 SERPINA7 SHBG NEU4 COL15A1 APBB2 CRK PPIC TFAP2B MGC4251 WARS IFNA8 PPIL2 ADH4 MAGI1 GRM5 OPCML MUM1L1 LOC400581 ECM1 SCRIB AQP5 QSCN6 ABCA10 ANXA2 GRIN2B ENPP5 ACOX1 DMD SUPT4H1 ZD52F10 HRH1 IHH S100A13 PAX8 TCF1 IFITM3 /// IFITM2 IFNB1 MC4R COL3A1 FLJ37440 SPP1 DCPS CETN2 LAMP2 GNL1 SSTR3 ST5 WFDC2 PDZK1IP1 LOC89944 ARHGDIG HTR1B CD86 RNASET2 CYP1A2 HOXD4 COL11A1 PYGO2 FUT2 RAI14 PERP DJ222E13.1 TNNI3 NUP50 POR LAMB2 MOSC1 SLC25A1 GAS6 ORM1 KRTHA3B STAR MATN2 UROD EFNB3 H19 NID2 GALNS SPRR1B WNT5A RUNX2 CREB3 ZNF592 NUDT2 LGALS3BP MT1B SHFM1 NHLH1 MYCL1 PSD ERCC4 KRT12 TACSTD2 MAGEA12 PTPRF ATN1 LTB4DH FLJ13710 EBAG9 DBH MYL7 MCRS1 MUC6 NRCAM GPR125 CRABP1 SGCA GPR23 GP5 LOC158563 MDS1 PECAM1 EEA1 GNB3 MELK ASAHL NFRKB LAMA4 CXORF53 MGC10993 ATP6V1C1 PCSK6 MSX2 CLDN3 IGSF9 SELP MC5R FLJ14981 ORC1L KLK1 IL3RA GHRHR METTL1 ADRB3 LCN2 RBBP7 KIAA0157 MRPL14 MGC20235 IGLL1 GGCX MAGEA4 C1ORF91 RFC2 SIM2 NPPB RPUSD2 TSHB KCNA6 N4BP3 ETV4 UCN SIAHBP1 SCGB1A1 PNMT CAPS COL6A3 FEZ2 ARHGAP8 GCNT2 FLJ21439 CPN2 RDBP ZNF141 CSN2 SLC2A4 DDR2 CPXM2 TRIM29 CCR9 ZNF167 PCDHB17 IRS1 SERPINB4 XDH MGC27165 COL2A1 ABCD1 STARD8 C20ORF19 VIP CCNE1 COL18A1 RASSF7 AMPD1 IL10 ERCC8 AYP1P1 CASP9 FLJ45445 /// LOC3491 CRYGB GUCY2C PDXK ITIH4 UGT8 PPFIA1 FLT1 CHGA CST1 /// CST2 /// CS UCP1 ST6GALNAC2 C10ORF72 KIAA0143 TFAP2C NR2F2 TPST2 RBL1 KLK11 CREBL1 HSPA1L NFKBIL1 POU6F1 SLC26A2 HTR7 CHRNE HLXB9 NALP2 LOC541578 LRRC48 CDSN CA6 EFNA1 C8ORF72 SCNN1G SSTR2 PRDX4 FZD10 MAPK13 RRH SPINT2 IFITM1 AGPAT2 CYP2C19 ADORA2A PLCB4 KRTHB3 FAM3A MAPK8 PTK6 SPAR NR5A2 CNGA2 PTPN4 E2F5 MGST2 CETN1 TMEM16K DHFRP1 DRIM STK3 TG SLC35D1 COL1A2 RBP1 MVK CDH1 FCGR2A LU CRAT PRPH VTCN1 PYGM GNAT2 CENPE LEP C15ORF31 TSHR CBLN1 DRD3 IL17 PSMD5 ITK PTGS1 SLC18A2 PLAC4 HTR2B CHRM1 GOLGA1 GRIN2C S100G AGRP RNF113A CKAP4 PRSS11 ARHGEF5 AKAP1 KRTAP5-9 THAP11 QPRT ASMT AADAC TUBA2 /// LOC112714 NRK IRF5 CHRM5 ACRV1 KRTHA5 FAM80B RCN1 CXCL14 SLC14A2 EEF1D C4ORF9 GABRE EPHA1 ALPP CXORF6 C17ORF41 THRB CCKAR EIF2AK4 CYP2A6 IL1R2 PRKCI ABLIM1 LOC283501 YAF2 LHX2 KRT18 TPBG IGSF2 PLOD1 LZTR1 IL13RA2 EPHA5 ISYNA1 TOB1 C7 PTCH KIAA0153 OR3A1 AGTR2 EYA2 VDR UQCRB DKFZP434B1231 PCDH1 CBLB POP1 PRKCA CDH15 SLC34A1 SCGB2A1 SAA1 ITGA3 GAPVD1 SGSH TPM2 TFAP4 DDX11 SRM SP1 CSF2 ARPC1A FLJ90834 KIAA0195 IGLC1 /// IGLC2 /// NFKB2 ETV3 ATP12A TCN2 SLC17A4 KRT2B BCL2L14 FLJ21839 AFAP OAS2 DCC CHRNB2 LMAN2 USP13 IL13 S100A5 GPR172A PDHA1 CD8B1 CLCN5 HLF IGFBP1 TST BF TTN ABCC5 FAS FXN CRYM C9ORF115 CCR5 MTFR1 POLA2 GLI3 GPR SERPINA5 ST14 IL8RB AQP7 PLGLB1 CXCR3 NOTCH2NL CASP8 PBX2 S100A1 LGALS3 /// GALIG COLEC11 GCK C13ORF1 MYL3 CALCR TTC16 SILV CSNK2A2 SLC12A1 TAP1 GCG CXCL6 MDK CYP11A1 CLIC5 ACO1 ZNF35 SMPDL3B ASS EMP2 NGFR CRISP1 IFI27 SRY SCTR C6ORF152 EDN3 ZNF609 FBXO46 MT1F HSPG2 CDKN2A MAOA DAG1 RBPMS COL1A1 SGCD MSTP9 PRSS8 THRA EFNB1 CTNND1 CFTR CXCL1 HNRPD SH3YL1 COL4A5 KRTHB6 BRD1 PKP2 GH2 RBP4 DEFB1 FAM84A KCNMA1 MAPKAPK2 TEC ANXA4 TNFRSF25 SGCG SLC18A1 RBMY1A1 PDE3A RAB34 TAF1 FHL3 GPC3 BDNF RNASE1 FGF7 CEACAM4 NRP1 HOXA4 KIAA0194 FLJ20209 GSTM5 ARL3 PTPRU CTTN SLC5A3 HRK PHB2 CLDN4 ADORA3 OSTALPHA LAMC1 PEG3 PRAME SLC10A2 HLA-DQB2 GPX3 HMGB2 SRF SLC6A2 HYAL2 BST2 SLC30A3 SP100 GPR64 GJB2 OIP106 LOC400688 MYL5 MYF6 GALNT4 EVPL ACAA1 SEMA3B LTBP2 ARF4L NPR2 CHN2 POLR3D USP6NL FLJ25530 KLK7 RFX5 IVL MTSS1 FSHB DUSP26 C3F MPST CYP4B1 LRRFIP2 CALCB OPRM1 PLAUR IL1A PLAT EPHB4 FLJ90586 BMP5 EPPB9 CSTB PDIA5 CD4 IL3 RHOC PPP2R2B TPD52 SMAD6 RIN2 CHD8 CCL20 COL6A1 EXTL2 SPAG11 FHL2 SMPD1 SLC45A4 DUX4 /// LOC399839 / MPHOSPH1 ARSA HERC2 /// LOC283755 ACHE TNNT1 DDOST PDHA2 CDO1 SCARF1 PRCP CKM F25965 OR2H1 COL4A6 INPP1 TAP2 PIK3R3 FMOD DIO3 IGFBP2 SPP2 SQLE G3BP STAB2 SLPI SPRR2B ADM UBXD6 LYZ CP HLA-DQA1 CARD14 KRTHA1 PSITPTE22 INHBA PYCR1 PTHLH EDA MYL4 CSF2RB VGLL4 MAT1A PCCA HIST1H4K /// HIST1H4 ACVR1B GRB14 RPS6KA2 HMOX2 COL4A2 IL8RA SEMA3F GRM8 MASP1 CDH3 FOLR1 FZD9 HTR1E ADH6 MUC1 AXIN1 DLG4 PITX1 CST1 /// CST4 DHX34 MICB EGLN3 ACVRL1 LEFTY2 SMARCA1 PDK4 LOC143381 DHCR24 KCTD1 ATP11A IL20RA DKFZP434B0335 /// GA MSLN PKP1 EVI1 TGM2 PTHR2 IDUA SART3 FOXO1A SAH ADAR /// ADRBK2 /// GLI2 KRTHB5 CSN1S1

RAMASWAMY_GCM_PANCREAS na PRSS3 PKN2 PLXDC2 CPZ C1QR1 TCTE1L NTF3 GPX2 CTSD AGRN TGFBR1 MMP2 ACADS HYAL1 DAF ZNF593 KRT23 CLDN5 FAM3C SH3BGRL2 CLDN10 ENPP1 CCL19 ABHD2 THBD P2RY4 MFGE8 ADCYAP1R1 MYO1E GGTL3 GC MFAP5 THBS2 CXCL12 ME1 BTN3A1 CEACAM1 HBE1 MYH11 TACSTD1 BPHL IGHA1 /// IGHA2 /// CEL DNM2 S100A10 CYP2C9 LTBR DES KLK10 LOXL2 NOS2A RLBP1 CPD MUC13 SH3MD2 FLJ43339 MGC16037 ITGA5 PTPRR PNLIPRP2 TRAM2 TGFB1I1 SERPINE1 CYP2A13 ORF1-FL49 PPP1R1B SDC1 GPR19 KCNJ15 CCL14 /// CCL15 KRT8 ITGA1 CDH17 MVD CEACAM5 EPLIN CDK3 GJB1 IGFBP3 KLF5 LISCH7 MMP7 LOC92691 PLS1 NS5ATP13TP2 GALC CRYZ IF TRPC1 TRIMP1 TRIP10 LOC91461 SMOC2 VASP SFN RGS2 FGFR1 EGR2 KCNJ11 SEPT8 F5 CST1 LAMB1 SPARC FLII C4A /// C4B DSG2 C6ORF206 SDC4 PRKCZ TNF BIRC3 PCGF2 TM4SF1 VCL NEB RGS5 EDNRA IL6R DEFA5 TFPI BGN KDELR2 NCOR2 PHLDA2 COL15A1 NEO1 SLC26A3 LHFPL2 ELA2B /// ELA2A PPIC MYLK PDLIM5 SLC20A1 PIGC CHGB CDK10 SLC2A1 DDR1 DYRK1A MT2A /// LOC441019 CHRND SEPW1 TMEM51 AQP5 POSTN ASL ABCC3 AADACL2 LGP1 QSCN6 SULT1C1 MT1H ANXA2 IFNW1 DNAJB4 IGL@ /// IGLC1 /// I EPHA4 COX17 JAG1 S100A13 HSPB1 MFAP2 TCF1 TGFBI SLC39A14 CNN2 COL3A1 PSD3 SPP1 IGH@ /// IGHG1 /// I RER1 ST5 PCDHGB4 /// PCDHGA8 PDZK1IP1 E2F4 FAM3B HSPA1A SLC40A1 DKFZP761H039 CYP1A2 ZBTB7A PCDHGA12 COL11A1 TDO2 SERPINA3 SHRML FUT2 PRSS2 DAZ1 /// DAZ3 /// DA RAI14 PERP LAMB2 MOSC1 GAS6 MRPS21 GNB4 ARRDC1 LMO4 FER1L3 GOLGA2 CYP2C18 CEBPD ARHGEF12 LGALS3BP SLC18A3 TLE3 AHR GEM CRIP1 GNAT1 MGC4677 CYP3A5 PAM TACSTD2 ADAM9 PTPRF PTPRK PLCB3 SOD3 MUC6 ROR1 TMED1 LOC400986 CDS1 SGNE1 KIAA0792 KIAA0251 QDPR OTUD4 OGDH ABLIM2 GPC5 GABRP IL1R1 CMIP MC5R RGS3 TGFB2 KLK1 PNLIPRP1 EPS8L3 GHRHR SPARCL1 PSG6 ESRRA LCN2 CGB /// CGB5 /// CGB DLX2 LOC127262 LOXL1 CTSK TGFBR2 IGLL1 GGCX EPS8 ARHGEF16 NPAS2 APOA4 PML F12 MMP23A DSTN CASR IGFBP4 CA9 CDH11 TOB2 PPP1R1A CEACAM6 CSF1 MVP HLA-DRB1 PON3 KRT17 COL12A1 CDR2 COL6A3 AKR1C4 CTSS MDFI PLEC1 SLC12A4 TOP3B RBMS3 PRB1 /// PRB2 PSEN1 CSN2 GREM1 CPXM2 HPX IGL@ TRIM29 CAPN2 HIST2H4 RNF103 IRS1 XDH TFF3 NNMT CFH /// CFHL1 RORA LAMB3 UGT1A10 /// UGT1A8 / COX7A1 PRSS1 UGT2B15 TDE1 DEFA6 SERPINH1 COL18A1 LUM RASSF7 ARHGAP10 CYP27A1 GSN ACTN1 CST1 /// CST2 /// CS SCG2 HOXB2 VIL1 FSTL1 AGT PTGIR MAPK12 MTMR11 MMP11 CLOCK NR2F2 TPST2 ELA2B SERPINB3 PROS1 IGLL1 /// LOC91316 / GLG1 CPA3 CYP4A11 HLXB9 SPRR2D ME3 CD24 EFNA1 AK3L1 TRIM40 GLRB GRN SPINT2 AGPAT2 TCF7L2 DEFB4 MT1G BDKRB2 MMP15 RRAS TPM1 SPINK1 FBP1 JMJD2B RAP1GA1 ANXA3 CYP3A4 NQO1 CD164 SHC1 LMNA CD59 DRIM LOC149448 TG COL1A2 RBP1 FBLIM1 NT5M GJA4 CDH1 ATP1B1 PI3 FLAD1 FMNL1 CRAT SEPT11 ITPR3 MYO5B PELO BZRP MOXD1 TRPM1 MUC5AC CEACAM7 BENE CCL17 ACTA2 ENG MUC5B SNED1 COL16A1 HTR2B PLAU COL5A2 CTRB1 /// LOC440387 A2M ABL2 TNS SSX2 /// SSX3 LAMA3 CDC27 PRSS11 RNPEP PCSK1N LPGAT1 DIO2 TSPAN3 LAMC2 GATM INS CXCL14 PODN ANXA13 APOA1 /// LOC440837 ISLR TSC1 PCSK1 SLC8A1 ABCD3 SLC25A13 CYP11B1 ELN MRC1 PDLIM4 PGC MARK3 ABLIM1 BIK ZPBP LOC283501 DNASE2 PLEKHM1 MEOX1 PON2 KRT18 PRICKLE1 TPBG PURA TOB1 TGFBR3 C7 IFNGR2 C21ORF2 IFI35 KIAA0153 LTBP1 VDR HEYL PCDH1 GSTT1 CBLB NUMB COL6A2 PTS ITGA3 TCIRG1 KNS2 AKAP12 TPM2 SOX9 REG1A DNAJB1 P2RX7 PNLIP WISP1 CTRC ALDOB SFXN3 KIAA1117 CSF2 CYB5R3 CPB1 PRELP LMOD1 IGLC1 /// IGLC2 /// TCN2 TXNIP SERPINB5 PLK3 GPNMB NEUROD1 ADH1B AKR1C3 THBS4 NFATC2IP FBLN2 PPGB HTRA3 F3 CAPN1 RARRES2 GPR81 MST1R C1S ELF3 GBP3 CPA2 MGC11242 ACTN2 HRAS TST TAX1BP3 CST3 PLVAP HSD11B1 TFF2 RORB AEBP1 TNFRSF1A RAC1 ZXDA FGFR4 ITGB4 ERBB2 REG3A CXCR3 SERPING1 GGTLA1 SCP2 HAND2 TUBA1 PDE4C LGALS3 /// GALIG CSPG2 URB COLEC11 TJP3 MYL3 C3 AMY2B ALDH1A1 TTR GATA6 REG4 DIRAS1 SLC9A3R2 P2RY6 GCG ABP1 FA2H RAB25 MDK FLJ35348 GALE CDC42EP1 PDE1A EMP2 PDGFRB OLFM4 IFI27 SCTR REG1B EPHA2 FLJ13352 IGFBP6 SERPINA1 HSPG2 SMAD3 UGT2B4 CTNNA1 DAG1 RBPMS COL1A1 GOLPH2 C21ORF51 PLEKHC1 SLC35D2 SGCD MSTP9 PRSS23 DHRS3 RUNX1 PRSS8 COL10A1 EFNB1 FMO2 CTNND1 RAB31 C14ORF173 MMP1 BIRC5 SLC11A2 FABP2 CRP PTPRH FAM38A SRPX RBP4 INPP5A SFRP2 LOC91316 KIAA0683 TRIP4 ANXA4 TNFRSF25 S100P FLNA PGBD3 FBN1 MGAT1 PTPRM CTSE MAP3K7IP1 MYL9 RNASE1 ITGA6 SLCO2A1 SERPINB6 ABCC8 PLA2G1B RGS19IP1 PYGB ALDH2 APOD PCSK2 CAV2 RUNX1T1 SSB1 MC2R DERL3 LAD1 CDC42BPB CTTN AKAP13 HIC1 CLDN4 ELA3A /// ELA3B TSG101 THY1 /// LOC94105 TRIM66 SLC35A2 LAMC1 HSPA6 ELA3B MGLL CDK2AP1 LAMA2 LOC92799 KCNK1 BST2 HEXB GJB2 MGP FXYD3 C9ORF127 DAP CRYGD HABP2 EVPL NBL1 FUT3 /// FUT6 C1ORF93 INMT SHB LTBP2 FAP KIAA1462 KRT19 HOXC5 DCLRE1A IGKC /// IGKV1-5 S100A11 CAST TSPAN8 THEDC1 STS CTGF CYP1B1 KIAA0247 MYOM1 BC002942 ATXN1 SARS ITGA2 FUT6 IER3 PLAT GRK5 CST6 TCN1 CLPS VILL SERPINF1 BACE2 KRT7 PCOLCE RHOC ECE1 IGH@ PPY THBS1 FRZB PRKCD F10 CCL20 PTAFR ROR2 CTAGE5 STC1 FHL2 PAPPA TIMP3 OLFML2B ADH1A /// ADH1B /// SMTN EIF2C1 BCL2L13 CRHR1 C16ORF45 LGMN CYP24A1 CDW92 MATN1 JUP CORO2A PTPN12 INPP1 MCAM TAGLN FMOD CTRL DIO3 UGT2B7 LRP1 RIT2 LGALS4 DMWD PLN CYP3A7 /// CYP3A4 // ERBB3 MYOG CLEC10A TM4SF4 CPA1 TTC10 LYZ DNAJB12 RARS PMP22 HLA-DQA1 GPRC5A VTN CPE APOA2 HSD17B2 INHBA ADRA2A HRASLS3 PHYHIP HRC MAT1A PDGFRA KIAA0963 KRTHB1 FN1 ITGB1 ANXA5 KIAA0318 COL4A2 HMOX2 NOC4 CKMT1B CCL7 PRSS1 /// PRSS2 /// EDN2 CDH3 MYOD1 LRRC32 LEPROT GP2 CRISPLD2 MUC1 MXRA5 AOC3 CMKOR1 TFF1 MAP3K5 C1R RYR1 CST1 /// CST4 IGFBP5 ITGB5 FUT3 CIB1 MYH9 FSTL3 DKFZP434B0335 /// GA DDC GRP IGHM MSLN STX1A MIA DPEP1 TPSAB1 /// TPSB2 FOXO1A TERT PRSS7 ADAR /// ADRBK2 /// DAB2 HOXB1 SMPDL3A APOA1 QPCT COMP PTD015 LDLR

RAMASWAMY_GCM_PROSTATE na TACC2 ENOSF1 TRPV6 C5ORF18 ALDH9A1 ZCCHC2 ATP6AP1 ANAPC11 FBXW4 SPCS2 CHKA SH3BGRL2 BMPR1A ZNF652 EXT2 MGC13170 P4HB C9ORF88 STAP2 RNPC2 GCSH C19ORF10 MUM1 PRKAA1 FOLH1 ACADSB IBTK TDE2L RBM35A BTN3A1 TRUB1 C2ORF30 FASN MYH11 HEBP2 TMEM49 FLJ34443 MT2A DBI LIMK2 XBP1 LTBR DES PSMD7 DHRS6 LRBA MGC18216 SH3MD2 DKFZP434D0215 RBM6 TGFB1I1 SELE SNX22 TRIB1 SYNPO2 PAF1 SORBS1 TSPYL1 NEDD4L TAZ SBLF SNX9 MGC14376 LOC90557 /// DKFZP43 C14ORF154 TCEA2 NDN TOMM20 EPLIN RPS4Y1 CHMP2A SNRPN /// SNURF C7ORF27 TM4SF6 CSE1L DKFZP547K1113 LPPR2 COL21A1 FLJ21127 NDRG1 MTCBP-1 KIAA0859 TRPC1 CUTL2 CYHR1 GADD45G LOC286044 ASMTL CD9 GMPR RGS2 LITAF MGC17624 C20ORF194 EGR2 PDZRN3 EIF2B4 ARFIP2 THOC2 MTHFR RAMP1 KIAA1333 DSG2 SLC35C2 ELL2 MGST1 HES1 MRPL32 HPS5 EDNRA SELT ZDHHC14 KIAA1102 KIAA1272 ADARB1 PHLDA2 CX3CL1 MLPH DNAJC10 PPP1R7 KIAA1344 MYLK PDLIM5 ZFYVE21 ALDH1A3 DCXR XYLT2 ATP11B MUM1L1 RBL2 C2ORF18 IDH3B LOC115294 ARF4 FOXA1 LOC112869 FBXL3 IGF1R KCNAB1 HRB2 D2LIC BET1L GIT2 SLURP1 ENPP5 DMD HNMT C9ORF61 ZNF613 CALD1 IVD MRPL33 KIAA1280 HIST2H2BE EI24 SPOP TROVE2 C10ORF116 CKLFSF4 PEX11A LAMP2 SEC14L2 DDT DUSP3 CNKSR1 OAT GRINA FAM3B LOC153561 SC4MOL HSPA1A KIAA0776 GLUD1 YPEL1 SHRML ARID5A D4S234E FLJ21749 PERP MLLT4 ENTPD6 AR ZCCHC6 GAS6 MRPS21 IRS2 SEPT10 EDF1 ISG20L1 SLC39A7 PTPN3 MYCNOS ATF3 FER1L3 GOLGA2 ZNF33A KRTHA4 CYP4F3 /// CYP4F2 ARHGEF12 CEBPD EGR1 NUDT2 LOC376745 C21ORF25 SHFM1 SORBS2 PTGS2 HK1 C6ORF125 DUSP16 B4GALT4 TACSTD2 ADAM9 BMS1L DSP PTPRF NDUFB10 LTB4DH LOC57149 TRA1 PKD1-LIKE FLJ13710 PAK4 SOD3 SLC37A1 P2RY5 AMOTL1 MCP CGNL1 SPON2 DHX32 ZNF511 SEC24D TES SPCS1 KIAA0251 ARL1 FLJ21963 GABRP IL1R1 PNPLA2 DST ALDH7A1 SGEF UAP1 TMEFF2 KIAA0268 /// UNQ6077 LRP2BP AKAP6 COQ6 POFUT2 NFIB C2ORF31 PHOX2B C1ORF22 KIAA1160 /// RAB43 SELENBP1 PPARD RASL12 PDE9A DKFZP434K1421 YIPF6 ADSSL1 DSTN PRKAR2A MALT1 SNTB2 ITGA8 SIM2 PLAC9 RABAC1 TOB2 DHRS7 RSC1A1 PPP3CA TSC22D1 VPS24 F11R ARMCX1 PARVA GDF15 PPAPDC1B SEPP1 LRRFIP1 RBMS3 DNAJB14 SLC17A5 ARFIP1 RNF185 CAMTA1 SOAT1 GSTM2 /// GSTM1 /// C9ORF3 BIRC2 PPP1R16A FLJ22709 MTAC2D1 RNF103 CD81 IRS1 AGL PSCA YTHDF3 DEFA6 ZAK PLA2G2A PEX7 DSC2 SLC31A1 SCCPDH ID2 MGC23280 PHLDB2 FLJ45445 /// LOC3491 JUNB LOC90826 UTRN STK39 TRAF3IP1 FSTL1 STRN3 CCDC2 CAMK1 C10ORF72 RANBP9 COLEC12 GMEB1 KIAA0143 OSBPL9 CSRP1 TFAP2C PVRL2 CROT KLK11 FLJ20487 SLC39A10 LOC90355 GAGEC1 NCOA3 SUMF1 SMO LOC92689 SORD ACY1L2 WFDC1 UQCR EFNA1 GENX-3414 MAPK13 ARMCX3 SYT7 ISL1 CNN3 DKFZP761A132 GGTL4 /// GGT1 /// G CELSR1 SPINT2 PMM2 HSD17B4 DBT NXN ACOT2 NOL3 TPM1 LOC400451 CPSF3L HSA272196 ANXA3 LOC403313 BHLHB2 MGC34132 MAF FOXP1 ACPP ANKRD25 TM9SF1 DKFZP564M082 DKFZP566O084 SLC39A6 STK3 SART1 FLJ22794 FBLIM1 ANKH CDH1 NANS BCL2L2 LU ADAMTS1 RTF1 CRAT MRPL24 SLC25A4 PELO ZC3H12A SPFH2 ALOX15B YSG2 NUBP1 SOCS3 ACTA2 SLC39A1 ZFP36 HOXB13 FLNB LPP TNS MR-1 CKLFSF6 ARHGEF5 TLK1 AKAP1 TSEN34 RG9MTD2 LPGAT1 SLC26A6 CYR61 NRK TLOC1 ARHGEF15 PTK9 PRSS15 C4ORF10 PODN MRPL40 MARCH6 RAB2 NR4A1 ADRB2 MRPS9 GPR124 LOC144871 KLK2 ID2B ZKSCAN1 PIM3 SIX1 SEMA3C SERP1 PRKCI PENK LOC51161 BIK PRKD1 MRPS16 TOB1 TGFBR3 NPDC1 KIAA1458 LTBP1 UQCRB GSTT1 PTS HRPT2 TGFB3 IQWD1 SGSH KIAA0146 H2AFJ TPM2 LOC171220 /// DSTN SLC35A1 MGC17299 VPS16 UBE4A PGM3 GATA2 MSL3L1 PLA2G7 CYB5R3 LMOD1 PIGH MOV10 USP36 DKFZP564G2022 TRGC2 NPAL3 IDH1 PER2 CD2AP LOC126669 FAM18B C20ORF149 PSEN2 GK001 LARS2 HKR1 F3 KLF3 THUMPD1 ACSL1 SPAG16 MB PPP1R12A MGC11242 LOC399884 GOSR1 PPL E2IG5 NEFH C22ORF13 ADHFE1 CREBL2 C14ORF147 ZNF697 SCP2 SCUBE2 FLJ22269 COPB2 CAMKK2 FLJ21062 CANT1 RAB25 YIPF1 IL4 XPA EMP2 SEC23A TMEM4 ZNF444 HMGCS2 PTPLB BZW1 /// LOC151579 CTGLF1 PIGV JMJD3 FLJ20850 HOXA11 MRPS23 HSPG2 MAOA CISH AZGP1 G3BP2 POU1F1 GOLPH2 PLEKHC1 FLJ39370 PRSS23 DHRS3 ATP2C1 NPY MARVELD3 TIMM23 BRP44 GPR89 SEC22L3 SLC35F5 SLIT2 DUSP1 GPD1L C14ORF4 SH3YL1 PPA2 BAG3 INPP5A KCTD2 ANPEP KCNMA1 HOOK2 ACTG2 PLDN PTRF TXNRD2 C6ORF51 EGR3 MGC16028 CRY2 PGBD3 PRPSAP1 WDR42A MORC3 PCP4 GABARAPL2 FAM11A MAP3K7IP1 TMEM23 DNAJC16 GOLPH3 MYL9 ORMDL2 C1ORF116 SERPINB6 PYGB MPI AIM1 MGC9913 TNFSF10 FLJ20273 GRK1 TMEM76 KCNMB1 COMMD1 CTTN HPN TMED7 CLDN4 DKFZP564K1964 EPHX2 KIAA0152 NBR1 NFE2L1 WIPI49 TBL1XR1 GNG10 /// LOC552891 HEXB FXYD3 MGP RAB13 MRPL27 ANKRD15 NBL1 RBM35B SLC14A1 RBM7 INMT HOXC5 DKFZP313A2432 TMED10 RERE GFPT1 TTC3 P2RX4 SLC27A2 DKFZP761B107 UBE4B BOK L3MBTL2 CYP1B1 C3F C1ORF66 KLK3 CTDSPL SPIN2 SEC24A COCH C1ORF24 PIK3R2 MTX2 FHOD3 ANTXR2 SULT2B1 RAB4A PRSS16 COL7A1 C8ORF35 DPM3 TARP /// TRGV9 ADRB1 REXO2 VILL COBLL1 HYPK LOC146517 TMEM14C GZMH ALDH4A1 C6ORF210 LMAN1 ALG2 TM7SF2 SVIL C6ORF111 ECE1 MGC5987 KCTD14 F10 PER1 KIAA0367 FOS GOLGA4 RBM25 SEL1L ROR2 FHL2 GLO1 SFRP1 PRIM2A C1ORF21 HERPUD1 AMD1 SMTN CCK SC5DL DPCD COQ4 CIRBP CTNNB1 LGMN TSPAN13 TUSC3 CORO2A LOC134145 PRKCBP1 C3ORF10 EXOC8 TAGLN C9ORF10 ERBB3 MAL2 RWDD2 TMBIM1 KIAA0556 DGCR2 MTCH1 NEK3 CPE AUH CARD14 KRT15 KIAA0310 DMN MLH3 MYBPC1 MGC70870 ARFGAP3 CKB STEAP4 FURIN IDI1 PRDX6 BTG2 TMEM50B CPT2 DPP4 TP53AP1 HGD PLA2G12A SS18L2 CYB561 CDH3 STK25 FLJ13576 PCYOX1 CRISPLD2 NDUFC1 AOC3 CYP51A1 SUCLG2 MAP3K5 CXADR STK38L WWP1 MYO5C HDLBP GULP1 FAAH SMARCA1 IL16 DHCR24 TRIM23 ECHDC2 MAGED2 MYO7A BIT1 SLC22A5 PKP1 MGC11257 CNN1 C16ORF34 KIAA0934 GUCY1A3 DNAH8 SAH PPP1R12B MSMB SLC2A12 BAG2 LDLR

RAMASWAMY_GCM_RENAL na AVPR1B AGER KIR2DL3 RREB1 SLC9A3 CCL1 CD40LG REGL RNASE4 C5ORF18 ATP8A2 ACADS HYAL1 CA12 CRYAB CTF1 HSU79275 ADCYAP1 C9 PIGB SPN LOC402055 PDE6A B4GALT1 ACTC GNPDA1 MAGEA11 ELA2 MYO1E PPP2R5A PTGER3 INSR TMEM41B REN GSTA1 /// GSTA2 /// MLL3 FXR2 OCLN ACAA2 ANK1 GABRA1 SLC10A1 BDH MLF1 LIFR RARG MYH2 PDE1C CYP2C9 IGLC2 SLC6A3 SNCG NOS2A LOXL2 RLBP1 GDF5 KMO FXYD2 PTGIS SLC6A9 PPARG PBX1 NCAM1 SLC17A2 FLT4 ELF5 OR1E1 HMGA2 ORF1-FL49 STAT4 UBE2B MYB WIT-1 SDC1 NFIC CYB5 CHIC1 KCNJ15 CX3CR1 KRT16 HDHD1A FOXF2 TTPA SLC12A3 TCEA2 PVALB CTLA4 HSD3B2 IGFBP3 PGM5 GNG11 CAPZA2 ELK4 HNF4A MAGEA5 SUOX IFNA5 PLS1 BHMT PRL KCNJ4 TNFAIP1 SLC17A1 CRYZ STOM NAGA RGR CSTF3 PGM1 ERVK6 FGFR2 DCI ZNF174 HLA-DQB1 MAGEA9 CA1 MAPK11 ABCD2 DSG2 SDC4 ADIPOQ MESDC2 PCGF2 HES1 RGS5 C16ORF5 MUT GJA1 SERPINA7 TFPI CBS LOC166994 /// LOC340 SHBG PCK1 PSG7 IFNA16 TNNT2 IFNA5 /// IFNA6 /// CLUL1 CHD5 HLA-G SP2 TBX2 VCAM1 NEDD9 BNIP1 IFNA8 MAGI1 ADH4 GRM5 BBOX1 IDH3B CDK10 MRPL4 COX10 ONECUT1 SULT1C1 D2LIC TACR3 GRIK1 CCL8 DMD ITGB6 CLU C9ORF61 HRH1 JAG1 SPTB PAX8 ABAT MC4R CDH6 FOXC1 GUCY1A2 C10ORF116 GNL1 CFDP1 SSTR3 SPRR2C DRD1 SOSTDC1 MMP16 PDZK1IP1 LOC389203 CD86 GJA5 TNFRSF11B ARSB CYP1A2 HOXD4 GZMA FUT2 OAS1 RIT1 AKR1C1 SLC25A1 NQO2 KRTHA3B PKP4 UROD EIF4A2 H19 GTPBP1 SPRR1B LLGL1 WNT5A ATP7A WDR79 CYP2C18 TFCP2 MT1B FGB NHLH1 HARSL EGF MTERF KCNJ5 CYP3A5 ERCC4 KRT12 TACSTD2 MAGEA12 LTB4DH GYS1 FLJ20152 SOD3 RAPGEF5 ELL NRCAM MYH3 CGNL1 GPR23 GP5 HTR2A MDS1 KIAA0251 MEP1B GAGE1 GNB3 TGFA NFRKB ADORA1 MYO5A PPARA PCSK6 TTC1 SELP MC5R IFNA2 RBP5 NTRK2 MCC KLK1 GHRHR KIAA0157 GGCX MITF SLC25A16 SIM2 TNFAIP6 CMYA5 NPPB TSHB KCNA6 S100A9 ZNF8 RGN FEZ2 SDC2 CHRNB3 KNG1 SEPP1 RDBP PAX5 ZNF141 DLG2 SLC2A4 DDR2 LGALS2 CCR9 ZNF167 CLTA PCDHB17 SERPINB4 MMP8 RXRG CHRNA4 MYH7 MGC27165 ATP5I UGT1A10 /// UGT1A8 / VIP COX7A1 BCAT2 HNF4G ABCB1 AMPD1 PHLDB2 AYP1P1 ACADL PCYT1A GUCY2C ITIH4 DGCR5 FLT1 ITGA2B HOXB2 UCP1 NEU1 MTMR11 ENTPD1 DSG3 HSPA1L CREBL1 PROS1 NFKBIL1 NPY1R CYP4A11 CHRNE TEF HLXB9 ZNF132 CRADD CCR2 GZMB WNT2 FANCC CD24 AMHR2 MAPK13 ELAVL2 GRIN2A CYP2C19 DBT HAL ASIP KRTHB3 FABP3 CYP2D6 SPINK1 MAPK8 PTK6 SPAR ILVBL SLC11A1 GLP1R CDH5 PXN GSTA2 /// GSTA3 /// CYP3A4 CFHL2 PAH CETN1 TMEM16K ADAMTS5 CD59 SDHA GLB1L MVK GJA4 ATP1B1 NANS FCGR2A LU DHRS2 SLC25A4 ABCG1 PYGM GNAT2 CENPE CEACAM7 C15ORF31 TSHR NAT2 DRD3 IL17 PSMD5 MMP19 ITK DLD SHMT1 GOLGA1 PITX2 GPX4 RNF113A ZNF187 LRRC16 LOC339929 ATM /// NPAT ARHGEF5 KRTAP5-9 DNASE1L1 MYH8 ASMT AADAC ALDH5A1 SLC6A4 CHRM5 PIB5PA ACRV1 ALPL CR1 GATM ANK3 ITGA7 SLC14A2 C4ORF9 ADRB2 TSC1 KRT13 NOTCH4 SLC1A2 EPHA1 CXORF6 FAHD1 IQCB1 EIF2AK4 CCKAR THRB CYP2A6 TCEAL1 MUC15 FLJ23186 IFNA21 YAF2 CTH F8 YES1 PGK1 MET HOXC6 EPHA5 IFI35 OR3A1 GABRQ UQCRB ENPEP PCDH1 POP1 PRKCA CDH15 ITGA3 ACTL8 IDS TFAP4 DDX11 DAZL POU3F4 SRM SPAM1 SP1 BFSP1 CD151 CSF2 CYB5R3 IGLC1 /// IGLC2 /// NFKB2 ETV3 GFER PPP3CB SLC17A4 KRT2B HMGCL AFAP DSCR1L1 DCC B1 S100A5 PDHA1 CD8B1 AHSG CHIT1 HLF ID3 MGC11242 ACY1 MAGEA3 HSHUR7SEQ ABCC5 FAS FXN CRYM SYN2 CCR5 PLVAP TNFRSF14 MTFR1 DEFB127 IL8RB FGFR4 AQP7 GOT2 PLGLB1 CXCR3 GRK6 EHHADH CASP8 PBX2 RERG TNK1 CHRNA5 GCK PRNP LIPC C13ORF1 UBC MYL3 CALCR NIFUN ALDH1A1 APOL1 SLC31A2 CSNK2A2 AK1 SLC12A1 G6PC CXCL6 HUMBINDC ALK ESM1 NR2C1 ACO1 RPS6KA3 HMOX1 HSPA9B OR1D2 CART1 NGFR KIAA0040 ZNF10 FAH SCTR C6ORF152 NR5A1 FBXO46 MT1F MAOA HSPG2 RNF5 CD80 OS9 PITPNA TAT BLVRA LIF FABP6 MSTP9 ASPA THRA CTNND1 ERV3 CDC25C AMT CFTR SPRR2A CSN3 SLC6A12 GPD1L HNRPD COL4A5 KRTHB6 CRP NRF1 HRSP12 CCKBR APXL NR4A3 GH2 RBP4 PTHR1 DEFB1 KCNMA1 MAPKAPK2 TNFRSF25 ADORA2B MAGEA2 /// MAGEA2B SLC18A1 LYST KLKB1 EML1 HTR2C PRKG1 PTPRM GPA33 SOX3 TAF1 BDNF FGF7 AVPR1A SLCO2A1 CEACAM4 PRB2 PPP2R3A CAV2 HOXA4 FLJ20209 RUNX1T1 TM4SF12 GSTM5 SLC5A2 PTPRU NR3C2 EPOR PHB2 ADORA3 PNPLA4 SLC15A1 EPHX2 NOTCH3 BTN2A2 CCND1 SLC10A2 ACAT1 HLA-DQB2 PLCL1 HMGB2 FSHR LOC92799 SLC6A2 HYAL2 SLC30A3 GPR64 AKR1B1 /// EIF3S9 WWOX MUC8 RAB13 MYL5 MYF6 HOXC5 SLC17A3 PDHB CYP19A1 USP6NL PYY PLA1A IVL MTSS1 FSHB DHODH MGC72104 CYP2J2 MDM2 RDS C6 CALCB PPT2 POU2F2 TRBV21-1 OPRM1 GAGE1 /// GAGE2 /// KIAA0664 FLJ90586 BMP5 HGFAC EPPB9 SERPINF1 KRT7 FCER1A ALDH4A1 PDIA5 ACP2 MGAT5 CD4 IL3 PRRT1 FEV PPP2R2B SMAD6 RIN2 IFNA4 PMVK HTN3 CHD8 CCL20 TAF15 KIAA1328 FDX1 CTAGE5 IGF2 KCNA4 SPAG11 GHR FGFR3 SMPD1 CLCN6 COL14A1 DUX4 /// LOC399839 / ZNF177 MPHOSPH1 TPMT CXCL5 RGS16 TTYH2 SCARF1 DPF2 GCM1 CKM OR2H1 SLC2A2 AIG1 MCAM UGT2B7 HIGD1B ITGAD MMRN2 PIGF CYP3A7 /// CYP3A4 // MTF1 CYP4F12 ADM NR1H4 TBR1 KCNQ1 DGCR2 HLA-DQA1 SV2B PL6 ARAF ZNF37A SLC7A2 HUMPPA AUH GOT1 OTC APP KRTHA1 HSD17B2 GNRH1 INHBA LOC81691 EDA HRASLS3 GRB10 MYL4 ST13 GPR1 HCG8 ACVR1B KIAA0274 GML IL8RA SEMA3F GABPA MASP1 HRG FOLR1 LRRC32 SLC1A1 BCL2 MUC1 ADH6 AXIN1 UMOD LDHC DHX34 PHF16 CNNM4 EGLN3 ACVRL1 NPY6R WDR23 SMARCA1 PDK4 TJP2 HTR1A DDC FLJ37034 TNNI2 SLC22A5 GSTA2 EVI1 PTHR2 MAOB FOXO1A ART3 DAB2 GLI2 CSN1S1 SMPDL3A THRA /// NR1D1 BAD FMO1 KEL

RAMASWAMY_GCM_UTERUS_ADENO na OVGP1 GSTP1 CKS1B EPHX1 IRF3 C1QR1 CPZ PPP2CB C8G PRKACG CDKL1 KIAA0174 COG2 FKBP2 DCHS1 PUM1 ADD2 ALDH9A1 PBP ZNF593 TAF10 LLGL2 ATP6AP1 SPCS2 B4GALT1 CLDN10 EXT2 P4HB ENPP1 CRIP2 ADCYAP1R1 GCSH TNXB CD63 GGTL3 PTGER3 ZNF2 MUM1 TXLNA CXCL12 TDE2L ANK1 MYH11 TACSTD1 IFNAR1 MLF1 BPHL IGHA1 /// IGHA2 /// RARG DUSP7 LIMK2 XBP1 PEA15 LTBR IGLC2 KLK10 SMR3A TNPO1 CPD VLDLR ENO1 CASP7 TGFB1I1 RAD23A SSBP1 NDUFB7 CAMLG WIT-1 CCL14 /// CCL15 IGF1 HIF1A PRKAR1B SMARCA4 KRT8 ITGA1 P4HA1 LOC90557 /// DKFZP43 MVD ALG3 BCL9 ESR1 SFRS7 NDN CLPP LRPAP1 CCT3 PGAM1 KIAA0240 RPL13A SCAMP1 IGFBP3 CA8 KLF5 LISCH7 GNG11 MMP7 SUOX CUL3 CSE1L COL21A1 BCR AGA TNFAIP1 COX7A2 SRP9 TRPC1 IF TRIP10 PMPCA PPIB ERVK6 SFN GMPR MRPS31 FGFR1 BTN3A3 /// BTN3A2 // FGFR2 DCI C1ORF26 TUBA3 ARFIP2 ATP5J HOXB5 NARS RAMP1 SNRP70 ITPK1 PRKCZ GCHFR BIRC3 TESK1 HES1 NDUFS8 EDNRA GJA1 UCP2 FDFT1 COL15A1 EFNA5 PARG CX3CL1 B7 YWHAE GANAB PPP1R7 PDLIM5 MYLK PCSK7 INHA SALL1 PSME2 SUZ12 LGALS1 PIGC VRK1 SF3B3 CDKN1C CDK10 SLC2A1 ATP2A2 ARF4 SCRIB SCAP PGAM2 SEPW1 AQP5 LOC126208 MTAP ASL QSCN6 CRISP3 FLOT2 MT1H ANXA2 RHD PCOLN3 TRAP1 PMM1 FAM54B HRH1 IHH S100A13 COX6B1 SMAD1 HSPB1 MFAP2 PAX8 IFITM3 /// IFITM2 HMGB1 SLC39A14 TLE1 NT5E PSMC5 RPS6KA1 IMMT CETN2 PTK7 CFDP1 ST5 SMAD5 WFDC2 PSMA4 UMPS RNASET2 SLC6A14 LOC153561 HSPA1A BRD3 NAP1L4 PCDHGA12 SERPINA3 TRADD RIT1 ANXA11 AR LAMB2 GAS6 MRPS21 IL11RA PPP2R5E GRSF1 H19 SLC39A7 BAG1 LMO4 H3F3A GOLGA2 ZNF33A XRCC6 ILK PIK3R1 NUDT2 LGALS3BP SLC18A3 EFCAB1 FUT1 SHFM1 CRIP1 JUND PAM BRF1 TACSTD2 ADAM9 PTPRF LTB4DH GYS1 DYRK4 FY PTPRK TRA1 HAPLN3 POLR2L UBXD3 SOD3 C20ORF52 DNCL1 SGCA EPRS UBL4 CDS1 KIAA0792 POLR2A SDBCAG84 OTUD4 PLOD2 SC65 RPN1 KIAA0090 IL1R1 ALDH7A1 AP2S1 RGS3 IFNA2 CASP6 CELSR2 HDAC2 HAX1 ESRRA RPL9 PHKG2 COX5B HNRPK TNFAIP2 C19ORF25 RPL38 CTSK IFI44L MITF NPC1 PML NF2 F12 LRRC17 DSTN IGFBP4 TOB2 EIF4E CMYA5 GP1BA RPUSD2 RPL34 ETV4 TSC22D1 HIST1H4I CAPS SURF1 INPPL1 FADD VDAC2 FEZ2 SEC24C TNFRSF4 GDF15 KNG1 SEPP1 RDBP SLC12A4 GBE1 XIST SEC14L1 PMCH CSN2 MGC10433 DDR2 PRLR BLZF1 C11ORF8 EIF2B5 CLTA CD81 IRS1 ATP5I TFF3 COL2A1 POU5F1 RRM1 COX7A1 DEFA6 CPT1B SERPINH1 GALT COL18A1 SUPT5H AQP3 PEX7 RASSF7 LUM ATP6V0C PSMB5 POLR2H PDXK GSN MYL2 PAFAH1B3 SNW1 ITGA2B HOXB2 CSNK1E NFYC CCDC2 ST6GALNAC2 MNAT1 PTGIR CEBPZ MMP11 RPL7L1 CLOCK CSRP1 NR2F2 NEDD8 CYFIP1 DAD1 HTR7 CYP4A11 RPS24 C2F SORD LAMP1 CD24 PRKACA EFNA1 NDP AK3L1 COASY PRDX4 TRIM40 CNN3 GLRB UTP14C STX5A SPINT2 IFITM1 AGPAT2 SLC30A1 DBT NXN MT1G BDKRB2 PROL5 MMP15 PTOV1 UROS FAM3A RAP1GA1 RPS28 E2F5 NQO1 FMO3 DDX6 SHC1 RNF4 GTF2E2 CD59 PCYT2 STK3 SLC39A6 MGC10471 COL1A2 RBP1 CYP11B1 /// CYP11B2 PPP2R5D FBLIM1 PFDN5 CDH1 NANS PI3 FLAD1 CRAT MAP2K5 FOSL2 ABCG1 VTCN1 BRD2 ACTB HEXA MOXD1 POLD3 PHKB RPN2 SEPHS2 ACTA2 PTGS1 HSPA5 ENG BET1 PLAU RBM4 A2M FDPS CKAP4 LAMA3 CDC27 ARHGEF5 CAPNS1 RNPEP AKAP1 GATA1 ITPKA SMCX LPGAT1 DIO2 CYR61 TUBA2 /// LOC112714 TSPAN3 AP2M1 LAMC2 PTK9 RCN1 ANK3 SLN C4ORF10 PTPN9 MARCH6 ZNF84 FKBP4 APOA1 /// LOC440837 ISLR CREBL1 /// TNXB EEF1D C4ORF9 RRAGA TSC1 ABCD3 SULT1A3 /// SULT1A4 CCKAR SEMA3C EHMT2 MARK3 GABRA3 SEC6L1 ABLIM1 MLC1SA LOC283501 RW1 PON2 H3F3A /// LOC440926 YAF2 KRT18 HSPH1 STIP1 TPBG LOC161527 H3F3A /// LOC347376 PLOD1 RPL10A RPL8 TOB1 TGFBR3 MSX1 KIAA0179 TRIP12 LTBP1 EYA2 HADHSC GSTT1 FBXO18 CBLB NUMB COL6A2 SCGB2A1 PCDHGB4 SAA1 KPNB1 TCIRG1 PDIA6 PSAP CASC3 KNS2 G1P2 TMSB10 TPM2 DNAJB1 PPP2R4 EPHB3 NDUFV3 CD151 CSF2 CYB5R3 GMFB LMOD1 IER2 LTF FLJ35725 SDF2 PPP3CB HOXB7 SPUF PAX2 SSR4 SH3BP2 ECHS1 GTF2F1 HMGCL AFAP CENPB GCN1L1 SCNN1A CHRNB2 BSCL2 CDKN1A LMAN2 RELA RPS27 /// ZFPL /// R CAPN1 KEAP1 ITGB8 RARRES2 PLEKHG3 ELF3 ID3 ZNF261 PFKP HRAS TST GOSR1 YY1 TCTEL1 TAX1BP3 HFE ATP6V0D1 NOLC1 ZNF638 RHAG MTFR1 SERPINA5 RPL39 RORB RAC1 BNIP3 ERBB2 CXCR3 RPS3A SERPING1 HAND2 CASP8 COX7C WDR34 LGALS3 /// GALIG TJP3 TYRO3 WIRE UBC GSS MYL3 COPB2 C3 ARMET SLC9A3R2 SEC61B THRAP4 PSMD1 RAB25 FLJ35348 MDK HARS TFPI2 YIPF1 POLR2E GALE RARA FAM50A MIF PDGFRB CCT6A GTF3C2 POLD2 FH BAMBI IFI27 TMEM4 BCKDHA EPHA2 FLJ13352 SND1 NR5A1 HOXA11 MT1F HSPG2 CISH UBE2D2 RPL23A /// LOC130773 CTNNA1 DAG1 RBPMS COL1A1 CALR PLEKHC1 DDB1 FABP6 ASPA DHRS3 CDC37 LOC440118 PRSS8 CUL2 FMO2 LMO1 ATP6V0B RANBP2 HIST1H2AE CCBL1 SLC11A2 CXCL1 FUCA1 PSME4 COL4A5 PKM2 SCNN1B RPA3 ACLY INPP5A KCNMA1 PRKG2 PKD1 DCTN2 WDR42A VIL2 GBA /// GBAP MGAT1 TMOD1 SSR2 HYOU1 DNAJC16 MYL9 BDNF ITGA6 SERPINB6 PDLIM1 RGL2 KHSRP RPL37A MPI ACTA1 ZNF278 RUNX1T1 LOC51035 ALDH3B2 MC2R KCNMB1 ARL3 LAD1 CTTN AKAP13 DUSP8 MYH10 TSG101 MTIF2 PFN2 SLC35A2 FKBP1A LIMK1 KIAA0232 C4ORF8 CCND1 PRAME GSK3A NBR1 NFE2L1 VRK2 RFXANK CDK2AP1 GLB1 FSHR SRF KCNK1 GNG10 /// LOC552891 BST2 MGP RAB13 ATP2A1 MAP3K4 ANKRD15 DAP TIMM13 USP5 ALCAM ARF4L HOXC5 HOXD10 CHD4 PIAS3 TMED10 S100A11 TRIP11 TTC3 P2RX4 KIF1A SCGB2A2 C3F BC002942 ATXN1 MDM2 MPST PPT2 IER3 METAP1 FLJ90586 SEMA5A AHCY ITPKB ACP2 EBNA1BP2 RCN2 RHOC POLRMT KCTD14 RAD23B SMAD6 PMVK GOLGA4 COL6A1 EXTL2 CTAGE5 CLTC SFRP1 MEIS4 JTV1 SMTN RCD-8 ARSA PSMB3 MGC15407 COQ4 CTNNB1 RPL24 /// SLC36A2 TUSC3 MKNK1 EIF3S10 PRCP EIF1AX AKR1A1 BMP1 FAM57A POLR2F MCAM TAGLN PIK3R3 UGT2B7 LRP1 DMWD RPL13 C9ORF10 U2AF1 SLPI MTF1 FOXJ1 ACP6 TTC10 ADM DNAJB12 SNX17 VWF RARS DGCR2 NSFL1C PMP22 ARAF G1P3 C21ORF33 SPA17 TAF1B ARF5 APP CUEDC1 MGMT GRB7 ELP3 SERPINA6 GRM4 RAE1 ST13 EIF2S1 NPC2 ZNF516 VGLL4 PSCD2L PRDX6 PHYHD1 PSMF1 ITGB1 GRB14 LGALS8 MLLT3 HMOX2 COL4A2 CPT2 HGD TATDN2 PPAP2B KARCA1 SERPINA2 EDN2 CDH3 STK25 PTGER1 SHC3 PRCC SAT HLCS SREBF1 GP2 PEX19 MUC1 CPM SEC13L1 CXADR KPNA2 C1R PHF16 ITGB5 ACVRL1 HDLBP RABIF CIB1 TJP2 GSTO1 IL20RA MYH9 AFF2 C1ORF122 MYO7A PPP1R11 IGHM EVI1 CNN1 FOXO1A DUSP4 PPP1R12B DAB2 PRDX2 CSN1S1 BAD PDCD8 CDC34 PAF53 PPOX

BHOJWANI_DIAG_VS_REL_B&T_35PAIR na ZNF211 CKS1B RTN3 NCALD PDHA1 ZNF274 CALM1 THUMPD1 TOP2A ZNF586 SHFM1 FADS3 EPS15L1 EIF3S10 NPM3 RNF113A ASAH1 HIST1H1C KIAA0368 HCAP-G DUSP6 HRK RBM10 TBCC BCL7A RPS12 F2R MRPS18B TGIF UBE2V1 /// KUA-UEV C6ORF79 NME2 SUI1 SNW1 FLJ10719 PRPF18 COX7C FLJ14346 C6ORF82 RRAGA BTG1 UBC CBX5 CXCR4 RPL36A SLC25A13 PSRC1 CDC2 RPS19 COX7B LIMK2 TIMM13 CLEC11A PTTG1 UBAP2 PRDM4 NDUFB11 AP2S1 NPC2 GAS7 BTG2 MMP9 RPS24 ZNF468 KNTC2 TRAF6 RAD23A KLF7 UQCRB LEPROT TCF3 ORC6L KPNB1 SMARCC1 LOC146909 ATP5G2 C20ORF3 ANP32B BLVRA PCDH9 MYNN SHMT2 SRP46 UQCRH P2RX5 NDUFB3 BAG5 BIRC5 PANX1 KIF2C PRKRIR CDC20 C6ORF62 NDUFS5 C14ORF2 RPL22 RPLP2 PFDN5 ACP1 MGAT2 PKP4 RPLP1 FOS RPS21 ZNF688 KIF4A STK6 SEMA6A

BHOJWANI_DIAG_VS_REL_PREB_32PAIRS na ZNF211 RPL32 CSDA CKS1B RTN3 PDHA1 ZNF274 CCNB2 CALM1 THUMPD1 SSR2 TOP2A ZNF586 DLEU1 SHFM1 EPS15L1 RRM1 GNA12 HIST1H1C CDC27 KIAA0368 HCAP-G C14ORF32 DUSP6 HRK BCL7A TBCC RPS12 F2R MRPS18B UBE2V1 /// KUA-UEV SUI1 NME2 SNW1 FLJ10719 PRPF18 RGS10 CGI-37 COX4I1 COX7C C6ORF82 FLJ14346 RRAGA DDX52 BTG1 UBC CBX5 CXCR4 RPL36A RPS19 CDC2 PSRC1 COX7B UBXD2 LIMK2 TIMM13 PTTG1 PRDM4 NDUFB11 AP2S1 NPC2 GMPS GAS7 BTG2 MMP9 RPS24 NP PPP1R15A KNTC2 RAD23A TRAF6 KLF7 UQCRB NRAS ORC6L RPS16 KPNB1 GYPC ATP5O ALOX5 LOC146909 KIAA0101 C20ORF3 RPS29 SFRS7 BLVRA PCDH9 SHMT2 SRP46 UQCRH P2RX5 BAG5 BIRC5 PANX1 KIF2C DUSP1 ADAMTS9 PRKRIR CDC20 C6ORF62 NDUFS5 C14ORF2 RPL22 AKR1C1 PFDN5 RPLP2 ACP1 RPS20 MGAT2 RPLP1 FOS RPS21 ZNF688 KIF4A STK6

BHOJWANI_DIAG_VS_EARLYREL_23PAIRS na ZNF211 C22ORF18 NDUFB11 AP2S1 CKS1B FEN1 PDHA1 CCNB2 ZNF274 KNTC2 PA2G4 TOP2A CLK1 DLEU1 FADS3 EPS15L1 EIF3S10 HMGB1 RRM1 NPM3 DDX3X MKI67 ZBTB10 HCAP-G ALOX5 LOC146909 KIAA0101 ANP32B BCL7A BRE SHMT2 CHAF1A MRPS18B BIT1 TPT1 SUI1 NUP62 FLJ10719 LY86 BIRC5 KIF2C FLJ22222 FLJ14346 PRKRIR CDC20 C6ORF62 CBX5 CD69 BRRN1 PRDX2 CDC2 RPS19 ACP1 EDG1 RALBP1 TAP1 RPLP1 FOS PSMD1 PTTG1 UBAP2 PRDM4 STK6

BHOJWANI_EARLY_VS_LATE_PREB_BINARY na TUBA6 SPFH1 MONDOA PSMD14 CKS1B GLO1 ST3GAL1 CCNB2 LILRA2 PP TOP2A TACC3 PLK4 TYMS GNG5 NUSAP1 CERK LSM3 CARS ICT1 COPS8 HCAP-G ERG CLK4 P4HB RECK INSR ACTR5 ACAT1 FBXL11 MTHFD1 CENTD1 H2AFZ QDPR BRRN1 SULT1A3 /// SULT1A4 SHCBP1 GAK GTF2A2 CCNB1 TCF4 GRB10 PTTG1 ACOT7 PABPC1 CD33L3 CKS2 DECR1 MAD2L1 ANXA2 MAC30 SORD TRAP1 KNTC2 MRPS35 SEMA3F HSPC047 COX15 LMBR1L POLE2 CDKN3 LDHA HPRT1 FABP5 ADK EIF4EBP2 MKI67 PRPS2 K-ALPHA-1 KIAA0101 GNPTAB CHD7 JMJD2B SULT1A1 HSPA8 AP3D1 KIAA0913 TPX2 FLJ22635 ELK3 ADRBK2 UCHL3 AHCY SSRP1 LDHB BIRC5 TTK ITPR1 ACTG1 KIF11 PCMT1 TRIAD3 RSU1 ZNF198 CSNK1G2 PAICS GDPD3 UBE2C DNCL2A HA-1 ARL6IP KIF4A

BHOJWANI_EARLY_VS_LATE_PREB_LINREG na SPFH1 MGAT4A SLC16A1 MRPS27 JTV1 MAP3K8 LILRA2 UQCRC2 PP PA2G4 HK2 FLJ10774 HSPD1 KARS SHFM1 KIAA0564 NME7 TNFSF10 ICT1 COPS8 HCAP-G ERG CLK4 NAP1L1 LRPPRC ADSL ATIC MTIF2 C14ORF166 PCLO GOT2 NME2 ACAT1 SEC11L1 COL5A1 C1QBP BUB1 MRPL3 PSMB7 SAE1 GAK HUWE1 GADD45B TCF4 ACOT7 NIT2 PCCB C6ORF66 ENO1 CCT6A MRPS35 CCT5 SEMA3F SSBP1 HADHSC LDHA RPL4 HNRPA1 /// LOC120364 POLR2D ETFA HLA-F PARK7 ATP5B HFL-B5 ADK IPO7 MKI67 ALOX5 ATP5F1 RAD51 IMMT MRPL18 TRIP3 EIF3S1 HSPA8 MTX2 ATP5A1 DDB1 BRE RUVBL1 TPX2 KIAA0913 ELK3 MGST2 ALDOA MULK DNAJC7 UCHL3 CTPS LDHB AHCY SSRP1 C12ORF8 SMYD3 CCT2 ACTG1 KIF11 FLJ36166 PAICS PACS2 UBE2C MTMR2 RPLP1 RGL1 EEF1B2 NPM1

TOREN_UP_IN_CD133 na BAALC MAN1A1 DSU FBN1 OBSL1 SPFH2 MEST MEIS1 ABCC1 ENOSF1 CYLN2 KIAA1305 EFHC2 SEPT6 /// N-PAC ATP9A FLNB C21ORF25 HSU79274 ANGPT1 GOLGA1 NME7 NGFRAP1 C14ORF110 ADCYAP1 ARHGAP22 CLGN MGC3032 GSTM5 ERG STOML2 MYH10 TSPAN3 DEPDC6 GCSH RCN1 TRIP6 CACNB2 TARBP1 EGFL7 CDK2AP1 IL12RB2 BST2 KIAA1043 PHGDH FAIM PLOD2 HOP CRYGD TIMM13 TCF4 NBL1 DST PLSCR4 MLC1SA PON2 EHD2 PGDS PROM1 KRT18 LTBP3 CMAH CASP6 MAC30 PDZK3 LRBA NPR3 ISYNA1 SH3BP4 SSBP2 FSCN1 HMGA2 NPDC1 FADS1 MYB FLJ13197 TGFBRAP1 VAV3 IGLL1 SERPINE2 KIAA1815 REC8L1 C21ORF96 NDN TCEA2 KDELC1 SCRN1 MCCC1 GATA2 PAWR DKFZP434A0131 MLC1 MGC39900 TSC22D1 MAST4 PLS3 SMYD3 CHD9 KIT SEPT6 PKD2 HNRPA0 PAICS ARMCX1 ZNF6 AKR1C3 SEPP1 LPIN1 CDK4 KIAA1797 EXTL2 PLEKHA5 FLT3 NPM1 FANCL NUDT11 TXNRD3 DAPK1 ELMO1 NME1 TCEAL4 FHL1 THSD1 /// THSD1P APEX1 C11ORF8 DSG2 KIAA0125 UNG SYPL1 PPM1H GNAI1 HLF CFH /// CFHL1 HDGFRP3 TNFSF4 TGIF2 KIAA1102 TFPI SOCS2 NY-REN-58 SCCPDH TENS1 MYCN ZNF447 DPPA4 ALDH18A1 BZW2 ANKRD6 SERPING1 FLJ14054 HOXB2 LOC150759 FSTL1 ALDH6A1 PTPLA ANKRD28 JARID1B ALDH1A1 CD34 LOC81691 SV2A CEP70 HINT1 PHACTR4 MDK CPA3 F2RL1 MSH5 DDAH2 RABGAP1 SCHIP1 FRMD4B ASB9 PDGFC MLLT3 CD200 CRHBP SLC39A8 ME3 WBP5 HOXA5 HSPCB RNF144 LOC81558 HTR1F LOX MAP7 HSPB1 FABP5 LENG4 ADAM28 SPINT2 NAP1L3 DPYSL3 BLMH MYO5C PHF16 PLCB4 SLC35F2 RBPMS B4GALT6 SMARCA1 ITM2C SPINK2 TIE1 CCNB1IP1 RUNX1 ATP2C1 MAGED1 NPTX2 EVI1 HOXA10 TSPYL5 LAPTM4B EFHC1 FZD6 CYTL1 TRIM24 C5ORF13 MPL GUCY1A3 FLJ36166 BCL11A DNMT3B IL18 CBFA2T3 HOXA9 KCTD3 GABPB2 BTBD3 NRIP1 PLAGL1 SEPT11 PPP1R14B BSPRY IMPDH2

TOREN_DOWN_IN_CD133 na CD3Z KCTD12 IFI30 LYST CORO1A FCN1 BCL11B CALM1 LILRA2 C5ORF18 CEBPD PILRA GPR65 ITK NID1 ZFP36 KLRF1 C6ORF1 FYB IQGAP1 KYNU CLEC4A MNDA CNIH4 KLRD1 TBC1D8 FLJ20273 CD48 CKAP4 HK3 LILRA3 FYN TSPAN14 RNASE6 RAB27A ELA2 MS4A1 EGFL5 KLRK1 CD3G IL7R CD1D HSPA6 SLC15A3 PSTPIP1 TSC1 FCGR2C LPXN IL18RAP CD96 XCL1 /// XCL2 PIP3-E FLJ22662 NCF2 IGLC2 CSTA KIAA0882 NPL S100A12 IGF2R LCK CTSB KLRC1 /// KLRC2 CEACAM8 SECTM1 MAFB TGFBR3 TBX21 PTGDS OSTM1 CD2 PRKCA NADK TLR4 IL13RA1 TFEB PRTN3 PARP8 IL1RN PRF1 PSAP GPR171 CD8A ALOX5 DEFA4 SGSH CDA MS4A3 FPR1 KLRB1 TNFRSF7 SYNE2 CST7 FAIM3 CD14 CEACAM6 S100A9 GZMH GNLY CD7 TRA@ CTSS XCL2 MAN1C1 PRKCD PLEC1 FOS CD36 MGAT4A SLC7A7 LILRB2 DOK2 CD8B1 LGALS2 AZU1 HCK TRBV19 /// TRBC1 GZMK FPRL1 RORA LILRB2 /// LILRB3 CRTAM CCR7 TRD@ NELL2 AQP3 ALOX5AP FCGR3B CST3 TRAT1 ADAM19 CHST2 ITGB7 SAMHD1 C11ORF17 /// NUAK2 TRAC LAT ZAP70 BCL6 CLEC10A PDXK TRA@ /// TRD@ COTL1 CLEC7A ZFP36L1 ACTN1 TLR2 NEDD9 IL4R LGALS3 /// GALIG PFC CSPG2 LILRB1 SGK BPI IL2RB MTMR11 NCF1 RBL2 RASGRP1 ENTPD1 TIAM1 VNN2 TRA@ /// TRDV2 /// T CD3E LILRA5 LR8 IL10RA ECGF1 TRA@ /// TRAC RTN1 FCGR3A /// FCGR3B GZMB IGL@ /// IGLC1 /// I FGR CD24 HNMT MYBL1 SIGLEC7 FGL2 STX11 UBE2D1 DEFA1 /// DEFA3 UGCG MYO1F CDC25B TGFBI TYROBP F13A1 SUCLG2 SERPINA1 IL32 FCER1G FBP1 CD163 CTSG ITGAX SLC11A1 CD86 NKG7 RAB31 SCPEP1 LY86 GZMA CD3D ITGAM BLVRB DUSP1 RNASE2 CYBB CPVL CCL4 LEF1 RNASE3 CSF2RA CCL5 SMPDL3A FCGR2A QPCT ITGB2 CTSH CSF1R MS4A6A

TOREN_COMBINED_CD133 na MAN1A1 DSU SPFH2 MEST CORO1A ENOSF1 LILRA2 C5ORF18 CYLN2 GPR65 ITK ZFP36 FLNB ATP9A C6ORF1 KYNU CLEC4A MNDA CNIH4 GOLGA1 NGFRAP1 NME7 C14ORF110 KLRD1 TBC1D8 CD48 ADCYAP1 CKAP4 ARHGAP22 CLGN ERG STOML2 TSPAN14 RNASE6 TSPAN3 RAB27A ELA2 DEPDC6 GCSH RCN1 CD3G CD1D TARBP1 PSTPIP1 TSC1 FCGR2C IL18RAP KIAA1043 CD96 TCF4 PIP3-E IGLC2 PLSCR4 MLC1SA CSTA PON2 PGDS S100A12 KRT18 IGF2R LCK CTSB LTBP3 CMAH KLRC1 /// KLRC2 CEACAM8 SECTM1 MAC30 PDZK3 LRBA NPR3 ISYNA1 SH3BP4 TGFBR3 FSCN1 HMGA2 NPDC1 FADS1 TBX21 MYB PRKCA NADK TLR4 TGFBRAP1 IL1RN PSAP GPR171 CD8A DEFA4 SGSH CDA FPR1 REC8L1 TCEA2 NDN SYNE2 MCCC1 GATA2 CST7 DKFZP434A0131 MLC1 CD14 SMYD3 GNLY PLS3 CHD9 CD7 XCL2 MAN1C1 PAICS AKR1C3 LPIN1 KIAA1797 NPM1 PLEKHA5 FANCL MGAT4A LILRB2 NME1 DOK2 CD8B1 FHL1 DSG2 HCK PPM1H HLF TGIF2 CCR7 TFPI KIAA1102 TRD@ NELL2 ALOX5AP CST3 TRAT1 ADAM19 CHST2 TENS1 SAMHD1 C11ORF17 /// NUAK2 ZAP70 TRA@ /// TRD@ COTL1 CLEC7A ZFP36L1 TLR2 SERPING1 FLJ14054 NEDD9 IL4R LGALS3 /// GALIG PFC CSPG2 ALDH6A1 RBL2 ANKRD28 RASGRP1 ALDH1A1 SV2A MDK LILRA5 LR8 DDAH2 FRMD4B ASB9 PDGFC IGL@ /// IGLC1 /// I HNMT SIGLEC7 RNF144 HTR1F DEFA1 /// DEFA3 LOX MAP7 HSPB1 MYO1F TGFBI DPYSL3 BLMH SERPINA1 IL32 RBPMS FCER1G B4GALT6 CD163 SPINK2 TIE1 CCNB1IP1 RUNX1 ATP2C1 NKG7 CD86 RAB31 MAGED1 GZMA CD3D FZD6 EFHC1 ITGAM DUSP1 TRIM24 RNASE2 CYBB FLJ36166 CPVL CCL4 LEF1 CSF2RA DNMT3B CBFA2T3 BTBD3 NRIP1 ITGB2 CTSH CSF1R MS4A6A PPP1R14B BAALC KCTD12 CD3Z FBN1 OBSL1 IFI30 LYST MEIS1 BCL11B FCN1 ABCC1 CALM1 KIAA1305 CEBPD EFHC2 PILRA NID1 SEPT6 /// N-PAC KLRF1 C21ORF25 FYB IQGAP1 HSU79274 ANGPT1 FLJ20273 HK3 MGC3032 GSTM5 FYN LILRA3 MYH10 MS4A1 EGFL5 KLRK1 TRIP6 IL7R HSPA6 CACNB2 SLC15A3 EGFL7 CDK2AP1 IL12RB2 BST2 LPXN FAIM PHGDH PLOD2 XCL1 /// XCL2 HOP CRYGD TIMM13 NBL1 FLJ22662 NCF2 DST KIAA0882 NPL EHD2 PROM1 CASP6 MAFB SSBP2 PTGDS OSTM1 CD2 FLJ13197 IL13RA1 TFEB PRTN3 VAV3 PARP8 IGLL1 PRF1 SERPINE2 ALOX5 MS4A3 KIAA1815 KLRB1 C21ORF96 TNFRSF7 KDELC1 SCRN1 FAIM3 PAWR MGC39900 CEACAM6 TSC22D1 S100A9 GZMH MAST4 KIT TRA@ CTSS SEPT6 PKD2 HNRPA0 ARMCX1 ZNF6 PRKCD SEPP1 CDK4 PLEC1 FOS EXTL2 FLT3 NUDT11 TXNRD3 CD36 DAPK1 SLC7A7 ELMO1 TCEAL4 LGALS2 THSD1 /// THSD1P APEX1 AZU1 C11ORF8 TRBV19 /// TRBC1 GZMK KIAA0125 UNG SYPL1 GNAI1 FPRL1 CFH /// CFHL1 RORA LILRB2 /// LILRB3 CRTAM HDGFRP3 TNFSF4 AQP3 FCGR3B SOCS2 NY-REN-58 SCCPDH ITGB7 MYCN TRAC LAT BCL6 ZNF447 DPPA4 PDXK CLEC10A ALDH18A1 BZW2 ACTN1 ANKRD6 LOC150759 HOXB2 FSTL1 LILRB1 SGK BPI IL2RB NCF1 MTMR11 PTPLA JARID1B ENTPD1 CD34 LOC81691 TIAM1 VNN2 CEP70 TRA@ /// TRDV2 /// T HINT1 PHACTR4 CPA3 F2RL1 CD3E MSH5 RABGAP1 IL10RA SCHIP1 ECGF1 TRA@ /// TRAC RTN1 FCGR3A /// FCGR3B GZMB MLLT3 CRHBP CD200 FGR SLC39A8 ME3 CD24 WBP5 MYBL1 HOXA5 FGL2 HSPCB STX11 LOC81558 UBE2D1 UGCG CDC25B FABP5 ADAM28 LENG4 SPINT2 NAP1L3 TYROBP F13A1 SUCLG2 MYO5C PHF16 PLCB4 SLC35F2 FBP1 SMARCA1 CTSG ITGAX ITM2C SLC11A1 SCPEP1 NPTX2 LY86 EVI1 HOXA10 TSPYL5 LAPTM4B CYTL1 BLVRB C5ORF13 MPL GUCY1A3 RNASE3 BCL11A CCL5 IL18 SMPDL3A HOXA9 FCGR2A KCTD3 GABPB2 QPCT PLAGL1 SEPT11 BSPRY IMPDH2

LAMB_COMBINED_HDACI na FUCA1 MT1X ANP32B CTPS GLRX TUBA3 TYMS HIST1H2BG DHRS2 KPNB1 CLU

LAMB_COMBINED_ESTADIOL na EGR3 STC1 EPB41L3 IFI30 ARNT2 LAMB1 SMPD1 HOXC4 /// FLJ12825 TOP2A STXBP1 MB CA12 KYNU CDC6 TM4SF1 GNE ABCC5 NELL2 WISP2 GNS BMP4 ENC1 MXD4 EPOR ERBB3 LHFPL2 PIB5PA ECH1 ERBB2 FMO5 CTSL AREG FOLH1 CXCL12 MMD CALCR PRPS1 RBL2 RASGRP1 NFRKB RET IL1R1 RFC4 BIK SNCG SLC16A5 KIAA0922 TGFB2 L1CAM INHBB KRTHB1 ASB9 ADCY9 CD24 BAK1 /// BCL2L7P1 MYBL1 BLNK MYO1B EFNA1 CLU GLRB CYP1B1 PTGES MUC1 SELENBP1 TGFB3 BCAS1 TFF1 CBX6 RAP1GA1 GREB1 ANXA3 PVALB DBN1 AP1G1 IGFBP4 IER3 IRF6 RAB31 SLC22A5 SLIT2 PEG10 DUSP1 CYP1A1 KRT7 COL4A5 DUSP4 ALDH4A1 NDRG1 PDZK1 PPP1R3C SDC2 LAMB2 MAPT SIAH2 MATN2 FOS CTSH NRIP1 ABCG1

LAMB_COMBINED_PHENOTHIAZINE na MGC11335 HRB2 ATF3 IDI1 SC5DL UBE3A MINA CUTC KIAA0984 TTC15 PTGDS DKC1 BOP1 NUFIP1 BMPR2 JUND DHODH LRFN4 METTL5 MHC2TA RBM8A TU3A PRPF31 BRIP1 SLC19A1 PAK1IP1 PRR5 FDFT1 SPATA5L1 DIO2 SQLE PCF11 MRPS2 TBRG4 SDC3 PNN SEMA6B FAM61B SC4MOL CGI-37 FLJ12571 SKP2 HMGCS1 MRS2L INSIG1 RPP40 PRPF4 DKFZP564I122 LENEP USF2 ZYX HCG4P6 FLJ13984 ELMO3 GDF15 LPIN1 ABCB9 ISG20L2 SFRS3 ODC1 STK6

LAMB_COMBINED_PHENOTHIAZINE_ALT1 na TTC15 KIAA0984 MGC11335 INSIG1 PTGDS HRB2 RPP40 ATF3 NUFIP1 DKFZP564I122 LENEP IDI1 JUND USF2 ZYX LRFN4 MHC2TA GDF15 CGI-37 SC4MOL FLJ12571 MRS2L SQLE

LAMB_COMBINED_PHENOTHIAZINE_ALT2 na MGC11335 HRB2 ATF3 IDI1 ARVCF FGFR3 FLJ13909 TRGC2 /// TRGV9 /// UBE4B OPRS1 SMARCD1 TNIK SLC17A7 TTC15 KIAA0984 PTGDS NUFIP1 JUND COX5B SYNGR1 LRFN4 MHC2TA ETV5 SGSH MAST2 NMT2 DIO2 SQLE C7ORF28A /// C7ORF28 SFRS7 TBRG4 SLC35E3 FLJ14627 PPP2R1B NFAT5 DAPK3 SC4MOL CGI-37 FLJ12571 SKP2 PAFAH1B1 MRS2L ACD INSIG1 RPP40 TRADD DKFZP564I122 KIAA0690 LENEP EXOSC9 USF2 C1ORF181 ZYX PRDX2 FAM26B GDF15 PRKAB1 CEBPE DHRS2 LDLR

LAMB_COMBINED_PHENOTHIAZINE_ALT3 na S100P AKAP9 ATF3 OKL38 NXT2 LARS2 SLC25A36 UBE3A MLLT10 ZNF189 FADS1 LMBR1L C14ORF1 GEM PGS1 PDCD4 JUND ANP32A RAI17 ATXN1 ID1 FBXO46 TP53 TLK1 ANXA1 ZNF14 CALML4 JMJD2B ARIH2 ERBP CD44 ALAD RFC2 HEXIM1 BRCA2 NUP62 NBR1 SC4MOL CEBPA FKBP4 ING3 FOXO1A TMEM28 INSIG1 CYP1A1 NUCB2 C9ORF16 GDPD3 ARMCX1 DMN IRS2 RIMS3

LAMB_COMBINED_GEDUNIN na S100P GNL3L MAPK6 TDP1 ATF3 RGS3 DDX46 HSPH1 GTPBP2 MGC11308 STIP1 STCH ZC3HAV1 SOX4 PSD4 BYSL DNAJB4 RECQL C1ORF103 FLJ10700 RFX5 PPAP2B COG2 MYC MAP1LC3B SCO2 DLG1 CRYAB DNAJA1 RRS1 POLR1C SNAPC4 DDIT4 RRM2 SMARCA4 AKAP1 DNAJB1 MGC14376 C20ORF111 ALG6 MRPL18 ELAC2 EIF5 TBRG4 GEMIN4 HSPCA HSPA1A /// HSPA1B NKTR UMPS ZNF692 TSC22D3 PHF20 MGC4504 TARSL1 HSPA6 BIRC5 LCMT2 CHORDC1 PWP2H PRPF4 WBSCR20C ANAPC2 DNAJC11 KIAA0859 LOC400506 RANGAP1 NUP50 HBEGF HNRPA0 AKR1C1 HSPB8 CACYBP DNAJB6 BAG3 POLRMT CDC7 AKR1C2 DNAJB9 DDIT3 RAPGEF2 SERP1 CSTF1 ABCE1 CDKN1A IMP3

LAMB_COMBINED_OBESITY na CD36 GHR GSTP1 ETFDH CNP PRKCDBP VIL2 PSEN2 LEP OXTR SLC12A2 NDRG4 CD38 TSHR LRP2 APLP2 SYNJ2 PIK3R1 CPT1A HYOU1 KRTHA6 PBP CRYAB PDHA2 NGFRAP1 NME7 LDB3 ACY1 PDHX TST CHKB GNA12 CHKB /// CPT1B BF UCP3 CHP SLC2A2 PTPRF HDAC6 ID2 PSMB4 FDFT1 SLC15A2 APOBEC1 COL15A1 MTCH2 BICD2 TSPAN3 CX3CL1 MFGE8 RNF130 CST8 RUSC1 CPA1 FGFR4 AQP7 AKT1 ZNF692 OLFM1 LXN EHHADH GGTLA1 ITGA7 GSTM3 WBSCR1 ST3GAL2 COL11A2 COX4NB ALDH1L1 PDE2A CDS1 MGLL PEMT TKT MAP1A ST6GAL1 SLC37A4 CAT CITED1 QDPR ALDH1A1 APOC1 PTN C17ORF41 OIP106 PSMB8 SPOCK2 PENK PFKM FURIN MECR DLAT KRT18 GM2A SLC29A1 CA5B CLCN3 BASP1 PPARG NCKAP1 DNAJC4 NPR3 BTG3 ACOX1 SCARB1 MARK1 PFKFB1 ADRB3 SCD LCN2 EDG2 CLU SCTR SLC1A1 HSPB1 RBBP7 PAX8 FABP5 SPINT2 PTER LOXL1 KPNB1 PAPSS1 PGRMC1 SPP1 NOL3 PRG1 DNAJB1 PEX11A CYP4B1 PDIA4 CD82 USP12 ACSL6 PAWR MGST2 ALDOA P2RX5 OAT CCL13 PGM5 PSAT1 ATP1A1 HMGCS1 SLC11A2 PICALM RNF4 DUSP1 ADFP INSIG1 CEBPB COQ3 TGOLN2 TMEM53 RBP1 RCN2 PLCD1 C1ORF9 SLC25A1 RBP4 GAMT ATP1B1 AKR1B1 PC FMO1 SLC12A4 RGC32 SIAT4B LITAF ITPR3 EGR2 GOLGB1

LAMB_COMBINED_ALZHEIMERS_HATA na HMMR CLCNKB CD36 LAMB1 LCK TNFRSF8 TGM4 COL10A1 EFEMP1 GNG11 MCM3AP ACSL4 PCK2 FGL2 COL11A1 AVPR1A PDCD11 ART3 PPP3CB DEFA1 /// DEFA3 CAV2 RPS15 RANBP1 AKAP8 PHKG2 HBLD2 GBP2 RPS27 TM4SF5 CSN1S1 RANBP1 /// LOC389842 ACOT2 MYH8 POU2F1 DPYS MCM3APAS S100A4 EGR2 ADORA2B FCER1G SEC14L2

LAMB_COMBINED_ALZHEIMERS_RICCIARELLI na RPL21 FOXN1 AD7C-NTP IFITM3 EIF5A PPP1R8 RPL41 ALOX15B FXYD6 TUBB GLS RPS24 GAP43 FAIM2 SGCB PLEKHA1 JAK3 ACTA2 DCHS1 ACTR1A ACTB

LAMB_DEX_UP na RPL27A POU5F1 /// POU5F1P1 OR2H2 VIL2 ACOXL DCTN1 LRCH1 C15ORF25 CYP3A4 ADAMTS2 ZNF611 /// LOC388558 LOC200205 DHRS7 AIP DHX9 KIAA1109 SLC35C2 CYB561 MR1 B3GAT3 SCIN ZNF160 PCYT2 TCF3 WDTC1 FAM13A1 NCR1 KIAA0894 SLC9A2 HDGFRP3 MYOZ3 APBA3 DBT CENPB RPS20 MYST4 C14ORF105 SLC4A5 MKI67 OSBPL10 MGC2731 CALCA PAX5 ADAT1 ABHD9 GATAD1 WNT16

LAMB_DEX_DOWN na YWHAZ PON2 FLJ13448 CRADD HSPA9B CALM1 NP TMEM50B SLC39A8 C15ORF15 RAB8B UBE2N ZFP36 C6ORF120 RPS6KB1 RAB21 DAF SPAG9 DNAJA1 BZW1 /// LOC151579 C22ORF5 PDE4B OSBP BZW1 RTN4 EIF1AX GNAI3 SLC12A6 DNAJA2 SLC25A37 TFRC METAP2 SNF1LK NR4A2 MCL1 MGC14376 SPRED2 SEC24A PHLDA2 G3BP2 DXYS155E IFRD1 SLC38A2 MAX PLEKHC1 EIF5 BHLHB2 PLAUR LCP2 YWHAE H41 KLHL18 RIPK2 CAPZA2 CD164 MAP2K1 RYBP IFNGR1 GSPT1 NR4A1 ACTR3 INSIG1 CHIC2 SGK SLC2A3 NEU1 PRNP TXNDC4 MAPRE2 NR4A3 DNAJB6 PVRL2 CHSY1 PBEF1 DYRK1A LAT1-3TM /// IMAA // ARF4 SFRS5 EMP1 LOC57228 MAPK1 FOS TSC22D2 CDKN1A RCHY1 ARL6IP FOSL2

LAMB_COMBINED_DEX na POU5F1 /// POU5F1P1 VIL2 FLJ13448 CALM1 C15ORF15 ZFP36 SLC35C2 RAB21 RPS6KB1 DAF C22ORF5 EIF1AX RTN4 GNAI3 HDGFRP3 MYOZ3 SLC25A37 CALCA ABHD9 GATAD1 PHLDA2 DXYS155E IFRD1 SLC38A2 RPL27A OR2H2 EIF5 ACOXL LCP2 YWHAE LRCH1 C15ORF25 RIPK2 AIP MAP2K1 RYBP IFNGR1 GSPT1 NR4A1 ACTR3 SGK NEU1 PRNP TXNDC4 NCR1 PVRL2 CHSY1 PBEF1 ARF4 DYRK1A EMP1 TSC22D2 ADAT1 CLEC2B RCHY1 WNT16 YWHAZ PON2 CRADD HSPA9B LOC200205 ZNF611 /// LOC388558 NP TMEM50B SLC39A8 RAB8B UBE2N DHX9 C6ORF120 CYB561 MR1 SCIN BZW1 /// LOC151579 ZNF160 TCF3 DNAJA1 SPAG9 WDTC1 PDE4B OSBP BZW1 FAM13A1 SLC9A2 KIAA0894 SLC12A6 DNAJA2 DBT C14ORF105 MYST4 MKI67 TFRC METAP2 OSBPL10 SNF1LK NR4A2 MGC2731 MCL1 SPRED2 MGC14376 SEC24A G3BP2 MAX PLEKHC1 PLAUR BHLHB2 DCTN1 H41 KLHL18 CYP3A4 ADAMTS2 CAPZA2 DHRS7 CD164 KIAA1109 B3GAT3 INSIG1 CHIC2 SLC2A3 PCYT2 MAPRE2 NR4A3 DNAJB6 APBA3 CENPB RPS20 SLC4A5 LAT1-3TM /// IMAA // SFRS5 LOC57228 PAX5 MAPK1 FOS CDKN1A ARL6IP FOSL2

GYORFFY_5FU na EPHX1 PCOLCE2 PHYH RNASE4 CBX1 LGALS3BP SEPHS2 MYL9 ZNF532 CUTC ELOVL5 COL5A2 C20ORF35 IL27RA ASAH1 XAGE1 KLHL7 NTS ANXA1 FNBP1 ALS2CR8 PTPRK FYN CCNG1 LAMC1 TP53I3 FKBP11 OXCT1 GPR126 ADD3 PMAIP1 FKBP1B HIST1H2BK FZD2 HIC2 LIMK2 DST USP9X EHD2 TGFB2 CKIP-1 CPD SLC29A1 ALDH3A2 NETO2 MICA /// MICB PLP2 FADS1 PNMA2 POLE2 GCLM UGDH LSS CAP2 TMSB4X /// TMSL3 COCH TGFB1 MAGEA4 C10ORF38 NRG1 PARD3 LARP5 CSF1 THEM2 PEG10 ADFP FGG C11ORF24 KIAA0859 FGD6 STRA13 COL6A3 M6PRBP1 GALNT3 LASP1 THBS1 AKR1C3 MGAT2 FLRT2 SEC14L1 SPANXC MAGED4 FHL2 NME4 RGS4 SOAT1 ACSL1 CFLAR TRIOBP GLA UCHL1 SOX18 KLHL9 STK38 EFHD1 PLA2G4A VCL GJA1 DPAGT1 SEC31L1 TFPI NEDD4 TAGLN TENS1 ITGAV LPHN2 SNX6 ZNF447 ARID5B APBB2 CRK ADM PDLIM5 LXN LGALS3 /// GALIG CHST7 ANG /// RNASE4 CASP4 OPTN MARCKS PTPLA M-RIP MAFF ALDH1A1 APOC1 CSRP1 TBCE TPST2 CYFIP2 ANGPTL4 RAB20 YIF1A CXX1 CKB CDC42EP1 DDAH2 IDI1 GSTA4 ABCC2 CLCN3 MICA FNDC3B GPR56 SEC23A C1ORF2 DPY19L1 SCD GTPBP4 GENX-3414 MXI1 FTL TGFBI SPANXA1 /// SPANXB1 CYP51A1 COL3A1 SEPHS1 NXN MICB RRAS DKK1 TPM1 OGFRL1 BBX EFNB2 SMARCA1 PLEKHC1 EDIL3 TRIM2 MAGED1 ITSN1 BRP44 LDHB BHLHB3 BIRC5 BLVRB FLJ10847 MLLT11 ECHDC3 SLC2A3 EBP PBX3 RIT1 SLC2A3 /// SLC2A14 AKR1C1 PLSCR1 BAG3 AKR1C2 PPP1CA CDC34 HSPA2 TUBD1 ENAH SGCE CABYR

GYORFFY_CISPLATIN na FGFR2 C20ORF18 ATP6V1G2 IFI30 MEST SH3BGRL3 ABR RAD21 SPTLC2 LOC440270 AFF1 SYNJ2 BLES03 RBM9 FKBP2 PTGS1 FLJ20366 NMU TRIP13 FADS3 DAF PFKP UGT1A10 /// UGT1A8 / TNFSF10 CKMT1B /// CKMT1A NDUFS8 UPK1B MAL CCNE1 KIAA1102 CAV1 LNK IRAK1BP1 CCNG2 GALNT7 UCP2 SOCS2 ANXA1 POP4 FMOD ALS2CR8 CORO1C ABHD2 C19ORF2 SOD3 ID4 DEPDC6 EMP3 ARID5B CTNNAL1 SLPI PDXK STEAP1 HLA-G CNTNAP2 GSTM1 TP53I3 CILP ZFP36L2 PMS2L11 SLC20A1 GSTM3 TFAP2A NEDD9 PXDN NR4A1 GSTM2 GPAA1 GPX3 OPTN ITPA KCNK1 DIPA SLC7A8 VAPA DDR1 CA2 ICAM1 PROS1 HPCAL1 PRKCI RAB40B IL1R1 RPS14 CYP2C9 BMP7 DST TXNDC13 KLK5 MACF1 FOSL1 DRAP1 CTH LOH11CR2A PAPPA2 PRODH MAP2K6 HHEX F8A1 ASS BTG2 CELSR2 SURB7 PBX1 PLP2 HLA-A GLRX2 FSCN1 DMBT1 LTBP1 CDH3 SPON1 FAM46A PAX8 ABHD11 FTL FAM13A1 KIBRA IFITM1 SLC7A11 VAV3 FOXC1 MT1F BACH1 SERPINE2 EHD4 TMSB4X /// TMSL3 UGT1A6 AKAP12 HNRPA3 IGFBP5 DKK1 SWAP70 NAB1 LANCL1 C1ORF24 TGFB1 CES2 CALCB TFB1M SULT1A1 WFDC2 AHCYL1 GSTO1 TRIB3 GARS FGF9 SCRN1 GOLGA8A ARMCX2 RNASET2 ADAM10 HSD17B8 MAP4K4 LAPTM4B SULT1A2 TUBB2 PHLDA1 GAS1 ARF6 SLC6A12 KRT17 ADFP TNCRNA COL4A5 ENPP2 MPP2 TSPAN12 NOTCH2 YARS ZNF192 KLF2 SDC2 GCNT2 CTAGE4 /// LOC441296 LEPREL1 LU FLJ21106 GNAS GUCY1B3 FOS ADORA2B

GYORFFY_CYCLOPHOSPHAMIDE na PRSS3 MAGED4 S100P MYH10 ELOVL2 SOAT1 ADM IGFBP3 FNDC3B IMP-3 KLF5 MAGED1 OPN3 BRP44 SNRPN LDHB NID1 ZIC1 SALL1 GALC MXI1 AGL PTGS2 FKBP1B IF BDH GLS DDIT4 TMEFF1 SPP1 HOXA1 TPM1 HSPA2 COCH SGCE

GYORFFY_DOX na HCLS1 AURKB GSTP1 PRC1 MEST PSG4 FLJ11259 TOP2A SEPT6 /// N-PAC CHEK1 DLEU2 /// BCMSUNL NRP1 CKAP2 PLAU NUSAP1 CDK2 LPHN1 SOD2 RANBP1 LMNB1 DKK3 BNC1 CAV1 DSP RPS26 /// RPS26L /// HCAP-G FRMD4A MT1E DOCK4 KIF15 H2AFY FKBP1A EZH2 PRUNE G0S2 FLJ10719 ADD3 HSDL2 HMGB2 CAT CREG1 AXL IL8 HBEGF TACSTD1 HIST1H2BD PTX3 NUDT15 CDC2 ANKRD15 FLJ12443 PRKCI FLJ14668 ABLIM1 PITPNC1 ALDH7A1 HMMR GTSE1 LTBP2 ANKHD1 DLC1 MAD2L1 TMPO PIR MAC30 PKIA TTF2 WDR61 HMGN3 KNTC2 HMGA2 AOX1 PSG6 TGFB1I1 ZDHHC11 SERPINE1 EDG2 SFXN1 HADHSC C2ORF31 RAB3B BCL2A1 KIAA1598 KIAA1026 FN5 GGCX PGRMC1 GUSB AKAP12 DENND2A OSBPL10 KIAA0101 C10ORF38 DCC1 RFC3 MYCBP KLF5 TMEM14A TDG TTK LOC388650 RACGAP1 REV3L COL6A3 TRPC1 SEPT6 CTSS RAFTLIN ZWINT BBS4 ACP1 OIP5 KIF4A FANCL SNX10 CCNB2 C11ORF32 IGFBP7 CXCL5 FLJ20758 QKI BIRC2 MME NDUFA1 KIF1B NSMAF COX5A TSPAN13 AGL ATP5I BIRC3 IL1B FGF5 TFPI HIST1H1C KIF23 MCAM ID2 GPR C19ORF2 CENPA ERBB3 RBBP8 GPSN2 ACTN1 RIPK2 ZFP36L2 LSM7 DSC3 ELL3 STC2 RAGE RFC5 HIST1H2AC DIRAS1 INHBA SERPINB2 SORT1 TFPI2 FOSL1 FLI1 ACO1 FN1 EMP2 SYDE1 PSPH DONSON COX17 MICAL2 KIF20A ETFB LOX SUCLG1 ORC6L NUDT4 /// NUDT4P1 MT1G DZIP1 DKK1 GNPTAB NMT2 PRG1 TPM1 DAG1 DCPS SULT1A1 RAB11FIP1 C16ORF33 FLJ23235 TRIM2 IBRDC3 SRD5A1 SPR BAT2 MMP1 SKP2 GPSM2 FLJ21749 CDKN2C FLJ13910 APOBEC3B VEGFC RPA3 CDC7 PTD015

GYORFFY_ETOPOSIDE na S100P CST1 EPB41L3 MDFIC HDAC4 ABCC1 FHL1 MTUS1 ROBO1 ISG20 C19ORF28 DUSP2 IGFBP7 AMIGO2 BIRC2 HTATIP2 ACSL3 GPM6B EN2 KYNU SORBS2 TFF3 DAF CDK2 ZNF32 DCT MAGEA3 DKK3 CKMT1B /// CKMT1A ASAH1 BIN3 KIAA1102 TFPI MAGEA12 ALPP /// ALPPL2 FDFT1 FYN NAP1L1 COL15A1 C14ORF147 ROPN1B ERBB3 RASIP1 F2R SOX10 ATP6V0A4 TDRD3 MAGEA6 CHGN PBK TFAP2A NEDD9 PMP22 SLC7A5 WIPI49 GSTM4 LGALS3 /// GALIG CSPG2 GPR37 ST6GALNAC2 SLC27A3 PMP2 PIP5K1B LY6E RBM35A CPS1 KCNAB2 CADPS2 ANKRD28 PHGDH NES SILV SORT1 RAB20 SH3TC2 FLJ11193 TFPI2 TCFL5 MACF1 CTH MAGEC1 MUC13 MAC30 EMP2 PDGFC MBP DMD RAB17 BAMBI HMG20B HMGA2 HOXA5 SOCS6 SLC16A6 ZDHHC11 MXI1 EXOSC7 MAP7 COL9A3 FAM46A PDE4B FLJ11017 ACADM SORBS1 IFITM1 ALPPL2 DDIT4 AKAP12 SPP1 LIPA FOXF2 ALDOC FUT8 NMT2 CREM UBL3 LANCL1 C1ORF24 TRIB2 PPT2 AVPI1 C10ORF38 AHCYL1 DBN1 SLC22A18 TRIM2 DSCR1 FGF2 MOCOS ST7 NQO1 GSTT2 OCA2 HEPH ATP1A1 NAV2 GPSM2 TMEM47 PLP1 ITPR1 INSIG1 MLLT11 BACE2 TYRP1 DUSP4 EVER1 MLANA APOBEC3B ZP3 /// POMZP3 MBNL2 IRS2 SMPDL3A TNKS BAG2 MATN2 NRIP1 HSPA2

GYORFFY_MTX na ARFIP1 S100P CST1 ARL6IP4 SDF4 HDAC4 GPR172A EVL CREB3 ATF5 SH3BGRL3 ENOSF1 PIK3C2B IGFBP7 DGCR6 /// DGCR6L GPX2 ACTR1A CD99 H2AFX CPSF1 C20ORF45 KDELR1 PDK1 GNE MAGEA3 HDGFRP3 CKMT1B /// CKMT1A ZNF580 NDUFS8 CKAP4 SEMG1 PIN1 MAP2K2 PPID FNBP1 FMOD P4HB LPHN2 COL15A1 MFGE8 CLSTN1 CD302 PAPOLA TSPAN4 GCSH EGFL5 RAB1A HGS MYO1E BNIP3 SH3GLB2 SPG20 LAMC1 DAPK3 EPRS NFE2L1 ZNF175 PPRC1 FSTL1 EXOC7 GPX3 PCQAP KCNK1 BST2 CPS1 C19ORF6 CDK2AP2 ALPP THRAP5 PVRL2 PHF10 AIM2 RAPGEF2 ABP1 SLC26A2 SF3B1 SCAP1 HRB2 CSTA MCF2L CTH DRAP1 LOH11CR2A LCK MAP2K6 GAD1 KLRC1 /// KLRC2 PHACTR2 TTF2 MBD3 CGA TOB1 FADS2 HLA-A SLC27A2 FER DCP2 BAT3 FADS1 EDG2 USH1C PNMA2 CLPTM1 PPP2R5C PDLIM7 FAM62A MAP7 UGCG GRN CHES1 B3GNT3 TCF12 TLE1 SUCLG2 EI24 PSAP TRIM28 ALOX5 HDHD1A PTOV1 CIZ1 TLN1 ARHGDIA C10ORF116 PEX11A F12 CLN3 TGFB1 C10ORF38 CALR DBN1 KLK6 RBMS1 CD151 H1FX IRF6 EDG4 CST6 GNG11 MSLN MAGED1 HNRPH1 PTPRG NFKB2 UTX PPP3CA ITPR1 GNA11 DUSP4 EIF5A EED MPHOSPH6 NUP88 ANXA11 RAB11FIP2 PPAP2A PLD3 TMEFF1 GAMT RPL23A PI3 FLRT2 CNOT3 PER2 BTBD3 CDC34 DHX15 DHRS2 KCMF1 FGFR1 SGCE ATP5D WDR3 UBE1

GYORRFY_MITOMYCIN na HCLS1 WNT5A IMPA2 FHL1 NFYA EGR1 CXCL5 AMIGO2 MYLIP BIRC2 ACSL1 MME FLJ20366 ACSL3 UCHL1 EIF5B TFF3 MYO10 KIAA0648 IL1B PLAU CDK2 SOD2 DCT CKMT1B /// CKMT1A MAGEA3 DKK3 APEH ASAH1 BIN3 TFPI KIAA1102 KIF23 ATRX MAGEA12 CLGN MT1E FDFT1 WTAP FYN S100A4 ID4 ERBB3 SFRS1 F2R PDXK TDRD3 G0S2 MAGEA6 PBK STC2 TFAP2A PMP22 NEDD9 LOC151162 RAGE WIPI49 GSTM4 LGALS3 /// GALIG SGNE1 SLC27A3 CPS1 CADPS2 SILV JMJD1C ANKRD15 DDR1 SERPINB2 SORT1 RAB20 TTC11 S100A10 ABLIM1 MDK TFPI2 ALCAM HMGA1 DRAP1 LTBP2 L1CAM ACO1 HHEX PIR EMP2 MAC30 S100A11 PKIA GPR56 MBP COL4A2 DMD COX17 HMG20B RAB17 BAMBI AOX1 HMGA2 SLC16A6 SERPINE1 SLC1A1 FAT SAT MAP7 COL9A3 FAM46A KIAA1598 KIAA1026 SORBS1 ICMT EI24 BAT2D1 DDIT4 AKAP12 CTDSPL ACOT2 NMT2 PRG1 UBL3 CTNNA1 COCH TRIB2 C1ORF24 AVPI1 C10ORF38 AHCYL1 SART2 FLJ23235 MAGOH ZNF281 TRIM2 ELK3 RFC3 SH3BP5 ST7 TGM2 MAP4K4 NQO1 ATP1A1 GPSM2 PLP1 LMNA CHIC2 BACE2 DUSP4 GAGE5 /// GAGE7 /// HEY1 APOBEC3B RBMXL1 TPD52 SMPDL3A CD9 MATN2 LITAF FOS NRIP1

GYORRFY_MITOXANTRON na S100P MAGED4 PRKCDBP DAPK1 MTHFD2 LAMB1 HDAC4 GAGE2 /// GAGE4 /// MEST TIA1 IMP-3 ISG20 LRP16 CAMK2N1 EGR1 IGFBP7 ACTA2 CGI-116 IRF7 C6ORF1 ACSL3 UCHL1 IQGAP1 C20ORF45 EN2 LGR4 TFF3 DAF ALDH2 MYCBP2 MAGEA3 SEC31L1 EVI5 NEFH PPP1R2 TAGLN SLCO4A1 FDFT1 ADARB1 FYN ENC1 CREBL2 ZNF447 GCH1 RBBP8 LAMC1 MAGEA6 C3ORF14 OXCT1 C3ORF4 IDH3A LIPG KIAA1008 SPON2 SLC7A5 WARS CSPG2 GLIPR1 GPX3 GAGE4 /// GAGE5 /// BST2 KCNAB2 SATB2 PHGDH DYRK2 PBEF1 PROSC FZD2 RCHY1 ASNS BMP7 CUGBP2 EIF2S1 CTH SCHIP1 SET /// LOC389168 ASS CHRDL1 PBX1 IL13RA2 HMG20B SMAD2 SCD RCBTB1 HOXA5 SLC16A6 CLU MXI1 C2ORF31 LOC81558 SPON1 TRIB1 FNDC3A SORBS1 IFITM1 CXADR GAGE3 DDIT4 SALL2 CKLF G1P2 LIPA NR4A2 FOXF2 PLCB4 TPM1 UBL3 P4HA1 COCH RUNX3 CES2 BBX AHCYL1 AGPAT5 TRIM2 CD58 ZFAND1 PLAT UQCRH SRD5A1 ABCA1 IGFBP3 MOCOS GAGE1 /// GAGE2 /// MAGED1 BRP44 GLUL PTPRE NAV2 COL11A1 HLA-DRB1 TMEM47 FLRT3 BACE2 DUSP4 EIF5A GAGE5 /// GAGE7 /// PDE8A NDUFS5 CPVL PDE4D SDC2 APOBEC3B PRDX2 RPL31 TPD52 ZP3 /// POMZP3 LU TNKS SFN BTBD3 RGS2 NRIP1 DHRS2 PLAGL1 HSPA2 NID2 EXTL2

GYORFFY_PACLITAXEL na FANCL DDX23 STC1 MRPS15 GSTP1 SNX10 TIA1 CCNB2 APLP2 HSPC009 CXCL5 BIRC2 ARF3 ACSL1 NDUFA1 DLEU2 /// BCMSUNL UNG PSMB1 NUSAP1 VAMP8 PIK3C2A FGF5 EIF2AK3 FAM3C NDUFS8 BGN DSP DKFZP586K1520 GTF2H3 GALNT7 HCAP-G RPS26 /// RPS26L /// MCAM NDUFB2 FDFT1 FLJ22104 COPE PSMB6 SGCB VCAM1 ZFP36L2 KIAA0152 MRPL40 RAGE ADD3 ATP6V1A NDUFA13 SGNE1 RFXANK SUCLA2 CAT CREG1 IL8 PTGES3 WDR8 NUDT15 GPI MRP63 SERPINB2 RPL10 TIMM13 TFPI2 LTBP2 IDH2 SP140 WDR61 IL13RA2 HMGN3 COX17 AOX1 WSB2 SPTBN1 NDUFB7 SERPINE1 FLJ13576 SLC22A3 PPP1CB HMGB1 ANP32A NUDT4 /// NUDT4P1 HSD17B4 ITGB1BP1 DCTD GUSB RRM2 G1P2 OSBPL10 SNRPA1 PRG1 LAMP2 RHOT2 TMEM59 UBE2L6 DHCR24 C16ORF33 PSMA4 C1ORF63 TRIM2 MMP1 BGN /// SDCCAG33 SC4MOL TDG SKP2 MAPKAPK5 MED4 INSIG1 CDKN2C FLJ21749 NDUFA3 PKM2 COL6A3 APOBEC3B PRDX2 D15F37 /// LOC440248 SRPR CAMK2B PPIB ZP3 /// POMZP3 ALG8 KCNMA1 LITAF ITM2B LDLR

GYORFFY_TOPOTECAN na FLJ10154 MGC4707 MEST TCF8 TIA1 GTF2I /// GTF2IP1 ACTA2 TMEM22 CKAP2 ACAT2 RANBP1 AIM1 DKK3 DSP FRMD4A PTPRK FLNC HNRPDL EZH2 LAMC1 AKT3 FLJ10719 ADD3 LIN7C HMGB2 CAT FLJ22624 RBM35A TNFRSF6B /// RTEL1 ABCD3 TACSTD1 PLOD2 PTX3 RAB13 LIMK2 FGD1 HMGA1 C13ORF7 LOXL2 LTBP2 ANKRD10 DLC1 KRT19 TPK1 LOC161527 CASP6 MAC30 TSPAN8 MBP HMGN3 AOX1 HMGA2 MBD4 SERPINE1 HADHSC TBC1D4 BOP1 SNAPC1 SPAG9 COL6A2 CYB5 ANP32A EVA1 HNRPA3 G1P2 SMARCA4 SAP18 SNRPA1 TUBB6 NAB1 CSTF2T C1ORF24 NOC3L C10ORF38 SCRN1 GCA ALAD FTH1 FGF2 IGFBP3 VIM GALNT1 DKFZP547K1113 TXNIP STOM EVER1 TRPC1 CCNF GALNT3 CAMSAP1L1 RAFTLIN PIN4 FUSIP1 UBE2I XIST COL6A1 SEC14L1 TNFAIP8 UBE1 FANCL STC1 SFRP1 MAP1B FHL1 TUBGCP3 MTUS1 SERPINB1 MCTP2 CCNB2 LEPR /// LEPROT C11ORF32 IGFBP7 BIRC2 QKI NSMAF HTATIP2 ACSL3 UCHL1 UNG TSPAN13 RAB7L1 IL1RAPL1 SRI TST SUV420H1 MKNK2 GJA1 TFPI CCNG2 MCAM C1ORF78 FDFT1 FJX1 BID C19ORF2 ERBB3 C14ORF108 SATB1 GCH1 GMDS GPSN2 AUTS2 RIPK2 CTSL MASA ELL3 HOXB2 RAGE FOXD1 CSPG2 GSPT2 CAMK1 OPTN MLF1IP CDKN1C BRRN1 INHBA RAB20 MRP63 SERPINB2 TFPI2 RDX MACF1 DRAP1 KIAA0274 LOC58486 ASS CLCN3 PSPH PDGFC SLC39A8 COX17 CD24 SCD HDAC9 SOCS6 FLJ10781 H2AFV DNCH2 BCKDHA TCF3 HMGB1 NUDT4 /// NUDT4P1 RGS20 TCF7L2 DDIT4 DZIP1 SPP1 PRG1 NMT2 TPM1 PLEKHC1 SART2 CITED2 SARA1 MYO1D DUSP3 TRIM2 CUL4A SRD5A1 WEE1 MMP1 MAGED1 SC4MOL TGM2 ST7 SKP2 HMGCS1 GPSM2 RPA1 C16ORF34 FUCA1 INSIG1 NUDT4 EIF5A FLJ13910 PDE8A RAI14 NRIP3 CSF2RA FLJ22794 APOBEC3B LOC283232 CDH1 LARP6 KCNMA1 KCTD3 ULK2 ADAMTS1 SLC38A6 HSPA2 PTRF CABYR

GYORFFY_VINBLASTIN na HCLS1 AURKB FANCL PELO DDX23 PRC1 FEN1 SNX10 CDCA8 GTF2I /// GTF2IP1 CCNB2 IMP-3 SOD1 CXCL5 HSPC009 BIRC2 NDUFA1 MME TOP2A NSMAF KIF1B COX5A TSPAN13 DLEU2 /// BCMSUNL ATP5I PSMB1 BIRC3 CKAP2 IL1B PLAU CDK2 NUSAP1 ACAT2 RANBP1 FAM3C LMNB1 DKK3 GMNN HIST1H1C KIF23 GTF2H3 TRMT5 HCAP-G RPS26 /// RPS26L /// MCAM NDUFB2 ID2 YWHAQ /// MIB1 C7ORF24 GPR CENPA KIF15 DERA FKBP1A G0S2 ZFP36L2 LSM7 STC2 RAGE ADD3 NDUFA13 HMGB2 SUCLA2 RFC5 CAT CREG1 AXL BUB1 IL8 CCNA2 PTGES3 HOMER1 TACSTD1 BRRN1 PTX3 CDC2 NUDT15 DIRAS1 MELK INHBA ANKRD15 SERPINB2 MRP63 SORT1 CCNB1 HSPC051 PTTG1 ABLIM1 FLJ14668 TFPI2 GTF3A ATP5J2 HMMR NDUFC2 GTSE1 FOSL1 LTBP2 ANKHD1 FLI1 TMPO MAD2L1 IDH2 LRRC1 FN1 PDHB EMP2 SYDE1 MAC30 PIR PSPH EREG TTF2 WDR61 IL13RA2 DONSON HMGN3 KNTC2 COX17 AOX1 WSB2 DGUOK SPTBN1 KIF20A EDG2 SERPINE1 ZDHHC11 ETFB SFXN1 HADHSC TBC1D4 CTGF COX6B1 SLC22A3 SUCLG1 KIAA1598 ORC6L KIAA1026 HMGB1 NDUFC1 CRIPT NUDT4 /// NUDT4P1 ITGB1BP1 GGCX PGRMC1 RRM2 STRN GUSB KPNA2 C5ORF15 LOC146909 OSBPL10 DKK1 GNPTAB PRG1 DCPS SULT1A1 RAB11FIP1 FLJ23235 C16ORF33 NDUFA7 DCC1 TRIM2 SRD5A1 MYCBP MMP1 TMEM14A TDG SKP2 NDUFS6 THEM2 GPSM2 MAPKAPK5 TTK LOC388650 PSME4 FLJ21749 CDKN2C REV3L RACGAP1 NDUFA3 FLJ13910 KIF11 VEGFC APOBEC3B RPA3 ZWINT ACP1 DFFB NDUFV2 TPD52L1 OIP5 KIF4A

KOHLMANN_ADULT_MLL_LINEAGE_NETWORKS na NUCB1 CASP1 TANK PEX13 VIL2 WT1 SOCS3 PAK3 PIK3C2B IRF4 IRAK1 APLP2 CASP3 PILRA CARD9 RPS6KB1 FASLG PTPRCAP MNDA CDC42 KLRD1 TNFSF10 EMR1 IRF8 SPN PTPN6 ATN1 CCL3 /// CCL3L1 /// SPI1 SYK TRA1 KLRC3 CBX4 CCBP2 IRF1 CD63 TP53I3 KLRK1 INSR CD1D KIAA1579 /// JAK1 TFAP2A VPREB1 ETS2 NCSTN CASP10 TBL1XR1 MLX GTF2B SLC25A29 CBL IKBKG CREG1 FCGR2C LYN GAB2 ZYX PECAM1 CDC2 MSN THRB RB1 PCGF4 CEBPE LTBR SELP TNFRSF25 /// PLEKHG5 ICAM2 ICAM3 KLRC1 /// KLRC2 TRAF4 IGHA2 /// IGHG1 /// IL6ST CDC42EP5 ITGA4 IL13RA2 CASP7 MBP NEK6 IFNGR2 PRKCE ADAM15 BMX VDR SELE ABL1 TRAF3IP2 MYB PRKCA TLR4 IL13RA1 THEM4 TNFRSF13B TP73L MITF CD19 PLAUR NFKBIA DDEF2 TBL1X KIAA1579 CD151 CD14 PARVB EPHB4 HNF4A BCR /// LOC440820 PRDM1 RING1 BCR TNFSF13 HDAC3 KIT PRG4 NPAS2 /// LOC377064 FADD PCSK5 CTSS CD4 LY96 FES PSENEN PARD6A ZNF423 PAX5 TNFRSF10A NPM1 PSEN1 FLJ22814 CSDA CD36 JAK2 PSEN2 CCR3 SPRY2 RBPSUH HERPUD1 SLC12A2 HUMGT198A PTPRC SOAT1 CETP BAG4 TBK1 CFLAR PGD CAPN2 HCK PRKCZ CTNNB1 SEPT7 RPL23AP13 ULBP1 MYC TNFRSF10B TRAF2 JUP CORO2A YY1 IL6R JAK3 RHOH FAS SOCS2 MEF2A IGHD CTRL TNFRSF14 ADARB1 CASP9 NCOR1 LAT BCL6 VAV1 ARHGDIB TNFRSF1A LCP2 AKT1 ZFP36L1 GPS2 TLR1 TNFRSF1B MAP4 VCAM1 TLR2 RIPK1 STK39 CASP8 IL4R IFNGR1 PLD1 NFYC CDKN1B JAM3 STAT1 SHFM3P1 TNFRSF17 CTSZ CCR4 TRAF1 RHOG HRASLS3 NFYB REPS2 SIRPB1 CSF2RB IGF1R GIT2 RARA CRADD PTPNS1 EBF TMEM50B SCD SMAD2 SPTBN1 BLNK BCL3 TOLLIP CD22 /// MAG TCF3 CEP2 SREBF1 CHUK AP1G2 PEX19 HMGB1 ADAM28 SELPLG TNFSF13B VEGF TYROBP CDC42EP3 CD79A RPS6KA1 FCER1G POU1F1 ARHGEF7 MAG ITGAX DRPLA PTPN22 RUVBL1 NR1I2 IGHM IKBKB CITED4 DF CEBPA ARHGEF6 ITGAM SELL CEBPB BCL10 RXRA TRADD CCL4 TTRAP LEF1 BCL11A NCL CSF2RA TNFSF13 /// TNFSF12- GH2 CCL5 CSF3R PODXL TBP CBFA2T3 FCGR2A CCR1 ITGAL ITGB2 TNFRSF25

KOHLMANN_ADULT_DOWN_IN_11Q23_VS_TALL_AND_TRANSLOCATED_PREB na KIAA0882 CASP1 MS4A1 SCHIP1 LOC152485 MAPK14 LCK DOCK9 BCL11B CD3G STK32B GIMAP6 MME NPDC1 CD3D TRBV19 /// TRBC1 CYTL1 IGJ PTP4A3 SH2D1A TRBV21-1 /// TRBV19 FLJ21159 CD52 CNN3 ID3 SMAD1 NGFRAP1 NLGN4X PRKCA CHES1 MAL CTHRC1 FLJ37440 SHANK3 AKAP12 LTB TRAT1 CDC42BPB SYNGR3 SEMA6A

KOHLMANN_ADULT_UP_IN_11Q23_VS_TALL_AND_TRANSLOCATED_PREB na GREM1 DDEF2 PROM1 CLEC14A CACNA2D4 KCTD15 MEIS1 WT1 ABHD4 VLDLR HTRA3 CCNA1 KIAA0960 UPB1 KLRK1 HOXA10 FARP2 ZFHX1B IMP-2 CPEB2 EMB GPM6B PPM1H MORC2 CTGF ASF1A DEPDC1 CD72 PAN3 C20ORF103 KIAA1576 HOXA9 CSPG4 CSRP2 PSD2 QPRT FLT3

KOHLMANN_ADULT_COMBINED_11Q23_VS_TALL_AND_TRANSLOCATED_PREB na KIAA0882 GREM1 CASP1 PROM1 SCHIP1 CACNA2D4 MAPK14 LCK MEIS1 WT1 ABHD4 BCL11B DOCK9 HTRA3 VLDLR KIAA0960 UPB1 ZFHX1B STK32B IMP-2 MME TRBV19 /// TRBC1 NPDC1 GPM6B TRBV21-1 /// TRBV19 PPM1H CTGF ID3 CNN3 SMAD1 NGFRAP1 PRKCA PAN3 CHES1 MAL CTHRC1 FLJ37440 SHANK3 AKAP12 CSPG4 LTB TRAT1 CSRP2 CDC42BPB PSD2 QPRT SYNGR3 MS4A1 DDEF2 CLEC14A LOC152485 KCTD15 CCNA1 CD3G KLRK1 HOXA10 FARP2 GIMAP6 CD3D CYTL1 CPEB2 IGJ EMB PTP4A3 SH2D1A FLJ21159 MORC2 CD52 ASF1A NLGN4X DEPDC1 CD72 KIAA1576 C20ORF103 HOXA9 FLT3 SEMA6A

KOHLMANN_ADULT_DOWN_IN_AML_11Q23_VS_AML na MN1 BAALC MAN1A1 ESCO1 PGDS LOC339162 C6ORF69 CXCL3 CD1C IRX1 DNTT FLJ14054 STK32B NPDC1 HPGD ST18 TRH CYTL1 BCAT1 TPSAB1 CHI3L1 TPSAB1 /// TPSB2 KIAA0746 MEG3 EPX CD96 LOXL1 RUNX1T1 TFPI SHANK3 CLIPR-59 UNQ3033 TPSB2 PTRF FGF13 SLC24A3 RBPMS ITGA9 CPA3

KOHLMANN_ADULT_UP_IN_AML_11Q23_VS_AML na HOXA3 ITGAX LAT2 CACNA2D4 TM2D1 DACH1 MEIS1 H2AFY CPNE8 HOXA10 C1ORF54 HNMT HOXA5 TRPM4 MICAL1 PARP3 KIAA0746 LOC339005 /// LOC440 NIPA1 APOC2 PBX3 DEPDC1 HOXA7 ASCL2 HOXA4 PTN CKM HOXB9 HOXA9 ACRBP C22ORF16 ZNF521 SOCS2 C10ORF54 AK2 CKLFSF4 MGC17301 FBP1

KOHLMANN_ADULT_COMBINED_AML_11Q23_VS_AML na MN1 BAALC MAN1A1 ESCO1 PGDS LAT2 LOC339162 CACNA2D4 MEIS1 IRX1 DNTT STK32B HOXA5 NPDC1 HNMT ST18 TRH MICAL1 CHI3L1 TRPM4 LOC339005 /// LOC440 ASCL2 MEG3 EPX HOXA4 LOXL1 RUNX1T1 CKM TFPI SHANK3 SOCS2 ZNF521 C22ORF16 UNQ3033 C10ORF54 CKLFSF4 SLC24A3 RBPMS FBP1 HOXA3 ITGAX TM2D1 C6ORF69 DACH1 CXCL3 H2AFY CD1C CPNE8 HOXA10 C1ORF54 FLJ14054 HPGD CYTL1 BCAT1 TPSAB1 TPSAB1 /// TPSB2 PARP3 KIAA0746 NIPA1 APOC2 PBX3 DEPDC1 HOXA7 CD96 PTN HOXB9 HOXA9 ACRBP CLIPR-59 AK2 TPSB2 FGF13 PTRF MGC17301 ITGA9 CPA3

KOHLMANN_ADULT_DOWN_IN_ALL_AML_11Q23_VS_AML_ALL_TALL na ESCO1 MAN1A1 PGDS LOC152485 C6ORF69 CXCL3 CD1C IRX1 POU4F1 FLJ14054 STK32B MME NPDC1 HPGD STAB1 ST18 CYTL1 TPSAB1 TPSAB1 /// TPSB2 KIAA0746 SMAD1 CFH /// CFHL1 MEG3 LOXL1 RUNX1T1 TFPI CTHRC1 SHANK3 CLIPR-59 UNQ3033 TPSB2 FGF13 SLC24A3 RBPMS F2RL1 CPA3

KOHLMANN_ADULT_UP_IN_ALL_AML_11Q23_VS_AML_ALL_TALL na HOXA3 LAT2 CACNA2D4 MEIS1 CPNE8 SCPEP1 FARP2 HOXA10 ZFHX1B C1ORF54 HTATIP2 HOXA5 LOC401093 TRPM4 MICAL1 LOC339005 /// LOC440 TDE2L LOC116236 LOC284120 PBX3 DCAL1 MEF2C DEPDC1 MBNL1 HOXA7 HOXA4 C20ORF103 PTN SNX24 PARP8 HOXB9 HOXA9 SOCS2 AK2 LOC440388 FLT3

KOHLMANN_ADULT_COMBINED_ALL_AML_11Q23_VS_AML_ALL_TALL na ESCO1 MAN1A1 PGDS LAT2 CACNA2D4 MEIS1 IRX1 ZFHX1B STK32B MME HOXA5 NPDC1 HTATIP2 ST18 LOC401093 MICAL1 TRPM4 LOC339005 /// LOC440 LOC116236 DCAL1 SMAD1 CFH /// CFHL1 MBNL1 MEG3 HOXA4 LOXL1 RUNX1T1 TFPI PARP8 CTHRC1 SHANK3 SOCS2 UNQ3033 SLC24A3 RBPMS HOXA3 LOC152485 C6ORF69 CXCL3 CPNE8 CD1C SCPEP1 HOXA10 POU4F1 FARP2 C1ORF54 FLJ14054 STAB1 HPGD CYTL1 TPSAB1 TPSAB1 /// TPSB2 KIAA0746 LOC284120 TDE2L PBX3 MEF2C DEPDC1 HOXA7 PTN C20ORF103 SNX24 HOXB9 HOXA9 CLIPR-59 AK2 LOC440388 TPSB2 FGF13 FLT3 CPA3 F2RL1

HOLLEMAN_DNR_B_AND_T na MTX2 SLC11A1 C20ORF149 SHOC2 EIF2B4 CHD4 KCNN4 PCBP2 MED28 ICAM4 DUSP2 GMEB2 WHSC2 ING3

HOLLEMAN_ASP_B_AND_T na DKFZP434O0213 PLA2G4B ARRB2 GALNS SNRPG TRIP3 RPS3 FLJ20647 RPL6 STCH UBOX5 MRPL15 CCNI F8A1 NPHP4 HNRPF MED28 LOC94431 PSME3 NTRK3 PTGER4 DPY19L1 RPL3 GLTSCR2 EFHC1 ARF6 RAB5C DKFZP434C171 BCR DERL2 RPL7A MARCKS FLJ13855 ZNF318 SUMO2 GLDC DEDD RPL11 CCNG2 FLJ10652 TAP2 MTCP1 EIF3S12 FAM46C RCHY1 MICAL3 C14ORF32 MAD2L1BP

HOLLEMAN_VCR_B_AND_T na RY1 EPOR PISD SPATA2 RIPX RPS12 CD44 C13ORF18 RPS15A RBMS1 FKBP1A RPS24 VIM NME2 LSM7 DSC3 ATP13A2 PPM1B CALU RPS2 C6ORF1 RICS TRIM24 KCNN1 PTP4A3 ABHD3 RPL12 HMGB1 RASGRP2 RPL31 RPLP2 YRDC CBFA2T3 HNRPU SCNM1 CREB3L2 RPL23A GM632 KPNA2 TMSB10 RPL10 GNS JMJD2B

HOLLEMAN_PRED_B_AND_T na HRK KIAA0922 SLC11A1 DAPK1 NTAN1 MAGOH CST7 TIMM17A OTUB1 F8A1 U2AF2 HNRPUL1 MED28 POLH POLDIP3 MATN1 CA4 CRKL FAM38A STOM ZNF318 HNRPR ARPC2 SUMO2 KIR3DL2 PCBP1 MCL1 TAF5 MATK AES SMARCB1

HOLLEMAN_DNR_B_ALL na CTDSP2 THBD PCDH9 C20ORF149 SHOC2 NTAN1 EIF2B4 CHD4 CTCF CDC37 KCNN4 PCBP2 RHOA MED28 LARP1 CNTN5 EGR1 GMEB2 ATP1A1 LSM8 ING3

HOLLEMAN_ASP_B_ALL na GATA3 RPL5 ARRB2 MAN1A1 RPS3 H3F3A /// LOC440926 TPM4 RPL6 GNB2L1 H3F3A RPL13A F8A1 ELK3 EIF3S6IP GPR56 FBL EEF1G IGFBP7 NTRK3 RPL3 RPLP0 /// RPLP0-LIKE GLTSCR2 ARF6 DKFZP434C171 RAB5C BCR RPL7A EIF3S7 DERL2 RPL4 FLJ13855 JUP ZNF318 RPL11 CCNG2 HIST1H2BK FLJ12443 RAP2C EEF1B2 EIF3S12 RPS5 FAM46C CLEC11A C14ORF32

HOLLEMAN_VCR_B_ALL na KIAA0882 TCFL5 RPS11 SPATA2 RPS15A C13ORF18 MTMR9 BCL11B ATP13A2 C6ORF1 NFATC1 PDLIM1 S100A13 POM121 /// LOC340318 RPL12 LSM3 YRDC HNRPU SCNM1 CREB3L2 GM632 TMSB10 JMJD2B SH3GL1 RY1 EFNB2 PISD RIPX RPS12 CD44 CRMP1 RBMS1 PNN ZNF304 NME2 VIM ZNF263 SOX11 DSC3 MTF2 CDKN2D PPM1B CALU HLA-DQA1 TRIM24 KCNN1 CHCHD7 PSME4 ABHD3 ARHGAP29 RPLP2 PLCXD1 BTBD3

HOLLEMAN_PRED_B_ALL na KIAA0922 NTAN1 SNX3 OTUB1 F8A1 P2RY5 HDGF HNRPUL1 MED28 CNTN5 PCDHA9 PRPF18 GAPDH POLDIP3 UBE2Q1 BLVRB CDK2AP1 MATN1 CA4 SLC2A3 /// SLC2A14 STOM HNRPR CTCF MAFF CD69 C7ORF26 PVRL2 PSRC1 SUMO2 WAC PCBP1 MCL1 THAP11 AES SMARCB1

LUGTHART_CROSS_RES_BALL na GATA3 FLJ20647 SUMO4 NTAN1 ZNF331 CCNI F8A1 P2RY5 SLC4A1 DSCR1 TNFSF5IP1 DAZAP2 RHOA HNRPF CCNH MED28 MAGED1 PSMD11 PRPF18 MUC4 DHX9 RAB5C YLPM1 CD79B CAPN3 HMGB2 SLC2A3 SHOC2 H2AFZ RPS15 HNRPR CTCF MAFF CD69 ZNF161 SUMO2 MELK PCBP1 WAC WHSC2 IGHD TAF5 AES YWHAQ /// MIB1 NPM1 MAD2L1BP

LUGTHART_VCR_ASP_DISCORD_BALL na PLA2G4B RPS3 RPS11 C1ORF38 C13ORF18 RPS15A SPRY2 H3F3A NUBP1 SMTN STAG3 ISG20 RPS13 EEF1A1 ACOT9 IGFBP7 GLTSCR2 TNS1 RPL24 /// SLC36A2 RPS2 C6ORF1 RPL21 SYPL1 RPL37A JUP RPLP0 FAM11B RPL12 WASF2 ZNF278 TNS TERF2 DCK NACA RPL37 BTF3 MCAM EIF3S12 GALNT2 GATA3 RY1 RPL5 ARHGAP17 VAV1 RPS12 TPM4 CD44 RPS23 RPL18A /// LOC390354 GSN FKSG17 /// LOC389240 FKBP1A EIF3S3 BZW2 RPS2 /// LOC91561 // FBXL5 SMC4L1 RPS25 ZNF443 HNRPA1 /// LOC284387 MGC17330 PTP4A3 RPS17 FMR1 RPS19 RPL11 HIST1H2BK PLCXD1 CLEC11A FAM46C RPS14 RPS8 TCFL5 KIAA0882 RPL14 H3F3A /// LOC440926 ARHGEF18 RPL28 RPL30 GNB2L1 HHEX TCL1A RPS24 RPL10A FBL GPR56 RPL8 RPL19 PTGER4 RPLP0 /// RPLP0-LIKE RPL3 DKFZP434C171 RPS18 EIF3S7 RPL7A RPL4 GPSM3 PTMA NUMB RPL41 RPL35A RPS16 RPL18 RPS6 RPS9 RPL27A /// LOC389435 CENTB2 RPS5 RPL7 EEF2 JMJD2B ARRB2 RPS28 HNRPA1 EIF4B HNRPH3 CYB5R2 FAIM3 RPL6 PRKAR2B RPS7 RPL13A ELK3 UBA52 RNASET2 EEF1G MRPL23 SH3TC1 MGST3 RPS10 C12ORF8 BCR ITPR1 UBIAD1 LOC389223 /// LOC440 FUCA1 CHCHD7 RPL22 EVER1 RPL31 RPLP2 PFDN5 POU2AF1 RPL23A AAK1 RGL1 RPLP1 EEF1B2 RPS21 RPL13A /// LOC283340 RPL24 BAG1 IMPDH2

WEI_DOWN_IN_DEX_RESISTANCE na RPL27A POU5F1 /// POU5F1P1 OR2H2 VIL2 ACOXL DCTN1 LRCH1 C15ORF25 CYP3A4 ADAMTS2 ZNF611 /// LOC388558 LOC200205 DHRS7 AIP DHX9 KIAA1109 SLC35C2 CYB561 MR1 B3GAT3 SCIN ZNF160 PCYT2 TCF3 WDTC1 FAM13A1 NCR1 KIAA0894 SLC9A2 HDGFRP3 MYOZ3 APBA3 DBT CENPB RPS20 MYST4 C14ORF105 SLC4A5 MKI67 OSBPL10 MGC2731 CALCA PAX5 ADAT1 ABHD9 GATAD1 WNT16

WEI_UP_IN_DEX_RESISTANCE na YWHAZ PON2 FLJ13448 CRADD HSPA9B CALM1 NP TMEM50B SLC39A8 C15ORF15 RAB8B UBE2N ZFP36 C6ORF120 RPS6KB1 RAB21 DAF SPAG9 DNAJA1 BZW1 /// LOC151579 C22ORF5 PDE4B OSBP BZW1 RTN4 EIF1AX GNAI3 SLC12A6 DNAJA2 SLC25A37 TFRC METAP2 SNF1LK NR4A2 MCL1 MGC14376 SPRED2 SEC24A PHLDA2 G3BP2 DXYS155E IFRD1 SLC38A2 MAX PLEKHC1 EIF5 BHLHB2 PLAUR LCP2 YWHAE H41 KLHL18 RIPK2 CAPZA2 CD164 MAP2K1 RYBP IFNGR1 GSPT1 NR4A1 ACTR3 INSIG1 CHIC2 SGK SLC2A3 NEU1 PRNP TXNDC4 MAPRE2 NR4A3 DNAJB6 PVRL2 CHSY1 PBEF1 DYRK1A LAT1-3TM /// IMAA // ARF4 SFRS5 EMP1 LOC57228 MAPK1 FOS TSC22D2 CDKN1A RCHY1 ARL6IP FOSL2

WEI_COMBINED_DEX na POU5F1 /// POU5F1P1 VIL2 FLJ13448 CALM1 C15ORF15 ZFP36 SLC35C2 RAB21 RPS6KB1 DAF C22ORF5 EIF1AX GNAI3 RTN4 HDGFRP3 MYOZ3 SLC25A37 CALCA ABHD9 GATAD1 PHLDA2 DXYS155E SLC38A2 IFRD1 RPL27A OR2H2 EIF5 ACOXL LCP2 YWHAE LRCH1 C15ORF25 RIPK2 AIP MAP2K1 RYBP IFNGR1 NR4A1 GSPT1 ACTR3 SGK NEU1 PRNP TXNDC4 NCR1 PBEF1 CHSY1 PVRL2 DYRK1A ARF4 EMP1 TSC22D2 ADAT1 RCHY1 WNT16 YWHAZ PON2 CRADD HSPA9B ZNF611 /// LOC388558 LOC200205 NP TMEM50B SLC39A8 RAB8B NGLY1 UBE2N DHX9 C6ORF120 CYB561 MR1 SCIN SPAG9 DNAJA1 BZW1 /// LOC151579 ZNF160 TCF3 WDTC1 PDE4B OSBP BZW1 FAM13A1 KIAA0894 SLC9A2 SLC12A6 DNAJA2 DBT C14ORF105 MYST4 TFRC MKI67 METAP2 OSBPL10 NR4A2 SNF1LK MGC2731 MCL1 MGC14376 SPRED2 SEC24A G3BP2 MAX PLEKHC1 BHLHB2 PLAUR DCTN1 H41 KLHL18 CYP3A4 ADAMTS2 CAPZA2 DHRS7 KIAA1109 CD164 B3GAT3 CHIC2 INSIG1 SLC2A3 PCYT2 MAPRE2 NR4A3 DNAJB6 APBA3 CENPB RPS20 SLC4A5 LAT1-3TM /// IMAA // SFRS5 LOC57228 PAX5 MAPK1 FOS CDKN1A FOSL2 ARL6IP

KAGER_CURATED_FOLATE_PATHWAY_GENES na ABCC3 MTHFD2 ATIC ABCC2 FOLR2 FTH1 GCH1 ABCC1 SPR ABCG2 AMT MTHFS MTHFR FTCD ABCC4 TYMS ALDH1L1 MTHFD1 FOLR1 SHMT1 PTS ABCB1 /// ABCB4 GGH FOLR3 DHFR FPGS SLC19A1 PPAT MTR ABCB1 ALPI RUVBL2 GART

KOHLMANN_OVERLAPPING_MLL_GENES_FROM_YEOH_ARMSTRONG na HLA-DQB1 THRAP2 SCHIP1 ANXA2 PTPRC BASP1 SERPINB1 DNTT IGFBP7 CD24 MME HOXA5 HBS1L CD22 /// MAG ID3 TCF3 SMAD1 MBNL1 HLA-F LIG4 LOC54103 TERF2 ALOX5 LTB TSPAN7 TNFRSF7 CD44 GAB1 CCND2 CCNA1 PRKCH NPY ADAM10 KLRK1 HOXA10 TES FOXO1A LGALS1 HLA-DMA ECM1 BTK LDOC1 POU2AF1 HOXA9 DDR1 PLXNC1 LARGE RGL1 S100A10 DAD1 CD2AP

BOAG_UP_IN_PREB_VS_NORMAL_CD34 na PCDHGC3 /// PCDHGB4 MARCKSL1 KCTD7 MGAT4A MYRIP SIN3B LCK LOC51321 CALM1 LILRA2 CCL17 RCBTB2 CYLN2 VAMP1 C14ORF113 DCHS1 GPM6B CTGF S100A13 TRAF3IP2 PTK2 JUP WBSCR23 DENND3 ARHGEF4 NPR1 SFRS15 PLVAP CSRP2 CACNB3 DUSP6 TPST1 CHD7 HRK NR3C2 EFNB2 LRRC40 ITIH3 PPP3CC KIAA0913 BMP2 PARD3 PCLO EFNB1 PDGFA DYRK3 PPFIA1 AUTS2 SGCB TFIP11 ITSN1 PLCG1 POU4F1 CEACAM6 PXDN ARHGEF10 LGR5 ETS2 DPEP1 ATG4A PRKD2 C1ORF60 GNA11 SLC27A3 D4S234E KCNK12 ECM1 MORC4 PLCL3 CXORF6 CAMSAP1L1 RAFTLIN RBAF600 DGKD ZNF423 KIR3DL2 LARGE WDFY3 RGL1 TRA@ /// TRDV2 /// T PDE8B RAPGEF3 DLG3 FGFR1 SEMA6A

BOAG_DOWN_IN_PREB_VS_NORMAL_CD34 na CSF2RB FHL2 MDFIC SNX10 MEST PPGB CASP6 ENOSF1 PHACTR2 SERPINB1 EREG FNDC3B PSTPIP2 PDGFC PBX1 ISYNA1 GALNT11 SLC27A2 IGFBP7 C11ORF8 WBP5 MINA DLEU1 KIAA0125 KIAA0182 FAH C2ORF10 MAP7 NGFRAP1 RAB38 ELOVL6 ANKRD27 TMEM76 PAM ASAH1 MAP3K5 FXR1 FAM60A ANXA1 CST3 PPIE IMPACT DEPDC6 MTX2 PTPN22 SPINK2 SCRN1 CD302 PAWR CTSW PARVB FAM45B /// FAM45A EIF3S6IP PDLIM5 RARS HOXA10 LDHB RAB32 PIK3CB CYB5R4 ABCC4 FKBP4 VANGL1 TES MGLL LGALS1 CTSC FCER1A KIT FLJ36166 SSX2IP IL12RB2 NOTCH2 ALDH1A1 MAPKAPK3 RAD50 13CDNA73 NANS HOXA9 ITM2A PLXNC1 BAG2 CRYGD PRSS21 RAP1GDS1 PLAGL1 KIAA1797 DST CPA3

BOAG_COMBINED_UPDOWN_PREB_VS_NORMAL_CD34 na FHL2 MDFIC MGAT4A SNX10 MEST PPGB ENOSF1 LOC51321 SERPINB1 CALM1 LILRA2 CCL17 CYLN2 RCBTB2 IGFBP7 VAMP1 C14ORF113 C11ORF8 DCHS1 MINA DLEU1 KIAA0125 GPM6B PTK2 NGFRAP1 JUP WBSCR23 RAB38 DENND3 ELOVL6 ANKRD27 ARHGEF4 PAM TMEM76 ASAH1 CST3 ANXA1 FAM60A PLVAP PPIE CACNB3 DUSP6 TPST1 HRK NR3C2 DEPDC6 ITIH3 CD302 CTSW PCLO PPFIA1 EIF3S6IP FAM45B /// FAM45A SGCB AUTS2 TFIP11 PDLIM5 RARS POU4F1 PXDN PIK3CB FKBP4 CYB5R4 ETS2 ATG4A TES VANGL1 MGLL LGALS1 SLC27A3 CTSC KCNK12 IL12RB2 ECM1 ALDH1A1 MORC4 PLCL3 CXORF6 13CDNA73 RBAF600 DGKD CRYGD TRA@ /// TRDV2 /// T RAP1GDS1 RAPGEF3 DLG3 CPA3 DST CSF2RB PCDHGC3 /// PCDHGB4 MARCKSL1 KCTD7 MYRIP SIN3B LCK CASP6 PHACTR2 FNDC3B EREG PSTPIP2 PDGFC PBX1 ISYNA1 GALNT11 SLC27A2 WBP5 KIAA0182 FAH C2ORF10 CTGF MAP7 S100A13 TRAF3IP2 NPR1 MAP3K5 FXR1 SFRS15 CSRP2 CHD7 IMPACT EFNB2 LRRC40 MTX2 SPINK2 PTPN22 SCRN1 PPP3CC PAWR KIAA0913 BMP2 PARD3 EFNB1 PDGFA PARVB DYRK3 ITSN1 PLCG1 HOXA10 CEACAM6 LDHB RAB32 ARHGEF10 LGR5 ABCC4 DPEP1 PRKD2 C1ORF60 GNA11 FCER1A D4S234E KIT FLJ36166 SSX2IP NOTCH2 MAPKAPK3 RAD50 CAMSAP1L1 RAFTLIN NANS ITM2A HOXA9 PLXNC1 KIR3DL2 ZNF423 LARGE BAG2 PRSS21 RGL1 WDFY3 PLAGL1 PDE8B KIAA1797 FGFR1 SEMA6A

SCHMIDT_DEX_INDUCED_EARLY na P2RY14 FKBP5 KIF26A BTNL9 PFKFB2 SLA DDIT4 ZFP36L2 SERPINA1 FGR SNF1LK EPPK1 WFS1 SOCS1 LGALS3 /// GALIG MGC17330

SCHMIDT_DEX_REPRESSED_EARLY na HMMR ASPM CENPA CENPE CCNB2 ARPP-21 GIMAP7 PBK DLG7 TOP2A BIRC5 TTK KIF20A CDKN3 CDC20 CENPF KIF11 DEPDC1 NEK2 UBE2C CDCA1 KIF23 MKI67 FLJ11029 C10ORF3 CCNB1 GBP4 FAM72A PTTG1 KIF14

SCHMIDT_COMBINED_DEX_EARLY na P2RY14 HMMR ASPM CENPA CENPE BTNL9 CCNB2 SLA ARPP-21 ZFP36L2 GIMAP7 PBK FGR DLG7 EPPK1 TOP2A WFS1 BIRC5 SOCS1 TTK KIF20A CDKN3 LGALS3 /// GALIG MGC17330 CDC20 FKBP5 CENPF KIF11 DEPDC1 NEK2 KIF26A CDCA1 UBE2C PFKFB2 KIF23 DDIT4 FLJ11029 MKI67 C10ORF3 SERPINA1 CCNB1 SNF1LK GBP4 FAM72A KIF14 PTTG1

SCHMIDT_DEX_INDUCED_LATE na CUGBP2 MAD1L1 FZD8 BIRC3 TNFSF8 HBA2 SCML4 FLJ10970 CDC42EP3 SESN1 S100A8 ANXA1 LOC64744 STAB1 FOXO3A ITGAM LYZ /// LILRB1 TNCRNA S100A9 FCER1G

SCMIDT_DEX_REPRESSED_LATE na E2F7 CDC45L ATAD2 FEN1 MAD2L1 KIF15 TPX2 MAC30 FAM80B RFC3 PFS2 DTL TYMS CHEK1 MCM10 NUSAP1 BUB1 MCM4 FLJ37673 RAD51AP1 BRRN1 ZBTB24 CDC2 MELK RRM2 HCAP-G BRIP1 KIAA0101 E2F8 OIP5

SCHMIDT_COMBINED_DEX_LATE na CUGBP2 E2F7 FZD8 FEN1 MAD2L1 SCML4 MAC30 DTL S100A8 LOC64744 CHEK1 TYMS BIRC3 NUSAP1 HBA2 FLJ37673 MCM4 CDC42EP3 RRM2 HCAP-G ANXA1 BRIP1 KIAA0101 E2F8 FCER1G CDC45L ATAD2 TNFSF8 KIF15 TPX2 RFC3 FAM80B PFS2 STAB1 MCM10 FOXO3A ITGAM S100A9 TNCRNA MAD1L1 BUB1 RAD51AP1 FLJ10970 BRRN1 ZBTB24 CDC2 MELK SESN1 LYZ /// LILRB1 OIP5

SCHMIDT_DEX_REPONSE_TOP62_PROBES na P2RY14 HMMR ASPM CENPA CENPE BTNL9 CCNB2 SLA ZFP36L2 ARPP-21 GIMAP7 PBK FGR EPPK1 DLG7 TOP2A WFS1 BIRC5 KIF20A SOCS1 TTK CDKN3 LGALS3 /// GALIG MGC17330 CDC20 FKBP5 CENPF KIF11 NEK2 KIF26A DEPDC1 UBE2C CDCA1 PFKFB2 KIF23 DDIT4 MKI67 FLJ11029 SERPINA1 C10ORF3 SNF1LK CCNB1 FAM72A GBP4 PTTG1 KIF14

SCHMIDT_UNIQUE_CELL_CYCLE_CLUSTER na HMMR ASPM CENPA CENPE CCNB2 PBK DLG7 TOP2A BIRC5 TTK KIF20A CDKN3 CDC20 CENPF KIF11 NEK2 DEPDC1 CDCA1 UBE2C KIF23 MKI67 FLJ11029 C10ORF3 CCNB1 FAM72A KIF14 PTTG1

SCHMIDT_MTX_RESPONSE_GENES na ATM GRP58 JRKL HIST1H1E MCCC2 ACP2 GJA7 GTF2H2 AKAP7 B4GALT4 ANKRD1 POU4F1 S100A8 IGFBP5 BIRC5 XIST TUBA1 LCN2 ITGB2 RPS14 KLRC3 S100A9

SCHMIDT_DEX_RESPONSE_TOP_CANDIDATES na P2RY14 FKBP5 KIF26A BTNL9 ZBTB16 MYCPBP PFKFB2 SLA DDIT4 ARPP-21 ZFP36L2 GIMAP7 SERPINA1 FGR LGALS3 EPPK1 WFS1 GBP4 SOCS1 MGC17330

KIRSCHNER_SCWABE_ALL_EARLY_VS_LATE_RELAPSE na BIK AURKB ZNF174 KIAA0882 GMPS FOXM1 MAD2L1 MRPL48 ORC1L CDCA8 MGC16943 CGI-121 CCNB2 PP RAD54L HSPD1 KIFC1 SPAG16 DLEU1 AGL ICF45 NUSAP1 ETFA SPBC25 CSH2 MTX1 KIF23 HCAP-G IQWD1 FAM49A SGSH CTNNBL1 KIAA0101 IDS RAD51 PPIE HDLBP COX7A2L CDC45L PSMA4 FLJ10241 SHMT2 MTIF2 KIF15 ESR2 TPX2 FLJ20641 RBBP6 PBK LDHB CTPS MGC17330 RPESP ALG5 RACGAP1 MLF1IP KIF11 MNS1 CBX5 EXOSC9 MPHOSPH6 RAD51AP1 CACYBP SAE1 PRO0149 UBE2C ACP1 MELK SEPT10 SFN TNFRSF25 PTTG1

DIK_DOWN_IN_CALM_AF10_TALL na PRSS3 POLR2E FANCL C10orf70 CSPG6 LMO4 FEN1 ARL6IP4 MPDU1 CENTB1 SDF4 CORO1A NCF4 HLX1 ENO1 BRDG1 ILK PDGFC MCM5 OPN3 C10ORF70 S100A8 FH QKI SPAG16 APRT C12orf8 POLD1 MSRB2 OGFR TNFSF10 SYNGR1 GGH SNCA TYROBP IRF8 PLXND1 KIAA0368 PRPF31 TRA1 MCM2 MRPL18 LSM4 NASP ITM2C CHMP2B GCA CD302 PSMD13 NKG7 C22ORF9 LYZ LY86 AKR7A2 APOBEC3F ARSB FKBP4 UBE2E3 AHSA1 WARS PICALM IGJ PDCD11 C12ORF8 PPP3CA GLIPR1 MCM7 PRSS2 SAH NBS1 BST2 STOM TUBB PKD2 CSF3R BANF1 HOXB9 PROSC TTC11 CDK4 NCR3 UROD MATK CYBA NRIP1 CTSH PLEK NUDT1 IFI16 TFEC SNX15

DIK_UP_IN_CALM_AF10 na SFPQ H3F3B JUNB HNRPA1 AEBP1 TCF8 DDX24 ZC3HAV1 COMMD3 SOX4 CHN2 FTH1 ANXA2 BTG2 C1orf9 GADD45A PIK3R1 PLEKHB2 PSCD1 HOXA10 CAPN2 CLK1 HOXA5 PNMA2 SKIL ZA20D2 RNPC1 INSIG1 PSME4 CDKN1B SLC2A3 SFRS6 PPM1A TUBB CD69 DNAJB6 C1ORF9 YPEL5 DDX3X HOXA9 PIGA PCGF4

DIK_COMBINED_CALM_AF10 na PRSS3 FANCL C10orf70 LMO4 FEN1 H3F3B ARL6IP4 CENTB1 SDF4 TCF8 DDX24 CORO1A SOX4 ILK MCM5 PIK3R1 PLEKHB2 OPN3 S100A8 QKI CAPN2 SPAG16 C12orf8 MSRB2 OGFR SFRS6 TNFSF10 SYNGR1 GGH SNCA IRF8 KIAA0368 PIGA TRA1 MCM2 LSM4 JUNB AEBP1 CD302 COMMD3 LYZ GADD45A APOBEC3F FKBP4 UBE2E3 AHSA1 WARS GLIPR1 MCM7 CDKN1B BST2 NBS1 PROSC TTC11 PCGF4 MATK PLEK NUDT1 IFI16 SNX15 POLR2E SFPQ CSPG6 MPDU1 ZC3HAV1 CHN2 ANXA2 BTG2 HLX1 NCF4 ENO1 BRDG1 PDGFC C10ORF70 FH CLK1 HOXA5 APRT PNMA2 ZA20D2 POLD1 RNPC1 PPM1A TYROBP PLXND1 YPEL5 DDX3X PRPF31 MRPL18 ITM2C NASP HNRPA1 CHMP2B GCA PSMD13 FTH1 C1orf9 NKG7 C22ORF9 PSCD1 AKR7A2 LY86 HOXA10 ARSB SKIL PICALM IGJ PDCD11 C12ORF8 PPP3CA INSIG1 PSME4 SLC2A3 PRSS2 SAH STOM TUBB PKD2 CD69 DNAJB6 CSF3R C1ORF9 HOXB9 BANF1 HOXA9 CDK4 UROD NCR3 CTSH NRIP1 CYBA TFEC

FINE_ASP_SENS_VS_RES na CAMK2D BCL6 HLA-DQB1 SLC12A7 NR3C1 C1ORF21 TCEAL4 CMAH IL2RG SERPINB1 C1orf21 SLA LOC283824 LGR6 PDPN DOCK8 EGR1 PTCH MGC3036 HLA-DQA1 PTPN14 IL24 GUCY1A3 PBX3 ID3 NLGN4X GCET2 TRA@ IGKC DDX3Y LAF4 KIAA1576 PPP1R16B PSCDBP GYPC CD34 EVI2A CXXC5 ATRNL1 DAD1 ASNS BSPRY

FINE_ASP_RESPONSE_GENES na FLJ38482 FARSLB FEN1 TCF8 WT1 ARHGAP26 LOC55831 ACTL6A MGC5576 DERL2 MAP1LC3B ELOVL5 M11S1 PDPK1 FLJ10980 PLAU ACAT2 WNK1 ARL4 MLL5 FDPS ASAH1 TERF2 DHFR SUPT16H ACTC TBC1D15 HMGB3 C21orf33 ERG ZHX1 UBE2R2 HIST1H2BL CBX4 VDAC1 SLC26A6 SLC38A2 CPEB4 PAXIP1L C10ORF118 CEBPG H2AFY WHSC1 DDAH1 PRSS15 DUSP12 FLJ14213 HDAC1 PPARBP FLJ34922 SRP19 MUM1 WHSC1L1 RYBP FLJ21657 MLL3 RAB2 BTG1 HSPE1 TDE2L DUSP10 ZNF512 SLC25A29 LOC222171 C2ORF30 HIST1H2AM PPIG SLC25A13 C14orf1 SFRS2 SF1 ABCA5 MGC3130 FLJ12171 SSNA1 MGC5309 XBP1 RIMS3 TIGA1 SLC22A1LS IGF2R YES1 PGK1 TMPO TNPO1 CUGBP1 TPBG LRP8 VLDLR CICE EPC1 C7 MGC16037 MSX1 FADS1 ZNFN1A1 LOC222136 C9ORF77 RAPH1 SNAPC1 C20ORF22 CDK7 ANKRD11 RAB3GAP1 SLC7A11 PLXND1 SMARCC1 TCIRG1 HIF1A CPR8 SNRPD3 SLC43A1 HNRPA3 SIRT5 HDHD1A HIST1H4C ITGA1 DDX11 KIAA1553 HBXAP MOSPD2 CEACAM5 NASP HNRPA1 CNOT4 CCT3 NR3C1 CST7 SPINL BMP2 LOC157570 PTPRZ1 RFC3 CGGBP1 SLC1A5 PLEKHA1 KLF5 SEC24B WDR19 CDC23 BBC3 MATR3 UPP1 IARS FGG H1F0 ZNF148 XRCC5 ALB GALNT3 TMEM77 CNOT10 PAICS WDR26 CD9 CSNK1A1 HP1BP3 LPIN1 PAPD5 SCD4 KIF21B POLE LAS1L AKAP9 DDX23 C20ORF18 KIAA1212 JDP2 TUBA3 MTHFD2 NME1 ATF5 TUBGCP3 PHB CAPN1 NARS MCM5 TEX27 PXK NSMAF KLRC2 MARS UBTF ACSL3 MED9 CUTL1 TESK1 ID3 ZNF261 GRB2 TST PSMB2 FLJ21865 STK32C C21ORF34 ATP5G3 CARS LOC201191 NOLC1 PIK3CA PPM1F FAS BRI3BP OSBPL8 ZNF297B PCK1 DNCI2 SLC13A5 KIAA0828 BID FAM44A IFRD1 BCHE ZNF24 CDW52 SQSTM1 LOC152485 ANKRD12 HSPCA PPIC GOT2 STRN4 DKFZP564O1863 TUBA1 CHTF18 ZNF500 WARS ING3 PPIL2 PRKY PRNP KIAA1128 TIMM44 RABL5 ECM1 HIST1H2AC DTNBP1 DIRAS1 C20orf18 THRAP4 DDR1 DYRK1A NUP205 SLC7A1 C8orf13 C14orf24 RCHY1 ODC1 ASNS ATP5J2 FUBP1 D2LIC TUBE1 ASS FNDC3B HSPA9B C14ORF24 FH VPRBP C10orf7 SEC63 ITGB4BP KIAA0182 ACAD9 FAM96B HIST1H2AL ZNF131 PRDX3 ATG16L2 AGXT2 E2F6 HSPA4 TLE1 HIST2H2BE CDC42EP3 BAT2D1 HSPC049 ANP32E HIST1H2BC SPOP RNF5 SWAP70 DMPK CENTB2 ARIH2 HLA-E MCM3 GARS RASA2 TCF15 ABCA1 UMPS SLC35B3 MNAB PPHLN1 FLJ10815 SC4MOL LDHB BHLHB3 FAM62B WDR22 ANAPC7 C20orf155 ATP6V1F KRAS2 PAG1 HN1 SNAP29 TUBB CLCN7 NUDT6 ACLY SLC25A1 NR1D2 IRS2 USP30 HA-1 PPP2R5E MATN2 ZNF703 BAALC NUCB1 MDH1 ATF3 JMJD2A LOC285533 KCTD10 C8ORF13 MRPS27 MTHFD1L MGC3101 FLJ13912 MGC5306 LGALS3BP ZSWIM6 EIF4EBP1 GPC1 UXS1 BDNF SIAT9 ASH1L PFAS KARS MST1 C8A JUND LMNB1 RTN4 PAM LSM3 TBC1D14 BTBD4 TM4SF12 ADAM9 STK19 LOC158427 POLR1A ALPI FYN C20ORF155 DTNA GALNT2 GABRA2 MYH10 HIST1H3D GAB3 SFRS1 HKE2 SAA4 TACC1 FLJ11773 P2RY5 CAPN7 PEG3 SUI1 WBSCR22 MTF2 KIAA0152 AGTR1 FLJ12688 CGI-37 CDKN2D NFE2L1 HOMER3 CIDEB GSTM3 POLK C9ORF76 COMMD5 CHORDC1 FALZ FKBP5 PMAIP1 SYNC1 BST2 LOC285958 PRPS1 HIST1H2BD PDCL ASAHL TERF1 HIST1H2BK SLC38A1 SPAG1 SESN2 PEX12 NUTF2 SET07 ARID4B MAN1A2 SFRS3 KCNG1 SFRS2IP KLF6 FLJ20373 MAD2L1 PFKL CLIC4 IL6ST DATF1 HAX1 P2RX4 VPS37A ATP6V0E FADS2 SFTPB NDRG3 ZBTB12 ST18 C1ORF119 OSR1 NT5C3 DCLRE1C TMEM39A UBE2W OSTM1 PTMA RBBP7 SMC1L1 EIF2S2 G22P1 NEK1 UHMK1 EIF4EBP2 GLS CAP2 BACH1 DDX49 SERPINE2 HIST1H2BF C10ORF46 CTDSPL CTNNBL1 SARS TMEM55A NTHL1 SETMAR YIPF6 C1ORF24 HIST1H4B ATXN2L NOLA2 HSPA8 MTO1 RALA CHMP2B SHMT2 WDFY1 ZNF281 TRIM11 SDCBP SBP2 VIM SH3BP5 PSAT1 ARRB1 AHCY CTPS GNG12 FLJ12716 FLJ39075 HIBCH CHPF ALG2 CDR2 PHIP KIF11 GALNACT-2 OAS3 YARS HNRPR MPG ING1 GATAD2B TAF3 SERTAD1 AKR1B1 SMARCE1 ATXN7 DDIT3 TCEA1 WDFY3 KIAA1999 RBM25 PGM2 DNAJB14 SUMO3 SEL1L IDH3G ADSS KBTBD11 SGTB ATF4 MDFIC GLO1 SFRS11 TALDO1 PRIM2A SPRY2 ELMO1 HERPUD1 SF3A1 RARRES3 SEH1L EIF3S4 MGC15407 PP2447 PP SMAD7 TIMP1 RAD18 PCK2 LOC149603 RGS16 EMX2 KBTBD4 ACIN1 EP300 XPOT MCM4 DNAJC1 EIF1AX RB1CC1 NDUFB9 DNAL4 DNAH11 VprBP AMH TAF9L FAM44B SCCPDH IGFBP2 SSB3 SIRT2 S100A4 DMWD CREBBP CHD2 MTMR6 ZNF403 THRAP1 ARHGDIB NUP93 STARD4 ALDH18A1 RAB1A GC20 ZNF518 RARS PSME3 RNF20 DKFZP761M1511 SLC7A5 MTHFD1 AKT1S1 NEK3 C21ORF33 SGK FLJ38663 GOT1 NCE2 MIER1 SGPP1 ASF1B MAP2K1IP1 APP KIAA1432 CD34 JMJD1C APPL PYCR1 RHOG ICAM1 SLC39A10 HSPA1L MTMR4 ARFGAP3 C1orf24 KIAA1961 ARL10C KPNA4 TRAFD1 NCOA3 PRDX6 EXOSC2 TNKS2 DPP8 LOC153222 TULP3 LGALS8 M17S2 MGC2747 ZNF564 ZNF672 ACY1L2 TUBG1 ZNF566 C5orf13 SOCS7 LDHA ARMCX3 C14ORF1 FKBP8 FLJ21901 SAT UGCG HPRT1 SRP72 SLC2A14 AOC3 MORF4L1 ZCCHC10 CPM ROCK1 CHEK2 FECH JMJD2B ZNF134 SLC3A2 TTC7A BBX CPXM S100B TRIM23 GEMIN4 PPP2R1B JUN LOC51668 APOH MFI2 BRD4 KIAA1959 NRM RNF13 FLJ13213 ARHGEF10 CYTL1 C16ORF34 C10ORF7 C5ORF13 GUCY1A3 ENPP2 GRP58 HELZ SBDS MAP2K3 ARNT PRDX2 RGS12 RBM12 STMN1 SLC4A5 REST LOC221143 PARP1 DNAJB9 APG12L RBP3 ACTB BRD2 KIAA0863

FINE_ASP_SENS_VS_RES_SAM_SUPPL na C3ORF23 RFXDC2 ASB8 UBE2G2 HOXB5 EIF4E3 ZC3H11A SEC61A1 APTX BTBD2 APLP2 TARS PIP5K3 ZNF262 NSUN2 CNOT6L EIF2AK2 GOSR2 LNK DHFR CHML NY-REN-58 JMJD2C EIF2S3 CPSF2 ILF3 VAMP2 DERP6 SLC38A2 NCOR1 TMED7 N4BP1 POLM MGC17943 CAPZB NRBP UBE2D3 LOC144871 GOT1 EEA1 LOC339287 LOC285505 LOC91137 SLC7A1 SPG4 PHF14 ASNS KAAG1 ATP13A1 PFKM ARFGEF2 KLF6 NCOA3 ITCH LOC161527 TMED10 CLIC4 YME1L1 EBF YY1AP1 SLC39A8 ASXL2 ACY1L2 DCP2 BCS1L TOR1A FLJ10374 JMJD3 PRKCA SLC2A14 IGKC EDD1 ZNF335 SLC30A1 FLJ13220 RNF25 ENSA TXNDC10 SPRED2 SEC24A KCTD4 AP1G1 LOC51668 PIGO H41 PCBP2 BRD4 FLJ12529 WRNIP1 KIAA1109 TP53RK SHC1 LOC114227 TncRNA TNCRNA PRKRIR DDEF1 BCL10 STX17 FLJ20308 BTAF1 SLC2A3 FAF1 CDC42BPA RIOK3 KIAA0553 PTGFRN PAG1 LOC90799 GTF2F1 WDR68 LARP1 RAB5A WAC STK6 TXNRD2

ANDERSSON_B_ALL_VS_NBM_NORMAL_HSC na LGALS1 TERT CTGF TMED6 ZNF588 GAB1 SELPLG GUK1 RAB33A PSMA6 KHDRBS3 PXDN HOXB2 CSRP2 HARS2 BLNK DPEP1 SCML2 PILRB

ANDERSSON_T_ALL_VS_NBM_NORMAL_HSC na TUSC3 MLLT11 CHI3L2 NPC2 FAM50B FXYD2 ZMYND10 MAPRE1 PSMA6 ALDH1A2 ACTA2 CD99 EPHB6 TSPAN7

ANDERSSON_AML_VS_NBM_NORMAL_HSC na PRRX1 GLB1 DCX DPH2L1 NAP1L2 MYST3 ZNF544 PPGB ZNF135 MRPL33 MAP1B CKM

ANDERSSON_TCF3_PBX1_VS_BALL na MAD2L2 LOC147166 SNX3 FGF9 APBB2 PRKCB1 IRF4 GOLGA3 SESN1 EXTL3 ABI2 NFATC4 SLC27A2 AOX1 GLI3 ADARB1 PILRB TRIB2

ANDERSSON_IGH@_MYC_VS_BALL na TRPM2 NUCB2 HLA-E SMARCA1 ST6GAL1 LAIR1 SORBS3 EIF4B SHMT2 PPM1G SLC16A1 GAB2 MGC24039 FKBP4 SNTA1 GPR30 LTBP1 DPEP1

ANDERSSON_ETV6_RUNX1_VS_BALL na PTPN18 PRSS3 ITPR1 TUSC3 KIT TMED6 E2F6 ADK GNG11 SCARB1 CD9 PP C21ORF2 ACAT1 TNK1 RGS16 PILRB MDK DAPK2

ANDERSSON_HYPERDIP_VS_BALL na NOV PCDH9 EIF5 SCML1 ANKHD1 TRIM10 GALNT3 ACYP2 HNRPH2 LOC387882 DTX1 TTC3 SH3BP5 PPM1F ITSN1 PRPS2 CAPN2 ZNF185 SUMO3 PHKA2

ANDERSSON_NK_VS_BALL na PSCD4 VBP1 MAD2L1 INPP4A SLC25A5 MAF

ANDERSSON_P190_BCR_ABL_VS_BALL na CCNH SH3BP5 P2RX1 TNK1 NEK3

ANDERSSON_MLL_VS_BALL na LGALS1 MAP7 TBL1X PLA2G4A PWP1 GAS2 PRSS12 SERPINB1 YKT6 TP53I3 HCP5 GNG11 IMP-2 C1ORF34 ZNF135 TCF4 RGS16 EFNA1 ANK2

ANDERSSON_MLL_VS_AML na ANXA3 IRF5 LHFPL2 SMPDL3B MEIS1 SPRY1 LTF SSPN C2 PIP5K1B PCGF2 TFF3 PBX3 IGHV1OR15-1 KIT ZNF544 CAMP DEXI AKR1C3 MGMT INHBA NEDD4 WDR9 EVI2A COMTD1 PTK7

ANDERSSON_MLL_VS_BALL_AND_AML na LGALS1 C2 TFF3 PBX3 KIT IGHV1OR15-1 ZNF544 LHFPL2 PLA2G4A C10ORF74 MEIS1 SPRY1 IL17R IFIT2 INHBA TEGT SEPP1 IMP-2 EVI2A CKB CFHL1 DLK1 PTK7
